# Evolutionary dynamics and molecular epidemiology of *West Nile virus* in New York State: 1999–2015

**DOI:** 10.1093/ve/vez020

**Published:** 2019-07-21

**Authors:** Sean M Bialosuknia, Yi Tan, Steven D Zink, Cheri A Koetzner, Joseph G Maffei, Rebecca A Halpin, Emmi A Mueller, Mark Novotny, Meghan Shilts, Nadia B Fedorova, Paolo Amedeo, Suman R Das, Brett Pickett, Laura D Kramer, Alexander T Ciota

**Affiliations:** 1 The Arbovirus Laboratory, New York State Department of Health, Wadsworth Center, 5668 State Farm Road, Slingerlands, NY, USA; 2 Department of Medicine, Vanderbilt University Medical Center, Nashville, 1161 21st Street, Nashville, TN, USA; 3 J. Craig Venter Institute, Virology, 9605 Medical Center Drive, Rockville, MD, USA; 4 Department of Biomedical Sciences, State University of New York at Albany School of Public Health, Rensselear, NY, USA

**Keywords:** West Nile virus, viral evolution, intrahost diversity, displacement

## Abstract

Following its introduction into New York State (NYS) in 1999, *West Nile virus* (WNV; *Flavivirus*, *Flaviviridae*) underwent a rapid expansion throughout the USA and into Canada and Latin America. WNV has been characterized as being evolutionarily stable, with weak geographic structure, a dominance of purifying selection and limited adaptive change. We analyzed all available full-genome WNV sequences, focusing on the 543 available sequences from NYS, which included 495 newly sequenced 2000–15 isolates. In addition, we analyzed deep-sequencing data from 317 of these isolates. While our data are generally in agreement with the limited pace of evolutionary change and broad geographic and temporal mixing identified in other studies, we have identified some important exceptions. Most notably, there are 14 codons which demonstrated evidence of positive selection as determined by multiple models, including some positions with evidence of selection in NYS exclusively. Coincident with increased WNV activity, genotypes possessing one or more of these mutations, designated NY01, NY07, and NY10, have increased in prevalence in recent years and displaced historic strains. In addition, we have found a geographical bias with many of these mutations, which suggests selective pressures and adaptations could be regional. Lastly, our deep-sequencing data suggest both increased overall diversity in avian tissue isolates relative to mosquito isolates and multiple non-synonymous minority variants that are both host-specific and retained over time and space. Together, these data provide novel insight into the evolutionary pressures on WNV and the need for continued genetic surveillance and characterization of emergent strains.

## 1. Introduction

*West Nile virus* (WNV; *Flavivirus*, *Flaviviridae*) is a single-stranded positive sense RNA virus with a genome of approximately 11 kb encoding a single open reading frame (ORF) comprised of three structural genes (*C*, *prM*, and *E*) and seven non-structural genes (*NS1*, *NS2A*, *NS2B*, *NS3*, *NS4A*, *NS4B*, *NS5*) (Brinton 2002; Mukhopadhyay et al. 2003). WNV is the most geographically widespread arbovirus in the world and the most prevalent arbovirus in the USA. Internationally, the virus is comprised of up to five proposed lineages, which differ by 20–25 per cent nucleotide identity (Chancey et al. 2015). All US strains belong to lineage 1A. Like other arthropod-borne flaviviruses, including *Zika virus* (ZIKV), *yellow fever virus* (YFV), *dengue virus* (DENV), and *Japanese encephalitis virus* (JEV), WNV is responsible for a considerable public health burden in areas where it is endemic. In the USA, WNV has been diagnosed in over 50,000 individuals, including approximately 24,000 reports of neurologic disease and 2,200 mortalities (CDC 2015). Given that most infections are subclinical and most West Nile fever cases go undiagnosed, it is estimated that there have been over three million infections in the USA since the first reports of WNV in Queens, New York in 1999 (Busch 2006; Peterson, Macedo, and Davis 2006).

WNV is maintained in an enzootic cycle between primarily *Culex* spp. mosquitoes and birds, with mammals including humans generally representing dead-end hosts due to insufficient viremia levels for transmission to mosquitoes. Although the magnitude of avian viremia is highly variable (Komar et al. 2003), more than 300 species are known to be competent amplifying hosts (CDC 2015). Following its introduction to New York State (NYS) in 1999, WNV underwent a rapid westerly expansion across the USA, reaching California by 2002. By 2003, WNV activity was reported throughout the lower 48 states, as well as into Canada and Latin America (Kramer, Styer, and Ebel 2008).

Naïve hosts and highly competent vectors allowed for rapid expansion throughout the Americas, yet some evidence for additional adaptive change in the US strains has been noted. The invasive genotype included a positively selected mutation in the WNV *NS3* gene, resulting in a T249P amino acid substitution in the helicase protein which is associated with human disease outbreaks globally and has been shown to increase avian virulence (Brault et al. 2007). In addition, in concert with the western expansion of WNV, a new genotype, WN02, rapidly displaced the NY99 genotype (Ebel et al. 2004). The WN02 genotype is characterized by a single valine to alanine amino acid change in the *E* protein, which has been shown to enhance vector competence in *Culex* spp. mosquitoes, particularly *Culex tarsalis* which flourish in the agrarian environment of the Midwest (Moudy et al. 2007). An additional genotype, SW/WN03, characterized by positively selected sites K314R in the *NS5* protein, and A85T in the *NS4A* protein, has been shown to be circulating since 2003 (McMullen et al. 2011).

Despite these notable mutations, WNV has generally been characterized as being evolutionarily stable, with a dominance of purifying selection and limited adaptive change. Previous phylogenetic studies utilizing global or US isolates have suggested a lack of strong geographic structure or association of specific genotypes with mosquito prevalence or human disease. Studies utilizing more geographically focal datasets (Goddard et al. 2002; Vaidyanathan and Scott 2007; Amore et al. 2010; Ehrbar et al. 2017; Nelson et al. 2018) have indicated that significant diversity exists on regional scales, yet the phenotypic importance of this variability is unclear. Estimates of evolutionary rates in the USA have ranged from 3.6×10^−4^ to 8.2×10^−3^ substitutions/site/year, which stand in contrast to the evolutionary potential resulting from the high replication rates and lack of proofreading for RNA viruses (Drake and Holland 1999; Snappin et al. 2007; Bertolotti et al. 2008). Despite consensus genome stability, WNV, like all RNA viruses, exists within individual hosts and vectors as a diverse set of genomes collectively referred to as the mutant swarm. Extensive studies have demonstrated the importance of minority variants in viral fitness, virulence, and adaptability (Grubaugh and Ebel 2016; Grubaugh et al. 2016; Ehrbar et al. 2017; Nelson et al. 2018), yet large-scale assessment of intra-host variants in nature is generally lacking (Jerzak et al. 2005; Ehrbar et al. 2017).

In order to provide a more comprehensive and updated characterization of the molecular epidemiology and evolution of WNV, we sequenced 495 WNV isolates from NYS and subsequently analyzed all available full-genome WNV sequences, focusing on the well-represented sequences from NYS. We also analyzed intra-host variability from 317 of these 495 newly sequenced isolates which had adequate read depth across the genome. These data provide novel insight into the extent of genetic diversity of WNV in NYS and throughout the USA, the temporal and geographic structure of WNV evolution, and the relationship of WNV genetics to epidemiological patterns. In addition, we have identified a number of novel sites with evidence of positive selection and minority polymorphisms shared over time and space which are biased for vertebrate or invertebrate hosts.

## 2. Results

### 2.1 WNV surveillance

**Figure 1.**
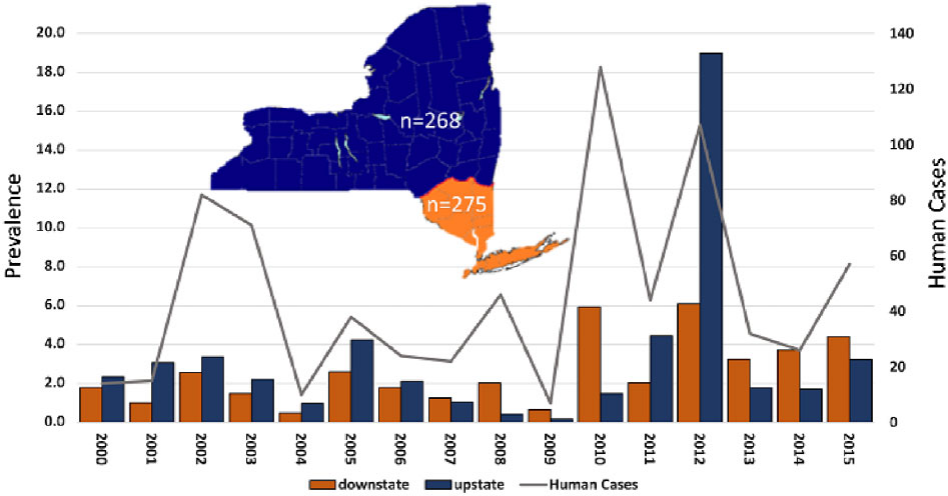
Regional West Nile virus prevalence in *Culex pipiens* and *restuans* mosquitoes in New York state and total diagnosed human cases. MLE refers to maximum likelihood estimate of prevalence in mosquitoes (no. of positive/1000 tested). The number of isolates sequenced from each region (n) is shown on the map.

The prevalence of WNV-positive *Culex* spp. mosquitoes (no. of positive/1000) was calculated using maximum likelihood estimate (MLE) (https://www.cdc.gov/westnile/resourcepages/mosqSurvSoft.html) determined for individual transmission seasons (late May–early October) for upstate NYS counties (including Albany, Broome, Cattaraugus, Cayuga, Chautauqua, Chemung, Clinton, Cortland, Erie, Essex, Fulton, Genesee, Greene, Herkimer, Jefferson, Lewis, Livingston, Madison, Monroe, Montgomery, Niagara, Oneida, Onondaga, Ontario, Orleans, Oswego, Otsego, Rensselaer, Richmond, Saratoga, Schenectady, Schoharie, Schuyler, Seneca, St. Lawrence, Steuben, Tompkins, Warren, Washington, and Wyoming counties) and downstate NYS counties (including Bronx, Columbia, Dutchess, Kings, Nassau, Orange, Putnam, Queens, Rockland, Suffolk, Ulster, and Westchester counties) (Fig. 1).

Mean WNV prevalence in *Culex pipiens/Culex restuans* from 1999 to 2015 was 2.8 positive mosquitoes/1,000 tested, with modestly higher proportions upstate (3.2) relative to downstate (2.6). Substantial temporal and geographic variability was identified, with values ranging from 0.7 to 7.4 downstate and 0.2 to 19.0 upstate. Although prevalence in upstate and downstate are often disparate, the highest values in both regions were measured in 2012. Prevalence in mosquitoes is highly predictive of human cases [Pearson’s correlation, *r* = 0.77, *P* = 0.0004 (Veksler, Eidson, and Zurbenko 2009)] in both regions yet the spillover threshold (defined here as prevalence in mosquitoes/reported cases) is approximately 10-fold higher upstate (0.61 vs 0.062), which could be partially explained by this region having a less dense human population. Additionally, there is a significant correlation between human cases and mosquito prevalence (MLE) for both upstate (Pearson’s *r*^2^ = 0.84, *P*< 0.0001) and downstate (Pearson’s *r*^2^ = 0.57, *P* = 0.0007) mosquito prevalence.

### 2.2 Comparative sequence analyses

The global dataset consisted of 1,886 taxa including all publicly available full-genome sequences with complete metadata. This includes 941 sequences from US isolates, including 543 from NYS. Of the 543 isolates NYS sequences, 287 were obtained from birds, 180 were obtained from *Culex* species mosquitoes, 65 from non-*Culex* mosquitoes, 7 from non-human mammals and 4 from humans (Table 1). The non-*Culex* mosquito isolates were derived from *Aedes*, *Anopheles*, *Coquillettidia*, and *Culiseta* genera. Suffolk County is the most highly represented with 73 isolates (13.4%), followed by Nassau County (43, 7.9%), Onondaga County (42, 7.7%), Erie County (35, 6.4%), Rockland County (32, 5.9%), and Westchester County (31, 5.7%). All other counties were represented by 30 or fewer isolates. These isolates spanned 16 years from 1999 to 2015. Although overall numbers of mosquito and avian isolates are similar, pre-2010 isolates are predominately avian whereas 2010–5 isolates are predominately mosquito derived. NYS isolates were further geographically classified as upstate (268) and downstate (275) based on the county of origin, described above.

**Table 1.** Summary data for New York State West Nile virus isolates sequenced in this study.

Year	Mosquito	Avian	Other	Total
1999	1	3	2	6
2000	5	2	0	7
2001	6	8	2	16
2002	28	35	3	66
2003	26	29	3	58
2004	9	49	0	58
2005	14	38	0	52
2006	15	34	0	49
2007	12	33	0	45
2008	13	25	0	38
2009	11	22	0	33
2010	13	7	0	20
2011	24	0	1	25
2012	32	2	0	34
2013	26	0	0	26
2014	5	0	0	5
2015	5	0	0	5
Total	245	287	11	543

### 2.3 WNV phylogeny

**Figure 2.**
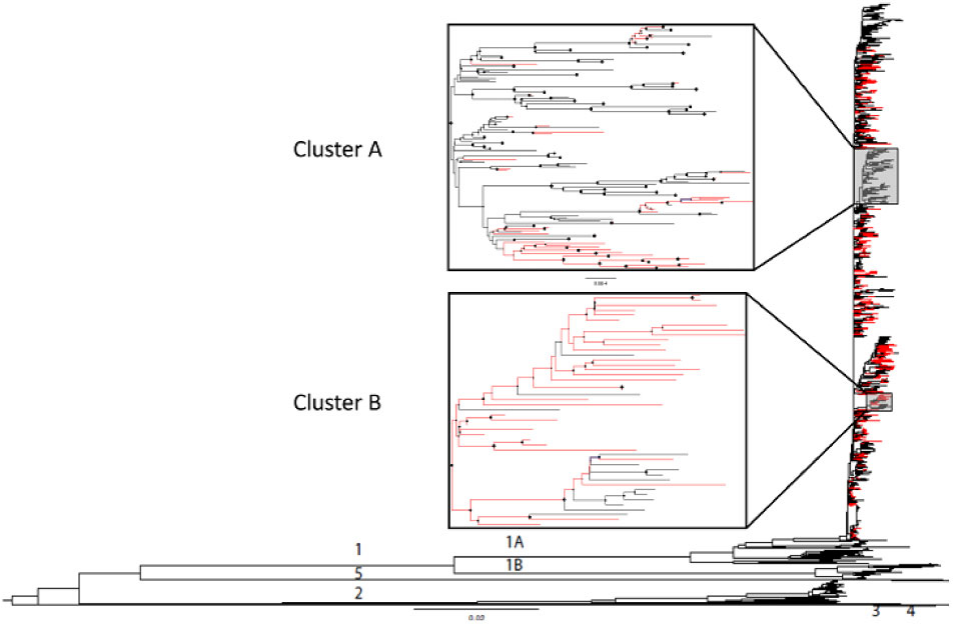
Maximum likelihood phylogeny of WNV open reading frame based on the global dataset of available sequences including 543 strains isolated in NYS (in red). Previously identified lineage designations are depicted as 1A, 1B, 2, 3, 4, and 5. Clusters A and B are well-supported US clades (posterior support >0.75).

The maximum likelihood (ML) tree obtained from the global dataset shows the monophyletic nature of the lineage 1A US strains, as well as the more divergent lineages distributed globally (Fig. 2). NYS strains are widely distributed throughout lineage 1A, illustrating a general absence of broad temporal or geographic structure. This lack of strong structure was consistent with a lack of highly supported clades, with two exceptions, designated Cluster A and Cluster B (posterior probabilities of 0.99 and 0.82, respectively) within the phylogeny of the US dataset (Fig. 2). The only shared amino acid change in Clade A is the WN02 V449A mutation, while strains in Clade B share several amino acid differences. Specifically, 93.3 per cent of the sequences in this clade contain T1195I, 93.3 per cent contain H1262Y, 97.8 per cent contain S1839T, and all contain S287I.

### 2.4 Selection analyses and amino acid variability

Average nucleotide divergence among sequences was 0.02 and 0.005 per cent from the global and NYS only datasets, respectively, while average amino acid divergence was 0.02 and 0.002 per cent, respectively. The most divergent sequences had 0.16 and 0.01 per cent nucleotide differences for global and NYS datasets, respectively. Calculated d*N*/d*S* (number of non-synonymous to synonymous mutations per non-synonymous/synonymous sites) were 0.08 and 0.06 for global and NYS datasets, respectively. Number of non-synonymous mutations were similar for vertebrate and mosquito isolates. This is consistent with a dominance of purifying selection, though the strength of selection was somewhat variable among genes. In the NYS dataset the highest d*N*/d*S* (0.48) was observed in the capsid gene. The other structural genes, specifically the *prM* and *E* genes, had d*N*/d*S* values of 0.08 and 0.07, respectively, indicating greater purifying selection. Among non-structural genes, *NS4B* had the highest d*N*/d*S* (0.14), while the most conserved genes were *NS3* and *NS5* (0.05 and 0.06, respectively; Supplementary Table S3).

A total of 31 non-synonymous consensus mutations were shared among at least 2.0 per cent (>10) of the NYS isolates (Table 2). The majority of these (*n* = 25/31) occurred in non-structural genes, particularly in the *NS2A* and *NS2B* genes (*n* = 10) as well as *NS4A* and *NS4B* (*n* = 8). Ten of these thirty-one mutations demonstrated evidence of positive selection in the US dataset by at least two models (Supplementary Table S1). Resulting amino acid substitutions at these positions include S36N/G [*C* S36N/G], H1262Y [*NS2A* H119Y], K1331R [*NS2A* K188R], A1367T/V [*NS2A* A224T/V], V2259M [*NS4A* V134M], K2297R [*NS4B* K23R], I2513M [*NS4B* I238M], K2842R/E [*NS5* K314R/E], R2950K [*NS5* R421K], and R3074S [*NS5* R545S]). An additional two positions showed evidence of positive selection (d*N* >d*S*), but mutations at these sites were not identified in >2.0 per cent of isolates. These include V721A/I/F [*E* V431A/I/F] and E2522G/K [*NS4B* E248G/K]. When considering NYS strains independently two positions (A2209S/T/V [*NS4A* A85S/T/V] and G2377E [*NS4B* H103Y]) showed evidence of positive selection by at least two models (Table 2; Supplementary Table S1). Amino acid position 2209 (*NS4A* 85) was found to be highly polymorphic, with four different amino acids identified at this residue. Included in this is *NS4A* A85T, identified as a defining mutation in the SW/WN03 genotype (McMullen et al. 2011). We identified this mutation in NYS in 2002, prior to its isolation in the Southwest USA.

**Table 2.** Shared non-synonymous mutations identified in ≥2.0 per cent of West Nile virus isolates from New York State, 2000 − 15.

NT	AA	Gene	Frequency	Years	Variant	Selection
G301A	S36N	*C*	0.02	02-12	G	^a^
A408G	K104R	*C*	0.02	06-14	E	
T459C	V121A	*C*	0.02	08-13	–	
T915C	M273T	*M*	0.02	04-14	–	
C1443T	V449A	*E*	0.90	02-15	–	
T1501A	L468Q	*E*	0.02	01-03	–	
G2893T	K932N	*NS1*	0.02	07-10	R	
C3681T	T1195I	*NS2A*	0.04	07-15	–	
A3794G	M1233V	*NS2A*	0.05	01, 05-07, 09-10	A/L/T	
C3809T	L1238F	*NS2A*	0.04	03, 06-15	–	
C3881T	H1262Y	*NS2A*	0.03	02-04, 06-08, 11, 15	–	^a^
G4089A	R1331K	*NS2A*	0.11	06, 09-15	–	^a^
G4196A	A1367T	*NS2B*	0.04	00-04, 08	V/T	^a^
T4515C	M1473T	*NS2B*	0.02	05-08	–	
G4540T	I1481M	*NS2B*	0.02	03-05, 08-09	V	
G4574A	V1493I	*NS2B*	0.02	01-08	–	
G4577A	V1494I	*NS2B*	0.02	06-14	–	
A5152T	E1685D	*NS3*	0.04	03-04, 11-13	–	
T5612A	S1839T	*NS3*	0.05	07, 09-15	F	
G6722A	A2209T	*NS4A*	0.10	02-12	S, V	^b^
G6872A	V2259M	*NS4A*	0.02	03-05, 07, 09-10	–	^a^
G6957T	S2287I	*NS4B*	0.05	07, 09-15	–	
A6987G	K2297R	*NS4B*	0.03	03-06	–	^a^
G7003A	M2302I	*NS4B*	0.02	05-06, 09, 13	–	
A7058G	T2321A	*NS4B*	0.02	02-08	–	
G7227A	G2377E	*NS4B*	0.06	01-07, 09-15	–	^b^
A7736G	I2513M	*NS4B*	0.12	05-15	–	^a^
A8300G	V2735I	*NS5*	0.02	10, 12, 14	–	
A8622G	K2842R	*NS5*	0.05	02-08	–	^a^
G8946A	R2950K	*NS5*	0.02	06-09, 15	–	^a^
A9319T	R3074S	*NS5*	0.04	02-07	–	^a^

a Positive selection detected in the US dataset.

b Positive selection detected in NYS dataset only.

**Figure 3.**
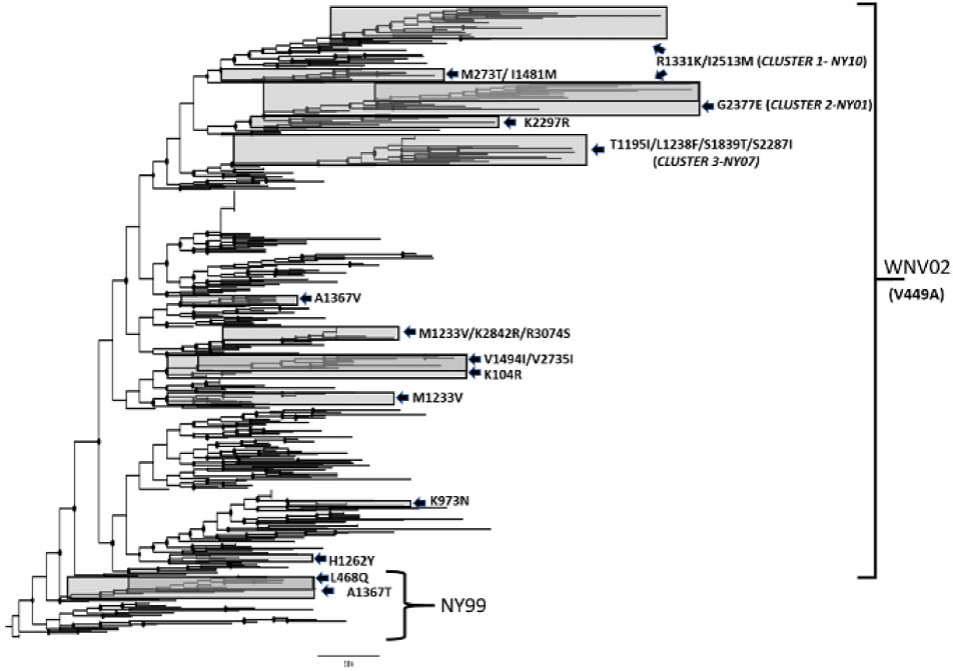
Maximum likelihood phylogeny based on amino acid sequences of NYS WNV isolates. Clades with shared amino acids which are supported (>0.75 posterior support) are shaded and individual amino acid substitutions are indicated. The most recent NYS isolates belong to one of the three clusters, designated NY10, NY01, and NY07 based on the initial year of isolation. Posterior support for these clusters is 0.92, 0.99, 1.00, and 0.96, respectively.

When the phylogeny of NYS isolates is inferred with amino acid sequences there are 16 clusters defined by 23 shared amino acid differences (Fig. 3). Recent NYS isolates fall into one of three clusters containing shared amino acid substitutions showing evidence for positive selection. These clusters are supported by posterior support ≥0.92. Included in this is Cluster 1, designated NY10, characterized by the shared amino acid mutations R1331K and I2513M; Cluster 2, designated NY01, characterized by the shared amino acid mutation G2377E; and Cluster 3, designated NY07, characterized by four shared amino mutations, T1195I, L1238F, S1839T, and S2287I.

### 2.5 Temporal structure

**Figure 4.**
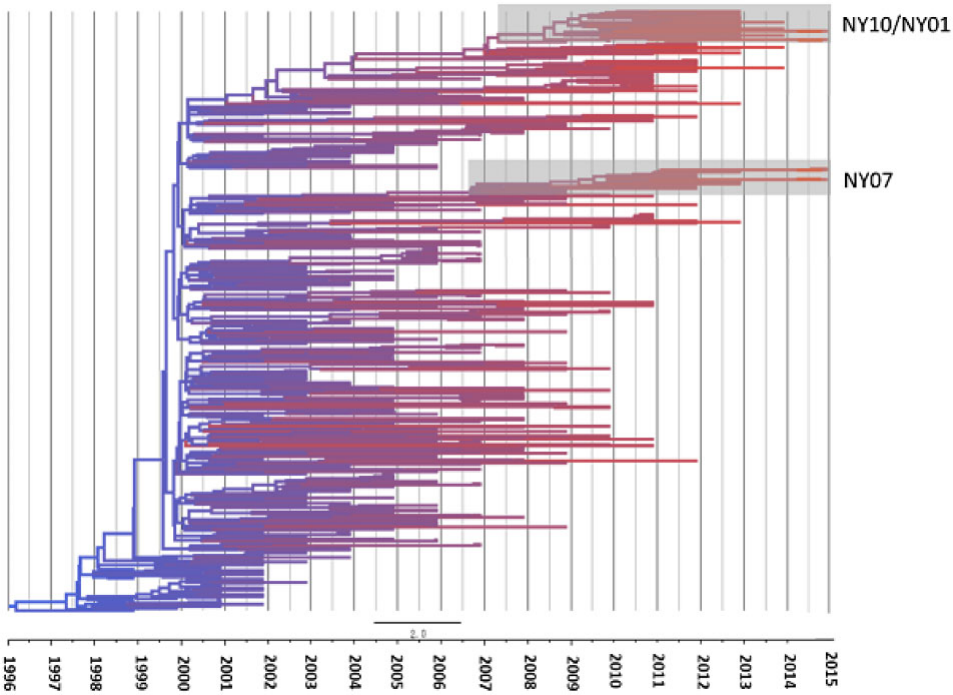
Time tree based on Bayesian analysis of WNV isolates from NYS. Branch color reflects the age of the sequence, red branches representing the most recent strains. Clusters containing genotypes NY07 and NY10/NY01 which are highlighted have posterior probabilities of 0.98 and 1.0, respectively.

**Figure 5.**
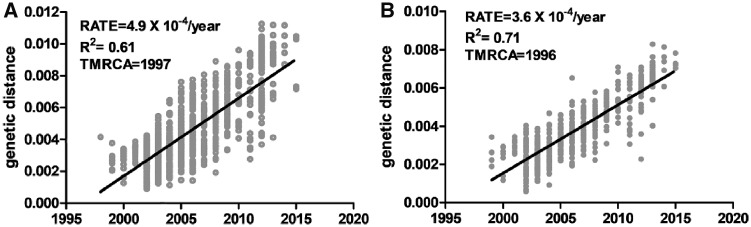
Temporal structure of WNV strains in the (A) US dataset and (B) the NYS dataset. Genetic distance refers to proportion of nucleotide differences relative to inferred root. Each WNV isolate is designated by an individual point and shown relative to the best fit line resulting from linear regression analyses. Rate refers to evolutionary rate, i.e. mutations/base/year. TMRCA refers to time to most recent ancestor. Analyses were completed in the program TempEst (64).

**Figure 6.**
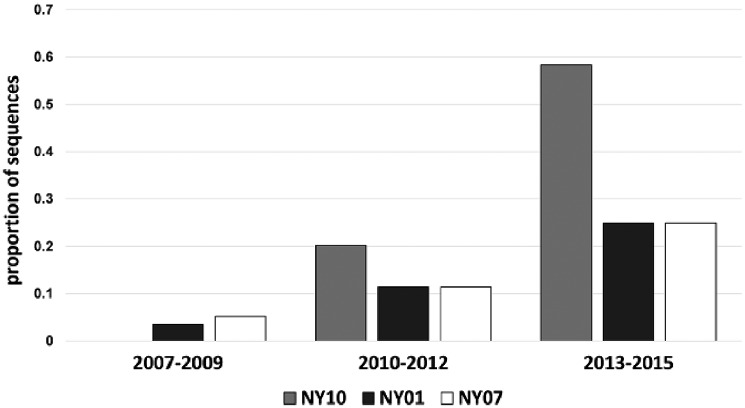
WNV genotype displacement in NYS, 2007–15. Genotypes are defined by shared amino acid differences. WNV NY10 contains R1331K and I2513M. NY01 contains G2377E. NY07 contains T1195I, L1238F, S1839T, and S2287I. G2377E also occurred in 12 of 37 NY10 strains and 1 of 3 2015 NY07 strains.

Overall, analyses demonstrate temporal clustering but also significant overlap among years (Figs 4 and 5). The evolutionary rate of WNV in the USA is 4.9×10^−4^ substitutions/site/year, when compared with the 3.6×10^−4^ substitutions/site/year for the NYS dataset. *R*^2^ values of the association between genetic and temporal distance are relatively low, 0.61 and 0.71, respectively for the USA and NYS datasets, which is consistent with high levels of temporal mixing. The most recent common ancestors were estimated at 1997 and 1996, for the USA and NYS datasets, respectively (Fig. 5). Despite temporal overlap there has been some genotype displacement in recent years (Figs 3–6). Notably, genotypes NY01, NY10, and NY07, named for the first year of identification and characterized by shared amino acid differences described in the previous section, have increased in prevalence over the last decade. All NYS strains from 2013 to 2015 belong to one of these genotypes (Fig. 6). In fact, some recent NY07 and NY10 strains contain the NY01 mutation, G2377E, suggesting this mutation has been selected independently multiple times. Both NY07 and NY10/NY01 clusters are highly supported by the inferred temporal phylogeny (posterior support ≥0.98; Fig. 4).

### 2.6 Geographic structure

Consistent with a panmictic US population, WNV strains from NYS and other regions were interspersed throughout the phylogenetic tree (Fig. 2). This is likely due both to the fact that all US strains were originally derived from NYS ancestors and that there are inherent sampling biases in the available US dataset. This sampling bias may not allow for accurate resolution of geographic structure on a national scale. Despite this, when NYS strains are considered independently some geographic structure emerges. We used Bayesian analysis to determine if there was any phylogenetic clustering by assigning common traits (location) to corresponding sequences in the NYS dataset. Specifically, upstate and downstate NYS strains are more phylogenetically separated than would be expected by chance via Bayesian Tip-association Significance Testing (BaTS) (AI <0.001, PS <0.001; Supplementary Fig. S1) (Parker, Rambaut, and Pybus 2008). In addition, among currently circulating NYS genotypes, NY10 (Cluster 1, R1331K/I2513M) and NY01 (Cluster 2, G2377E) occur more frequently upstate (*χ*^2^, *P* < 0.05). NY07 (Cluster 3, T1195I/L1238F/S1839T/S2287I) occurs more frequently downstate, yet this bias is not statistically significant (*χ*^2^, *P* = 0.14). Additional historic amino acid substitutions were also found to have a geographic bias. This included V121A, M273T, and E1685D, all of which were identified more frequently in downstate strains (*χ*^2^, *P* < 0.05).

### 2.7 Intrahost diversity

**Figure 7.**
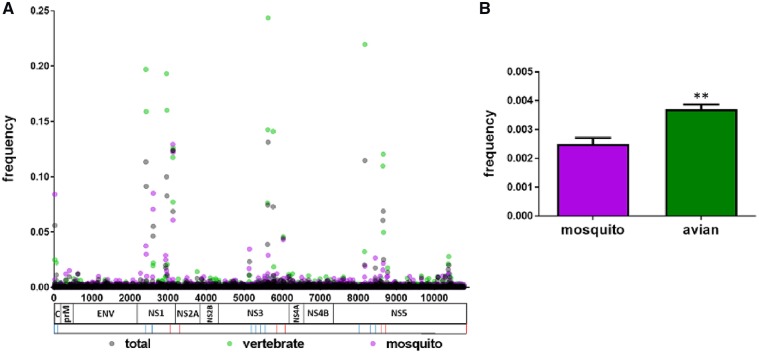
Intrahost diversity of WNV isolates from NYS, 2000 to 2015. Data are shown as proportion of non-consensus reads (A) at each position across the WNV genome and (B) overall, for the 317 isolates for which deep-sequencing data was available. Significance (*t*-test, *P* < 0.001) is denoted with **. Sequencing primers, forward and reverse, correspond to those in Supplementary Table S1 and are indicated by blue (forward) or red (reverse) lines below genome.

**Figure 8.**
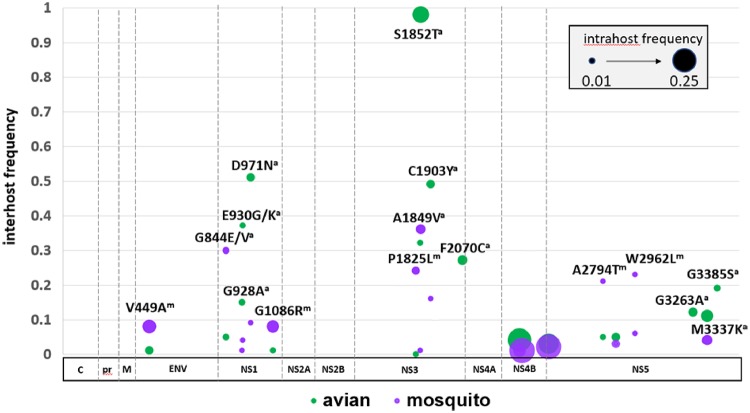
Shared minority amino acid substitutions identified in >2.0 per cent of WNV isolates from NYS. Interhost frequency (proportion of avian [green] and mosquito [purple] isolates possessing individual substitutions) is depicted by the *Y*-axis. A host bias (higher proportion of individual change in avian or mosquito isolates, *χ*^2^, *P* < 0.05) is indicated by ^a^ (avian) or ^m^ (mosquito). Intrahost frequency (proportion of mutant swarm possessing each substitution) is indicated by circle radius.

Minority sequence data across the WNV genome was analyzed for a total of 317 NYS isolates, including 145 avian and 163 mosquito samples. All isolates contained between 9.5 and 10.5 log_10_ copies/ml, as quantified by qPCR. There was no correlation between intrahost diversity and viral titer (Pearson’s *r* =−0.026, *r*^2^ = 0.0006, *P* = 0.65) or sequencing depth (Pearson’s *r* = −0.012, *r*^2^=0.0001, *P* = 0.62). The mean number of alternate alleles was 3.4×10^−3^/site, with an average of 31.8 non-consensus single nucleotide variants (SNVs)/isolate identified at levels >1.0 per cent. Diversity varied across the genome, with hot-spots identified in the *NS1*, *NS3*, and *NS5* genes (Fig. 7). We defined hot-spots as clusters of minority SNVs at a frequency above 0.01. In the *NS1* gene, we identified hot-spots at nucleotide positions 2412, 2421, 2605, 2610, 2955, 2964, 2931, 2934, 2974, 3126, and 3129. In the *NS3* gene there were hot-spots at positions 5128, 5136, 5610, 5617, 5625, 5757, 5771, and 6021. The *NS5* gene had hot-spots at positions 8161, 8172, 8440, 8643, 8655, 8661, and 8769. We compared both levels of diversity and individual minority SNVs identified here with previously published data and found no agreement, suggesting cell culture isolation did not contribute significantly to the intrahost diversity or mutant swarm composition identified in this study (Ciota et al. 2007a,b,c; Van Slyke et al. 2015). Although there are temporal fluctuations in mutant swarm size, we found no correlation between intrahost diversity and MLE or reported human cases (Pearson’s correlation, *P* > 0.05). Significantly more minority SNVs were identified in avian isolates relative to mosquito isolates (*t*-test, *P* < 0.001; Fig. 7), with mean SNV per isolate of 38.2 (4.4 amino acid substitutions per isolate) in avian samples, and mean SNV per isolate of 26.1 (2.2 amino acid substitutions per isolate) in mosquito samples. Levels of intrahost diversity were similar among isolates from different avian species. We also compared these measures between *Culex* and non-*Culex* isolates. *Culex* mosquito isolates (*n* = 103) showed a mean of 19.4 SNVs per isolate (2.0 amino acid substitutions per isolate) and non-*Culex* mosquitoes (*n* = 60) had significantly higher intrahost diversity, with mean SNV per isolate of 37.8 (2.5 amino acid substitutions per isolate; *t*-test, *P* < 0.001). Proportions of non-synonymous minority mutations were similar among vertebrates and invertebrates (∼0.10). Although most minority variants were synonymous and most non-synonymous variants were not shared among isolates, a total of twenty-one shared amino acid substitutions were identified in >2.0 per cent of isolates (seven or more isolates, Fig. 8). The majority of these (seventeen/twenty-one) were not found in consensus sequences of other isolates. Interestingly, the signature change of the WNV02 genotype, C1409T, was identified in the mutant swarm of thirteen mosquito isolates acquired prior to 2003, including a *Cx. pipiens* isolate (KX547395) from Suffolk county in 2000, prior to the identification of this mutation in consensus sequences. Three additional shared non-synonymous consensus mutations in the *NS4B* gene were found in mutant swarms, including two amino acid substitutions, G2377E (intrahost frequencies =10% vertebrates, 28% mosquitoes) and I2513M (intrahost frequencies = 18% vertebrates, 23% mosquitoes) with evidence of positive selection. All but one of the minority mutations not identified in any consensus sequence were found to have a bias for mosquito or avian hosts (*χ*^2^, *P* < 0.05; Fig. 8). These host-specific minority mutations on average occurred more frequently and at higher intrahost variant frequencies in avian isolates. Specifically, individual avian-biased minority amino acid substitutions, which are disproportionally located in the *NS1*, *NS3*, and *NS5* genes, were identified in an average of 35.1 per cent of avian-derived mutant swarms, with all avian isolates possessing at least three of these ten mutations. Included in this is the mutation T5617A (intrahost frequencies = 14% vertebrates, 5% mosquitoes), resulting in a S1852T amino acid substitution in the *NS3* gene, which was identified in 98 per cent of avian isolates. Individual mosquito-biased amino acid substitutions occurred in an average 17.1 per cent of isolates, with the most variable site, position 2594 (*NS1*, AA 844, intrahost frequencies = 3% vertebrates, 3% mosquitoes) demonstrating polymorphism in 30% of mosquito isolates. Despite a high frequency of isolates possessing these shared non-synonymous mutations, intrahost frequencies on average were low, ranging from 0.01 to 0.14 per cent of reads (Fig. 8).

## 3. Discussion

Large-scale phylogenetic studies are required to understand the extent and pace of pathogen evolution, the relative role of selective and stochastic forces in driving genetic change and the potential for phenotypic variability contributing to epidemiological fluctuations. The evolutionary potential of RNA viruses is vast, yet the pace of change is largely dependent on the consistency and strength of evolutionary pressures. Adaptive trade-offs result from a need to traverse highly diverse environments both within and between hosts, which increases the strength of purifying selection (Coffey et al. 2013). In addition, both to overcome the detrimental effects of mutational load in diverse systems and the potential for fitness costs resulting from frequent bottlenecks (Weaver et al. 1999), mutational robustness is likely to evolve (Wilke and Novella 2003). Together, these evolutionary pressures significantly constrain the pace of adaptive evolution of WNV and other arboviruses. Furthermore, adaptive evolution was not required for WNV to succeed in the USA since large populations of competent vectors and naïve hosts were already established. Consistent with this, WNV has generally experienced unconstrained movement in the USA and few phenotypically relevant mutations and/or positions under selection have been identified on a national scale since the 1999 introduction of WNV to NYS (Di Giallonardo et al. 2016).

While our data are generally in agreement with the pace of evolutionary change identified in other studies, as well as a large-scale geographic and temporal mixing, we have identified some important exceptions to this both in NYS and the USA as a whole. Most notably, we have identified fourteen amino acid changes showing evidence for positive selection in the USA and/or NYS, including nine novel positions. While sites with elevated d*N*/d*S* ratios could be under fluctuating selective pressures, individual mutations associated with emergent genotypes, which are increasing in frequency, are likely under directional positive selection (Hughes and Friedman 2008). None of these positively selected sites are in the structural genes, which is perhaps unsurprising as substantial alterations to host usage have not changed since the introduction and therefore targets for binding and entry have likely been static (Lin et al. 2006). Adaptive evolution seems to instead be occurring in non-structural genes that have the capacity to fine-tune immune evasion and replicase function (Munoz-Jordan et al. 2003; Liu et al. 2004; Lin et al. 2006; Ashour et al. 2009; Mazzon et al. 2009). The previously identified residue showing evidence for positive selection associated with the SW03 genotype, *NS4A*-85, as well as positions *NS5*-314 and *NS2A*-224 (McMullen et al. 2011; Anez et al. 2013), were confirmed here. Previous studies suggested that this genotype first appeared in 2003 in New Mexico and Arizona, and not in NYS until 2005 (McMullen et al. 2011). Interestingly, our updated dataset now places SW03 in NYS in 2002, demonstrating a much different evolutionary and geographic history. While the phenotypic significance of the *NS4A*-85 position is not known, it is highly polymorphic with four unique amino acid residues identified. Phenotypic studies comparing SW03 to WN02 strains demonstrate both similar fitness and virulence in birds, and similar competence in *Cx. tarsalis* (Duggal et al. 2014; Worwa et al. 2018). Interestingly, the *NS4A*-A85T mutation has not been identified in NYS since 2012 and this and other genotypes have now largely been displaced in the region by NY01, NY07, and NY10. These displacement events have been coincident with an overall increase in WNV activity in NYS since 2010, suggesting that mutations associated with these genotypes could confer fitness gains that contribute to increases in prevalence in mosquitoes and vertebrate hosts. While environmental factors, even on fine geographic and temporal scales, likely influence the spread and abundance of WNV vectors and WNV transmission, these data are supportive of a role for adaptive evolution in driving increased prevalence.

With the exception of a single AA change in *NS3* (S1839T), the non-synonymous mutations associated with the emergent genotypes confer amino acid substitutions in the *NS2A* and *NS4*. These proteins are multifunctional and have been shown to be involved in RNA replication (Rossi et al. 2007), membrane permeabilization (Chang et al. 1999) virion formation (Kummerer and Rice 2002; Liu, Chen, and Khromykh 2003; Leung et al. 2008), host antiviral responses (Liu et al. 2005; Liu et al. 2006; Melian et al. 2013), and vector competence (McElroy et al. 2006). The geographic bias identified with these mutations suggests the possibility of regional adaptations, perhaps maximizing transmission by distinct mosquito populations. A previous study in our laboratory demonstrated clustering of WNV genotypes on geographic scales of finer granularity (Ehrbar et al. 2017). Although an alternative explanation for this is simply stochastic drift, the unfettered movement of WNV strains that has been demonstrated here suggests such clustering is unlikely over multiple years without an accompanying adaptive advantage. While our ability to identify several positions for which there was significant evidence for positive selection was aided by the larger dataset and the longer evolutionary history studied here, clearly smaller geographic and temporal scales can also reveal important regional evolutionary pressures. For example, changes at amino acids 2209 and 2377 were identified as under positive selection in NYS but not when the entire US dataset is considered, again suggesting selective pressures can be regionally unique. Previous studies have demonstrated population and strain-specific differences in competence of mosquitoes for WNV and other arboviruses (Vaidyanathan and Scott 2007; Kilpatrick et al. 2010; Sudeep et al. 2015). Future studies with relevant field populations and diverse strains will help to clarify the extent to which these substitutions represent adaptations to local populations of mosquitoes.

Identifying intrahost minority variants can be useful to survey for phenotypically relevant consensus mutations prior to displacement. The WNV02 mutation, C1409T, for instance, was identified in the mutant swarm prior to its selective sweep. Other consensus mutations were also identified as minority variants in strains lacking these mutations in consensus sequences. While this suggests deep-sequencing could be a useful tool to predict the emergence of new genotypes, the majority of non-synonymous minority mutations shared among isolates had a significant bias for vertebrate or invertebrate hosts and therefore are not likely to ever be fixed in the consensus. These mutations, which are often shared over time and space, particularly among birds, are either selected against but retained at low levels in alternate hosts via molecular memory (Ruiz-Jarabo et al. 2000; Arias et al. 2004), or repeatedly selected during individual infections. Regardless, this provides evidence that host-specific fitness could to some extent be optimized by shifting mutant swarm populations and provides targets for understanding host-specific mechanisms of viral fitness.

As has been found in previous studies, high levels of intrahost diversity exist in the *NS1* gene (Ehrbar et al. 2017) suggesting density-dependent selection, rather than purifying selection could dominate certain regions of this gene. Despite this, the majority of shared non-synonymous minority mutations are located in the replication complex, including in the helicase portion of the *NS3* and both the methyltransferase and RNA-dependent RNA polymerase portions of the *NS5*. Mutations with host bias in these regions are not necessarily surprising, as previous studies demonstrate that the phenotypic consequences of altered polymerase function and fidelity is host-dependent (Van Slyke et al. 2015).

The fact that numbers of shared minority mutations and overall intrahost diversity is higher from avian isolates stands in contrast to a number of experimental studies demonstrating stronger purifying selection and lower intrahost diversity in birds relative to mosquitoes (Jerzak et al. 2008; Deardorff et al. 2011; Ciota et al. 2013; Grubaugh et al. 2016), yet this has not reliably been the case with natural isolates (Jerzak et al. 2005; Nelson et al. 2018). These discrepancies could partially be attributable not only to methodology (depth/region of sequencing, method of infection and/or amplification, timepoints assayed, viral load, process errors), but also to species and tissue types studied (Ciota et al. 2008, 2012; Jerzak et al. 2008; Brackney et al. 2011). Although additional passaging was not done, and highly permissive host-specific cell lines were used for these studies, an important consideration for interpretation is that all WNV strains deep-sequenced were isolated on cell culture. Previous studies demonstrate that a single round of amplification is not likely to generate the levels of intrahost diversity identified here, and that the expected signature of intrahost diversity which is an artifact of culture would be higher levels of variability in mosquito cell-derived isolates (Ciota et al. 2007a,b,c, 2011; Van Slyke et al. 2015). The fact that we observed the opposite (i.e. higher intrahost diversity in avian derived samples isolated on vertebrate cells) and the fact that none of the variants we identified have been previously identified with experimental strains passaged in cell culture, together suggest that these minor variants were likely naturally occurring. Despite this, it is certainly feasible that the proportions of individual intrahost variants was influenced by isolation. To achieve sufficient sequencing depth for these analyses without amplification of samples with relatively low viral loads would be difficult, yet further studies are required to fully validate the effect of this methodology on mutant swarm composition. Additionally, not knowing the ancestral sequence of these natural isolates, in contrast to experimental infections, could complicate interpreting diversity. While experimental studies have generally appropriately utilized virus derived from blood at peak viremia, the majority of our isolates are derived from highly infected brain tissue. Given that crossing the blood–brain barrier likely imposes a tight bottleneck, it is somewhat surprising that there is more diversity in isolates from brain tissue, but this could simply be a result of higher levels of replication over relatively long periods of time following colonization of the CNS. While these viral populations are not directly evolutionarily important, as invasion of the CNS is a dead-end for WNV, neuroinvasiveness and neurovirulence to some extent are simply artifacts of maximizing fitness in the vertebrate host, so shared non-synonymous mutations could be generic markers of WNV fitness in vertebrates.

Overall, these analyses demonstrate that adaptive evolution of WNV, though limited in the USA, is likely more extensive than once thought. In addition, genetic variation may be constrained by host cycling but remains substantial, both at the inter- and intrahost levels, as well at relatively fine geographic scales. The extent to which this genetic variability is epidemiologically relevant is unclear, yet the temporal and regional fluctuation in WNV prevalence and the evidence for both regionally specific evolution and ongoing strain displacement warrant future studies utilizing appropriate field populations and continued genetic and phenotypic evaluation of WNV.

## 4. Materials and methods

### 4.1 WNV surveillance and testing

MLE of mosquito infection were calculated using data from the NYS Arbovirus Laboratory (Wadsworth Center, NYSDOH) surveillance program. WNV-positive mosquito pools and vertebrate samples were identified using real-time qRT-PCR assay as previously described (Zink et al. 2013). *Culex pipiens* and *Culex restuans* are pooled prior to testing because they are morphologically indistinguishable. WNV prevalence (MLE) was calculated based on mosquito surveillance pool sizes using an Excel Add-In (https://www.cdc.gov/westnile/resourcepages/mosqSurvSoft.html). Human case incidence was obtained from the NYS Bureau of Communicable Disease Control (https://www.health.ny.gov/professionals/diseases/reporting/communicable/).

### 4.2 Primer design

WNV RT and PCR primers were generated using a combination of manual primer design (utilizing CLC Genomics workbench software, QIAGEN), previous publications, or an internal automated primer design pipeline (Papin et al. 2010; Youn et al. 2012). Primer design was based on a consensus multiple sequence alignment of published WNV Lineage 1 complete genomes (accessed from GenBank in September 2015). Several overlapping amplicons with an average length of approximately 3 kb were generated (Supplementary Table S2).

### 4.3 Sample preparation

Homogenized samples previously identified as WNV-positive using real-time RT-PCR were amplified once on C6/36 (*Aedes albopictus*) or Vero (African green monkey kidney) cell culture (for mosquito and vertebrate isolates, respectively) and RNA was extracted using MagMax nucleic acid isolation chemistry (Applied Biosystems). Extracted RNA was used to synthesize cDNA using the SuperScript III Reverse Transcriptase kit (ThermoFisher Scientific), followed by RNase H digestion. Four reverse primers (also used as RT primers) were diluted to 1 μM and pooled in equal volumes. cDNA was generated from 4 μl undiluted RNA, using the pooled reverse primers and SuperScript III Reverse Transcriptase for 50 min at 50 °C followed by inactivation for 15 min at 70 °C. Targeted PCR reactions were performed on 2 μl of cDNA template using 10 μM of each primer and Phusion High Fidelity DNA Polymerase (New England Biolabs) to generate overlapping approximately 3 kb amplicons across the genome. Negative controls were used to detect any contamination, and no indications of contamination were observed. Forward and reverse primers were designed to generate eight amplicons including: AGTAGTTCGCCTGTGTGARCTG and ACATTCAGTYGTGTTGCTYTCTC, TGTGAGGATTAAYAACAATTAACAC and CCATCGCCCCAYAARGTRTGC, GCTYTTCCTCTCMGTGAACG and ATTTTGGGTACTCMGTCTCR, AGTGTGCGGTYTACGRTCAG and ARTCGTCYTCATTCGTGTGC, RATGGCTGAAGCMYTGAGAG and TTCCATTCTTCCYARRAGCACC, AGGYACYTCAGATCCATTCC and GCACRTACTTCACTCCTTCTG, YTGGTGYTACTAYATGGCAACC and ARCASARGATCTCCTAGTCTATCC, AAAYCCRCTCTCACGRAAYTCC and ATCCTGTGTTCTCGCACCACCAG. Additional redundant primers included: TGYTGAGGAARAAACAGATCACT and CCKSGTTCCACTTCCCAAG, YTGGAACWCTGGATAYGAATG and GTGATRGTGTCCCATGGYTT, TGCACCGAGGRCCAAGGG and ARCASARGATCTCCTAGTCTATCC (Supplementary Table S2). Polymerase chain reaction involved a 30 s initial denaturation step at 98°C followed by 35 cycles of 98°C for 5 s, 55°C for 20 s, and 72°C for 150 s; and a final extension at 72°C for 5 min. The resulting amplicons were first visualized and quantified using the QIAxcel Advanced System (QIAGEN) prior to pooling in equal concentrations for library construction.

### 4.4 Genome sequencing

Samples 1 through 186 were sequenced using an Ion Torrent PGM instrument (ThermoFisher Scientific). In this case, pooled DNA amplicons were sheared, and Ion Torrent-compatible barcoded adaptors were ligated to the sheared DNA using Ion Xpress Plus Fragment Library Kit (ThermoFisher Scientific) to generate 400-bp libraries.

Samples 187 through 495 were sequenced using Illumina MiSeq instrument (2×300 bp PE, Illumina). All deep-sequencing analyses were carried out using Illumina MiSeq data. In this case, DNA amplicons were pooled and subjected to adaptor ligation and library construction (as detailed in manufacturer’s instructions, Illumina).

### 4.5 Genome assembly and annotation

After sequencing, reads from each sample were deconvoluted by barcode and trimmed to eliminate low-quality regions, barcode sequences, and PCR primers. Trimmed reads were subjected to *de novo* assembly with the CLC Bio software suite (clc_novo_assemble). The resulting contigs and were then queried against a custom full-length WNV reference database to determine the closest reference sequence. Contigs were then mapped to the selected reference sequence for each sample using the CLC Bio software suite (clc_ref_assemble_long). For sites where the majority of reads disagreed with the sequence from the reference strain, the reference sequence was updated accordingly. A final mapping of all next-generation reads to the selected reference sequences was performed using the CLC Bio software (clc_ref_assemble_long;.Code available upon request). Curated assemblies were validated and annotated with the Viral Genome ORF Reader (VIGOR) software (Wang, Sundaram, and Stockwell 2012). VIGOR was also used to validate assemblies and detect potential frameshifts. The annotation was subjected to manual inspection and quality control before submission to GenBank.

All sequences generated in this study were submitted to GenBank (accession numbers KX547164 through KX547621) as part of Bioproject PRJNA262930.

For this collection, the average number of reads used to generate the consensus whole genome sequence for each sample was 12,701. In order to identify statistically significant minor variants, deep-sequencing analysis was also performed using an average of 262,662 reads in the full assembly for each sample. The mean coverage for the collection was 4,722×.

### 4.6 Sequence data

ORF sequences were obtained from GenBank, excluding any laboratory or vaccine strains. Additionally, any sequences lacking information on the host, year, or location of collection were excluded from these analyses. The dataset was manually inspected for incomplete sequences, which were subsequently removed, and then aligned using MAFFT (Katoh and Standley 2014). In all, the Global, the USA, and NYS data sets consisted of 1872, 1239, and 543 sequences, respectively. The global dataset used all full-genome WNV sequences available through GenBank as of September 2017, and the USA dataset used all available full-genome sequences available through GenBank as of the same date. The NYS dataset used taxa with the GenBank accession numbers AF196835, AF202541, AF206518, AF260967, AF404755, AF404756, AF533540, DQ164186 through DQ164195, FJ151394, HM488199 through HM488207, HM488237 through HM488252, HM756660 through HM756673, HM756675, HM756678, HQ596519, HQ596519, HQ671721 through HQ671730, HQ705660, JF488094 through JF488097, JF730042, JF899528, JF889529, JN183885 through JN183888, JN367277, JQ700437, JQ700442, KJ145827 through KJ145829, KJ786934, KX547164 through KX547176, KX547178 through KX547621. An additional NYS intrahost dataset was generated for analysis of minority variants, which consisted of 317 sequences. After alignment, the 5′ and 3′ untranslated regions were removed from the genomes resulting in the ORF datasets that were used in all subsequent analyses. All significance testing for clustering of mutations of interest was performed in GraphPad Prism 5.

Sequences were inspected for evidence of recombination using RDP 4.95 (Martin and Rybicki 2000) and no significant evidence of recombination events was found. An ML phylogeny was estimated from the sequence data using FastTree in Sate 2.2.7 using the GTR G20 model and SATe-II-fast with the max subproblem set to 200 bp (Liu and Warnow 2014). The decomposition strategy was set to centroid and the program was set to return the best tree with blind mode enabled.

Analysis of clustering by upstate and downstate was accomplished through use of BaTS, using a Bayesian tree generated in BEAST.

### 4.7 Evolutionary rate

Evolutionary rates were estimated using the Bayesian Markov chain Monte Carlo method implemented in the program BEAST2 (Bouckaert et al. 2014). The GTR + I substitution model was found to be the best-fit for this dataset using bModelTest (Bouckaert and Drummond 2017) and all subsequent Bayesian analyses were run using this substitution model, an uncorrelated log normal relaxed clock, and a logistic population growth model. All analyses were run for at least 200 million generations to allow all models to converge and for all parameters to reach a sufficient estimated sampling size (>200), discarding the first 10 per cent of these generations as burn-in, sampling every 20,000 steps. Maximum clade credibility trees, using statistical support for nodes given as posterior probability values, were estimated using TreeAnnotator in BEAST 2.

The ML tree was used to perform root-to tip regression analyses to obtain evolutionary rates in TempEst (Rambaut et al. 2016).

### 4.8 Analysis of selection

Selective pressure (positive or purifying) was estimated for codons across the WNV genome by calculating variability in the ratio of non-synonymous mutations (d*N*) to synonymous (d*S*) mutations per codon site using the HyPhy package (Pond, Frost, and Muse 2005). Three tests of significance were used, including the fixed-effects likelihood, single-likelihood ancestor counting, and fast, unbiased Bayesian approximation models (Pond, Frost, and Muse 2005). The d*N*/d*S* values for each gene in the WNV genome, as well as the entire ORF were also estimated using HyPhy.

### 4.9 Minor variant detection

Three hundred and seventeen of the 495 genomes that were sequenced and assembled demonstrated sufficient quality and depth of coverage (>1,000×) to enable the identification of the minor variants circulating in the population. This involved generating a consensus sequence from all sequence reads for each sample using CLC mapping assembly (clc_ref_assemble_long) as discussed above. A custom script was then used to map the complete set of high-quality trimmed sequence reads from each viral strain to the associated consensus sequence. Separate scripts were then used to calculate the major and minor alleles, determine the nucleotide location of the observed allele, and predict whether the change in each codon would result in an amino acid substitution. These annotations were then used to provide biological context and meaning to all detected minor variants. To find statistically significant variations in the population, all forward and reverse reads covering each position were checked and a statistical model using a binomial distribution was generated to ensure that coverage at each base position was above a specific threshold (0.1%) with a 95% confidence interval followed by multiple-hypothesis correction using the Bonferroni method. Positions lacking sufficient coverage to call a minor allele with 95 per cent confidence were not reported in the output. The variations observed in the sequencing reads for each strain were reported against the consensus sequence of the same strain to obtain the percentage of minor alleles in the population.

## Supplementary Material

vez020_Supplementary_Data
