# Supplementary material for: Evolutionary dynamics and molecular epidemiology of West Nile virus in New York State: 1999–2015
Source: Virus Evol. 2019 Jul 21;5(2):vez020. doi: 10.1093/ve/vez020 (PMC6642743; doi:10.1093/ve/vez020)
Supplement: vez020_Supplementary_Data [file vez020_supplementary_data.zip › Supplementary Legends.docx]

**Supplementary Figure 1**. Depiction of BEAST phylogeny generated by classifying

upstate (blue) or downstate (orange) derived WNV isolates from NYS.

**Supplementary Table 1:** Results of West Nile virus selection analyses for U.S. and NYS datasets. Data are shown as dN/dS (SLAC p-value, FEL p-value, FUBAR p-value). Positions with statistical significance by at least two tests are in bold text.

**Supplementary Table 2.** Sequencing primers used for amplicon generation and library preparation for deep sequencing.

**Supplementary Table 3.** dN/dS values by gene and host for West Nile virus in NYS.
