# Supplementary material for: Evolutionary dynamics and molecular epidemiology of West Nile virus in New York State: 1999–2015
Source: Virus Evol. 2019 Jul 21;5(2):vez020. doi: 10.1093/ve/vez020 (PMC6642743; doi:10.1093/ve/vez020)
Supplement: vez020_Supplementary_Data [file vez020_supplementary_data.zip › Supplementary Table 1.docx]

| dN/dS (SLAC p-value (α=0.05), FEL p-value (α=0.05), FUBAR p-value (α=0.10)) | | |
| --- | --- | --- |
| **Codon** | **US** | **NYS** |
| 1 | 0.2226 (< 0.0001, 0.0231, 0.9357) | 0.3352 (0.0036, 0.9944, 0.5440) |
| 2 | 1.5136 (0.8637, 0.2192, 0.4220) | 0.9963 (0.1991, 0.4478, 0.0902) |
| 3 | 0.3166 (< 0.0001, 0.0779, 0.7199) | 0.4981 (0.6872, 0.6831, 0.3627) |
| 4 | < 0.0001 (0.8741, 0.0060, 1.0000) | < 0.0001 (1.0000, 1.0000, 0.9846) |
| 5 | 1.0000 (0.6252, 1.0000, 0.9552) | < 0.0001 (< 0.0001, 1.0000, 0.5703) |
| 6 | 1.0000 (1.0000, 1.0000, 0.9216) | < 0.0001 (< 0.0001, 1.0000, 0.5103) |
| 7 | 0.6212 (0.6125, 0.2325, 0.7037) | 0.5002 (0.6668, 0.6664, 0.3562) |
| 8 | 0.3613 (0.0002, 0.1104, 0.6803) | < 0.0001 (< 0.0001, 1.0000, 0.6213) |
| 9 | 0.6214 (0.7067, 0.2327, 0.2500) | 0.4994 (0.6671, 0.6674, 0.3385) |
| 10 | 0.6341 (0.4124, 0.2430, 0.8480) | 0.9213 (0.7681, 0.7644, 0.5007) |
| 11 | 1.0000 (1.0000, 1.0000, 0.8693) | < 0.0001 (< 0.0001, 1.0000, 0.5591) |
| 12 | 1.0000 (0.8711, 1.0000, 0.9436) | < 0.0001 (< 0.0001, 1.0000, 0.5425) |
| 13 | < 0.0001 (0.2949, 0.0061, 0.9807) | < 0.0001 (1.0000, 1.0000, 0.7976) |
| 14 | < 0.0001 (0.1958, 0.0083, 0.9841) | < 0.0001 (1.0000, 1.0000, 0.8089) |
| 15 | 1.0000 (0.4677, 1.0000, 0.8264) | < 0.0001 (< 0.0001, 1.0000, 0.5113) |
| 16 | 1.0000 (0.5895, 1.0000, 0.9870) | < 0.0001 (< 0.0001, 1.0000, 0.7226) |
| 17 | 1.3505 (< 0.0001, 0.3430, 0.2148) | 0.8804 (0.3763, 0.3805, 0.1962) |
| 18 | 1.0000 (1.0000, 1.0000, 0.9379) | < 0.0001 (< 0.0001, 1.0000, 0.5342) |
| 19 | 1.0000 (< 0.0001, 1.0000, 0.8772) | < 0.0001 (< 0.0001, 1.0000, 0.5510) |
| 20 | < 0.0001 (< 0.0001, 0.0060, 0.9854) | < 0.0001 (1.0000, 1.0000, 0.7428) |
| 21 | < 0.0001 (0.6738, 0.0014, 0.9870) | < 0.0001 (< 0.0001, 1.0000, 0.7226) |
| 22 | 1.0000 (0.0002, 1.0000, 0.8998) | < 0.0001 (< 0.0001, 1.0000, 0.6213) |
| 23 | 1.0000 (< 0.0001, 1.0000, 0.8772) | < 0.0001 (< 0.0001, 1.0000, 0.5510) |
| 24 | 1.0000 (1.0000, 1.0000, 0.9525) | < 0.0001 (< 0.0001, 1.0000, 0.5996) |
| 25 | 1.0000 (0.9636, 1.0000, 0.8401) | < 0.0001 (< 0.0001, 1.0000, 0.4412) |
| 26 | 1.0000 (0.0613, 1.0000, 0.8984) | < 0.0001 (< 0.0001, 1.0000, 0.6391) |
| 27 | < 0.0001 (< 0.0001, 0.0397, 0.9739) | < 0.0001 (1.0000, 1.0000, 0.6732) |
| 28 | 1.0000 (0.7130, 1.0000, 0.8516) | < 0.0001 (< 0.0001, 1.0000, 0.5051) |
| 29 | < 0.0001 (0.0002, 0.0060, 0.9854) | < 0.0001 (< 0.0001, 1.0000, 0.5103) |
| 30 | < 0.0001 (0.7351, 0.0425, 0.9871) | < 0.0001 (1.0000, 1.0000, 0.8162) |
| 31 | 1.0000 (0.8958, 1.0000, 0.9431) | < 0.0001 (< 0.0001, 1.0000, 0.5865) |
| 32 | 0.2862 (0.0284, 0.0575, 0.7188) | < 0.0001 (< 0.0001, 1.0000, 0.5533) |
| 33 | 1.0000 (< 0.0001, 1.0000, 0.8455) | < 0.0001 (< 0.0001, 1.0000, 0.5601) |
| 34 | 0.4455 (0.4360, 0.0934, 0.8751) | 0.3352 (0.0036, 0.9944, 0.5440) |
| 35 | < 0.0001 (0.3801, 0.0397, 0.9903) | < 0.0001 (1.0000, 1.0000, 0.7889) |
| **36** | **2.2371 (0.0074, 0.0224, 0.1636)** | 0.8218 (0.7568, 0.7950, 0.4183) |
| 37 | < 0.0001 (0.1845, 0.0424, 0.9986) | < 0.0001 (1.0000, 1.0000, 0.6987) |
| 38 | 0.3170 (0.9988, 0.0782, 1.0000) | 0.2491 (0.9991, 0.9632, 0.9984) |
| 39 | 0.3245 (0.7130, 0.0835, 0.5027) | < 0.0001 (< 0.0001, 1.0000, 0.5952) |
| 40 | < 0.0001 (0.9547, 0.0056, 0.9984) | < 0.0001 (1.0000, 1.0000, 0.9973) |
| 41 | 0.9508 (< 0.0001, 0.4649, 0.9171) | 0.4625 (0.9062, 0.9000, 0.7924) |
| 42 | < 0.0001 (< 0.0001, 0.0056, 0.9985) | 0.4999 (0.8889, 0.8889, 0.6348) |
| 43 | 1.0000 (0.9987, 1.0000, 0.9552) | < 0.0001 (< 0.0001, 1.0000, 0.7334) |
| 44 | 1.3238 (0.3235, 0.2560, 0.4222) | < 0.0001 (1.0000, 1.0000, 0.9810) |
| 45 | < 0.0001 (0.5084, 0.0313, 0.9862) | < 0.0001 (1.0000, 1.0000, 0.9256) |
| 46 | 1.1698 (< 0.0001, 0.3957, 0.9528) | < 0.0001 (< 0.0001, 1.0000, 0.6238) |
| 47 | < 0.0001 (< 0.0001, 0.0083, 1.0000) | < 0.0001 (< 0.0001, 1.0000, 0.7585) |
| 48 | 1.0000 (0.0008, 1.0000, 0.8401) | < 0.0001 (< 0.0001, 1.0000, 0.6993) |
| 49 | < 0.0001 (0.9060, 0.0061, 0.9991) | < 0.0001 (1.0000, 1.0000, 0.9848) |
| 50 | < 0.0001 (0.9877, 0.0126, 0.9972) | < 0.0001 (1.0000, 1.0000, 0.9086) |
| 51 | < 0.0001 (< 0.0001, 0.0397, 0.9960) | < 0.0001 (1.0000, 1.0000, 0.9607) |
| 52 | 0.9587 (0.0585, 0.4707, 0.7614) | < 0.0001 (< 0.0001, 1.0000, 0.9110) |
| 53 | < 0.0001 (< 0.0001, 0.0117, 0.9993) | 0.3337 (0.9990, 0.9988, 0.7334) |
| 54 | < 0.0001 (0.9028, 0.0117, 0.9998) | < 0.0001 (< 0.0001, 1.0000, 0.8483) |
| 55 | 1.0000 (0.8889, 1.0000, 0.9411) | < 0.0001 (1.0000, 1.0000, 1.0000) |
| 56 | 1.0000 (1.0000, 1.0000, 0.9061) | < 0.0001 (1.0000, 1.0000, 0.8317) |
| 57 | < 0.0001 (0.5798, 0.0050, 0.9997) | < 0.0001 (1.0000, 1.0000, 0.9797) |
| 58 | < 0.0001 (< 0.0001, 0.0061, 0.9999) | < 0.0001 (1.0000, 1.0000, 0.9797) |
| 59 | 0.6352 (< 0.0001, 0.2440, 0.6701) | < 0.0001 (< 0.0001, 1.0000, 0.6216) |
| 60 | 0.9581 (0.7066, 0.4703, 0.2005) | 0.1667 (0.9877, 0.9877, 0.8855) |
| 61 | < 0.0001 (< 0.0001, 0.0093, 0.9995) | < 0.0001 (1.0000, 1.0000, 0.9596) |
| 62 | < 0.0001 (1.0000, 0.0050, 0.9874) | 0.4206 (0.9735, 0.9123, 0.6496) |
| 63 | < 0.0001 (< 0.0001, 0.0313, 0.9862) | 1.4993 (0.5931, 0.5928, 0.3708) |
| 64 | < 0.0001 (< 0.0001, 0.0061, 1.0000) | < 0.0001 (1.0000, 1.0000, 0.9663) |
| 65 | 0.3480 (< 0.0001, 0.1006, 0.9439) | < 0.0001 (1.0000, 1.0000, 1.0000) |
| 66 | < 0.0001 (0.7130, 0.0425, 0.9670) | 0.4709 (0.9001, 0.8975, 0.6489) |
| 67 | 0.6494 (< 0.0001, 0.2555, 0.4809) | < 0.0001 (1.0000, 1.0000, 0.9749) |
| 68 | < 0.0001 (< 0.0001, 0.0313, 0.9701) | < 0.0001 (< 0.0001, 1.0000, 0.8341) |
| 69 | 1.0000 (< 0.0001, 1.0000, 0.9798) | < 0.0001 (1.0000, 1.0000, 0.9601) |
| 70 | < 0.0001 (< 0.0001, 0.0044, 0.9964) | 1.0000 (0.4444, 0.4444, 0.2132) |
| 71 | 1.0000 (< 0.0001, 1.0000, 0.7969) | < 0.0001 (1.0000, 1.0000, 0.8931) |
| 72 | < 0.0001 (< 0.0001, 0.0083, 0.9948) | < 0.0001 (1.0000, 1.0000, 0.9086) |
| 73 | < 0.0001 (0.7130, 0.0053, 0.9999) | < 0.0001 (< 0.0001, 1.0000, 0.6238) |
| 74 | 0.3166 (< 0.0001, 0.0779, 0.8902) | < 0.0001 (1.0000, 1.0000, 0.9784) |
| 75 | 0.6033 (0.6667, 0.2907, 0.6035) | < 0.0001 (1.0000, 1.0000, 0.9641) |
| 76 | 0.3012 (0.4823, 0.0674, 0.7953) | 0.2358 (0.9968, 0.9671, 0.9141) |
| 77 | 1.0000 (< 0.0001, 1.0000, 0.9560) | < 0.0001 (1.0000, 1.0000, 0.9827) |
| 78 | 1.0000 (0.6667, 1.0000, 0.9870) | < 0.0001 (< 0.0001, 1.0000, 0.7454) |
| 79 | 1.0000 (0.9095, 1.0000, 0.9379) | < 0.0001 (1.0000, 1.0000, 0.9592) |
| 80 | 1.0000 (0.4444, 1.0000, 0.8897) | < 0.0001 (< 0.0001, 1.0000, 0.9110) |
| 81 | < 0.0001 (0.9410, 0.0126, 0.8565) | < 0.0001 (< 0.0001, 1.0000, 0.5813) |
| 82 | < 0.0001 (0.0260, 0.0419, 1.0000) | < 0.0001 (< 0.0001, 1.0000, 0.8417) |
| 83 | 1.0000 (0.7130, 1.0000, 0.8305) | < 0.0001 (< 0.0001, 1.0000, 0.7242) |
| 84 | < 0.0001 (< 0.0001, 0.0117, 1.0000) | 0.2393 (0.8249, 0.9661, 0.4124) |
| 85 | 1.0000 (0.1976, 1.0000, 0.9431) | 0.3335 (0.9999, 0.9995, 0.7363) |
| 86 | < 0.0001 (0.2963, 0.0060, 0.9996) | < 0.0001 (1.0000, 1.0000, 0.9640) |
| 87 | 1.0000 (1.0000, 1.0000, 0.9351) | < 0.0001 (< 0.0001, 1.0000, 0.5813) |
| 88 | < 0.0001 (0.6667, 0.0422, 0.9995) | < 0.0001 (1.0000, 1.0000, 0.9776) |
| 89 | < 0.0001 (0.2280, 0.0056, 0.9960) | < 0.0001 (1.0000, 1.0000, 0.9937) |
| 90 | 0.3013 (0.1759, 0.0674, 0.9554) | < 0.0001 (1.0000, 1.0000, 0.9663) |
| 91 | < 0.0001 (0.1318, 0.0397, 0.8402) | < 0.0001 (1.0000, 1.0000, 0.9936) |
| 92 | 0.3013 (< 0.0001, 0.0674, 0.6318) | < 0.0001 (< 0.0001, 1.0000, 0.6139) |
| 93 | < 0.0001 (0.1975, 0.0037, 0.9880) | < 0.0001 (< 0.0001, 1.0000, 0.7522) |
| 94 | 0.3186 (0.1317, 0.0793, 0.5366) | < 0.0001 (1.0000, 1.0000, 0.9372) |
| 95 | 0.3170 (0.2964, 0.0782, 0.9481) | < 0.0001 (1.0000, 1.0000, 0.9917) |
| 96 | < 0.0001 (0.6667, 0.0053, 0.9941) | 0.5000 (0.6667, 0.6667, 0.5733) |
| 97 | 1.0000 (0.2964, 1.0000, 0.9436) | < 0.0001 (1.0000, 1.0000, 0.9661) |
| 98 | < 0.0001 (< 0.0001, 0.0095, 0.9930) | < 0.0001 (< 0.0001, 1.0000, 0.8632) |
| 99 | 0.5577 (< 0.0001, 0.1804, 0.3102) | < 0.0001 (1.0000, 1.0000, 0.9597) |
| 100 | 0.8102 (0.3624, 0.3789, 0.7033) | < 0.0001 (1.0000, 1.0000, 0.9982) |
| 101 | 0.3171 (0.1314, 0.0783, 0.7057) | < 0.0001 (1.0000, 1.0000, 0.8316) |
| 102 | 1.8138 (0.2493, 0.1743, 0.2102) | < 0.0001 (1.0000, 1.0000, 0.9059) |
| 103 | 0.9516 (< 0.0001, 0.4655, 0.7102) | < 0.0001 (1.0000, 1.0000, 0.9973) |
| 104 | 0.9505 (0.5084, 0.4647, 0.6920) | < 0.0001 (1.0000, 1.0000, 0.9091) |
| 105 | < 0.0001 (0.6667, 0.0044, 0.9964) | < 0.0001 (1.0000, 1.0000, 0.9154) |
| 106 | < 0.0001 (0.5084, 0.0060, 0.9854) | 0.0544 (1.0000, 1.0000, 0.9999) |
| 107 | < 0.0001 (0.0935, 0.0060, 0.9854) | < 0.0001 (1.0000, 1.0000, 0.8474) |
| 108 | 0.3166 (0.4444, 0.0779, 0.9777) | < 0.0001 (1.0000, 1.0000, 0.9582) |
| 109 | 0.6030 (0.8846, 0.2175, 0.7563) | < 0.0001 (< 0.0001, 1.0000, 0.8338) |
| 110 | < 0.0001 (0.6592, 0.0060, 0.9854) | < 0.0001 (1.0000, 1.0000, 0.9892) |
| 111 | 0.9528 (0.9995, 0.4664, 0.7658) | < 0.0001 (< 0.0001, 1.0000, 0.8341) |
| 112 | 1.5977 (< 0.0001, 0.1658, 0.4799) | < 0.0001 (< 0.0001, 1.0000, 0.7530) |
| 113 | 1.0464 (0.4735, 0.4690, 1.0000) | 1.0264 (0.2776, 0.4342, 0.3181) |
| 114 | 0.8915 (< 0.0001, 0.4075, 0.6843) | < 0.0001 (< 0.0001, 1.0000, 0.8333) |
| 115 | 0.3171 (0.6944, 0.0782, 0.9290) | < 0.0001 (1.0000, 1.0000, 0.9908) |
| 116 | 0.3104 (0.8889, 0.0736, 0.8645) | 0.1594 (0.9972, 0.9890, 0.8846) |
| 117 | < 0.0001 (1.0000, 0.0425, 0.9973) | 0.5000 (0.9630, 0.8889, 0.8133) |
| 118 | 0.9529 (< 0.0001, 0.4665, 1.0000) | < 0.0001 (1.0000, 1.0000, 0.9087) |
| 119 | 1.5989 (< 0.0001, 0.1655, 0.1022) | < 0.0001 (1.0000, 1.0000, 0.8569) |
| 120 | < 0.0001 (< 0.0001, 0.0037, 0.9995) | < 0.0001 (1.0000, 1.0000, 0.8932) |
| 121 | 2.7994 (< 0.0001, 0.0065, 0.9361) | < 0.0001 (< 0.0001, 1.0000, 0.7454) |
| 122 | < 0.0001 (< 0.0001, 0.0060, 0.9854) | < 0.0001 (< 0.0001, 1.0000, 0.6621) |
| 123 | < 0.0001 (0.0026, 0.0061, 0.9948) | < 0.0001 (< 0.0001, 1.0000, 0.7334) |
| 124 | 0.3480 (< 0.0001, 0.1006, 0.8104) | 0.5143 (0.6870, 0.6840, 0.4395) |
| 125 | 1.0000 (< 0.0001, 1.0000, 0.8886) | < 0.0001 (1.0000, 1.0000, 0.9596) |
| 126 | < 0.0001 (0.1901, 0.0126, 0.9992) | < 0.0001 (1.0000, 1.0000, 0.9536) |
| 127 | < 0.0001 (0.2963, 0.0107, 0.9999) | < 0.0001 (1.0000, 1.0000, 0.9567) |
| 128 | 0.6134 (0.9074, 0.2261, 0.5668) | < 0.0001 (1.0000, 1.0000, 0.9881) |
| 129 | < 0.0001 (0.3625, 0.0117, 0.9998) | 0.2107 (0.9657, 0.9739, 0.8163) |
| 130 | 1.0000 (0.4445, 1.0000, 0.9174) | < 0.0001 (1.0000, 1.0000, 0.9927) |
| 131 | < 0.0001 (1.0000, 0.0056, 0.9999) | < 0.0001 (1.0000, 1.0000, 0.9433) |
| 132 | < 0.0001 (< 0.0001, 0.0060, 0.9905) | < 0.0001 (< 0.0001, 1.0000, 0.8055) |
| 133 | 0.3477 (0.7922, 0.1004, 0.9440) | < 0.0001 (< 0.0001, 1.0000, 0.7525) |
| 134 | 1.0000 (0.6991, 1.0000, 0.9870) | < 0.0001 (1.0000, 1.0000, 0.9673) |
| 135 | 0.2226 (0.2865, 0.0231, 0.9358) | < 0.0001 (1.0000, 1.0000, 0.8770) |
| 136 | 1.0000 (0.6667, 1.0000, 0.9569) | < 0.0001 (0.0010, 1.0000, 0.8341) |
| 137 | 0.3480 (0.4883, 0.1006, 1.0000) | < 0.0001 (< 0.0001, 1.0000, 0.8417) |
| 138 | < 0.0001 (1.0000, 0.0053, 0.9941) | < 0.0001 (1.0000, 1.0000, 0.8711) |
| 139 | < 0.0001 (1.0000, 0.0061, 1.0000) | < 0.0001 (1.0000, 1.0000, 0.9597) |
| 140 | < 0.0001 (0.4445, 0.0050, 0.9999) | < 0.0001 (1.0000, 1.0000, 0.9567) |
| 141 | < 0.0001 (0.7774, 0.0065, 0.9733) | < 0.0001 (< 0.0001, 1.0000, 0.7242) |
| 142 | 1.0451 (0.3420, 0.4698, 0.8534) | < 0.0001 (< 0.0001, 1.0000, 0.7522) |
| 143 | 0.3013 (0.6882, 0.0674, 0.9831) | < 0.0001 (< 0.0001, 1.0000, 0.6233) |
| 144 | 0.3245 (0.6883, 0.0835, 0.8500) | < 0.0001 (1.0000, 1.0000, 0.9600) |
| 145 | 2.4529 (0.6950, 0.0195, 0.2250) | < 0.0001 (1.0000, 1.0000, 0.9640) |
| 146 | < 0.0001 (0.4487, 0.0060, 0.9957) | 0.5001 (0.8889, 0.8889, 0.8177) |
| 147 | < 0.0001 (0.6449, 0.0050, 1.0000) | < 0.0001 (1.0000, 1.0000, 0.9166) |
| 148 | 0.3170 (0.6746, 0.0782, 0.9290) | 0.4614 (0.9020, 0.9003, 0.6823) |
| 149 | < 0.0001 (0.9468, 0.0093, 1.0000) | < 0.0001 (1.0000, 1.0000, 0.9872) |
| 150 | 1.0000 (0.9258, 1.0000, 0.9490) | < 0.0001 (1.0000, 1.0000, 0.9568) |
| 151 | < 0.0001 (0.0015, 0.0061, 1.0000) | < 0.0001 (1.0000, 1.0000, 0.9088) |
| 152 | 1.9293 (0.1843, 0.0736, 0.1437) | < 0.0001 (< 0.0001, 1.0000, 0.8338) |
| 153 | < 0.0001 (0.0878, 0.0060, 0.9854) | < 0.0001 (< 0.0001, 1.0000, 0.9110) |
| 154 | < 0.0001 (0.8002, 0.0060, 0.9904) | < 0.0001 (< 0.0001, 1.0000, 0.6216) |
| 155 | < 0.0001 (0.0356, 0.0053, 0.9754) | < 0.0001 (< 0.0001, 1.0000, 0.6233) |
| 156 | < 0.0001 (< 0.0001, 0.0423, 0.9997) | 0.4999 (0.9822, 0.8889, 0.8600) |
| 157 | < 0.0001 (0.9826, 0.0087, 0.9999) | < 0.0001 (1.0000, 1.0000, 0.9086) |
| 158 | 0.9535 (0.3048, 0.4669, 0.9658) | < 0.0001 (1.0000, 1.0000, 0.9685) |
| 159 | < 0.0001 (0.7130, 0.0083, 0.9999) | < 0.0001 (< 0.0001, 1.0000, 0.8341) |
| 160 | 0.5843 (0.5084, 0.2022, 0.4751) | < 0.0001 (1.0000, 1.0000, 0.9971) |
| 161 | < 0.0001 (0.6991, 0.0061, 0.9988) | 0.2222 (0.9909, 0.9709, 0.7852) |
| 162 | 1.0000 (< 0.0001, 1.0000, 0.9870) | < 0.0001 (1.0000, 1.0000, 0.9774) |
| 163 | < 0.0001 (0.2966, 0.0065, 0.9933) | < 0.0001 (1.0000, 1.0000, 1.0000) |
| 164 | 1.0000 (0.6667, 1.0000, 0.9525) | < 0.0001 (< 0.0001, 1.0000, 0.7520) |
| 165 | < 0.0001 (0.1975, 0.0060, 0.9854) | < 0.0001 (< 0.0001, 1.0000, 0.8341) |
| 166 | 1.6724 (< 0.0001, 0.1719, 0.7703) | < 0.0001 (< 0.0001, 1.0000, 0.7522) |
| 167 | 0.2226 (0.1122, 0.0231, 0.9358) | < 0.0001 (< 0.0001, 1.0000, 0.8498) |
| 168 | < 0.0001 (0.4447, 0.0087, 1.0000) | < 0.0001 (< 0.0001, 1.0000, 0.8338) |
| 169 | 0.3244 (0.7130, 0.0834, 0.4299) | < 0.0001 (1.0000, 1.0000, 0.9993) |
| 170 | < 0.0001 (< 0.0001, 0.0065, 0.9994) | < 0.0001 (1.0000, 1.0000, 0.9880) |
| 171 | < 0.0001 (0.7130, 0.0050, 1.0000) | < 0.0001 (1.0000, 1.0000, 0.9590) |
| 172 | < 0.0001 (0.6667, 0.0060, 0.9957) | < 0.0001 (1.0000, 1.0000, 0.9167) |
| 173 | < 0.0001 (0.3526, 0.0050, 0.9997) | < 0.0001 (1.0000, 1.0000, 0.9387) |
| 174 | 1.0000 (0.0572, 1.0000, 0.8456) | < 0.0001 (1.0000, 1.0000, 0.8931) |
| 175 | < 0.0001 (0.5927, 0.0073, 0.9885) | < 0.0001 (1.0000, 1.0000, 0.8568) |
| 176 | < 0.0001 (0.5551, 0.0087, 0.9993) | < 0.0001 (1.0000, 1.0000, 0.9282) |
| 177 | < 0.0001 (0.4601, 0.0093, 0.9999) | < 0.0001 (1.0000, 1.0000, 0.9662) |
| 178 | 0.6966 (0.9907, 0.2934, 0.6322) | < 0.0001 (1.0000, 1.0000, 0.9607) |
| 179 | < 0.0001 (0.5084, 0.0425, 0.9973) | < 0.0001 (< 0.0001, 1.0000, 0.8333) |
| 180 | 1.0000 (0.5084, 1.0000, 0.9611) | < 0.0001 (1.0000, 1.0000, 0.9687) |
| 181 | < 0.0001 (0.0476, 0.0061, 0.9978) | 1.5000 (0.1976, 0.2963, 0.1106) |
| 182 | < 0.0001 (0.4444, 0.0056, 1.0000) | < 0.0001 (< 0.0001, 1.0000, 0.6811) |
| 183 | < 0.0001 (0.1843, 0.0053, 0.9675) | < 0.0001 (1.0000, 1.0000, 0.9315) |
| 184 | < 0.0001 (< 0.0001, 0.0065, 1.0000) | < 0.0001 (< 0.0001, 1.0000, 0.8338) |
| 185 | < 0.0001 (0.0292, 0.0093, 1.0000) | 0.0104 (0.9874, 0.9999, 0.4414) |
| 186 | < 0.0001 (0.9998, 0.0073, 0.9885) | < 0.0001 (1.0000, 1.0000, 0.9353) |
| 187 | < 0.0001 (0.6950, 0.0065, 0.9894) | < 0.0001 (1.0000, 1.0000, 0.9052) |
| 188 | 0.9522 (0.6944, 0.4660, 1.0000) | 0.1562 (0.9921, 0.9896, 0.7788) |
| 189 | < 0.0001 (0.1317, 0.0065, 0.9894) | 0.3124 (0.9699, 0.9614, 0.6146) |
| 190 | < 0.0001 (0.9889, 0.0087, 0.9989) | 0.5000 (0.8889, 0.8889, 0.7009) |
| 191 | 1.0000 (0.7587, 1.0000, 0.9798) | 0.3344 (0.9856, 0.9968, 0.4430) |
| 192 | < 0.0001 (0.0619, 0.0087, 1.0000) | < 0.0001 (< 0.0001, 1.0000, 0.6985) |
| 193 | 0.6040 (< 0.0001, 0.2184, 0.9454) | < 0.0001 (< 0.0001, 1.0000, 0.7454) |
| 194 | 1.0000 (0.5084, 1.0000, 0.9431) | 0.5000 (0.8889, 0.8889, 0.6347) |
| 195 | 1.6229 (< 0.0001, 0.1853, 0.4056) | < 0.0001 (< 0.0001, 1.0000, 0.8417) |
| 196 | < 0.0001 (0.3526, 0.0061, 0.9948) | 0.4223 (0.8080, 0.9186, 0.4474) |
| 197 | < 0.0001 (< 0.0001, 0.0083, 0.9997) | 0.5000 (0.9547, 0.8889, 0.7675) |
| 198 | 0.4164 (0.8398, 0.1528, 0.9999) | < 0.0001 (< 0.0001, 1.0000, 0.8417) |
| 199 | 0.3482 (0.1842, 0.1007, 0.9977) | < 0.0001 (1.0000, 1.0000, 0.9356) |
| 200 | < 0.0001 (0.6668, 0.0041, 0.9905) | < 0.0001 (1.0000, 1.0000, 0.9576) |
| 201 | < 0.0001 (0.0818, 0.0142, 0.9763) | < 0.0001 (1.0000, 1.0000, 0.9354) |
| 202 | < 0.0001 (0.0047, 0.0060, 0.9854) | < 0.0001 (1.0000, 1.0000, 0.9574) |
| 203 | < 0.0001 (0.5084, 0.0044, 0.9995) | < 0.0001 (1.0000, 1.0000, 0.9999) |
| 204 | 1.0000 (1.0000, 1.0000, 0.8871) | < 0.0001 (1.0000, 1.0000, 0.9687) |
| 205 | < 0.0001 (0.9999, 0.0050, 0.9988) | < 0.0001 (1.0000, 1.0000, 0.9917) |
| 206 | 0.6336 (0.1975, 0.2426, 0.5016) | < 0.0001 (1.0000, 1.0000, 0.9088) |
| 207 | 0.3014 (0.5550, 0.0675, 0.9832) | < 0.0001 (1.0000, 1.0000, 0.9374) |
| 208 | < 0.0001 (0.4444, 0.0085, 0.9980) | < 0.0001 (1.0000, 1.0000, 0.9958) |
| 209 | < 0.0001 (0.4445, 0.0099, 0.9954) | < 0.0001 (< 0.0001, 1.0000, 0.7546) |
| 210 | < 0.0001 (< 0.0001, 0.0131, 1.0000) | < 0.0001 (< 0.0001, 1.0000, 0.7770) |
| 211 | 0.8770 (0.9962, 0.4081, 0.7941) | < 0.0001 (1.0000, 1.0000, 0.9875) |
| 212 | < 0.0001 (0.0051, 0.0085, 0.9998) | < 0.0001 (1.0000, 1.0000, 0.9903) |
| 213 | < 0.0001 (0.6783, 0.0037, 0.9987) | < 0.0001 (< 0.0001, 1.0000, 0.9479) |
| 214 | 0.3634 (0.1842, 0.1120, 0.7574) | 0.2310 (0.9198, 0.9684, 0.5785) |
| 215 | 1.0000 (1.0000, 1.0000, 0.9411) | < 0.0001 (1.0000, 1.0000, 0.9260) |
| 216 | < 0.0001 (1.0000, 0.0131, 0.9998) | < 0.0001 (< 0.0001, 1.0000, 0.8495) |
| 217 | < 0.0001 (< 0.0001, 0.0425, 0.9944) | < 0.0001 (< 0.0001, 1.0000, 0.7385) |
| 218 | < 0.0001 (0.0339, 0.0050, 0.9951) | < 0.0001 (1.0000, 1.0000, 0.9958) |
| 219 | 1.0000 (0.4829, 1.0000, 0.9525) | 1.5166 (0.6291, 0.6368, 0.6889) |
| 220 | < 0.0001 (0.9877, 0.0343, 0.9995) | < 0.0001 (< 0.0001, 1.0000, 0.8443) |
| 221 | < 0.0001 (< 0.0001, 0.0050, 1.0000) | < 0.0001 (< 0.0001, 1.0000, 0.8196) |
| 222 | < 0.0001 (0.7130, 0.0099, 0.9984) | < 0.0001 (< 0.0001, 1.0000, 0.8964) |
| 223 | 1.0000 (1.0000, 1.0000, 0.9216) | < 0.0001 (1.0000, 1.0000, 0.9546) |
| 224 | 0.3359 (0.0078, 0.0917, 0.6903) | < 0.0001 (< 0.0001, 1.0000, 0.8196) |
| 225 | 1.0000 (0.6669, 1.0000, 0.8693) | < 0.0001 (1.0000, 1.0000, 0.9922) |
| 226 | < 0.0001 (0.9992, 0.0050, 0.9980) | < 0.0001 (1.0000, 1.0000, 0.9795) |
| 227 | < 0.0001 (0.6993, 0.0425, 0.9984) | < 0.0001 (< 0.0001, 1.0000, 0.9008) |
| 228 | 0.9574 (0.9955, 0.4698, 0.9392) | < 0.0001 (1.0000, 1.0000, 0.9780) |
| 229 | < 0.0001 (< 0.0001, 0.0053, 0.9997) | < 0.0001 (1.0000, 1.0000, 0.9535) |
| 230 | 0.3166 (0.7130, 0.0779, 0.9516) | < 0.0001 (1.0000, 1.0000, 0.9467) |
| 231 | < 0.0001 (0.4444, 0.0060, 1.0000) | 0.2500 (0.9630, 0.9630, 0.7951) |
| 232 | < 0.0001 (0.2963, 0.0056, 0.9998) | < 0.0001 (1.0000, 1.0000, 0.9987) |
| 233 | < 0.0001 (0.4445, 0.0061, 0.9937) | < 0.0001 (< 0.0001, 1.0000, 0.8443) |
| 234 | 1.0000 (0.2963, 1.0000, 0.9798) | < 0.0001 (1.0000, 1.0000, 0.9892) |
| 235 | 1.0000 (0.0821, 1.0000, 0.9870) | < 0.0001 (1.0000, 1.0000, 0.9164) |
| 236 | < 0.0001 (< 0.0001, 0.0065, 0.9733) | < 0.0001 (1.0000, 1.0000, 0.9264) |
| 237 | < 0.0001 (< 0.0001, 0.0037, 0.9769) | < 0.0001 (1.0000, 1.0000, 0.9744) |
| 238 | 1.2076 (0.1976, 0.3574, 0.8654) | < 0.0001 (1.0000, 1.0000, 0.9929) |
| 239 | < 0.0001 (0.5084, 0.0060, 0.9904) | < 0.0001 (1.0000, 1.0000, 0.9981) |
| 240 | < 0.0001 (0.7130, 0.0061, 1.0000) | < 0.0001 (1.0000, 1.0000, 0.9972) |
| 241 | < 0.0001 (< 0.0001, 0.0050, 0.9951) | 0.6670 (0.9992, 0.9990, 0.6138) |
| 242 | < 0.0001 (0.1843, 0.0041, 0.9996) | < 0.0001 (1.0000, 1.0000, 0.9986) |
| 243 | 0.4162 (0.0878, 0.1527, 0.9908) | 0.4977 (0.6929, 0.6974, 0.5059) |
| 244 | < 0.0001 (0.2684, 0.0397, 0.9998) | < 0.0001 (1.0000, 1.0000, 0.9907) |
| 245 | 0.6962 (0.6784, 0.2930, 1.0000) | < 0.0001 (1.0000, 1.0000, 0.9590) |
| 246 | < 0.0001 (< 0.0001, 0.0060, 0.9966) | < 0.0001 (1.0000, 1.0000, 0.9638) |
| 247 | 1.0000 (< 0.0001, 1.0000, 0.9490) | < 0.0001 (1.0000, 1.0000, 0.9612) |
| 248 | 0.3358 (0.0392, 0.0916, 0.6905) | < 0.0001 (1.0000, 1.0000, 0.9599) |
| 249 | 1.0000 (0.9764, 1.0000, 0.9536) | < 0.0001 (1.0000, 1.0000, 0.9763) |
| 250 | 1.0000 (0.3349, 1.0000, 0.9798) | < 0.0001 (1.0000, 1.0000, 0.9780) |
| 251 | < 0.0001 (0.4823, 0.0060, 1.0000) | < 0.0001 (1.0000, 1.0000, 0.9590) |
| 252 | < 0.0001 (0.0879, 0.0397, 0.9982) | < 0.0001 (< 0.0001, 1.0000, 0.9479) |
| 253 | < 0.0001 (0.1321, 0.0041, 0.9970) | 0.6679 (0.9967, 0.9964, 0.6118) |
| 254 | < 0.0001 (0.6460, 0.0053, 0.9990) | 0.2886 (0.9710, 0.9684, 0.6489) |
| 255 | 0.7224 (< 0.0001, 0.3135, 0.9756) | < 0.0001 (< 0.0001, 1.0000, 0.9479) |
| 256 | < 0.0001 (< 0.0001, 0.0060, 0.9854) | 0.4964 (0.6926, 0.6979, 0.4855) |
| 257 | < 0.0001 (0.8536, 0.0142, 0.9915) | < 0.0001 (1.0000, 1.0000, 0.9978) |
| 258 | < 0.0001 (0.6667, 0.0061, 0.9961) | 1.2500 (0.5706, 0.5706, 0.3133) |
| 259 | < 0.0001 (0.0474, 0.0425, 0.9973) | < 0.0001 (< 0.0001, 1.0000, 0.7546) |
| 260 | < 0.0001 (0.4446, 0.0083, 0.9996) | < 0.0001 (1.0000, 1.0000, 0.9176) |
| 261 | 1.0000 (0.0240, 1.0000, 0.9480) | < 0.0001 (1.0000, 1.0000, 0.9770) |
| 262 | < 0.0001 (0.1977, 0.0061, 0.9995) | < 0.0001 (< 0.0001, 1.0000, 0.8430) |
| 263 | 2.8059 (0.5084, 0.0064, 0.3367) | 0.2500 (0.9630, 0.9630, 0.8299) |
| 264 | 1.0000 (< 0.0001, 1.0000, 0.8516) | < 0.0001 (< 0.0001, 1.0000, 0.7983) |
| 265 | < 0.0001 (0.6911, 0.0056, 0.9994) | < 0.0001 (1.0000, 1.0000, 0.9599) |
| 266 | 1.0000 (< 0.0001, 1.0000, 0.9798) | < 0.0001 (1.0000, 1.0000, 0.9837) |
| 267 | 1.5629 (< 0.0001, 0.1360, 0.4830) | < 0.0001 (1.0000, 1.0000, 0.9744) |
| 268 | 0.3995 (0.0010, 0.1397, 0.9789) | 0.2266 (0.0032, 0.9697, 0.8319) |
| 269 | < 0.0001 (< 0.0001, 0.0056, 0.9884) | 0.5047 (0.8628, 0.8684, 0.8819) |
| 270 | 0.8366 (0.4823, 0.3752, 0.8017) | < 0.0001 (1.0000, 1.0000, 0.9977) |
| 271 | 0.9204 (0.0585, 0.4421, 0.6328) | 0.5000 (0.8889, 0.8889, 0.7417) |
| 272 | 0.6037 (0.9803, 0.2181, 0.8782) | < 0.0001 (1.0000, 1.0000, 0.9826) |
| 273 | 1.3416 (0.9664, 0.2465, 0.5301) | 0.4874 (0.7047, 0.6839, 0.3863) |
| 274 | 1.0000 (0.4992, 1.0000, 0.9230) | 0.4326 (0.0036, 0.9088, 0.5684) |
| 275 | 1.0000 (0.0878, 1.0000, 0.9342) | < 0.0001 (1.0000, 1.0000, 0.9995) |
| 276 | 0.6973 (< 0.0001, 0.2939, 0.7809) | < 0.0001 (< 0.0001, 1.0000, 0.8334) |
| 277 | 1.0000 (0.2967, 1.0000, 0.9525) | < 0.0001 (1.0000, 1.0000, 0.9614) |
| 278 | 1.5598 (< 0.0001, 0.2041, 0.8919) | < 0.0001 (< 0.0001, 1.0000, 0.8553) |
| 279 | 2.7967 (0.6324, 0.0065, 0.7256) | < 0.0001 (< 0.0001, 1.0000, 0.7527) |
| 280 | 1.0000 (0.2584, 1.0000, 0.9525) | < 0.0001 (1.0000, 1.0000, 0.9795) |
| 281 | < 0.0001 (< 0.0001, 0.0425, 0.9937) | < 0.0001 (1.0000, 1.0000, 0.9384) |
| 282 | < 0.0001 (0.8988, 0.0397, 0.9960) | < 0.0001 (1.0000, 1.0000, 0.9998) |
| 283 | 0.3992 (1.0000, 0.1395, 0.9475) | 0.1667 (0.9877, 0.9877, 0.9132) |
| 284 | < 0.0001 (0.0878, 0.0397, 0.9991) | < 0.0001 (< 0.0001, 1.0000, 0.9479) |
| 285 | < 0.0001 (0.9944, 0.0083, 1.0000) | < 0.0001 (1.0000, 1.0000, 0.9767) |
| 286 | 0.3187 (0.1842, 0.0794, 0.9797) | < 0.0001 (1.0000, 1.0000, 0.9546) |
| 287 | < 0.0001 (0.9990, 0.0093, 1.0000) | < 0.0001 (1.0000, 1.0000, 0.9900) |
| 288 | < 0.0001 (< 0.0001, 0.0061, 0.9978) | < 0.0001 (1.0000, 1.0000, 0.9927) |
| 289 | < 0.0001 (< 0.0001, 0.0142, 0.9996) | < 0.0001 (1.0000, 1.0000, 0.9597) |
| 290 | < 0.0001 (0.9176, 0.0037, 0.9769) | < 0.0001 (1.0000, 1.0000, 0.9164) |
| 291 | < 0.0001 (0.4990, 0.0117, 1.0000) | < 0.0001 (1.0000, 1.0000, 0.9599) |
| 292 | < 0.0001 (0.1975, 0.0053, 0.9753) | < 0.0001 (1.0000, 1.0000, 0.9739) |
| 293 | 1.0000 (0.4406, 1.0000, 0.8871) | < 0.0001 (< 0.0001, 1.0000, 0.7527) |
| 294 | < 0.0001 (0.0392, 0.0126, 0.9955) | < 0.0001 (1.0000, 1.0000, 0.9622) |
| 295 | < 0.0001 (0.2963, 0.0060, 0.9990) | 0.2500 (0.9630, 0.9630, 0.9423) |
| 296 | 1.0000 (0.6911, 1.0000, 0.9870) | < 0.0001 (1.0000, 1.0000, 0.9405) |
| 297 | 0.5574 (0.2963, 0.1801, 0.8778) | < 0.0001 (< 0.0001, 1.0000, 0.7970) |
| 298 | < 0.0001 (0.0933, 0.0053, 0.9753) | < 0.0001 (< 0.0001, 1.0000, 0.8964) |
| 299 | 1.0000 (< 0.0001, 1.0000, 0.9342) | < 0.0001 (1.0000, 1.0000, 0.9881) |
| 300 | < 0.0001 (0.1051, 0.0065, 0.9894) | 0.9997 (0.4577, 0.4821, 0.3296) |
| 301 | < 0.0001 (0.2963, 0.0117, 0.9993) | < 0.0001 (1.0000, 1.0000, 0.9410) |
| 302 | 1.9536 (0.1840, 0.1485, 0.6528) | < 0.0001 (< 0.0001, 1.0000, 0.8196) |
| 303 | 1.0000 (0.7673, 1.0000, 0.9351) | < 0.0001 (1.0000, 1.0000, 0.9780) |
| 304 | < 0.0001 (0.6667, 0.0060, 0.9854) | < 0.0001 (1.0000, 1.0000, 0.9926) |
| 305 | 1.0000 (1.0000, 1.0000, 0.9525) | < 0.0001 (1.0000, 1.0000, 0.9164) |
| 306 | < 0.0001 (< 0.0001, 0.0107, 0.9960) | < 0.0001 (1.0000, 1.0000, 0.9589) |
| 307 | 1.0000 (0.9952, 1.0000, 0.9216) | < 0.0001 (1.0000, 1.0000, 0.9582) |
| 308 | < 0.0001 (0.2584, 0.0061, 0.9999) | < 0.0001 (< 0.0001, 1.0000, 0.9008) |
| 309 | < 0.0001 (0.4883, 0.0050, 0.9951) | < 0.0001 (1.0000, 1.0000, 0.9892) |
| 310 | 1.0000 (< 0.0001, 1.0000, 0.9798) | < 0.0001 (1.0000, 1.0000, 0.9676) |
| 311 | < 0.0001 (0.4445, 0.0083, 0.9996) | < 0.0001 (1.0000, 1.0000, 0.9789) |
| 312 | < 0.0001 (0.0391, 0.0065, 0.9933) | < 0.0001 (1.0000, 1.0000, 0.9386) |
| 313 | < 0.0001 (0.6603, 0.0397, 0.9960) | < 0.0001 (< 0.0001, 1.0000, 0.8196) |
| 314 | 0.3481 (< 0.0001, 0.1007, 0.9583) | < 0.0001 (1.0000, 1.0000, 0.9975) |
| 315 | < 0.0001 (0.7130, 0.0127, 1.0000) | < 0.0001 (< 0.0001, 1.0000, 0.7970) |
| 316 | 1.0000 (< 0.0001, 1.0000, 0.9351) | < 0.0001 (< 0.0001, 1.0000, 0.7229) |
| 317 | < 0.0001 (0.9976, 0.0056, 0.9999) | < 0.0001 (1.0000, 1.0000, 0.9984) |
| 318 | < 0.0001 (< 0.0001, 0.0065, 0.9733) | < 0.0001 (1.0000, 1.0000, 0.9767) |
| 319 | < 0.0001 (0.2585, 0.0037, 0.9770) | < 0.0001 (1.0000, 1.0000, 0.9925) |
| 320 | < 0.0001 (0.9998, 0.0087, 0.9982) | < 0.0001 (1.0000, 1.0000, 0.9467) |
| 321 | < 0.0001 (< 0.0001, 0.0083, 0.9999) | < 0.0001 (1.0000, 1.0000, 0.9957) |
| 322 | < 0.0001 (0.2879, 0.0050, 1.0000) | < 0.0001 (< 0.0001, 1.0000, 0.8965) |
| 323 | < 0.0001 (0.1312, 0.0060, 1.0000) | < 0.0001 (1.0000, 1.0000, 0.9384) |
| 324 | 0.4454 (0.6667, 0.0934, 0.8752) | < 0.0001 (1.0000, 1.0000, 0.9840) |
| 325 | < 0.0001 (0.2963, 0.0107, 1.0000) | < 0.0001 (0.0004, 1.0000, 0.9984) |
| 326 | 0.6336 (< 0.0001, 0.2427, 0.7357) | < 0.0001 (< 0.0001, 1.0000, 0.7970) |
| 327 | 0.3245 (1.0000, 0.0835, 0.9890) | < 0.0001 (1.0000, 1.0000, 0.9969) |
| 328 | < 0.0001 (0.2964, 0.0060, 0.9904) | < 0.0001 (1.0000, 1.0000, 0.9944) |
| 329 | < 0.0001 (0.2584, 0.0093, 0.9962) | < 0.0001 (1.0000, 1.0000, 0.9935) |
| 330 | < 0.0001 (0.0280, 0.0050, 0.9988) | < 0.0001 (1.0000, 1.0000, 0.9611) |
| 331 | < 0.0001 (0.0087, 0.0060, 0.9998) | 0.4976 (0.6929, 0.6974, 0.5058) |
| 332 | < 0.0001 (0.6667, 0.0065, 0.9982) | < 0.0001 (1.0000, 1.0000, 0.9770) |
| 333 | < 0.0001 (0.4359, 0.0083, 0.9948) | 1.0812 (0.9135, 0.7912, 0.4216) |
| 334 | < 0.0001 (0.6667, 0.0060, 0.9969) | < 0.0001 (< 0.0001, 1.0000, 0.8196) |
| 335 | 1.0000 (0.4887, 1.0000, 0.9870) | < 0.0001 (1.0000, 1.0000, 0.9836) |
| 336 | 0.4459 (0.3625, 0.0937, 0.8749) | 1.0000 (0.7408, 0.7408, 0.5671) |
| 337 | 0.6141 (0.1758, 0.2267, 0.9359) | < 0.0001 (0.0017, 1.0000, 0.9996) |
| 338 | 1.0000 (0.6944, 1.0000, 0.9870) | < 0.0001 (1.0000, 1.0000, 0.9787) |
| 339 | 0.6719 (0.5084, 0.2737, 0.4801) | 0.3715 (0.9702, 0.9266, 0.8625) |
| 340 | < 0.0001 (0.5083, 0.0061, 0.9998) | < 0.0001 (< 0.0001, 1.0000, 0.8430) |
| 341 | 2.5659 (1.0000, 0.0107, 0.3064) | < 0.0001 (1.0000, 1.0000, 0.9998) |
| 342 | 1.0000 (0.0817, 1.0000, 0.8657) | < 0.0001 (1.0000, 1.0000, 0.9849) |
| 343 | < 0.0001 (0.6603, 0.0425, 0.9973) | < 0.0001 (1.0000, 1.0000, 0.9590) |
| 344 | < 0.0001 (0.6667, 0.0061, 0.9988) | < 0.0001 (1.0000, 1.0000, 0.9378) |
| 345 | 1.0000 (0.0758, 1.0000, 0.9404) | < 0.0001 (< 0.0001, 1.0000, 0.8443) |
| 346 | 0.3480 (0.4444, 0.1006, 0.9926) | < 0.0001 (< 0.0001, 1.0000, 0.8964) |
| 347 | < 0.0001 (0.4446, 0.0085, 0.9946) | < 0.0001 (1.0000, 1.0000, 0.9987) |
| 348 | < 0.0001 (0.4602, 0.0037, 0.9974) | < 0.0001 (1.0000, 1.0000, 0.9467) |
| 349 | < 0.0001 (0.6667, 0.0142, 0.9997) | < 0.0001 (< 0.0001, 1.0000, 0.7970) |
| 350 | < 0.0001 (0.1317, 0.0087, 0.9997) | < 0.0001 (1.0000, 1.0000, 0.9826) |
| 351 | 0.4164 (1.0000, 0.1528, 0.9907) | < 0.0001 (1.0000, 1.0000, 0.9431) |
| 352 | 1.3007 (1.0000, 0.3610, 0.9209) | < 0.0001 (1.0000, 1.0000, 0.9769) |
| 353 | < 0.0001 (1.0000, 0.0061, 1.0000) | < 0.0001 (1.0000, 1.0000, 0.9874) |
| 354 | 1.2072 (0.1003, 0.3576, 0.8656) | < 0.0001 (1.0000, 1.0000, 0.9981) |
| 355 | < 0.0001 (0.6667, 0.0083, 0.9982) | < 0.0001 (1.0000, 1.0000, 0.9460) |
| 356 | 0.2786 (0.0033, 0.0528, 0.9443) | < 0.0001 (< 0.0001, 1.0000, 0.8964) |
| 357 | 0.3245 (0.1318, 0.0835, 0.8504) | < 0.0001 (< 0.0001, 1.0000, 0.8430) |
| 358 | < 0.0001 (0.2963, 0.0126, 1.0000) | < 0.0001 (1.0000, 1.0000, 0.9846) |
| 359 | 1.0000 (0.0261, 1.0000, 0.8984) | < 0.0001 (1.0000, 1.0000, 0.9831) |
| 360 | 0.3014 (1.0000, 0.0675, 0.8851) | < 0.0001 (1.0000, 1.0000, 0.9768) |
| 361 | < 0.0001 (0.5798, 0.0060, 0.9896) | < 0.0001 (1.0000, 1.0000, 0.9676) |
| 362 | < 0.0001 (0.7130, 0.0061, 0.9807) | < 0.0001 (1.0000, 1.0000, 0.9880) |
| 363 | < 0.0001 (0.7065, 0.0061, 1.0000) | < 0.0001 (1.0000, 1.0000, 0.9890) |
| 364 | < 0.0001 (0.1333, 0.0087, 0.9949) | < 0.0001 (1.0000, 1.0000, 0.9795) |
| 365 | < 0.0001 (0.4444, 0.0093, 1.0000) | < 0.0001 (< 0.0001, 1.0000, 0.8152) |
| 366 | 0.9060 (0.0668, 0.4311, 0.8422) | 0.5000 (0.7407, 0.8889, 0.4919) |
| 367 | 1.0000 (0.3624, 1.0000, 0.9870) | < 0.0001 (1.0000, 1.0000, 0.9444) |
| 368 | < 0.0001 (< 0.0001, 0.0060, 0.9947) | 1.0000 (0.7407, 0.7407, 0.5476) |
| 369 | 0.3358 (0.4602, 0.0916, 0.8795) | < 0.0001 (1.0000, 1.0000, 0.9801) |
| 370 | < 0.0001 (0.5084, 0.0061, 0.9991) | < 0.0001 (< 0.0001, 1.0000, 0.8152) |
| 371 | < 0.0001 (0.1791, 0.0099, 0.9984) | 0.5000 (0.8889, 0.8889, 0.8055) |
| 372 | < 0.0001 (0.4444, 0.0053, 0.9995) | < 0.0001 (< 0.0001, 1.0000, 0.8374) |
| 373 | < 0.0001 (0.3527, 0.0065, 0.9733) | < 0.0001 (< 0.0001, 1.0000, 0.8495) |
| 374 | < 0.0001 (0.1317, 0.0060, 0.9966) | < 0.0001 (1.0000, 1.0000, 0.9993) |
| 375 | < 0.0001 (0.5552, 0.0085, 0.9967) | < 0.0001 (1.0000, 1.0000, 0.9678) |
| 376 | < 0.0001 (0.7130, 0.0061, 0.9808) | < 0.0001 (1.0000, 1.0000, 0.9850) |
| 377 | < 0.0001 (< 0.0001, 0.0065, 0.9976) | < 0.0001 (1.0000, 1.0000, 0.9801) |
| 378 | 0.3611 (< 0.0001, 0.1103, 0.9903) | < 0.0001 (1.0000, 1.0000, 0.9884) |
| 379 | 0.9571 (< 0.0001, 0.4695, 0.4732) | < 0.0001 (1.0000, 1.0000, 0.9933) |
| 380 | 1.0000 (0.6882, 1.0000, 0.8736) | < 0.0001 (1.0000, 1.0000, 0.9793) |
| 381 | 1.0000 (0.5084, 1.0000, 0.9525) | < 0.0001 (1.0000, 1.0000, 0.9883) |
| 382 | < 0.0001 (0.6667, 0.0087, 0.9997) | < 0.0001 (< 0.0001, 1.0000, 0.9049) |
| 383 | 0.8774 (0.4359, 0.4082, 0.3018) | 0.4740 (0.6395, 0.8966, 0.3279) |
| 384 | < 0.0001 (0.6667, 0.0343, 0.9808) | < 0.0001 (1.0000, 1.0000, 0.9933) |
| 385 | < 0.0001 (1.0000, 0.0060, 0.9947) | 0.3651 (0.9617, 0.9273, 0.9582) |
| 386 | < 0.0001 (< 0.0001, 0.0083, 0.9948) | < 0.0001 (< 0.0001, 1.0000, 0.8544) |
| 387 | < 0.0001 (0.4159, 0.0083, 0.9987) | < 0.0001 (< 0.0001, 1.0000, 0.7956) |
| 388 | < 0.0001 (1.0000, 0.0065, 0.9894) | < 0.0001 (1.0000, 1.0000, 0.9767) |
| 389 | < 0.0001 (0.6448, 0.0041, 0.9905) | < 0.0001 (1.0000, 1.0000, 0.9546) |
| 390 | < 0.0001 (0.0586, 0.0056, 0.9876) | < 0.0001 (< 0.0001, 1.0000, 0.7232) |
| 391 | < 0.0001 (< 0.0001, 0.0454, 0.9798) | < 0.0001 (1.0000, 1.0000, 0.9445) |
| 392 | < 0.0001 (< 0.0001, 0.0056, 0.9991) | < 0.0001 (< 0.0001, 1.0000, 0.8430) |
| 393 | < 0.0001 (0.2243, 0.0053, 0.9905) | < 0.0001 (1.0000, 1.0000, 0.9976) |
| 394 | < 0.0001 (0.4890, 0.0056, 0.9943) | < 0.0001 (< 0.0001, 1.0000, 0.8965) |
| 395 | < 0.0001 (0.1314, 0.0087, 0.9843) | < 0.0001 (< 0.0001, 1.0000, 0.8964) |
| 396 | 1.0000 (0.0936, 1.0000, 0.9216) | < 0.0001 (1.0000, 1.0000, 0.9907) |
| 397 | < 0.0001 (< 0.0001, 0.0425, 0.9995) | < 0.0001 (< 0.0001, 1.0000, 0.7283) |
| 398 | 1.0000 (0.6667, 1.0000, 0.8736) | < 0.0001 (1.0000, 1.0000, 0.9546) |
| 399 | < 0.0001 (0.0012, 0.0056, 0.9943) | < 0.0001 (< 0.0001, 1.0000, 0.9186) |
| 400 | < 0.0001 (< 0.0001, 0.0060, 0.9995) | < 0.0001 (< 0.0001, 1.0000, 0.8835) |
| 401 | < 0.0001 (1.0000, 0.0060, 0.9947) | < 0.0001 (< 0.0001, 1.0000, 0.9049) |
| 402 | < 0.0001 (0.0590, 0.0037, 0.9991) | < 0.0001 (1.0000, 1.0000, 0.9515) |
| 403 | 0.3174 (0.4445, 0.0784, 0.8353) | < 0.0001 (< 0.0001, 1.0000, 0.7675) |
| 404 | < 0.0001 (0.3576, 0.0065, 0.9996) | < 0.0001 (1.0000, 1.0000, 0.9907) |
| 405 | 1.0000 (< 0.0001, 1.0000, 0.9490) | 0.1667 (0.9877, 0.9877, 0.9138) |
| 406 | < 0.0001 (< 0.0001, 0.0087, 0.9843) | 0.8655 (0.7899, 0.7814, 0.3987) |
| 407 | < 0.0001 (0.7681, 0.0061, 0.9987) | < 0.0001 (1.0000, 1.0000, 0.9922) |
| 408 | < 0.0001 (0.5084, 0.0060, 1.0000) | < 0.0001 (1.0000, 1.0000, 0.9879) |
| 409 | 1.0000 (0.0174, 1.0000, 0.8736) | < 0.0001 (< 0.0001, 1.0000, 0.7758) |
| 410 | < 0.0001 (0.6944, 0.0061, 1.0000) | < 0.0001 (1.0000, 1.0000, 0.9926) |
| 411 | < 0.0001 (0.6944, 0.0087, 0.9999) | < 0.0001 (< 0.0001, 1.0000, 0.9479) |
| 412 | < 0.0001 (< 0.0001, 0.0107, 1.0000) | 0.4998 (0.8890, 0.8889, 0.6956) |
| 413 | 0.3014 (0.6668, 0.0675, 0.8845) | < 0.0001 (1.0000, 1.0000, 0.9756) |
| 414 | 0.6336 (< 0.0001, 0.2426, 0.5016) | < 0.0001 (< 0.0001, 1.0000, 0.8196) |
| 415 | < 0.0001 (< 0.0001, 0.0061, 0.9948) | < 0.0001 (< 0.0001, 1.0000, 0.8965) |
| 416 | 1.3258 (0.0087, 0.2549, 0.4215) | < 0.0001 (< 0.0001, 1.0000, 0.8544) |
| 417 | 1.0000 (0.7130, 1.0000, 0.9216) | < 0.0001 (0.0297, 1.0000, 0.9942) |
| 418 | < 0.0001 (0.7066, 0.0044, 0.9995) | < 0.0001 (1.0000, 1.0000, 0.9958) |
| 419 | 0.6030 (0.2963, 0.2176, 0.8783) | < 0.0001 (1.0000, 1.0000, 0.9468) |
| 420 | < 0.0001 (0.9984, 0.0060, 1.0000) | < 0.0001 (1.0000, 1.0000, 0.9541) |
| 421 | < 0.0001 (0.2963, 0.0397, 0.9960) | < 0.0001 (1.0000, 1.0000, 0.9431) |
| 422 | 0.3166 (0.7130, 0.0779, 0.9888) | < 0.0001 (< 0.0001, 1.0000, 0.7401) |
| 423 | < 0.0001 (0.0196, 0.0073, 0.9894) | < 0.0001 (< 0.0001, 1.0000, 0.8430) |
| 424 | < 0.0001 (0.7066, 0.0053, 1.0000) | < 0.0001 (< 0.0001, 1.0000, 0.9008) |
| 425 | < 0.0001 (< 0.0001, 0.0060, 0.9999) | < 0.0001 (1.0000, 1.0000, 0.9982) |
| 426 | < 0.0001 (0.4445, 0.0060, 0.9904) | < 0.0001 (< 0.0001, 1.0000, 0.9479) |
| 427 | < 0.0001 (0.4444, 0.0142, 0.9830) | < 0.0001 (1.0000, 1.0000, 0.9164) |
| 428 | 1.0000 (0.1124, 1.0000, 0.9351) | < 0.0001 (1.0000, 1.0000, 0.9967) |
| 429 | 1.0000 (0.2333, 1.0000, 0.9525) | < 0.0001 (1.0000, 1.0000, 0.9675) |
| 430 | < 0.0001 (1.0000, 0.0061, 1.0000) | < 0.0001 (1.0000, 1.0000, 0.9914) |
| 431 | < 0.0001 (0.0880, 0.0060, 0.9808) | < 0.0001 (1.0000, 1.0000, 0.9638) |
| 432 | < 0.0001 (0.5551, 0.0117, 0.9873) | < 0.0001 (< 0.0001, 1.0000, 0.9008) |
| 433 | < 0.0001 (0.4444, 0.0083, 0.9982) | 0.2310 (0.9742, 0.9685, 0.7559) |
| 434 | < 0.0001 (1.0000, 0.0099, 0.9932) | < 0.0001 (1.0000, 1.0000, 0.9093) |
| 435 | < 0.0001 (0.9693, 0.0060, 0.9978) | < 0.0001 (1.0000, 1.0000, 0.9410) |
| 436 | < 0.0001 (0.4485, 0.0093, 0.9967) | < 0.0001 (< 0.0001, 1.0000, 0.8196) |
| 437 | < 0.0001 (0.7130, 0.0050, 1.0000) | < 0.0001 (1.0000, 1.0000, 0.9975) |
| 438 | < 0.0001 (0.2753, 0.0050, 0.9817) | 0.3333 (0.9547, 0.9547, 0.7627) |
| 439 | 1.0000 (0.5927, 1.0000, 0.9525) | < 0.0001 (1.0000, 1.0000, 0.9993) |
| 440 | < 0.0001 (1.0000, 0.0073, 0.9894) | < 0.0001 (< 0.0001, 1.0000, 0.9008) |
| 441 | 0.3925 (0.6988, 0.1343, 0.9911) | < 0.0001 (1.0000, 1.0000, 0.9589) |
| 442 | < 0.0001 (0.4775, 0.0099, 0.9997) | < 0.0001 (1.0000, 1.0000, 0.9844) |
| 443 | < 0.0001 (0.1313, 0.0060, 0.9991) | < 0.0001 (< 0.0001, 1.0000, 0.8495) |
| 444 | < 0.0001 (0.1313, 0.0053, 0.9999) | < 0.0001 (< 0.0001, 1.0000, 0.8430) |
| 445 | < 0.0001 (0.1975, 0.0142, 0.9998) | < 0.0001 (< 0.0001, 1.0000, 0.9064) |
| 446 | < 0.0001 (0.3528, 0.0107, 0.9993) | < 0.0001 (1.0000, 1.0000, 0.9997) |
| 447 | 0.6030 (0.0879, 0.2175, 0.9455) | < 0.0001 (< 0.0001, 1.0000, 0.9479) |
| 448 | < 0.0001 (0.4444, 0.0343, 0.9926) | < 0.0001 (1.0000, 1.0000, 0.9535) |
| 449 | 4.8881 (0.3623, < 0.0001, 0.0378) | < 0.0001 (< 0.0001, 1.0000, 0.7970) |
| 450 | < 0.0001 (0.7793, 0.0060, 0.9947) | < 0.0001 (< 0.0001, 1.0000, 0.8430) |
| 451 | 1.2782 (0.1314, 0.3188, 0.4010) | < 0.0001 (1.0000, 1.0000, 0.9546) |
| 452 | < 0.0001 (0.2822, 0.0050, 0.9980) | < 0.0001 (1.0000, 1.0000, 0.9964) |
| 453 | < 0.0001 (0.2332, 0.0343, 0.9926) | < 0.0001 (1.0000, 1.0000, 0.9831) |
| 454 | 0.6376 (< 0.0001, 0.2459, 0.8650) | < 0.0001 (1.0000, 1.0000, 0.9987) |
| 455 | < 0.0001 (0.9978, 0.0056, 0.9884) | < 0.0001 (1.0000, 1.0000, 0.9942) |
| 456 | < 0.0001 (0.7560, 0.0044, 0.9964) | < 0.0001 (< 0.0001, 1.0000, 0.8544) |
| 457 | < 0.0001 (0.9321, 0.0117, 1.0000) | < 0.0001 (< 0.0001, 1.0000, 0.8152) |
| 458 | < 0.0001 (< 0.0001, 0.0037, 0.9991) | < 0.0001 (< 0.0001, 1.0000, 0.7799) |
| 459 | 0.3170 (0.1977, 0.0782, 0.9969) | < 0.0001 (1.0000, 1.0000, 0.9780) |
| 460 | < 0.0001 (0.3625, 0.0050, 0.9992) | 0.3599 (0.9720, 0.9263, 0.7009) |
| 461 | < 0.0001 (0.7130, 0.0093, 0.9962) | < 0.0001 (< 0.0001, 1.0000, 0.8900) |
| 462 | 0.3188 (< 0.0001, 0.0794, 0.9999) | < 0.0001 (1.0000, 1.0000, 0.9787) |
| 463 | < 0.0001 (1.0000, 0.0061, 0.9990) | < 0.0001 (< 0.0001, 1.0000, 0.7229) |
| 464 | < 0.0001 (0.6667, 0.0093, 0.9989) | < 0.0001 (< 0.0001, 1.0000, 0.9049) |
| 465 | < 0.0001 (0.6667, 0.0131, 0.9963) | < 0.0001 (< 0.0001, 1.0000, 0.8176) |
| 466 | 2.5042 (0.6667, 0.0256, 0.6581) | < 0.0001 (1.0000, 1.0000, 0.9535) |
| 467 | < 0.0001 (0.6882, 0.0050, 0.9997) | < 0.0001 (1.0000, 1.0000, 0.9444) |
| 468 | 2.7109 (0.5084, 0.0437, 0.9714) | < 0.0001 (1.0000, 1.0000, 0.9900) |
| 469 | 0.3167 (0.1842, 0.0780, 0.7199) | < 0.0001 (1.0000, 1.0000, 0.9924) |
| 470 | < 0.0001 (0.7066, 0.0126, 0.9955) | 0.1444 (0.9924, 0.9917, 0.8791) |
| 471 | < 0.0001 (0.7767, 0.0060, 0.9978) | < 0.0001 (1.0000, 1.0000, 0.9754) |
| 472 | < 0.0001 (1.0000, 0.0073, 0.9961) | < 0.0001 (1.0000, 1.0000, 0.9774) |
| 473 | 1.0000 (0.6667, 1.0000, 0.8456) | < 0.0001 (1.0000, 1.0000, 0.9787) |
| 474 | < 0.0001 (0.2585, 0.0060, 0.9854) | < 0.0001 (1.0000, 1.0000, 0.9774) |
| 475 | < 0.0001 (0.5084, 0.0073, 0.9995) | < 0.0001 (< 0.0001, 1.0000, 0.8196) |
| 476 | < 0.0001 (0.3526, 0.0083, 0.9996) | < 0.0001 (1.0000, 1.0000, 0.9569) |
| 477 | 1.0000 (0.9970, 1.0000, 0.9490) | < 0.0001 (1.0000, 1.0000, 0.9802) |
| 478 | 0.3479 (0.7130, 0.1005, 0.7936) | < 0.0001 (1.0000, 1.0000, 0.9981) |
| 479 | < 0.0001 (< 0.0001, 0.0065, 0.9976) | < 0.0001 (< 0.0001, 1.0000, 0.8964) |
| 480 | < 0.0001 (< 0.0001, 0.0087, 0.9786) | 0.4747 (0.6897, 0.7299, 0.4890) |
| 481 | < 0.0001 (0.4445, 0.0073, 0.9885) | 0.2164 (0.9742, 0.9724, 0.8535) |
| 482 | 1.0000 (0.0052, 1.0000, 0.9552) | 1.0265 (0.4271, 0.4367, 0.4596) |
| 483 | < 0.0001 (0.6667, 0.0095, 0.9981) | < 0.0001 (1.0000, 1.0000, 0.9263) |
| 484 | < 0.0001 (0.4444, 0.0131, 0.9963) | < 0.0001 (1.0000, 1.0000, 0.9675) |
| 485 | < 0.0001 (0.4345, 0.0056, 0.9960) | 0.5000 (0.8889, 0.8889, 0.6959) |
| 486 | < 0.0001 (< 0.0001, 0.0060, 0.9808) | < 0.0001 (1.0000, 1.0000, 0.9541) |
| 487 | < 0.0001 (< 0.0001, 0.0065, 0.9976) | < 0.0001 (< 0.0001, 1.0000, 0.8544) |
| 488 | 0.3013 (0.0241, 0.0674, 0.9995) | < 0.0001 (1.0000, 1.0000, 0.9788) |
| 489 | 1.2294 (< 0.0001, 0.3450, 0.1815) | < 0.0001 (< 0.0001, 1.0000, 0.9064) |
| 490 | < 0.0001 (0.6667, 0.0061, 1.0000) | < 0.0001 (1.0000, 1.0000, 0.9953) |
| 491 | 0.4163 (0.1317, 0.1527, 0.9820) | < 0.0001 (1.0000, 1.0000, 0.9443) |
| 492 | < 0.0001 (0.1975, 0.0142, 0.9830) | < 0.0001 (1.0000, 1.0000, 0.9431) |
| 493 | < 0.0001 (1.0000, 0.0083, 0.9999) | < 0.0001 (1.0000, 1.0000, 0.9904) |
| 494 | 1.0000 (1.0000, 1.0000, 0.9870) | < 0.0001 (1.0000, 1.0000, 0.9590) |
| 495 | 0.6034 (0.4992, 0.2178, 0.9088) | < 0.0001 (1.0000, 1.0000, 0.9227) |
| 496 | < 0.0001 (0.9976, 0.0083, 0.9970) | < 0.0001 (1.0000, 1.0000, 0.9578) |
| 497 | 1.0000 (< 0.0001, 1.0000, 0.9216) | 0.4995 (0.8890, 0.8890, 0.8113) |
| 498 | 0.3013 (0.0023, 0.0674, 0.7951) | < 0.0001 (< 0.0001, 1.0000, 0.8544) |
| 499 | 1.0000 (0.1759, 1.0000, 0.9431) | < 0.0001 (< 0.0001, 1.0000, 0.9479) |
| 500 | < 0.0001 (0.0277, 0.0050, 0.9991) | 0.7407 (0.7709, 0.8204, 0.5361) |
| 501 | < 0.0001 (< 0.0001, 0.0117, 1.0000) | 0.4758 (0.6900, 0.7294, 0.5086) |
| 502 | 0.6495 (0.6944, 0.3211, 0.9442) | < 0.0001 (1.0000, 1.0000, 0.9787) |
| 503 | < 0.0001 (< 0.0001, 0.0083, 0.9949) | 0.8468 (0.5706, 0.5752, 0.4382) |
| 504 | < 0.0001 (0.5084, 0.0099, 0.9932) | < 0.0001 (1.0000, 1.0000, 0.9785) |
| 505 | 1.0000 (0.0390, 1.0000, 0.8339) | < 0.0001 (1.0000, 1.0000, 0.9946) |
| 506 | 1.0000 (0.2202, 1.0000, 0.9404) | < 0.0001 (< 0.0001, 1.0000, 0.8196) |
| 507 | 1.0000 (< 0.0001, 1.0000, 0.9798) | < 0.0001 (< 0.0001, 1.0000, 0.8965) |
| 508 | < 0.0001 (0.6667, 0.0117, 1.0000) | < 0.0001 (< 0.0001, 1.0000, 0.8185) |
| 509 | 1.0000 (0.4359, 1.0000, 0.9870) | < 0.0001 (1.0000, 1.0000, 0.9196) |
| 510 | < 0.0001 (0.1317, 0.0065, 0.9733) | < 0.0001 (1.0000, 1.0000, 0.9582) |
| 511 | < 0.0001 (0.2963, 0.0126, 1.0000) | 0.2500 (0.9630, 0.9630, 0.8292) |
| 512 | < 0.0001 (0.2964, 0.0053, 0.9990) | < 0.0001 (1.0000, 1.0000, 0.9846) |
| 513 | < 0.0001 (0.0469, 0.0126, 0.9997) | < 0.0001 (< 0.0001, 1.0000, 0.9049) |
| 514 | < 0.0001 (0.3349, 0.0093, 0.9869) | < 0.0001 (1.0000, 1.0000, 0.9590) |
| 515 | 1.0000 (0.6667, 1.0000, 0.9798) | < 0.0001 (1.0000, 1.0000, 0.9406) |
| 516 | 0.2786 (< 0.0001, 0.0528, 0.9443) | < 0.0001 (1.0000, 1.0000, 0.9982) |
| 517 | < 0.0001 (0.3526, 0.0037, 0.9880) | 0.4977 (0.6929, 0.6974, 0.5059) |
| 518 | < 0.0001 (0.2583, 0.0061, 0.9808) | 0.1083 (0.9978, 0.9975, 0.9503) |
| 519 | 1.0000 (< 0.0001, 1.0000, 0.9216) | 1.4987 (0.5930, 0.5929, 0.4334) |
| 520 | < 0.0001 (0.2280, 0.0037, 1.0000) | < 0.0001 (< 0.0001, 1.0000, 0.8374) |
| 521 | 0.9073 (0.6667, 0.4320, 0.8633) | 0.3333 (0.9548, 0.9548, 0.7615) |
| 522 | 0.3479 (< 0.0001, 0.1005, 1.0000) | < 0.0001 (1.0000, 1.0000, 0.9920) |
| 523 | < 0.0001 (< 0.0001, 0.0454, 0.9798) | < 0.0001 (< 0.0001, 1.0000, 0.8900) |
| 524 | < 0.0001 (< 0.0001, 0.0041, 0.9905) | < 0.0001 (< 0.0001, 1.0000, 0.7385) |
| 525 | < 0.0001 (0.2964, 0.0053, 0.9958) | < 0.0001 (1.0000, 1.0000, 0.9795) |
| 526 | 1.0000 (0.7130, 1.0000, 0.9342) | < 0.0001 (1.0000, 1.0000, 0.9841) |
| 527 | < 0.0001 (0.0038, 0.0073, 0.9964) | < 0.0001 (1.0000, 1.0000, 0.9927) |
| 528 | < 0.0001 (< 0.0001, 0.0050, 0.9960) | < 0.0001 (< 0.0001, 1.0000, 0.7229) |
| 529 | < 0.0001 (0.0759, 0.0436, 1.0000) | < 0.0001 (1.0000, 1.0000, 0.9915) |
| 530 | 0.2226 (0.7129, 0.0231, 0.9357) | < 0.0001 (1.0000, 1.0000, 0.9926) |
| 531 | < 0.0001 (0.2582, 0.0073, 0.9894) | < 0.0001 (1.0000, 1.0000, 0.9785) |
| 532 | < 0.0001 (0.6667, 0.0117, 0.9963) | < 0.0001 (1.0000, 1.0000, 0.9981) |
| 533 | < 0.0001 (0.9879, 0.0073, 0.9964) | < 0.0001 (< 0.0001, 1.0000, 0.8443) |
| 534 | < 0.0001 (0.7130, 0.0073, 0.9998) | < 0.0001 (1.0000, 1.0000, 0.9787) |
| 535 | < 0.0001 (0.5084, 0.0093, 0.9999) | < 0.0001 (1.0000, 1.0000, 0.9391) |
| 536 | < 0.0001 (0.2963, 0.0099, 0.9984) | < 0.0001 (1.0000, 1.0000, 0.9943) |
| 537 | < 0.0001 (0.0878, 0.0061, 1.0000) | 0.5000 (0.8889, 0.8889, 0.6959) |
| 538 | < 0.0001 (< 0.0001, 0.0050, 0.9998) | < 0.0001 (< 0.0001, 1.0000, 0.7229) |
| 539 | < 0.0001 (0.4776, 0.0060, 0.9904) | < 0.0001 (1.0000, 1.0000, 0.9818) |
| 540 | 1.0000 (0.2584, 1.0000, 0.9230) | < 0.0001 (1.0000, 1.0000, 0.8994) |
| 541 | < 0.0001 (< 0.0001, 0.0107, 0.9988) | < 0.0001 (< 0.0001, 1.0000, 0.8196) |
| 542 | < 0.0001 (0.1314, 0.0083, 0.9987) | < 0.0001 (1.0000, 1.0000, 0.9638) |
| 543 | 0.4404 (0.3527, 0.0899, 0.8328) | < 0.0001 (1.0000, 1.0000, 0.9883) |
| 544 | 1.0000 (0.2963, 1.0000, 0.9480) | < 0.0001 (0.0280, 1.0000, 0.8553) |
| 545 | < 0.0001 (< 0.0001, 0.0397, 0.9991) | < 0.0001 (1.0000, 1.0000, 0.9371) |
| 546 | 1.0000 (< 0.0001, 1.0000, 0.8462) | < 0.0001 (1.0000, 1.0000, 0.9991) |
| 547 | 1.0000 (0.1979, 1.0000, 0.9536) | < 0.0001 (1.0000, 1.0000, 0.9937) |
| 548 | 1.0000 (0.1791, 1.0000, 0.9174) | < 0.0001 (< 0.0001, 1.0000, 0.9144) |
| 549 | < 0.0001 (0.0666, 0.0073, 0.9987) | < 0.0001 (< 0.0001, 1.0000, 0.9049) |
| 550 | < 0.0001 (< 0.0001, 0.0060, 0.9854) | < 0.0001 (1.0000, 1.0000, 0.9780) |
| 551 | < 0.0001 (0.4447, 0.0061, 0.9978) | 0.2500 (0.9630, 0.9630, 0.8046) |
| 552 | < 0.0001 (0.1842, 0.0422, 1.0000) | < 0.0001 (1.0000, 1.0000, 0.9826) |
| 553 | 0.3687 (< 0.0001, 0.1161, 0.9898) | < 0.0001 (< 0.0001, 1.0000, 0.7926) |
| 554 | < 0.0001 (1.0000, 0.0343, 0.9808) | 0.4874 (0.7047, 0.6839, 0.3854) |
| 555 | < 0.0001 (0.9070, 0.0061, 0.9937) | < 0.0001 (< 0.0001, 1.0000, 0.7385) |
| 556 | < 0.0001 (0.2280, 0.0397, 0.9960) | < 0.0001 (1.0000, 1.0000, 0.9827) |
| 557 | < 0.0001 (0.4609, 0.0061, 0.9991) | < 0.0001 (1.0000, 1.0000, 0.9818) |
| 558 | < 0.0001 (0.4444, 0.0060, 0.9854) | < 0.0001 (< 0.0001, 1.0000, 0.9186) |
| 559 | < 0.0001 (0.6944, 0.0061, 0.9960) | < 0.0001 (< 0.0001, 1.0000, 0.8964) |
| 560 | < 0.0001 (0.2964, 0.0060, 0.9978) | < 0.0001 (1.0000, 1.0000, 0.9790) |
| 561 | < 0.0001 (0.7130, 0.0093, 1.0000) | < 0.0001 (< 0.0001, 1.0000, 0.8495) |
| 562 | 0.3482 (0.7130, 0.1007, 0.7935) | < 0.0001 (1.0000, 1.0000, 0.9467) |
| 563 | 0.3358 (< 0.0001, 0.0916, 0.6903) | < 0.0001 (1.0000, 1.0000, 0.9541) |
| 564 | 0.3893 (0.9991, 0.1318, 0.9566) | < 0.0001 (1.0000, 1.0000, 0.9801) |
| 565 | 0.8098 (0.1318, 0.3787, 0.7038) | < 0.0001 (< 0.0001, 1.0000, 0.8038) |
| 566 | < 0.0001 (1.0000, 0.0037, 0.9769) | < 0.0001 (1.0000, 1.0000, 0.9676) |
| 567 | < 0.0001 (0.2582, 0.0053, 0.9990) | < 0.0001 (1.0000, 1.0000, 0.9883) |
| 568 | 0.3014 (< 0.0001, 0.0675, 0.9708) | < 0.0001 (1.0000, 1.0000, 0.9859) |
| 569 | < 0.0001 (0.2964, 0.0083, 0.9997) | < 0.0001 (< 0.0001, 1.0000, 0.7462) |
| 570 | 1.0000 (0.2582, 1.0000, 0.9431) | 0.5132 (0.6535, 0.6608, 0.6239) |
| 571 | < 0.0001 (0.6667, 0.0397, 0.9960) | 0.9983 (0.4461, 0.4459, 0.4283) |
| 572 | 1.0000 (0.0754, 1.0000, 0.9569) | 0.5000 (0.8889, 0.8889, 0.7254) |
| 573 | < 0.0001 (0.9998, 0.0120, 1.0000) | 0.2500 (0.9630, 0.9630, 0.9240) |
| 574 | < 0.0001 (0.1313, 0.0056, 0.9954) | 0.2500 (0.9630, 0.9630, 0.8384) |
| 575 | < 0.0001 (0.1317, 0.0099, 0.9932) | < 0.0001 (1.0000, 1.0000, 0.9844) |
| 576 | < 0.0001 (0.1976, 0.0397, 0.9995) | < 0.0001 (1.0000, 1.0000, 0.9774) |
| 577 | < 0.0001 (< 0.0001, 0.0060, 0.9969) | < 0.0001 (1.0000, 1.0000, 0.9590) |
| 578 | < 0.0001 (0.7130, 0.0087, 0.9926) | < 0.0001 (1.0000, 1.0000, 0.9542) |
| 579 | < 0.0001 (0.6882, 0.0044, 0.9964) | < 0.0001 (1.0000, 1.0000, 0.9176) |
| 580 | 1.0442 (0.0209, 0.4704, 0.9617) | < 0.0001 (< 0.0001, 1.0000, 0.7675) |
| 581 | 1.0000 (0.0724, 1.0000, 0.9431) | < 0.0001 (1.0000, 1.0000, 0.9546) |
| 582 | 1.0000 (< 0.0001, 1.0000, 0.9870) | < 0.0001 (1.0000, 1.0000, 0.9785) |
| 583 | 0.6720 (0.1241, 0.2738, 0.7040) | < 0.0001 (1.0000, 1.0000, 0.9546) |
| 584 | 0.3166 (0.2964, 0.0779, 0.9748) | < 0.0001 (1.0000, 1.0000, 0.9988) |
| 585 | < 0.0001 (0.0390, 0.0397, 0.9991) | < 0.0001 (1.0000, 1.0000, 0.9920) |
| 586 | 0.6033 (0.4359, 0.2907, 0.6163) | < 0.0001 (1.0000, 1.0000, 0.9591) |
| 587 | < 0.0001 (0.3300, 0.0397, 0.9960) | < 0.0001 (< 0.0001, 1.0000, 0.8196) |
| 588 | < 0.0001 (< 0.0001, 0.0060, 0.9989) | < 0.0001 (1.0000, 1.0000, 0.9955) |
| 589 | < 0.0001 (0.6670, 0.0060, 0.9854) | < 0.0001 (< 0.0001, 1.0000, 0.9049) |
| 590 | < 0.0001 (0.0339, 0.0050, 0.9951) | < 0.0001 (1.0000, 1.0000, 0.9386) |
| 591 | 1.0000 (0.9991, 1.0000, 0.8886) | < 0.0001 (< 0.0001, 1.0000, 0.9380) |
| 592 | < 0.0001 (0.5550, 0.0142, 0.9763) | < 0.0001 (1.0000, 1.0000, 0.9926) |
| 593 | < 0.0001 (0.2879, 0.0056, 0.9943) | < 0.0001 (1.0000, 1.0000, 0.9926) |
| 594 | 0.6968 (0.9491, 0.2936, 0.9874) | < 0.0001 (1.0000, 1.0000, 0.9582) |
| 595 | < 0.0001 (< 0.0001, 0.0087, 0.9988) | < 0.0001 (< 0.0001, 1.0000, 0.8495) |
| 596 | 1.0000 (1.0000, 1.0000, 0.9536) | < 0.0001 (1.0000, 1.0000, 0.9444) |
| 597 | < 0.0001 (0.7130, 0.0060, 0.9989) | < 0.0001 (< 0.0001, 1.0000, 0.8443) |
| 598 | < 0.0001 (0.4444, 0.0061, 0.9991) | 0.6120 (0.8125, 0.8559, 0.6683) |
| 599 | < 0.0001 (0.0096, 0.0117, 0.9999) | < 0.0001 (< 0.0001, 1.0000, 0.8152) |
| 600 | 0.3166 (0.0879, 0.0779, 0.7199) | < 0.0001 (1.0000, 1.0000, 0.9936) |
| 601 | 0.3890 (< 0.0001, 0.1316, 0.9992) | < 0.0001 (1.0000, 1.0000, 0.9800) |
| 602 | 1.6023 (0.7130, 0.1912, 0.3911) | 0.4620 (0.9742, 0.9001, 0.8330) |
| 603 | < 0.0001 (0.8889, 0.0056, 0.9884) | 0.4328 (0.9128, 0.9088, 0.6842) |
| 604 | 1.5101 (0.3624, 0.1944, 0.7137) | < 0.0001 (< 0.0001, 1.0000, 0.9008) |
| 605 | < 0.0001 (0.2281, 0.0093, 0.9998) | < 0.0001 (1.0000, 1.0000, 0.9927) |
| 606 | 1.0000 (1.0000, 1.0000, 0.9480) | < 0.0001 (< 0.0001, 1.0000, 0.8544) |
| 607 | < 0.0001 (0.5017, 0.0065, 0.9733) | < 0.0001 (1.0000, 1.0000, 0.9842) |
| 608 | < 0.0001 (0.1317, 0.0050, 0.9951) | < 0.0001 (< 0.0001, 1.0000, 0.8196) |
| 609 | < 0.0001 (0.7130, 0.0056, 0.9997) | < 0.0001 (1.0000, 1.0000, 0.9925) |
| 610 | < 0.0001 (0.4444, 0.0099, 0.9999) | < 0.0001 (1.0000, 1.0000, 0.9263) |
| 611 | 1.0000 (0.7130, 1.0000, 0.8462) | < 0.0001 (1.0000, 1.0000, 0.9467) |
| 612 | < 0.0001 (< 0.0001, 0.0050, 0.9999) | < 0.0001 (1.0000, 1.0000, 0.9892) |
| 613 | < 0.0001 (0.2280, 0.0083, 0.9996) | 0.4758 (0.6900, 0.7294, 0.5086) |
| 614 | < 0.0001 (0.6667, 0.0083, 0.9948) | < 0.0001 (< 0.0001, 1.0000, 0.8095) |
| 615 | < 0.0001 (0.5084, 0.0397, 0.9999) | < 0.0001 (1.0000, 1.0000, 0.9999) |
| 616 | 1.0000 (0.3527, 1.0000, 0.9351) | 0.4996 (0.8890, 0.8890, 0.8192) |
| 617 | < 0.0001 (0.4444, 0.0397, 0.9960) | < 0.0001 (1.0000, 1.0000, 0.9860) |
| 618 | < 0.0001 (0.1841, 0.0343, 0.9817) | < 0.0001 (1.0000, 1.0000, 0.9612) |
| 619 | < 0.0001 (0.1552, 0.0142, 1.0000) | < 0.0001 (1.0000, 1.0000, 0.9967) |
| 620 | 0.3015 (0.5084, 0.0676, 0.9334) | < 0.0001 (1.0000, 1.0000, 0.9599) |
| 621 | < 0.0001 (0.8002, 0.0056, 0.9876) | < 0.0001 (< 0.0001, 1.0000, 0.8196) |
| 622 | 0.9062 (0.6667, 0.4312, 0.9448) | < 0.0001 (< 0.0001, 1.0000, 0.9004) |
| 623 | < 0.0001 (0.3624, 0.0065, 0.9858) | < 0.0001 (< 0.0001, 1.0000, 0.8152) |
| 624 | 1.0000 (0.6807, 1.0000, 0.9216) | < 0.0001 (1.0000, 1.0000, 0.9836) |
| 625 | < 0.0001 (0.0586, 0.0093, 0.9869) | < 0.0001 (1.0000, 1.0000, 0.9825) |
| 626 | < 0.0001 (0.6667, 0.0087, 1.0000) | < 0.0001 (1.0000, 1.0000, 0.9569) |
| 627 | < 0.0001 (0.1975, 0.0060, 0.9896) | < 0.0001 (1.0000, 1.0000, 0.9406) |
| 628 | < 0.0001 (0.7130, 0.0083, 0.9921) | < 0.0001 (1.0000, 1.0000, 0.9630) |
| 629 | < 0.0001 (0.0358, 0.0093, 1.0000) | < 0.0001 (< 0.0001, 1.0000, 0.8196) |
| 630 | < 0.0001 (< 0.0001, 0.0060, 0.9999) | < 0.0001 (1.0000, 1.0000, 0.9622) |
| 631 | 1.0000 (0.4444, 1.0000, 0.9611) | < 0.0001 (1.0000, 1.0000, 0.9795) |
| 632 | < 0.0001 (0.1317, 0.0131, 0.9963) | < 0.0001 (< 0.0001, 1.0000, 0.7527) |
| 633 | < 0.0001 (0.1321, 0.0083, 0.9948) | < 0.0001 (< 0.0001, 1.0000, 0.9008) |
| 634 | 0.3187 (0.9994, 0.0794, 0.9684) | < 0.0001 (1.0000, 1.0000, 0.9165) |
| 635 | < 0.0001 (< 0.0001, 0.0131, 1.0000) | < 0.0001 (1.0000, 1.0000, 0.9859) |
| 636 | 1.0000 (0.0601, 1.0000, 0.8401) | < 0.0001 (1.0000, 1.0000, 0.9446) |
| 637 | 0.3066 (< 0.0001, 0.0710, 0.5189) | < 0.0001 (1.0000, 1.0000, 0.9827) |
| 638 | 0.3245 (0.5083, 0.0835, 0.7772) | 0.5000 (0.8889, 0.8889, 0.6676) |
| 639 | < 0.0001 (< 0.0001, 0.0425, 0.9984) | < 0.0001 (< 0.0001, 1.0000, 0.8196) |
| 640 | < 0.0001 (0.1977, 0.0050, 0.9991) | < 0.0001 (1.0000, 1.0000, 0.9515) |
| 641 | 1.0000 (0.1981, 1.0000, 0.9552) | < 0.0001 (1.0000, 1.0000, 0.9780) |
| 642 | 0.3478 (< 0.0001, 0.1005, 0.7937) | < 0.0001 (1.0000, 1.0000, 0.9993) |
| 643 | < 0.0001 (1.0000, 0.0056, 0.9944) | 0.5000 (0.9630, 0.8889, 0.8506) |
| 644 | 1.0000 (< 0.0001, 1.0000, 0.9342) | < 0.0001 (1.0000, 1.0000, 0.9945) |
| 645 | < 0.0001 (0.4444, 0.0397, 0.9991) | < 0.0001 (< 0.0001, 1.0000, 0.7462) |
| 646 | < 0.0001 (< 0.0001, 0.0083, 0.9949) | < 0.0001 (1.0000, 1.0000, 0.9515) |
| 647 | < 0.0001 (0.0492, 0.0050, 0.9980) | < 0.0001 (< 0.0001, 1.0000, 0.8544) |
| 648 | < 0.0001 (0.0876, 0.0083, 0.9841) | < 0.0001 (1.0000, 1.0000, 0.9408) |
| 649 | < 0.0001 (1.0000, 0.0053, 0.9905) | < 0.0001 (1.0000, 1.0000, 0.9541) |
| 650 | < 0.0001 (< 0.0001, 0.0093, 0.9999) | < 0.0001 (< 0.0001, 1.0000, 0.7648) |
| 651 | < 0.0001 (1.0000, 0.0117, 0.9999) | < 0.0001 (< 0.0001, 1.0000, 0.8176) |
| 652 | < 0.0001 (0.9978, 0.0083, 0.9772) | < 0.0001 (1.0000, 1.0000, 0.9780) |
| 653 | 0.4046 (0.4891, 0.1436, 0.8214) | < 0.0001 (1.0000, 1.0000, 0.9955) |
| 654 | 1.0445 (0.3624, 0.4702, 0.4545) | < 0.0001 (< 0.0001, 1.0000, 0.8196) |
| 655 | 0.3188 (0.9998, 0.0794, 0.9525) | < 0.0001 (1.0000, 1.0000, 0.9767) |
| 656 | 0.3013 (< 0.0001, 0.0674, 0.9985) | < 0.0001 (1.0000, 1.0000, 0.9770) |
| 657 | 0.6377 (0.6667, 0.2459, 0.9779) | < 0.0001 (1.0000, 1.0000, 0.9957) |
| 658 | 0.3065 (0.0843, 0.0710, 0.9911) | < 0.0001 (1.0000, 1.0000, 0.9813) |
| 659 | < 0.0001 (0.5084, 0.0061, 0.9998) | 0.5135 (0.6531, 0.6605, 0.6239) |
| 660 | < 0.0001 (0.1842, 0.0060, 0.9904) | < 0.0001 (1.0000, 1.0000, 0.9176) |
| 661 | < 0.0001 (0.6667, 0.0083, 0.9949) | < 0.0001 (1.0000, 1.0000, 0.9993) |
| 662 | < 0.0001 (0.6882, 0.0425, 0.9944) | < 0.0001 (< 0.0001, 1.0000, 0.8196) |
| 663 | 1.0000 (0.7130, 1.0000, 0.8516) | < 0.0001 (1.0000, 1.0000, 0.9583) |
| 664 | < 0.0001 (0.6667, 0.0073, 0.9994) | < 0.0001 (< 0.0001, 1.0000, 0.9479) |
| 665 | < 0.0001 (< 0.0001, 0.0397, 0.9991) | < 0.0001 (1.0000, 1.0000, 0.9890) |
| 666 | < 0.0001 (0.1317, 0.0073, 0.9961) | < 0.0001 (< 0.0001, 1.0000, 0.9008) |
| 667 | < 0.0001 (0.5082, 0.0093, 1.0000) | 0.3607 (0.9697, 0.9242, 0.6999) |
| 668 | < 0.0001 (0.1318, 0.0093, 0.9999) | < 0.0001 (< 0.0001, 1.0000, 0.9064) |
| 669 | < 0.0001 (0.1318, 0.0117, 0.9873) | < 0.0001 (1.0000, 1.0000, 0.9410) |
| 670 | 1.0000 (0.4736, 1.0000, 0.9216) | < 0.0001 (< 0.0001, 1.0000, 0.8196) |
| 671 | < 0.0001 (< 0.0001, 0.0065, 0.9996) | < 0.0001 (1.0000, 1.0000, 0.9467) |
| 672 | 1.0000 (0.3624, 1.0000, 0.9536) | < 0.0001 (1.0000, 1.0000, 0.9657) |
| 673 | < 0.0001 (0.7130, 0.0142, 0.9943) | < 0.0001 (1.0000, 1.0000, 0.9515) |
| 674 | 1.0000 (1.0000, 1.0000, 0.9816) | < 0.0001 (1.0000, 1.0000, 0.9405) |
| 675 | < 0.0001 (< 0.0001, 0.0083, 0.9999) | < 0.0001 (1.0000, 1.0000, 0.9968) |
| 676 | < 0.0001 (0.6667, 0.0083, 0.9999) | < 0.0001 (1.0000, 1.0000, 0.9919) |
| 677 | < 0.0001 (0.9751, 0.0056, 0.9943) | < 0.0001 (1.0000, 1.0000, 0.9467) |
| 678 | 1.0000 (0.2275, 1.0000, 0.9342) | < 0.0001 (< 0.0001, 1.0000, 0.9008) |
| 679 | < 0.0001 (0.2202, 0.0060, 0.9854) | < 0.0001 (< 0.0001, 1.0000, 0.9479) |
| 680 | 1.0000 (0.9995, 1.0000, 0.9351) | < 0.0001 (< 0.0001, 1.0000, 0.7229) |
| 681 | 0.6030 (0.6882, 0.2905, 0.8111) | 0.3303 (0.9865, 0.9557, 0.8809) |
| 682 | 1.0000 (0.4445, 1.0000, 0.9230) | < 0.0001 (1.0000, 1.0000, 0.9596) |
| 683 | < 0.0001 (0.1977, 0.0060, 0.9999) | < 0.0001 (1.0000, 1.0000, 0.9901) |
| 684 | < 0.0001 (0.2201, 0.0053, 0.9991) | < 0.0001 (< 0.0001, 1.0000, 0.7401) |
| 685 | 0.3686 (0.1980, 0.1160, 0.9929) | < 0.0001 (< 0.0001, 1.0000, 0.6889) |
| 686 | 0.3686 (0.5084, 0.1160, 0.8283) | 0.4805 (0.9000, 0.8947, 0.7266) |
| 687 | 1.0000 (0.9932, 1.0000, 0.9798) | < 0.0001 (1.0000, 1.0000, 0.9956) |
| 688 | < 0.0001 (0.6944, 0.0099, 0.9999) | < 0.0001 (< 0.0001, 1.0000, 0.9380) |
| 689 | 1.0000 (< 0.0001, 1.0000, 0.9431) | < 0.0001 (1.0000, 1.0000, 0.9774) |
| 690 | 0.3778 (0.2492, 0.1230, 0.9528) | 1.4997 (0.2966, 0.2964, 0.2624) |
| 691 | < 0.0001 (0.2963, 0.0060, 0.9854) | < 0.0001 (< 0.0001, 1.0000, 0.9064) |
| 692 | 0.2787 (< 0.0001, 0.0528, 0.9997) | 0.4328 (0.9128, 0.9088, 0.7405) |
| 693 | < 0.0001 (0.7130, 0.0037, 0.9963) | < 0.0001 (< 0.0001, 1.0000, 0.8469) |
| 694 | < 0.0001 (0.6667, 0.0060, 0.9808) | < 0.0001 (1.0000, 1.0000, 0.9770) |
| 695 | < 0.0001 (0.0361, 0.0056, 0.9991) | < 0.0001 (1.0000, 1.0000, 0.9756) |
| 696 | 0.3166 (< 0.0001, 0.0779, 0.7055) | < 0.0001 (< 0.0001, 1.0000, 0.8196) |
| 697 | < 0.0001 (0.5083, 0.0061, 0.9867) | < 0.0001 (< 0.0001, 1.0000, 0.8196) |
| 698 | < 0.0001 (0.4776, 0.0117, 1.0000) | 0.9997 (0.7409, 0.7408, 0.4806) |
| 699 | 0.3013 (0.0023, 0.0674, 0.9831) | < 0.0001 (1.0000, 1.0000, 0.9957) |
| 700 | < 0.0001 (0.7408, 0.0050, 0.9998) | < 0.0001 (1.0000, 1.0000, 1.0000) |
| 701 | < 0.0001 (0.0586, 0.0050, 0.9874) | < 0.0001 (< 0.0001, 1.0000, 0.8396) |
| 702 | < 0.0001 (< 0.0001, 0.0126, 0.9997) | < 0.0001 (< 0.0001, 1.0000, 0.8469) |
| 703 | 0.3166 (0.0195, 0.0779, 0.8901) | < 0.0001 (1.0000, 1.0000, 0.9890) |
| 704 | < 0.0001 (< 0.0001, 0.0060, 0.9854) | 0.5000 (0.8889, 0.8889, 0.8113) |
| 705 | < 0.0001 (0.1313, 0.0061, 0.9958) | < 0.0001 (< 0.0001, 1.0000, 0.7546) |
| 706 | 1.0000 (< 0.0001, 1.0000, 0.9230) | < 0.0001 (1.0000, 1.0000, 0.9926) |
| 707 | < 0.0001 (1.0000, 0.0044, 0.9964) | 0.2898 (0.9710, 0.9681, 0.7506) |
| 708 | < 0.0001 (0.7129, 0.0425, 0.9984) | < 0.0001 (1.0000, 1.0000, 0.9405) |
| 709 | < 0.0001 (0.7130, 0.0061, 0.9998) | < 0.0001 (< 0.0001, 1.0000, 0.6874) |
| 710 | < 0.0001 (0.6667, 0.0061, 0.9937) | < 0.0001 (1.0000, 1.0000, 0.9808) |
| 711 | < 0.0001 (0.6882, 0.0425, 0.9984) | < 0.0001 (1.0000, 1.0000, 0.9747) |
| 712 | 1.0000 (0.3816, 1.0000, 0.9216) | < 0.0001 (1.0000, 1.0000, 0.9638) |
| 713 | < 0.0001 (0.1975, 0.0065, 0.9952) | < 0.0001 (1.0000, 1.0000, 0.9686) |
| 714 | < 0.0001 (0.2964, 0.0050, 0.9951) | < 0.0001 (< 0.0001, 1.0000, 0.9113) |
| 715 | < 0.0001 (0.7408, 0.0061, 0.9991) | 0.2320 (0.9977, 0.9682, 0.8901) |
| 716 | 1.0000 (0.2963, 1.0000, 0.9798) | < 0.0001 (1.0000, 1.0000, 0.9921) |
| 717 | < 0.0001 (0.0023, 0.0065, 0.9733) | 0.4644 (0.9119, 0.8994, 0.6074) |
| 718 | < 0.0001 (< 0.0001, 0.0117, 0.9999) | < 0.0001 (< 0.0001, 1.0000, 0.8549) |
| 719 | < 0.0001 (0.6668, 0.0060, 0.9991) | < 0.0001 (< 0.0001, 1.0000, 0.7592) |
| 720 | < 0.0001 (< 0.0001, 0.0131, 0.9963) | < 0.0001 (1.0000, 1.0000, 0.9356) |
| 721 | 2.8097 (< 0.0001, 0.0064, 0.0854) | < 0.0001 (1.0000, 1.0000, 0.9694) |
| 722 | < 0.0001 (0.6744, 0.0060, 0.9854) | < 0.0001 (1.0000, 1.0000, 0.9358) |
| 723 | < 0.0001 (0.3527, 0.0056, 0.9960) | < 0.0001 (1.0000, 1.0000, 0.9639) |
| 724 | < 0.0001 (0.1890, 0.0083, 0.9999) | < 0.0001 (1.0000, 1.0000, 0.9301) |
| 725 | < 0.0001 (< 0.0001, 0.0117, 1.0000) | < 0.0001 (< 0.0001, 1.0000, 0.6746) |
| 726 | 0.3015 (0.0936, 0.0675, 0.9915) | < 0.0001 (< 0.0001, 1.0000, 0.7949) |
| 727 | < 0.0001 (< 0.0001, 0.0131, 0.9992) | < 0.0001 (1.0000, 1.0000, 0.9621) |
| 728 | < 0.0001 (0.0879, 0.0083, 0.9970) | < 0.0001 (1.0000, 1.0000, 0.9995) |
| 729 | < 0.0001 (1.0000, 0.0056, 0.9884) | < 0.0001 (< 0.0001, 1.0000, 0.9113) |
| 730 | 1.0000 (0.6667, 1.0000, 0.9431) | 0.9763 (0.4947, 0.4663, 0.2686) |
| 731 | < 0.0001 (0.6667, 0.0061, 0.9978) | < 0.0001 (1.0000, 1.0000, 0.8732) |
| 732 | 0.3480 (< 0.0001, 0.1006, 0.9724) | < 0.0001 (1.0000, 1.0000, 0.9890) |
| 733 | 1.0000 (0.1843, 1.0000, 0.8540) | < 0.0001 (1.0000, 1.0000, 0.9621) |
| 734 | < 0.0001 (0.9956, 0.0343, 0.9808) | < 0.0001 (1.0000, 1.0000, 0.9744) |
| 735 | < 0.0001 (< 0.0001, 0.0083, 0.9948) | < 0.0001 (1.0000, 1.0000, 0.9160) |
| 736 | < 0.0001 (0.5084, 0.0117, 1.0000) | < 0.0001 (< 0.0001, 1.0000, 0.8854) |
| 737 | 1.0000 (0.0391, 1.0000, 0.9216) | < 0.0001 (< 0.0001, 1.0000, 0.9195) |
| 738 | < 0.0001 (0.2963, 0.0060, 0.9947) | < 0.0001 (1.0000, 1.0000, 0.8828) |
| 739 | < 0.0001 (0.0079, 0.0061, 0.9988) | < 0.0001 (1.0000, 1.0000, 0.9856) |
| 740 | < 0.0001 (0.6667, 0.0117, 1.0000) | < 0.0001 (1.0000, 1.0000, 0.9821) |
| 741 | < 0.0001 (< 0.0001, 0.0085, 0.9945) | 0.4927 (0.4663, 0.6941, 0.3233) |
| 742 | 0.4045 (0.5927, 0.1436, 0.9886) | 0.4542 (0.9708, 0.9025, 0.7816) |
| 743 | < 0.0001 (0.6944, 0.0425, 0.9986) | 0.9510 (0.6378, 0.7554, 0.3462) |
| 744 | < 0.0001 (0.6667, 0.0117, 1.0000) | < 0.0001 (< 0.0001, 1.0000, 0.7698) |
| 745 | 1.0000 (0.2273, 1.0000, 0.9216) | < 0.0001 (1.0000, 1.0000, 0.9898) |
| 746 | < 0.0001 (0.0073, 0.0056, 0.9991) | < 0.0001 (1.0000, 1.0000, 0.9644) |
| 747 | 1.0000 (< 0.0001, 1.0000, 0.9870) | < 0.0001 (1.0000, 1.0000, 0.9755) |
| 748 | < 0.0001 (0.8563, 0.0107, 0.9993) | < 0.0001 (< 0.0001, 1.0000, 0.7592) |
| 749 | 1.0000 (0.0408, 1.0000, 0.9798) | 0.5002 (0.8889, 0.8888, 0.5982) |
| 750 | 0.4414 (0.4992, 0.0906, 0.8314) | < 0.0001 (1.0000, 1.0000, 0.9951) |
| 751 | < 0.0001 (0.5084, 0.0050, 0.9960) | < 0.0001 (0.0303, 1.0000, 0.9843) |
| 752 | < 0.0001 (0.6784, 0.0343, 0.9808) | < 0.0001 (< 0.0001, 1.0000, 0.8096) |
| 753 | < 0.0001 (0.0667, 0.0060, 0.9216) | < 0.0001 (1.0000, 1.0000, 0.9987) |
| 754 | < 0.0001 (0.6667, 0.0397, 0.9982) | < 0.0001 (1.0000, 1.0000, 0.9901) |
| 755 | < 0.0001 (0.8344, 0.0425, 0.9986) | < 0.0001 (< 0.0001, 1.0000, 0.7682) |
| 756 | < 0.0001 (< 0.0001, 0.0056, 0.9960) | 0.9684 (0.4676, 0.4989, 0.3117) |
| 757 | 0.3186 (0.0878, 0.0793, 0.9684) | < 0.0001 (1.0000, 1.0000, 0.9160) |
| 758 | < 0.0001 (0.6988, 0.0126, 1.0000) | 0.4344 (0.8413, 0.9141, 0.4764) |
| 759 | < 0.0001 (0.9997, 0.0424, 0.9996) | < 0.0001 (< 0.0001, 1.0000, 0.8096) |
| 760 | < 0.0001 (0.9877, 0.0397, 0.9739) | < 0.0001 (< 0.0001, 1.0000, 0.7996) |
| 761 | 1.0000 (0.9096, 1.0000, 0.9798) | < 0.0001 (< 0.0001, 1.0000, 0.7949) |
| 762 | 1.0000 (0.9939, 1.0000, 0.9870) | < 0.0001 (1.0000, 1.0000, 0.9863) |
| 763 | < 0.0001 (0.7130, 0.0056, 0.9696) | < 0.0001 (1.0000, 1.0000, 0.9285) |
| 764 | 0.9522 (0.6668, 0.4659, 0.9671) | < 0.0001 (1.0000, 1.0000, 0.9879) |
| 765 | 0.3065 (0.0667, 0.0709, 0.9207) | < 0.0001 (1.0000, 1.0000, 0.9675) |
| 766 | < 0.0001 (0.2968, 0.0061, 0.9991) | < 0.0001 (1.0000, 1.0000, 0.8996) |
| 767 | < 0.0001 (0.1758, 0.0085, 0.9759) | < 0.0001 (< 0.0001, 1.0000, 0.8819) |
| 768 | < 0.0001 (0.4359, 0.0065, 0.9856) | < 0.0001 (1.0000, 1.0000, 0.9597) |
| 769 | 0.5736 (0.4445, 0.1936, 0.7396) | < 0.0001 (1.0000, 1.0000, 0.9811) |
| 770 | < 0.0001 (1.0000, 0.0107, 1.0000) | < 0.0001 (1.0000, 1.0000, 0.9357) |
| 771 | 1.0000 (< 0.0001, 1.0000, 0.9816) | < 0.0001 (1.0000, 1.0000, 0.9442) |
| 772 | < 0.0001 (< 0.0001, 0.0061, 0.9978) | < 0.0001 (< 0.0001, 1.0000, 0.8081) |
| 773 | 1.1990 (0.2963, 0.3802, 0.3350) | < 0.0001 (1.0000, 1.0000, 0.9185) |
| 774 | 0.3013 (0.2968, 0.0674, 0.8205) | < 0.0001 (< 0.0001, 1.0000, 0.7592) |
| 775 | 0.3890 (1.0000, 0.1316, 0.8826) | < 0.0001 (< 0.0001, 1.0000, 0.9195) |
| 776 | 1.0000 (0.0830, 1.0000, 0.8956) | < 0.0001 (< 0.0001, 1.0000, 0.8854) |
| 777 | < 0.0001 (0.6667, 0.0061, 0.9948) | < 0.0001 (< 0.0001, 1.0000, 0.7996) |
| 778 | 1.3949 (0.3625, 0.2644, 0.5637) | 0.5001 (0.8888, 0.8889, 0.7991) |
| 779 | 1.0000 (0.6667, 1.0000, 0.9216) | < 0.0001 (1.0000, 1.0000, 0.9621) |
| 780 | 1.0000 (0.4444, 1.0000, 0.9216) | < 0.0001 (< 0.0001, 1.0000, 0.8096) |
| 781 | 0.6962 (0.4736, 0.2931, 0.8704) | < 0.0001 (1.0000, 1.0000, 0.8828) |
| 782 | < 0.0001 (0.0077, 0.0424, 0.9998) | < 0.0001 (1.0000, 1.0000, 0.9357) |
| 783 | < 0.0001 (0.7130, 0.0126, 1.0000) | < 0.0001 (1.0000, 1.0000, 0.8873) |
| 784 | < 0.0001 (< 0.0001, 0.0117, 0.9910) | < 0.0001 (1.0000, 1.0000, 0.9403) |
| 785 | 1.0000 (0.0668, 1.0000, 0.8956) | < 0.0001 (1.0000, 1.0000, 0.9168) |
| 786 | < 0.0001 (0.0321, 0.0107, 0.9993) | 0.5000 (0.8889, 0.8889, 0.7314) |
| 787 | 0.3480 (< 0.0001, 0.1006, 0.9439) | < 0.0001 (1.0000, 1.0000, 0.9507) |
| 788 | < 0.0001 (0.0490, 0.0053, 0.9753) | 0.9854 (0.4663, 0.4818, 0.3234) |
| 789 | < 0.0001 (< 0.0001, 0.0083, 0.9996) | < 0.0001 (1.0000, 1.0000, 0.9744) |
| 790 | 0.7384 (< 0.0001, 0.3259, 0.9999) | < 0.0001 (< 0.0001, 1.0000, 0.6618) |
| 791 | < 0.0001 (0.4451, 0.0061, 0.9807) | < 0.0001 (1.0000, 1.0000, 0.9610) |
| 792 | 1.0000 (0.2682, 1.0000, 0.8612) | < 0.0001 (1.0000, 1.0000, 0.9621) |
| 793 | < 0.0001 (0.0174, 0.0050, 0.9999) | < 0.0001 (< 0.0001, 1.0000, 0.6874) |
| 794 | < 0.0001 (0.6667, 0.0056, 1.0000) | < 0.0001 (1.0000, 1.0000, 0.9413) |
| 795 | < 0.0001 (0.3300, 0.0087, 0.9926) | < 0.0001 (1.0000, 1.0000, 0.9404) |
| 796 | < 0.0001 (< 0.0001, 0.0061, 0.9960) | < 0.0001 (< 0.0001, 1.0000, 0.7515) |
| 797 | 1.0000 (0.2582, 1.0000, 0.9816) | < 0.0001 (1.0000, 1.0000, 0.9621) |
| 798 | 0.3244 (0.1318, 0.0835, 0.9891) | < 0.0001 (1.0000, 1.0000, 0.9597) |
| 799 | 0.3170 (0.6603, 0.0782, 0.9969) | < 0.0001 (1.0000, 1.0000, 0.9356) |
| 800 | 0.8369 (0.1317, 0.3754, 0.3812) | < 0.0001 (1.0000, 1.0000, 0.9718) |
| 801 | 1.0000 (0.6505, 1.0000, 0.9436) | < 0.0001 (< 0.0001, 1.0000, 0.7682) |
| 802 | < 0.0001 (0.5084, 0.0343, 0.9174) | < 0.0001 (1.0000, 1.0000, 0.9744) |
| 803 | < 0.0001 (< 0.0001, 0.0073, 0.9987) | < 0.0001 (1.0000, 1.0000, 0.9302) |
| 804 | < 0.0001 (< 0.0001, 0.0425, 0.9973) | 0.3334 (0.9997, 0.9997, 0.7299) |
| 805 | < 0.0001 (0.7130, 0.0044, 0.9891) | < 0.0001 (1.0000, 1.0000, 0.9226) |
| 806 | < 0.0001 (0.5084, 0.0087, 0.9988) | 0.4927 (0.6829, 0.6942, 0.4972) |
| 807 | < 0.0001 (0.2326, 0.0060, 0.9854) | 0.2562 (0.9586, 0.9611, 0.9343) |
| 808 | 0.2786 (0.6603, 0.0528, 0.8724) | < 0.0001 (1.0000, 1.0000, 0.9605) |
| 809 | < 0.0001 (0.1975, 0.0060, 0.9854) | < 0.0001 (1.0000, 1.0000, 0.9898) |
| 810 | < 0.0001 (< 0.0001, 0.0083, 0.9948) | 0.4844 (0.6837, 0.7063, 0.4840) |
| 811 | < 0.0001 (0.1313, 0.0117, 1.0000) | < 0.0001 (1.0000, 1.0000, 0.9274) |
| 812 | 1.0000 (0.8889, 1.0000, 0.9816) | 0.1676 (0.9958, 0.9875, 0.9629) |
| 813 | 1.1072 (0.2578, 0.4312, 0.7886) | 0.2500 (0.9877, 0.9630, 0.8914) |
| 814 | < 0.0001 (0.9250, 0.0053, 0.9870) | 0.1667 (0.9877, 0.9877, 0.8904) |
| 815 | < 0.0001 (< 0.0001, 0.0065, 0.9981) | 0.5000 (0.6667, 0.6667, 0.4310) |
| 816 | < 0.0001 (0.2326, 0.0083, 0.9987) | 0.2496 (0.9631, 0.9631, 0.9185) |
| 817 | < 0.0001 (< 0.0001, 0.0073, 0.9964) | 1.2551 (0.4952, 0.6606, 0.2021) |
| 818 | < 0.0001 (0.5416, 0.0061, 0.9937) | < 0.0001 (1.0000, 1.0000, 0.9718) |
| 819 | 1.0000 (0.6603, 1.0000, 0.9798) | 0.8664 (0.3781, 0.3905, 0.3615) |
| 820 | 1.0000 (0.6667, 1.0000, 0.9870) | < 0.0001 (1.0000, 1.0000, 0.9317) |
| 821 | < 0.0001 (0.1759, 0.0065, 0.9952) | < 0.0001 (1.0000, 1.0000, 0.9180) |
| 822 | < 0.0001 (0.4445, 0.0095, 0.9994) | < 0.0001 (1.0000, 1.0000, 0.8855) |
| 823 | < 0.0001 (0.6667, 0.0142, 1.0000) | < 0.0001 (< 0.0001, 1.0000, 0.8854) |
| 824 | 0.9515 (0.4823, 0.4655, 0.3280) | < 0.0001 (1.0000, 1.0000, 0.9364) |
| 825 | 0.4164 (0.4444, 0.1528, 0.9062) | < 0.0001 (1.0000, 1.0000, 0.9924) |
| 826 | 2.0858 (0.6603, 0.0717, 0.2259) | < 0.0001 (< 0.0001, 1.0000, 0.8854) |
| 827 | 1.0000 (0.1979, 1.0000, 0.8619) | < 0.0001 (1.0000, 1.0000, 0.9301) |
| 828 | < 0.0001 (0.5083, 0.0073, 0.9985) | < 0.0001 (1.0000, 1.0000, 0.9748) |
| 829 | < 0.0001 (< 0.0001, 0.0050, 0.9960) | 0.1450 (0.9993, 0.9916, 0.9333) |
| 830 | < 0.0001 (0.2963, 0.0093, 0.9993) | < 0.0001 (1.0000, 1.0000, 0.9180) |
| 831 | 1.0000 (0.5084, 1.0000, 0.9174) | 0.7704 (0.8413, 0.7770, 0.8318) |
| 832 | 0.6214 (0.0390, 0.2327, 0.8275) | < 0.0001 (1.0000, 1.0000, 0.9464) |
| 833 | < 0.0001 (0.4444, 0.0425, 0.9998) | < 0.0001 (< 0.0001, 1.0000, 0.8523) |
| 834 | < 0.0001 (0.0023, 0.0061, 1.0000) | < 0.0001 (< 0.0001, 1.0000, 0.8858) |
| 835 | 1.0000 (0.2818, 1.0000, 0.9431) | < 0.0001 (1.0000, 1.0000, 0.9898) |
| 836 | < 0.0001 (0.5599, 0.0060, 1.0000) | < 0.0001 (1.0000, 1.0000, 0.9984) |
| 837 | < 0.0001 (0.1842, 0.0060, 1.0000) | < 0.0001 (1.0000, 1.0000, 0.9288) |
| 838 | 0.6030 (1.0000, 0.2905, 0.6165) | < 0.0001 (1.0000, 1.0000, 0.8838) |
| 839 | 0.6338 (0.9805, 0.2428, 0.4854) | 0.4640 (0.9120, 0.8996, 0.5931) |
| 840 | < 0.0001 (0.7776, 0.0061, 0.9991) | < 0.0001 (1.0000, 1.0000, 0.9294) |
| 841 | 1.1073 (0.1576, 0.4312, 0.9111) | < 0.0001 (< 0.0001, 1.0000, 0.7794) |
| 842 | 0.6335 (0.5084, 0.2425, 0.5016) | < 0.0001 (< 0.0001, 1.0000, 0.8584) |
| 843 | 1.0000 (0.9994, 1.0000, 0.9351) | 0.4999 (0.8889, 0.8889, 0.6521) |
| 844 | 1.0000 (1.0000, 1.0000, 0.9216) | < 0.0001 (< 0.0001, 1.0000, 0.6603) |
| 845 | < 0.0001 (< 0.0001, 0.0083, 0.9987) | < 0.0001 (1.0000, 1.0000, 0.9315) |
| 846 | < 0.0001 (0.6667, 0.0087, 0.9982) | 1.0029 (0.7400, 0.7399, 0.7407) |
| 847 | < 0.0001 (1.0000, 0.0056, 0.9974) | < 0.0001 (< 0.0001, 1.0000, 0.8081) |
| 848 | < 0.0001 (< 0.0001, 0.0425, 0.9991) | < 0.0001 (1.0000, 1.0000, 0.9686) |
| 849 | < 0.0001 (0.6950, 0.0313, 0.9929) | 1.9699 (0.2177, 0.2320, 0.0973) |
| 850 | 1.0000 (0.0814, 1.0000, 0.9536) | < 0.0001 (< 0.0001, 1.0000, 0.7996) |
| 851 | < 0.0001 (0.0096, 0.0083, 0.9988) | < 0.0001 (1.0000, 1.0000, 0.9198) |
| 852 | < 0.0001 (0.0338, 0.0107, 0.9999) | 0.5000 (0.6667, 0.6667, 0.6590) |
| 853 | 0.2922 (< 0.0001, 0.0615, 0.8864) | < 0.0001 (1.0000, 1.0000, 0.9166) |
| 854 | < 0.0001 (0.4992, 0.0425, 0.9993) | 0.8656 (0.5539, 0.5637, 0.4336) |
| 855 | < 0.0001 (0.6667, 0.0073, 0.9998) | 0.4343 (0.9120, 0.9083, 0.5712) |
| 856 | < 0.0001 (0.6784, 0.0099, 0.9798) | < 0.0001 (1.0000, 1.0000, 0.9841) |
| 857 | < 0.0001 (0.0877, 0.0343, 0.9921) | < 0.0001 (< 0.0001, 1.0000, 0.7212) |
| 858 | 1.0000 (0.0175, 1.0000, 0.9870) | < 0.0001 (< 0.0001, 1.0000, 0.8854) |
| 859 | 1.0000 (0.2584, 1.0000, 0.9798) | < 0.0001 (1.0000, 1.0000, 0.9344) |
| 860 | 1.0000 (0.4445, 1.0000, 0.9351) | < 0.0001 (1.0000, 1.0000, 0.9931) |
| 861 | 0.3188 (< 0.0001, 0.0795, 0.9817) | < 0.0001 (1.0000, 1.0000, 0.9474) |
| 862 | < 0.0001 (0.2964, 0.0083, 0.9999) | < 0.0001 (< 0.0001, 1.0000, 0.7996) |
| 863 | < 0.0001 (0.9996, 0.0060, 0.9905) | 0.4978 (0.9633, 0.8895, 0.9139) |
| 864 | < 0.0001 (0.2635, 0.0065, 0.9894) | < 0.0001 (1.0000, 1.0000, 0.9918) |
| 865 | < 0.0001 (0.2878, 0.0073, 0.9964) | < 0.0001 (1.0000, 1.0000, 0.9624) |
| 866 | < 0.0001 (< 0.0001, 0.0424, 0.9872) | < 0.0001 (< 0.0001, 1.0000, 0.7794) |
| 867 | < 0.0001 (< 0.0001, 0.0053, 0.9995) | < 0.0001 (< 0.0001, 1.0000, 0.7949) |
| 868 | < 0.0001 (0.3512, 0.0050, 0.9817) | < 0.0001 (< 0.0001, 1.0000, 0.7794) |
| 869 | 0.3992 (0.7130, 0.1395, 0.9791) | < 0.0001 (< 0.0001, 1.0000, 0.6496) |
| 870 | < 0.0001 (0.5640, 0.0397, 0.9960) | < 0.0001 (1.0000, 1.0000, 0.9806) |
| 871 | 1.2684 (0.4345, 0.3239, 0.2247) | 0.5000 (0.8889, 0.8889, 0.6968) |
| 872 | < 0.0001 (0.1318, 0.0073, 0.9995) | < 0.0001 (1.0000, 1.0000, 0.8925) |
| 873 | 1.0000 (0.7888, 1.0000, 0.8264) | < 0.0001 (1.0000, 1.0000, 0.9901) |
| 874 | < 0.0001 (< 0.0001, 0.0056, 0.9954) | < 0.0001 (1.0000, 1.0000, 0.9855) |
| 875 | 0.3481 (0.0052, 0.1006, 0.9439) | < 0.0001 (< 0.0001, 1.0000, 0.9195) |
| 876 | < 0.0001 (0.3121, 0.0065, 0.9733) | < 0.0001 (1.0000, 1.0000, 0.9995) |
| 877 | < 0.0001 (0.7130, 0.0126, 0.9955) | < 0.0001 (1.0000, 1.0000, 0.9442) |
| 878 | < 0.0001 (0.0158, 0.0037, 0.9987) | 0.4927 (0.6829, 0.6942, 0.4972) |
| 879 | < 0.0001 (0.0547, 0.0083, 0.9949) | 0.2500 (0.9630, 0.9630, 0.8096) |
| 880 | < 0.0001 (0.2583, 0.0083, 0.9948) | < 0.0001 (1.0000, 1.0000, 0.9357) |
| 881 | < 0.0001 (0.9962, 0.0083, 0.9988) | < 0.0001 (1.0000, 1.0000, 0.9981) |
| 882 | < 0.0001 (< 0.0001, 0.0073, 0.9964) | 0.4644 (0.9739, 0.8994, 0.7439) |
| 883 | < 0.0001 (< 0.0001, 0.0060, 0.9966) | < 0.0001 (1.0000, 1.0000, 0.9402) |
| 884 | 1.0000 (0.6667, 1.0000, 0.9230) | 0.1245 (0.9959, 0.9959, 0.9908) |
| 885 | 0.6720 (0.3625, 0.2738, 0.9082) | 0.5000 (0.6667, 0.6667, 0.3873) |
| 886 | < 0.0001 (0.2584, 0.0060, 0.9853) | < 0.0001 (1.0000, 1.0000, 0.9317) |
| 887 | 0.2226 (0.5084, 0.0231, 0.9357) | < 0.0001 (1.0000, 1.0000, 0.9198) |
| 888 | < 0.0001 (0.6667, 0.0142, 0.9979) | < 0.0001 (0.0003, 1.0000, 0.8828) |
| 889 | 0.6336 (0.0390, 0.2426, 0.7357) | 0.5111 (0.8839, 0.8856, 0.8612) |
| 890 | 0.8096 (0.9120, 0.3785, 0.9647) | < 0.0001 (< 0.0001, 1.0000, 0.8096) |
| 891 | < 0.0001 (1.0000, 0.0061, 1.0000) | < 0.0001 (1.0000, 1.0000, 0.9673) |
| 892 | < 0.0001 (0.3122, 0.0093, 1.0000) | < 0.0001 (1.0000, 1.0000, 0.9180) |
| 893 | 0.3167 (0.1164, 0.0780, 0.9481) | < 0.0001 (1.0000, 1.0000, 0.9301) |
| 894 | < 0.0001 (0.5551, 0.0085, 1.0000) | < 0.0001 (1.0000, 1.0000, 0.9961) |
| 895 | 0.7998 (0.0937, 0.3715, 0.9889) | < 0.0001 (< 0.0001, 1.0000, 0.7212) |
| 896 | 0.9050 (0.9998, 0.4303, 0.9604) | < 0.0001 (1.0000, 1.0000, 0.9254) |
| 897 | 1.2767 (0.9547, 0.3196, 0.7917) | < 0.0001 (< 0.0001, 1.0000, 0.8096) |
| 898 | 0.3015 (0.6419, 0.0675, 0.6318) | < 0.0001 (1.0000, 1.0000, 0.9403) |
| 899 | 0.6028 (< 0.0001, 0.2173, 0.9549) | 0.2320 (0.9739, 0.9682, 0.7276) |
| 900 | 1.3523 (0.0667, 0.2830, 0.5345) | < 0.0001 (1.0000, 1.0000, 0.9507) |
| 901 | 1.2686 (0.9969, 0.3238, 0.5615) | < 0.0001 (< 0.0001, 1.0000, 0.8627) |
| 902 | 1.2990 (0.3625, 0.3616, 0.5740) | < 0.0001 (< 0.0001, 1.0000, 0.7551) |
| 903 | < 0.0001 (0.1319, 0.0073, 0.9885) | < 0.0001 (1.0000, 1.0000, 0.9670) |
| 904 | 0.3170 (0.7066, 0.0782, 0.8353) | < 0.0001 (1.0000, 1.0000, 0.9669) |
| 905 | < 0.0001 (< 0.0001, 0.0056, 0.9876) | < 0.0001 (< 0.0001, 1.0000, 0.6874) |
| 906 | 1.0000 (0.0261, 1.0000, 0.9798) | < 0.0001 (1.0000, 1.0000, 0.9830) |
| 907 | < 0.0001 (0.2964, 0.0060, 0.9904) | < 0.0001 (1.0000, 1.0000, 0.9811) |
| 908 | < 0.0001 (0.7130, 0.0061, 0.9999) | < 0.0001 (1.0000, 1.0000, 0.9588) |
| 909 | 1.0000 (< 0.0001, 1.0000, 0.9798) | < 0.0001 (< 0.0001, 1.0000, 0.8854) |
| 910 | < 0.0001 (0.2963, 0.0060, 0.9854) | < 0.0001 (1.0000, 1.0000, 0.9180) |
| 911 | < 0.0001 (0.4445, 0.0060, 1.0000) | < 0.0001 (< 0.0001, 1.0000, 0.7996) |
| 912 | 0.5575 (1.0000, 0.1802, 0.9974) | < 0.0001 (1.0000, 1.0000, 0.9460) |
| 913 | < 0.0001 (1.0000, 0.0060, 0.9977) | < 0.0001 (< 0.0001, 1.0000, 0.8174) |
| 914 | 0.6833 (< 0.0001, 0.3422, 0.9865) | 0.5000 (0.8889, 0.8889, 0.7613) |
| 915 | < 0.0001 (0.4890, 0.0117, 0.9964) | < 0.0001 (< 0.0001, 1.0000, 0.6618) |
| 916 | < 0.0001 (0.1317, 0.0061, 0.9948) | < 0.0001 (1.0000, 1.0000, 0.9890) |
| 917 | 1.0000 (0.0096, 1.0000, 0.9552) | < 0.0001 (1.0000, 1.0000, 0.9873) |
| 918 | 0.3358 (0.2965, 0.0916, 0.9981) | < 0.0001 (< 0.0001, 1.0000, 0.8854) |
| 919 | < 0.0001 (< 0.0001, 0.0126, 1.0000) | < 0.0001 (1.0000, 1.0000, 0.9364) |
| 920 | < 0.0001 (0.6667, 0.0061, 0.9987) | < 0.0001 (< 0.0001, 1.0000, 0.7654) |
| 921 | < 0.0001 (< 0.0001, 0.0053, 0.9905) | < 0.0001 (< 0.0001, 1.0000, 0.7996) |
| 922 | 0.9207 (0.6659, 0.4424, 0.7173) | < 0.0001 (1.0000, 1.0000, 0.9409) |
| 923 | < 0.0001 (1.0000, 0.0050, 0.9963) | < 0.0001 (< 0.0001, 1.0000, 0.8523) |
| 924 | 1.0000 (0.0391, 1.0000, 0.8736) | 0.2500 (0.9630, 0.9630, 0.7843) |
| 925 | < 0.0001 (0.6667, 0.0083, 0.9948) | < 0.0001 (1.0000, 1.0000, 0.9910) |
| 926 | 0.6965 (0.1310, 0.2933, 0.6257) | < 0.0001 (1.0000, 1.0000, 0.8678) |
| 927 | < 0.0001 (0.2280, 0.0065, 0.9651) | < 0.0001 (< 0.0001, 1.0000, 0.7996) |
| 928 | < 0.0001 (0.9998, 0.0056, 0.9974) | < 0.0001 (1.0000, 1.0000, 0.9964) |
| 929 | 0.7226 (0.0023, 0.3137, 0.9918) | 0.2079 (0.9684, 0.9747, 0.8545) |
| 930 | 1.0000 (0.6664, 1.0000, 0.9404) | 0.5125 (0.6540, 0.6612, 0.6318) |
| 931 | < 0.0001 (< 0.0001, 0.0050, 0.9963) | < 0.0001 (1.0000, 1.0000, 1.0000) |
| 932 | 1.9064 (0.1843, 0.0770, 0.1069) | < 0.0001 (< 0.0001, 1.0000, 0.8634) |
| 933 | 1.0000 (< 0.0001, 1.0000, 0.9351) | < 0.0001 (< 0.0001, 1.0000, 0.8854) |
| 934 | < 0.0001 (0.1670, 0.0087, 0.9988) | < 0.0001 (1.0000, 1.0000, 0.9777) |
| 935 | 0.7226 (0.0174, 0.3137, 0.9302) | < 0.0001 (1.0000, 1.0000, 0.9918) |
| 936 | < 0.0001 (0.4447, 0.0050, 0.9980) | < 0.0001 (< 0.0001, 1.0000, 0.8634) |
| 937 | 1.2079 (0.7726, 0.3979, 0.3720) | < 0.0001 (1.0000, 1.0000, 0.9630) |
| 938 | 0.9211 (0.9976, 0.4426, 0.4531) | 0.1667 (0.9877, 0.9877, 0.8916) |
| 939 | < 0.0001 (0.4488, 0.0085, 0.9997) | < 0.0001 (1.0000, 1.0000, 0.9735) |
| 940 | < 0.0001 (0.0585, 0.0061, 0.9978) | < 0.0001 (< 0.0001, 1.0000, 0.8854) |
| 941 | 1.0000 (< 0.0001, 1.0000, 0.9798) | 0.2499 (0.9630, 0.9630, 0.7306) |
| 942 | < 0.0001 (0.4445, 0.0053, 1.0000) | < 0.0001 (1.0000, 1.0000, 0.8720) |
| 943 | < 0.0001 (0.7130, 0.0037, 0.9999) | < 0.0001 (1.0000, 1.0000, 0.9301) |
| 944 | < 0.0001 (0.7130, 0.0436, 0.9998) | 0.9817 (0.9041, 0.7462, 0.6524) |
| 945 | 1.0000 (< 0.0001, 1.0000, 0.9351) | < 0.0001 (1.0000, 1.0000, 0.9413) |
| 946 | 0.3481 (0.5084, 0.1007, 1.0000) | < 0.0001 (< 0.0001, 1.0000, 0.8081) |
| 947 | < 0.0001 (0.3625, 0.0073, 1.0000) | < 0.0001 (< 0.0001, 1.0000, 0.6746) |
| 948 | < 0.0001 (0.1976, 0.0065, 0.9966) | < 0.0001 (< 0.0001, 1.0000, 0.6874) |
| 949 | < 0.0001 (0.1318, 0.0117, 0.9989) | < 0.0001 (1.0000, 1.0000, 0.8988) |
| 950 | 1.0000 (0.0879, 1.0000, 0.9216) | < 0.0001 (< 0.0001, 1.0000, 0.9113) |
| 951 | 1.0000 (< 0.0001, 1.0000, 0.8736) | < 0.0001 (< 0.0001, 1.0000, 0.9113) |
| 952 | < 0.0001 (0.1975, 0.0056, 0.9584) | < 0.0001 (1.0000, 1.0000, 0.9755) |
| 953 | < 0.0001 (0.2382, 0.0126, 0.9972) | 0.2500 (0.9630, 0.9630, 0.8080) |
| 954 | 0.3013 (0.0752, 0.0674, 0.9554) | 0.5000 (0.8889, 0.8889, 0.8001) |
| 955 | < 0.0001 (0.4883, 0.0037, 0.9983) | < 0.0001 (1.0000, 1.0000, 0.9694) |
| 956 | < 0.0001 (0.1317, 0.0050, 0.9999) | < 0.0001 (1.0000, 1.0000, 0.9641) |
| 957 | 0.3637 (0.9977, 0.1124, 0.9880) | < 0.0001 (< 0.0001, 1.0000, 0.7949) |
| 958 | 0.2226 (0.4437, 0.0231, 0.9358) | < 0.0001 (1.0000, 1.0000, 0.9919) |
| 959 | < 0.0001 (0.3625, 0.0117, 1.0000) | < 0.0001 (1.0000, 1.0000, 0.9792) |
| 960 | < 0.0001 (0.4444, 0.0425, 0.9871) | < 0.0001 (1.0000, 1.0000, 0.8925) |
| 961 | 0.6338 (0.7776, 0.2428, 0.5015) | < 0.0001 (< 0.0001, 1.0000, 0.6618) |
| 962 | 0.3480 (0.1316, 0.1006, 0.9724) | 0.4882 (0.7033, 0.6829, 0.4274) |
| 963 | < 0.0001 (0.1977, 0.0045, 0.9986) | < 0.0001 (1.0000, 1.0000, 0.9630) |
| 964 | 0.3358 (0.1975, 0.0916, 0.9960) | < 0.0001 (< 0.0001, 1.0000, 0.8549) |
| 965 | 0.2786 (0.9949, 0.0528, 0.8998) | < 0.0001 (1.0000, 1.0000, 0.9733) |
| 966 | < 0.0001 (< 0.0001, 0.0053, 0.9980) | < 0.0001 (1.0000, 1.0000, 0.9437) |
| 967 | 0.3012 (0.6882, 0.0673, 0.9998) | < 0.0001 (< 0.0001, 1.0000, 0.8107) |
| 968 | 0.3013 (< 0.0001, 0.0674, 0.8406) | < 0.0001 (1.0000, 1.0000, 0.9644) |
| 969 | < 0.0001 (0.6667, 0.0073, 0.9885) | < 0.0001 (1.0000, 1.0000, 0.9748) |
| 970 | < 0.0001 (0.7130, 0.0087, 0.9786) | < 0.0001 (1.0000, 1.0000, 0.9605) |
| 971 | < 0.0001 (0.2964, 0.0065, 0.9733) | < 0.0001 (< 0.0001, 1.0000, 0.7592) |
| 972 | 1.5730 (1.0000, 0.2000, 0.9287) | 0.4641 (0.9739, 0.8995, 0.7273) |
| 973 | 0.3166 (1.0000, 0.0779, 0.7199) | < 0.0001 (1.0000, 1.0000, 0.9625) |
| 974 | < 0.0001 (0.2963, 0.0060, 1.0000) | < 0.0001 (1.0000, 1.0000, 0.9416) |
| 975 | 0.3170 (< 0.0001, 0.0782, 0.9290) | < 0.0001 (1.0000, 1.0000, 0.9919) |
| 976 | < 0.0001 (< 0.0001, 0.0060, 0.9978) | < 0.0001 (< 0.0001, 1.0000, 0.8854) |
| 977 | < 0.0001 (0.0400, 0.0050, 1.0000) | < 0.0001 (1.0000, 1.0000, 0.9397) |
| 978 | < 0.0001 (0.2969, 0.0061, 0.9807) | < 0.0001 (1.0000, 1.0000, 0.9317) |
| 979 | < 0.0001 (0.5552, 0.0083, 0.9993) | < 0.0001 (< 0.0001, 1.0000, 0.6891) |
| 980 | 1.0000 (0.0878, 1.0000, 0.9431) | < 0.0001 (< 0.0001, 1.0000, 0.8549) |
| 981 | < 0.0001 (0.4883, 0.0053, 0.9980) | 0.4994 (0.9877, 0.8891, 0.9617) |
| 982 | 0.6135 (< 0.0001, 0.2261, 0.8060) | < 0.0001 (< 0.0001, 1.0000, 0.8081) |
| 983 | < 0.0001 (1.0000, 0.0397, 0.9999) | < 0.0001 (1.0000, 1.0000, 0.9180) |
| 984 | < 0.0001 (0.0035, 0.0061, 0.9958) | < 0.0001 (1.0000, 1.0000, 0.9397) |
| 985 | 1.0000 (0.9630, 1.0000, 0.8868) | < 0.0001 (1.0000, 1.0000, 0.9962) |
| 986 | < 0.0001 (0.1317, 0.0099, 0.9984) | 0.4342 (0.9120, 0.9083, 0.5687) |
| 987 | < 0.0001 (0.8767, 0.0037, 0.9947) | < 0.0001 (1.0000, 1.0000, 0.9913) |
| 988 | 1.0000 (0.1976, 1.0000, 0.8612) | < 0.0001 (1.0000, 1.0000, 0.9285) |
| 989 | < 0.0001 (0.7066, 0.0424, 0.9986) | < 0.0001 (< 0.0001, 1.0000, 0.8858) |
| 990 | < 0.0001 (0.8537, 0.0107, 0.9998) | < 0.0001 (< 0.0001, 1.0000, 0.7949) |
| 991 | < 0.0001 (0.1889, 0.0142, 0.9999) | < 0.0001 (1.0000, 1.0000, 0.9992) |
| 992 | 1.0000 (0.1843, 1.0000, 0.9798) | < 0.0001 (1.0000, 1.0000, 0.9874) |
| 993 | < 0.0001 (0.1842, 0.0060, 0.9998) | < 0.0001 (< 0.0001, 1.0000, 0.7515) |
| 994 | < 0.0001 (0.2584, 0.0073, 0.9961) | < 0.0001 (1.0000, 1.0000, 0.9294) |
| 995 | < 0.0001 (0.6988, 0.0037, 0.9963) | < 0.0001 (1.0000, 1.0000, 0.9694) |
| 996 | 0.2863 (0.0725, 0.0576, 0.7188) | < 0.0001 (< 0.0001, 1.0000, 0.6746) |
| 997 | 2.0009 (< 0.0001, 0.0824, 0.5413) | < 0.0001 (< 0.0001, 1.0000, 0.8081) |
| 998 | < 0.0001 (0.1317, 0.0053, 0.9676) | < 0.0001 (1.0000, 1.0000, 0.9459) |
| 999 | 0.6493 (0.5081, 0.2554, 0.9908) | < 0.0001 (1.0000, 1.0000, 0.9968) |
| 1000 | 0.3013 (< 0.0001, 0.0674, 0.9954) | < 0.0001 (< 0.0001, 1.0000, 0.7949) |
| 1001 | 1.0000 (< 0.0001, 1.0000, 0.9798) | 0.1547 (0.9923, 0.9899, 0.8880) |
| 1002 | < 0.0001 (1.0000, 0.0060, 0.9904) | < 0.0001 (1.0000, 1.0000, 0.9803) |
| 1003 | 1.0000 (< 0.0001, 1.0000, 0.8565) | < 0.0001 (1.0000, 1.0000, 0.9301) |
| 1004 | 1.0000 (< 0.0001, 1.0000, 0.9351) | 0.5000 (0.8889, 0.8889, 0.7377) |
| 1005 | < 0.0001 (0.6988, 0.0041, 0.9905) | < 0.0001 (1.0000, 1.0000, 0.9683) |
| 1006 | 1.0000 (1.0000, 1.0000, 0.9480) | 0.5000 (0.9877, 0.8889, 0.8699) |
| 1007 | 0.3480 (0.2583, 0.1006, 0.9586) | < 0.0001 (1.0000, 1.0000, 0.9678) |
| 1008 | < 0.0001 (0.1317, 0.0425, 0.9993) | < 0.0001 (1.0000, 1.0000, 0.9686) |
| 1009 | < 0.0001 (0.2963, 0.0056, 0.9583) | < 0.0001 (< 0.0001, 1.0000, 0.7583) |
| 1010 | < 0.0001 (< 0.0001, 0.0073, 0.9999) | < 0.0001 (1.0000, 1.0000, 0.9854) |
| 1011 | < 0.0001 (0.6667, 0.0083, 0.9997) | < 0.0001 (1.0000, 1.0000, 0.9356) |
| 1012 | 0.3166 (0.6667, 0.0779, 0.9748) | < 0.0001 (1.0000, 1.0000, 0.9709) |
| 1013 | 0.4045 (1.0000, 0.1436, 0.9608) | < 0.0001 (< 0.0001, 1.0000, 0.7949) |
| 1014 | < 0.0001 (0.8889, 0.0087, 1.0000) | < 0.0001 (1.0000, 1.0000, 0.9364) |
| 1015 | < 0.0001 (0.3625, 0.0050, 0.9960) | < 0.0001 (1.0000, 1.0000, 0.9507) |
| 1016 | 1.0000 (0.2684, 1.0000, 0.9798) | 0.3499 (0.9794, 0.9526, 0.6977) |
| 1017 | < 0.0001 (1.0000, 0.0093, 0.9962) | < 0.0001 (< 0.0001, 1.0000, 0.8531) |
| 1018 | < 0.0001 (1.0000, 0.0073, 0.9998) | < 0.0001 (1.0000, 1.0000, 0.9626) |
| 1019 | 1.0000 (< 0.0001, 1.0000, 0.9569) | < 0.0001 (< 0.0001, 1.0000, 0.8854) |
| 1020 | < 0.0001 (< 0.0001, 0.0099, 0.9990) | < 0.0001 (< 0.0001, 1.0000, 0.7591) |
| 1021 | 0.3013 (0.1168, 0.0674, 0.9979) | < 0.0001 (1.0000, 1.0000, 0.9285) |
| 1022 | < 0.0001 (< 0.0001, 0.0397, 0.9995) | < 0.0001 (1.0000, 1.0000, 0.9409) |
| 1023 | 1.0000 (< 0.0001, 1.0000, 0.9798) | < 0.0001 (< 0.0001, 1.0000, 0.7016) |
| 1024 | 0.3105 (< 0.0001, 0.0737, 0.9662) | < 0.0001 (1.0000, 1.0000, 0.9287) |
| 1025 | 0.3245 (< 0.0001, 0.0835, 0.7299) | 0.5000 (0.8889, 0.8889, 0.6600) |
| 1026 | < 0.0001 (0.9993, 0.0060, 0.9854) | < 0.0001 (1.0000, 1.0000, 0.8928) |
| 1027 | 1.5908 (0.6668, 0.1679, 0.8691) | < 0.0001 (< 0.0001, 1.0000, 0.8858) |
| 1028 | < 0.0001 (0.6950, 0.0126, 0.9955) | < 0.0001 (< 0.0001, 1.0000, 0.8858) |
| 1029 | 1.0000 (< 0.0001, 1.0000, 0.9404) | < 0.0001 (1.0000, 1.0000, 0.9442) |
| 1030 | 1.0000 (0.1975, 1.0000, 0.8305) | < 0.0001 (1.0000, 1.0000, 0.9841) |
| 1031 | 0.9746 (0.0035, 0.4821, 0.1421) | < 0.0001 (< 0.0001, 1.0000, 0.7490) |
| 1032 | < 0.0001 (0.1317, 0.0397, 0.9903) | 0.3611 (0.9118, 0.9263, 0.8008) |
| 1033 | < 0.0001 (0.0879, 0.0013, 0.9988) | < 0.0001 (1.0000, 1.0000, 0.9168) |
| 1034 | 1.0000 (0.8889, 1.0000, 0.9816) | 2.3209 (0.9550, 0.3842, 0.2190) |
| 1035 | < 0.0001 (1.0000, 0.0093, 0.9967) | 0.4642 (0.9120, 0.8995, 0.6110) |
| 1036 | 0.3482 (0.2325, 0.1007, 0.9400) | < 0.0001 (1.0000, 1.0000, 0.8855) |
| 1037 | 0.3013 (0.7066, 0.0674, 0.9489) | < 0.0001 (1.0000, 1.0000, 0.9198) |
| 1038 | < 0.0001 (1.0000, 0.0424, 0.9998) | < 0.0001 (< 0.0001, 1.0000, 0.8854) |
| 1039 | < 0.0001 (0.1243, 0.0061, 1.0000) | < 0.0001 (< 0.0001, 1.0000, 0.7187) |
| 1040 | 1.0000 (0.0279, 1.0000, 0.9216) | < 0.0001 (1.0000, 1.0000, 0.9879) |
| 1041 | < 0.0001 (< 0.0001, 0.0093, 1.0000) | < 0.0001 (< 0.0001, 1.0000, 0.9195) |
| 1042 | < 0.0001 (0.4444, 0.0309, 0.9963) | < 0.0001 (< 0.0001, 1.0000, 0.8081) |
| 1043 | < 0.0001 (< 0.0001, 0.0037, 0.9914) | < 0.0001 (1.0000, 1.0000, 0.9838) |
| 1044 | < 0.0001 (< 0.0001, 0.0053, 0.9971) | < 0.0001 (< 0.0001, 1.0000, 0.8062) |
| 1045 | 0.7377 (0.4444, 0.3253, 0.7112) | < 0.0001 (< 0.0001, 1.0000, 0.8858) |
| 1046 | < 0.0001 (0.2963, 0.0053, 1.0000) | < 0.0001 (1.0000, 1.0000, 0.9121) |
| 1047 | < 0.0001 (< 0.0001, 0.0095, 0.9994) | 0.9994 (0.4538, 0.4685, 0.3204) |
| 1048 | < 0.0001 (0.6667, 0.0044, 0.9998) | 0.1087 (0.9923, 0.9974, 0.8739) |
| 1049 | < 0.0001 (< 0.0001, 0.0093, 0.9962) | < 0.0001 (< 0.0001, 1.0000, 0.6603) |
| 1050 | < 0.0001 (< 0.0001, 0.0056, 0.9961) | < 0.0001 (1.0000, 1.0000, 0.9317) |
| 1051 | < 0.0001 (< 0.0001, 0.0142, 0.9992) | < 0.0001 (< 0.0001, 1.0000, 0.8096) |
| 1052 | < 0.0001 (0.2963, 0.0060, 0.9989) | < 0.0001 (1.0000, 1.0000, 0.9286) |
| 1053 | < 0.0001 (1.0000, 0.0050, 0.9989) | < 0.0001 (1.0000, 1.0000, 1.0000) |
| 1054 | < 0.0001 (< 0.0001, 0.0343, 0.9921) | < 0.0001 (1.0000, 1.0000, 0.9863) |
| 1055 | 0.3065 (0.1975, 0.0710, 0.9761) | < 0.0001 (1.0000, 1.0000, 0.9121) |
| 1056 | < 0.0001 (< 0.0001, 0.0343, 0.9987) | < 0.0001 (< 0.0001, 1.0000, 0.8819) |
| 1057 | < 0.0001 (0.4445, 0.0056, 0.9971) | < 0.0001 (< 0.0001, 1.0000, 0.7592) |
| 1058 | < 0.0001 (0.2963, 0.0093, 1.0000) | < 0.0001 (1.0000, 1.0000, 0.9621) |
| 1059 | 1.0000 (0.4463, 1.0000, 0.9798) | < 0.0001 (< 0.0001, 1.0000, 0.6618) |
| 1060 | < 0.0001 (< 0.0001, 0.0065, 0.9976) | < 0.0001 (1.0000, 1.0000, 0.9160) |
| 1061 | < 0.0001 (0.6670, 0.0073, 0.9885) | < 0.0001 (1.0000, 1.0000, 0.9998) |
| 1062 | 1.0000 (< 0.0001, 1.0000, 0.8462) | < 0.0001 (1.0000, 1.0000, 0.8827) |
| 1063 | 1.0000 (0.5084, 1.0000, 0.9436) | < 0.0001 (< 0.0001, 1.0000, 0.7836) |
| 1064 | 0.3478 (0.5796, 0.1004, 0.9960) | < 0.0001 (1.0000, 1.0000, 0.9919) |
| 1065 | < 0.0001 (0.1837, 0.0073, 0.9894) | < 0.0001 (< 0.0001, 1.0000, 0.9269) |
| 1066 | < 0.0001 (< 0.0001, 0.0060, 0.9808) | < 0.0001 (< 0.0001, 1.0000, 0.7466) |
| 1067 | < 0.0001 (0.3623, 0.0065, 0.9952) | < 0.0001 (1.0000, 1.0000, 0.8736) |
| 1068 | < 0.0001 (0.6667, 0.0117, 1.0000) | < 0.0001 (< 0.0001, 1.0000, 0.8126) |
| 1069 | 0.3244 (0.7130, 0.0834, 0.7293) | < 0.0001 (< 0.0001, 1.0000, 0.7917) |
| 1070 | < 0.0001 (0.0195, 0.0142, 0.9996) | < 0.0001 (< 0.0001, 1.0000, 0.7319) |
| 1071 | < 0.0001 (0.4445, 0.0087, 0.9982) | < 0.0001 (1.0000, 1.0000, 0.9872) |
| 1072 | 1.0000 (0.8001, 1.0000, 0.9552) | < 0.0001 (< 0.0001, 1.0000, 0.6481) |
| 1073 | 1.0000 (< 0.0001, 1.0000, 0.9216) | < 0.0001 (< 0.0001, 1.0000, 0.7701) |
| 1074 | < 0.0001 (0.0391, 0.0050, 1.0000) | < 0.0001 (< 0.0001, 1.0000, 0.6410) |
| 1075 | < 0.0001 (0.1975, 0.0050, 0.9998) | < 0.0001 (1.0000, 1.0000, 1.0000) |
| 1076 | < 0.0001 (0.8901, 0.0083, 0.9993) | < 0.0001 (< 0.0001, 1.0000, 0.8526) |
| 1077 | 0.3014 (0.0005, 0.0675, 0.9814) | < 0.0001 (< 0.0001, 1.0000, 0.7908) |
| 1078 | < 0.0001 (0.0879, 0.0425, 0.9986) | < 0.0001 (1.0000, 1.0000, 0.8962) |
| 1079 | < 0.0001 (0.6507, 0.0037, 0.9695) | 0.5000 (0.6667, 0.6667, 0.4761) |
| 1080 | 1.0000 (0.9972, 1.0000, 0.9404) | < 0.0001 (1.0000, 1.0000, 0.9948) |
| 1081 | < 0.0001 (0.6667, 0.0037, 0.9963) | < 0.0001 (< 0.0001, 1.0000, 0.7319) |
| 1082 | < 0.0001 (0.5084, 0.0087, 1.0000) | < 0.0001 (< 0.0001, 1.0000, 0.7847) |
| 1083 | < 0.0001 (0.2389, 0.0060, 0.9854) | 0.5000 (0.8889, 0.8889, 0.6691) |
| 1084 | 0.7375 (1.0000, 0.3252, 0.9669) | < 0.0001 (1.0000, 1.0000, 0.9501) |
| 1085 | < 0.0001 (0.9990, 0.0085, 0.9999) | 0.5000 (0.6667, 0.6667, 0.3710) |
| 1086 | < 0.0001 (0.4992, 0.0060, 0.9947) | < 0.0001 (< 0.0001, 1.0000, 0.7925) |
| 1087 | 0.7226 (0.4445, 0.3137, 0.7666) | < 0.0001 (< 0.0001, 1.0000, 0.7966) |
| 1088 | < 0.0001 (0.1317, 0.0061, 0.9960) | < 0.0001 (< 0.0001, 1.0000, 0.8875) |
| 1089 | 0.9049 (1.0000, 0.4302, 0.8638) | < 0.0001 (< 0.0001, 1.0000, 0.8704) |
| 1090 | < 0.0001 (0.2490, 0.0085, 0.9997) | < 0.0001 (< 0.0001, 1.0000, 0.7942) |
| 1091 | < 0.0001 (0.0935, 0.0050, 0.9998) | < 0.0001 (1.0000, 1.0000, 0.9349) |
| 1092 | < 0.0001 (0.0077, 0.0050, 0.9996) | 0.9949 (0.4623, 0.4490, 0.2513) |
| 1093 | < 0.0001 (0.1978, 0.0050, 0.9997) | < 0.0001 (< 0.0001, 1.0000, 0.6716) |
| 1094 | < 0.0001 (0.1977, 0.0073, 0.9894) | 0.3338 (0.9987, 0.9986, 0.7289) |
| 1095 | < 0.0001 (0.1978, 0.0037, 0.9995) | 0.5000 (0.8889, 0.8889, 0.8753) |
| 1096 | < 0.0001 (0.5234, 0.0060, 0.9216) | < 0.0001 (1.0000, 1.0000, 0.9940) |
| 1097 | < 0.0001 (0.1842, 0.0060, 0.9904) | < 0.0001 (< 0.0001, 1.0000, 0.9208) |
| 1098 | 0.6501 (0.0010, 0.3215, 0.9439) | < 0.0001 (1.0000, 1.0000, 0.8818) |
| 1099 | 0.4409 (< 0.0001, 0.0902, 0.8322) | 0.9781 (0.4624, 0.4645, 0.2545) |
| 1100 | 1.0000 (0.2280, 1.0000, 0.9490) | < 0.0001 (0.0206, 1.0000, 0.7836) |
| 1101 | < 0.0001 (0.0011, 0.0065, 1.0000) | < 0.0001 (1.0000, 1.0000, 0.9716) |
| 1102 | 1.0000 (0.1318, 1.0000, 0.9798) | < 0.0001 (1.0000, 1.0000, 0.9366) |
| 1103 | 1.0000 (1.0000, 1.0000, 0.8871) | 0.1000 (0.9986, 0.9986, 0.9681) |
| 1104 | < 0.0001 (< 0.0001, 0.0087, 0.9949) | < 0.0001 (1.0000, 1.0000, 0.9915) |
| 1105 | 0.2862 (0.0261, 0.0576, 0.8978) | 0.4973 (0.8896, 0.8897, 0.7918) |
| 1106 | < 0.0001 (0.2964, 0.0037, 0.9769) | 0.9620 (0.4784, 0.4803, 0.3174) |
| 1107 | < 0.0001 (0.1317, 0.0087, 0.9843) | 0.5008 (0.6674, 0.6674, 0.4359) |
| 1108 | 0.9065 (0.1318, 0.4314, 0.9017) | < 0.0001 (1.0000, 1.0000, 0.8726) |
| 1109 | < 0.0001 (0.6667, 0.0436, 0.9979) | < 0.0001 (1.0000, 1.0000, 0.9883) |
| 1110 | 1.0000 (0.4444, 1.0000, 0.9552) | < 0.0001 (1.0000, 1.0000, 0.9858) |
| 1111 | 1.0000 (0.0476, 1.0000, 0.9552) | < 0.0001 (1.0000, 1.0000, 0.9174) |
| 1112 | < 0.0001 (0.1976, 0.0425, 0.9871) | 0.4992 (0.6676, 0.6677, 0.4659) |
| 1113 | < 0.0001 (0.7130, 0.0085, 0.9999) | < 0.0001 (< 0.0001, 1.0000, 0.6572) |
| 1114 | < 0.0001 (0.2964, 0.0142, 0.9830) | < 0.0001 (< 0.0001, 1.0000, 0.8526) |
| 1115 | 1.8132 (0.5084, 0.1744, 0.6538) | 1.4945 (0.6012, 0.6411, 0.5585) |
| 1116 | < 0.0001 (< 0.0001, 0.0050, 0.9817) | < 0.0001 (1.0000, 1.0000, 0.9919) |
| 1117 | 3.2634 (0.0878, 0.0009, 0.0490) | < 0.0001 (1.0000, 1.0000, 0.9164) |
| 1118 | 0.2786 (0.1839, 0.0528, 0.9443) | < 0.0001 (1.0000, 1.0000, 0.9292) |
| 1119 | < 0.0001 (0.6667, 0.0056, 0.9696) | < 0.0001 (1.0000, 1.0000, 0.9877) |
| 1120 | < 0.0001 (0.0002, 0.0087, 0.9988) | < 0.0001 (1.0000, 1.0000, 0.9530) |
| 1121 | 1.0000 (0.6667, 1.0000, 0.9798) | < 0.0001 (1.0000, 1.0000, 0.9657) |
| 1122 | 1.0000 (0.3516, 1.0000, 0.8456) | < 0.0001 (1.0000, 1.0000, 0.9311) |
| 1123 | < 0.0001 (< 0.0001, 0.0056, 0.9985) | < 0.0001 (1.0000, 1.0000, 0.9764) |
| 1124 | 1.0000 (0.1976, 1.0000, 0.9870) | < 0.0001 (1.0000, 1.0000, 0.9566) |
| 1125 | 1.0000 (0.0177, 1.0000, 0.9404) | < 0.0001 (1.0000, 1.0000, 1.0000) |
| 1126 | < 0.0001 (0.3624, 0.0060, 0.9995) | < 0.0001 (< 0.0001, 1.0000, 0.8681) |
| 1127 | 1.0000 (0.6442, 1.0000, 0.9342) | < 0.0001 (1.0000, 1.0000, 0.9842) |
| 1128 | 1.0000 (< 0.0001, 1.0000, 0.9552) | < 0.0001 (1.0000, 1.0000, 0.9966) |
| 1129 | < 0.0001 (< 0.0001, 0.0343, 0.9817) | < 0.0001 (1.0000, 1.0000, 0.9926) |
| 1130 | 1.4643 (0.6667, 0.2116, 0.1393) | < 0.0001 (1.0000, 1.0000, 0.9992) |
| 1131 | 0.3686 (0.4823, 0.1160, 0.9897) | < 0.0001 (1.0000, 1.0000, 0.9118) |
| 1132 | < 0.0001 (0.1840, 0.0065, 0.8213) | < 0.0001 (< 0.0001, 1.0000, 0.7404) |
| 1133 | < 0.0001 (0.1312, 0.0073, 0.9885) | < 0.0001 (1.0000, 1.0000, 0.9965) |
| 1134 | 1.0000 (0.0878, 1.0000, 0.9431) | < 0.0001 (< 0.0001, 1.0000, 0.8526) |
| 1135 | < 0.0001 (0.3625, 0.0050, 0.9996) | 0.2493 (0.9986, 0.9632, 0.9946) |
| 1136 | < 0.0001 (0.2963, 0.0126, 1.0000) | < 0.0001 (1.0000, 1.0000, 0.9662) |
| 1137 | < 0.0001 (0.4445, 0.0083, 1.0000) | < 0.0001 (1.0000, 1.0000, 0.9761) |
| 1138 | < 0.0001 (0.1319, 0.0343, 0.9998) | < 0.0001 (1.0000, 1.0000, 0.9819) |
| 1139 | < 0.0001 (0.5358, 0.0131, 0.9993) | < 0.0001 (1.0000, 1.0000, 0.9832) |
| 1140 | 1.2076 (0.7130, 0.3980, 0.3597) | 0.3337 (0.9990, 0.9989, 0.7260) |
| 1141 | 1.0454 (0.0879, 0.4696, 0.7137) | < 0.0001 (1.0000, 1.0000, 0.9819) |
| 1142 | 0.9207 (0.2752, 0.4424, 0.3003) | < 0.0001 (1.0000, 1.0000, 0.9606) |
| 1143 | < 0.0001 (< 0.0001, 0.0061, 0.9937) | < 0.0001 (1.0000, 1.0000, 0.9937) |
| 1144 | < 0.0001 (< 0.0001, 0.0142, 1.0000) | < 0.0001 (1.0000, 1.0000, 0.9746) |
| 1145 | < 0.0001 (0.9795, 0.0053, 0.9984) | < 0.0001 (< 0.0001, 1.0000, 0.9208) |
| 1146 | 0.3186 (0.0004, 0.0793, 0.5366) | < 0.0001 (< 0.0001, 1.0000, 0.6951) |
| 1147 | < 0.0001 (0.1759, 0.0065, 0.9999) | < 0.0001 (1.0000, 1.0000, 0.9987) |
| 1148 | 1.0000 (0.1316, 1.0000, 0.9870) | < 0.0001 (< 0.0001, 1.0000, 0.8526) |
| 1149 | 1.0000 (0.2961, 1.0000, 0.8516) | < 0.0001 (1.0000, 1.0000, 0.9996) |
| 1150 | < 0.0001 (0.7130, 0.0065, 0.9894) | 2.3613 (0.8976, 0.9200, 0.1723) |
| 1151 | 1.0000 (0.0392, 1.0000, 0.8619) | 0.4973 (0.8896, 0.8897, 0.7918) |
| 1152 | 1.0000 (0.4444, 1.0000, 0.8736) | < 0.0001 (< 0.0001, 1.0000, 0.8681) |
| 1153 | 1.0000 (1.0000, 1.0000, 0.9230) | < 0.0001 (1.0000, 1.0000, 0.9875) |
| 1154 | < 0.0001 (< 0.0001, 0.0397, 0.9991) | < 0.0001 (< 0.0001, 1.0000, 0.7917) |
| 1155 | 1.0000 (0.9258, 1.0000, 0.8462) | 0.4979 (0.8577, 0.9008, 0.6442) |
| 1156 | < 0.0001 (0.0023, 0.0126, 0.9850) | 0.4028 (0.9695, 0.9176, 0.7966) |
| 1157 | 1.0000 (0.4992, 1.0000, 0.8081) | 0.5000 (0.8889, 0.8889, 0.8649) |
| 1158 | < 0.0001 (0.1317, 0.0083, 1.0000) | 0.4999 (0.6763, 0.6909, 0.4064) |
| 1159 | 1.0000 (0.6944, 1.0000, 0.9525) | < 0.0001 (< 0.0001, 1.0000, 0.8875) |
| 1160 | < 0.0001 (0.0930, 0.0117, 0.9910) | < 0.0001 (1.0000, 1.0000, 0.9978) |
| 1161 | < 0.0001 (0.0586, 0.0397, 0.9903) | < 0.0001 (1.0000, 1.0000, 0.8788) |
| 1162 | 0.3187 (0.8889, 0.0794, 0.6237) | < 0.0001 (1.0000, 1.0000, 0.9558) |
| 1163 | < 0.0001 (0.7408, 0.0050, 0.9996) | < 0.0001 (1.0000, 1.0000, 0.9307) |
| 1164 | 1.0000 (0.0666, 1.0000, 0.9230) | < 0.0001 (1.0000, 1.0000, 0.9407) |
| 1165 | 1.0000 (1.0000, 1.0000, 0.9404) | 0.1555 (0.9896, 0.9898, 0.8901) |
| 1166 | 0.6971 (< 0.0001, 0.2937, 0.9063) | < 0.0001 (1.0000, 1.0000, 0.9531) |
| 1167 | < 0.0001 (< 0.0001, 0.0126, 0.9995) | < 0.0001 (1.0000, 1.0000, 0.9971) |
| 1168 | 0.3506 (0.0880, 0.1025, 0.9355) | < 0.0001 (< 0.0001, 1.0000, 0.9269) |
| 1169 | 0.3165 (< 0.0001, 0.0779, 0.7199) | < 0.0001 (1.0000, 1.0000, 0.9764) |
| 1170 | < 0.0001 (< 0.0001, 0.0041, 0.9905) | < 0.0001 (1.0000, 1.0000, 0.9986) |
| 1171 | 1.0000 (0.3625, 1.0000, 0.9798) | < 0.0001 (1.0000, 1.0000, 0.9940) |
| 1172 | < 0.0001 (0.2585, 0.0050, 0.9989) | 0.5000 (0.4444, 0.6667, 0.2317) |
| 1173 | 1.0000 (< 0.0001, 1.0000, 0.8868) | < 0.0001 (1.0000, 1.0000, 0.9937) |
| 1174 | 0.3167 (0.0043, 0.0779, 0.8957) | < 0.0001 (1.0000, 1.0000, 0.9570) |
| 1175 | 1.2753 (0.2585, 0.3203, 0.1528) | < 0.0001 (< 0.0001, 1.0000, 0.6951) |
| 1176 | 1.0000 (0.3624, 1.0000, 0.8693) | < 0.0001 (< 0.0001, 1.0000, 0.9269) |
| 1177 | 0.6698 (0.3300, 0.2287, 0.7787) | < 0.0001 (1.0000, 1.0000, 0.9989) |
| 1178 | 0.3611 (0.3356, 0.1103, 0.9648) | < 0.0001 (0.0013, 1.0000, 0.9638) |
| 1179 | < 0.0001 (0.0879, 0.0061, 1.0000) | 2.8339 (0.1022, 0.2871, 0.9934) |
| 1180 | 1.0000 (0.9959, 1.0000, 0.9816) | < 0.0001 (1.0000, 1.0000, 0.9398) |
| 1181 | < 0.0001 (0.2492, 0.0425, 0.9872) | < 0.0001 (1.0000, 1.0000, 0.9593) |
| 1182 | 0.9521 (0.0585, 0.4659, 0.1997) | < 0.0001 (1.0000, 1.0000, 0.9658) |
| 1183 | 1.0000 (0.0878, 1.0000, 0.8455) | < 0.0001 (< 0.0001, 1.0000, 0.6951) |
| 1184 | < 0.0001 (0.2963, 0.0424, 0.9998) | < 0.0001 (1.0000, 1.0000, 0.9353) |
| 1185 | < 0.0001 (0.7130, 0.0425, 0.9995) | < 0.0001 (1.0000, 1.0000, 0.9915) |
| 1186 | 1.3945 (0.0585, 0.2645, 0.9992) | 0.1667 (0.9877, 0.9877, 0.9231) |
| 1187 | < 0.0001 (0.0878, 0.0424, 0.9999) | < 0.0001 (< 0.0001, 1.0000, 0.8875) |
| 1188 | 0.6962 (0.5084, 0.2930, 0.9955) | 0.4927 (0.6934, 0.7087, 0.4365) |
| 1189 | 1.5585 (0.6667, 0.2045, 0.1748) | 0.0610 (0.9998, 1.0000, 0.9962) |
| 1190 | 0.3112 (0.0467, 0.0741, 0.8790) | < 0.0001 (< 0.0001, 1.0000, 0.8126) |
| 1191 | < 0.0001 (0.4445, 0.0056, 0.9877) | 0.4427 (0.9050, 0.9058, 0.5517) |
| 1192 | < 0.0001 (0.9093, 0.0060, 0.9991) | < 0.0001 (< 0.0001, 1.0000, 0.8526) |
| 1193 | < 0.0001 (0.9997, 0.0050, 0.9997) | < 0.0001 (1.0000, 1.0000, 0.9531) |
| 1194 | < 0.0001 (0.0260, 0.0142, 0.9944) | < 0.0001 (1.0000, 1.0000, 0.8827) |
| 1195 | 1.8155 (< 0.0001, 0.0922, 0.0618) | < 0.0001 (1.0000, 1.0000, 0.9834) |
| 1196 | < 0.0001 (0.0002, 0.0065, 0.9856) | < 0.0001 (1.0000, 1.0000, 0.9842) |
| 1197 | 1.0000 (< 0.0001, 1.0000, 0.9525) | 0.2500 (0.9630, 0.9630, 0.9478) |
| 1198 | 0.6836 (< 0.0001, 0.3424, 0.9864) | 0.1667 (0.9959, 0.9877, 0.9793) |
| 1199 | < 0.0001 (0.2955, 0.0085, 0.9999) | < 0.0001 (1.0000, 1.0000, 0.9566) |
| 1200 | < 0.0001 (0.7130, 0.0142, 0.9966) | < 0.0001 (< 0.0001, 1.0000, 0.8875) |
| 1201 | 0.3476 (0.1319, 0.1003, 0.9977) | < 0.0001 (< 0.0001, 1.0000, 0.9269) |
| 1202 | < 0.0001 (0.4444, 0.0060, 1.0000) | < 0.0001 (< 0.0001, 1.0000, 0.9208) |
| 1203 | < 0.0001 (0.0116, 0.0397, 0.9998) | < 0.0001 (1.0000, 1.0000, 0.9454) |
| 1204 | < 0.0001 (< 0.0001, 0.0083, 0.9999) | < 0.0001 (< 0.0001, 1.0000, 0.7599) |
| 1205 | < 0.0001 (0.6667, 0.0056, 0.9994) | < 0.0001 (1.0000, 1.0000, 0.9593) |
| 1206 | < 0.0001 (0.0467, 0.0061, 0.9997) | < 0.0001 (1.0000, 1.0000, 0.9878) |
| 1207 | < 0.0001 (0.9764, 0.0061, 0.9996) | < 0.0001 (< 0.0001, 1.0000, 0.8704) |
| 1208 | < 0.0001 (0.4736, 0.0117, 1.0000) | < 0.0001 (1.0000, 1.0000, 0.9990) |
| 1209 | 1.0000 (0.2964, 1.0000, 0.9480) | < 0.0001 (1.0000, 1.0000, 0.9977) |
| 1210 | < 0.0001 (0.7130, 0.0073, 0.9994) | < 0.0001 (< 0.0001, 1.0000, 0.8955) |
| 1211 | < 0.0001 (0.6603, 0.0107, 1.0000) | < 0.0001 (1.0000, 1.0000, 0.9773) |
| 1212 | < 0.0001 (0.8295, 0.0053, 1.0000) | < 0.0001 (1.0000, 1.0000, 0.9954) |
| 1213 | < 0.0001 (< 0.0001, 0.0120, 1.0000) | < 0.0001 (1.0000, 1.0000, 0.9684) |
| 1214 | < 0.0001 (< 0.0001, 0.0060, 0.9854) | < 0.0001 (1.0000, 1.0000, 0.9657) |
| 1215 | 1.0000 (0.0692, 1.0000, 0.9216) | 0.7500 (0.7901, 0.7901, 0.4768) |
| 1216 | < 0.0001 (0.7130, 0.0065, 0.9999) | < 0.0001 (1.0000, 1.0000, 0.9694) |
| 1217 | < 0.0001 (0.3527, 0.0083, 0.9948) | < 0.0001 (1.0000, 1.0000, 0.9970) |
| 1218 | 0.6959 (0.1620, 0.2928, 0.9930) | < 0.0001 (1.0000, 1.0000, 0.8824) |
| 1219 | < 0.0001 (< 0.0001, 0.0099, 0.9953) | < 0.0001 (1.0000, 1.0000, 0.8650) |
| 1220 | < 0.0001 (0.6944, 0.0397, 0.9982) | < 0.0001 (1.0000, 1.0000, 0.9861) |
| 1221 | < 0.0001 (0.6881, 0.0061, 0.9990) | < 0.0001 (1.0000, 1.0000, 0.9844) |
| 1222 | < 0.0001 (0.2583, 0.0126, 1.0000) | < 0.0001 (1.0000, 1.0000, 0.9366) |
| 1223 | 1.3397 (0.0010, 0.2475, 0.5349) | 0.5000 (0.8889, 0.8889, 0.8393) |
| 1224 | < 0.0001 (0.0173, 0.0061, 0.9958) | < 0.0001 (0.0010, 1.0000, 0.9969) |
| 1225 | < 0.0001 (0.9995, 0.0050, 0.9988) | < 0.0001 (1.0000, 1.0000, 0.9312) |
| 1226 | < 0.0001 (0.1953, 0.0117, 1.0000) | < 0.0001 (1.0000, 1.0000, 0.8817) |
| 1227 | < 0.0001 (< 0.0001, 0.0060, 0.9969) | 0.5000 (0.6763, 0.6908, 0.4064) |
| 1228 | 1.0000 (0.0586, 1.0000, 0.9816) | 0.4786 (0.9070, 0.8952, 0.6183) |
| 1229 | < 0.0001 (0.0011, 0.0343, 0.9997) | < 0.0001 (0.0252, 1.0000, 0.9761) |
| 1230 | < 0.0001 (0.0880, 0.0093, 1.0000) | < 0.0001 (1.0000, 1.0000, 0.9432) |
| 1231 | 1.0000 (0.7130, 1.0000, 0.9525) | < 0.0001 (1.0000, 1.0000, 0.9959) |
| 1232 | 0.3891 (0.6881, 0.1316, 0.9992) | < 0.0001 (< 0.0001, 1.0000, 0.6572) |
| 1233 | 5.4719 (0.5926, < 0.0001, 0.0516) | < 0.0001 (1.0000, 1.0000, 0.9662) |
| 1234 | 0.3479 (0.7130, 0.1005, 0.9924) | < 0.0001 (0.0134, 1.0000, 0.9174) |
| 1235 | 0.9580 (0.1975, 0.4702, 0.4631) | < 0.0001 (< 0.0001, 1.0000, 0.7966) |
| 1236 | < 0.0001 (0.1314, 0.0120, 1.0000) | < 0.0001 (< 0.0001, 1.0000, 0.9208) |
| 1237 | < 0.0001 (0.9176, 0.0117, 0.9873) | < 0.0001 (1.0000, 1.0000, 0.9453) |
| 1238 | 3.6136 (0.2963, 0.0007, 0.8913) | < 0.0001 (1.0000, 1.0000, 0.9715) |
| 1239 | 0.9509 (0.1760, 0.4650, 0.6922) | < 0.0001 (1.0000, 1.0000, 0.9530) |
| 1240 | 0.3187 (0.3526, 0.0794, 0.9852) | < 0.0001 (< 0.0001, 1.0000, 0.9269) |
| 1241 | 1.4640 (0.9997, 0.2117, 0.1392) | < 0.0001 (1.0000, 1.0000, 0.9935) |
| 1242 | 1.0000 (0.0880, 1.0000, 0.9798) | < 0.0001 (< 0.0001, 1.0000, 0.7404) |
| 1243 | < 0.0001 (0.8002, 0.0050, 1.0000) | 1.0601 (0.9237, 0.8393, 0.4301) |
| 1244 | < 0.0001 (0.9818, 0.0053, 0.9753) | < 0.0001 (1.0000, 1.0000, 0.9817) |
| 1245 | 1.2073 (< 0.0001, 0.3982, 0.7247) | < 0.0001 (< 0.0001, 1.0000, 0.6716) |
| 1246 | < 0.0001 (0.6667, 0.0073, 0.9964) | < 0.0001 (1.0000, 1.0000, 0.8962) |
| 1247 | < 0.0001 (0.4444, 0.0053, 0.9980) | < 0.0001 (1.0000, 1.0000, 0.9877) |
| 1248 | 0.6343 (0.1975, 0.2432, 0.8970) | 1.6971 (0.3949, 0.5326, 0.1143) |
| 1249 | < 0.0001 (0.0721, 0.0397, 0.9960) | < 0.0001 (1.0000, 1.0000, 0.9725) |
| 1250 | < 0.0001 (0.9974, 0.0397, 0.9999) | < 0.0001 (< 0.0001, 1.0000, 0.7925) |
| 1251 | 1.0000 (< 0.0001, 1.0000, 0.9870) | < 0.0001 (< 0.0001, 1.0000, 0.7966) |
| 1252 | < 0.0001 (0.1319, 0.0397, 0.9991) | < 0.0001 (1.0000, 1.0000, 0.9729) |
| 1253 | < 0.0001 (0.3625, 0.0061, 1.0000) | < 0.0001 (< 0.0001, 1.0000, 0.8681) |
| 1254 | < 0.0001 (0.0933, 0.0061, 1.0000) | < 0.0001 (1.0000, 1.0000, 0.9851) |
| 1255 | 1.3946 (0.7130, 0.2645, 0.2665) | < 0.0001 (1.0000, 1.0000, 0.9976) |
| 1256 | < 0.0001 (< 0.0001, 0.0117, 1.0000) | < 0.0001 (< 0.0001, 1.0000, 0.7590) |
| 1257 | < 0.0001 (0.1841, 0.0117, 0.9989) | < 0.0001 (1.0000, 1.0000, 0.9350) |
| 1258 | 1.0000 (0.4992, 1.0000, 0.9174) | < 0.0001 (1.0000, 1.0000, 0.9209) |
| 1259 | 1.0000 (0.3102, 1.0000, 0.9870) | < 0.0001 (1.0000, 1.0000, 0.9117) |
| 1260 | < 0.0001 (0.0051, 0.0061, 0.9999) | 0.5001 (0.8889, 0.8889, 0.8358) |
| 1261 | 0.4162 (0.0937, 0.1527, 0.9523) | < 0.0001 (1.0000, 1.0000, 0.9480) |
| **1262** | **5.5839 (< 0.0001, < 0.0001, 0.0042)** | 2.9052 (1.0000, 1.0000, **0.0008**) |
| 1263 | < 0.0001 (0.7066, 0.0065, 0.9981) | < 0.0001 (1.0000, 1.0000, 0.9668) |
| 1264 | < 0.0001 (< 0.0001, 0.0061, 0.9998) | < 0.0001 (1.0000, 1.0000, 0.9845) |
| 1265 | < 0.0001 (0.1318, 0.0085, 0.9945) | 0.7248 (0.7651, 0.7928, 0.7329) |
| 1266 | 0.6033 (0.0004, 0.2907, 0.6036) | < 0.0001 (1.0000, 1.0000, 0.9851) |
| 1267 | 0.3170 (0.4736, 0.0782, 0.8354) | < 0.0001 (1.0000, 1.0000, 0.9842) |
| 1268 | < 0.0001 (0.0260, 0.0424, 0.9996) | < 0.0001 (1.0000, 1.0000, 0.9991) |
| 1269 | 0.7990 (< 0.0001, 0.3709, 0.9969) | < 0.0001 (< 0.0001, 1.0000, 0.8878) |
| 1270 | 1.0000 (0.2963, 1.0000, 0.9798) | < 0.0001 (1.0000, 1.0000, 0.9773) |
| 1271 | 0.3358 (0.1310, 0.0916, 0.8852) | < 0.0001 (1.0000, 1.0000, 0.9117) |
| 1272 | 0.6344 (0.1318, 0.2433, 1.0000) | 0.4996 (0.7408, 0.8890, 0.5582) |
| 1273 | 0.3610 (0.1312, 0.1103, 0.8776) | < 0.0001 (1.0000, 1.0000, 0.9973) |
| 1274 | 0.3244 (< 0.0001, 0.0835, 0.7294) | < 0.0001 (1.0000, 1.0000, 0.8788) |
| 1275 | < 0.0001 (< 0.0001, 0.0083, 0.9996) | < 0.0001 (1.0000, 1.0000, 0.9970) |
| 1276 | 0.6496 (0.0878, 0.3211, 0.8943) | < 0.0001 (< 0.0001, 1.0000, 0.9269) |
| 1277 | < 0.0001 (< 0.0001, 0.0053, 0.9995) | 0.2460 (0.9895, 0.9641, 0.8873) |
| 1278 | < 0.0001 (< 0.0001, 0.0131, 0.9992) | < 0.0001 (1.0000, 1.0000, 0.9924) |
| 1279 | < 0.0001 (0.0817, 0.0424, 0.9986) | < 0.0001 (1.0000, 1.0000, 0.9851) |
| 1280 | 0.6377 (0.0261, 0.2460, 0.9504) | < 0.0001 (1.0000, 1.0000, 0.9567) |
| 1281 | 0.3480 (0.7408, 0.1006, 0.9998) | < 0.0001 (1.0000, 1.0000, 0.9122) |
| 1282 | 0.3186 (0.2963, 0.0793, 0.8343) | < 0.0001 (1.0000, 1.0000, 0.9716) |
| 1283 | 1.0000 (0.0936, 1.0000, 0.9798) | 0.4682 (0.9945, 0.9013, 0.9282) |
| 1284 | 1.0000 (< 0.0001, 1.0000, 0.9870) | 2.0000 (0.0173, 0.1975, 0.9677) |
| 1285 | < 0.0001 (0.8821, 0.0013, 0.9998) | < 0.0001 (< 0.0001, 1.0000, 0.7314) |
| 1286 | < 0.0001 (0.1760, 0.0424, 0.9977) | < 0.0001 (1.0000, 1.0000, 0.8774) |
| 1287 | 1.0000 (0.7130, 1.0000, 0.9342) | < 0.0001 (1.0000, 1.0000, 0.9712) |
| 1288 | < 0.0001 (0.0587, 0.0061, 0.9987) | < 0.0001 (1.0000, 1.0000, 0.9406) |
| 1289 | < 0.0001 (0.7130, 0.0013, 1.0000) | < 0.0001 (1.0000, 1.0000, 1.0000) |
| 1290 | 1.0000 (0.2581, 1.0000, 0.9490) | < 0.0001 (0.0274, 1.0000, 0.9653) |
| 1291 | < 0.0001 (0.9966, 0.0117, 1.0000) | < 0.0001 (1.0000, 1.0000, 0.9443) |
| 1292 | < 0.0001 (0.6944, 0.0050, 0.9999) | < 0.0001 (< 0.0001, 1.0000, 0.8293) |
| 1293 | < 0.0001 (0.4775, 0.0050, 0.9998) | < 0.0001 (1.0000, 1.0000, 0.9442) |
| 1294 | < 0.0001 (0.7130, 0.0050, 0.9997) | < 0.0001 (1.0000, 1.0000, 0.9853) |
| 1295 | < 0.0001 (0.3121, 0.0131, 0.9998) | < 0.0001 (1.0000, 1.0000, 0.9333) |
| 1296 | < 0.0001 (0.0096, 0.0053, 0.9994) | < 0.0001 (1.0000, 1.0000, 0.9641) |
| 1297 | < 0.0001 (< 0.0001, 0.0083, 0.9948) | < 0.0001 (1.0000, 1.0000, 0.9538) |
| 1298 | 1.3950 (< 0.0001, 0.2643, 0.6689) | < 0.0001 (< 0.0001, 1.0000, 0.9090) |
| 1299 | < 0.0001 (0.6988, 0.0083, 0.9999) | < 0.0001 (1.0000, 1.0000, 0.9621) |
| 1300 | < 0.0001 (0.7130, 0.0093, 1.0000) | < 0.0001 (< 0.0001, 1.0000, 0.7146) |
| 1301 | < 0.0001 (0.0262, 0.0425, 0.9670) | < 0.0001 (1.0000, 1.0000, 0.9793) |
| 1302 | < 0.0001 (0.1900, 0.0425, 0.9969) | < 0.0001 (1.0000, 1.0000, 0.9025) |
| 1303 | < 0.0001 (< 0.0001, 0.0061, 0.9960) | < 0.0001 (1.0000, 1.0000, 0.9887) |
| 1304 | < 0.0001 (0.0933, 0.0423, 0.9998) | < 0.0001 (< 0.0001, 1.0000, 0.9090) |
| 1305 | < 0.0001 (1.0000, 0.0425, 0.9969) | < 0.0001 (< 0.0001, 1.0000, 0.7411) |
| 1306 | 0.3015 (1.0000, 0.0675, 0.9488) | < 0.0001 (< 0.0001, 1.0000, 0.7032) |
| 1307 | < 0.0001 (0.7831, 0.0093, 1.0000) | < 0.0001 (1.0000, 1.0000, 0.9634) |
| 1308 | 0.3106 (0.1759, 0.0737, 0.8789) | < 0.0001 (1.0000, 1.0000, 0.9980) |
| 1309 | 0.6738 (0.3122, 0.3364, 0.7439) | < 0.0001 (< 0.0001, 1.0000, 0.6926) |
| 1310 | 0.2921 (0.7130, 0.0613, 0.6958) | < 0.0001 (< 0.0001, 1.0000, 0.7209) |
| 1311 | 0.7068 (0.0016, 0.3013, 0.8994) | < 0.0001 (1.0000, 1.0000, 0.8740) |
| 1312 | < 0.0001 (0.4740, 0.0397, 0.9995) | < 0.0001 (1.0000, 1.0000, 0.9585) |
| 1313 | < 0.0001 (0.1318, 0.0053, 0.9971) | < 0.0001 (1.0000, 1.0000, 0.9131) |
| 1314 | < 0.0001 (0.1672, 0.0424, 1.0000) | < 0.0001 (< 0.0001, 1.0000, 0.7292) |
| 1315 | 1.0000 (0.7130, 1.0000, 0.8213) | < 0.0001 (1.0000, 1.0000, 0.8500) |
| 1316 | < 0.0001 (< 0.0001, 0.0083, 0.9997) | 0.4863 (0.4742, 0.6854, 0.2474) |
| 1317 | < 0.0001 (0.6804, 0.0142, 1.0000) | < 0.0001 (1.0000, 1.0000, 0.9211) |
| 1318 | < 0.0001 (< 0.0001, 0.0041, 0.9905) | < 0.0001 (1.0000, 1.0000, 0.9949) |
| 1319 | 1.0000 (0.6950, 1.0000, 0.9816) | < 0.0001 (< 0.0001, 1.0000, 0.9090) |
| 1320 | < 0.0001 (0.0587, 0.0424, 0.9986) | < 0.0001 (< 0.0001, 1.0000, 0.7032) |
| 1321 | < 0.0001 (0.1316, 0.0425, 0.9973) | < 0.0001 (1.0000, 1.0000, 0.9684) |
| 1322 | < 0.0001 (< 0.0001, 0.0397, 0.9999) | < 0.0001 (< 0.0001, 1.0000, 0.8413) |
| 1323 | 1.0000 (0.0339, 1.0000, 0.9870) | < 0.0001 (< 0.0001, 1.0000, 0.9090) |
| 1324 | 0.3480 (1.0000, 0.1006, 0.9978) | < 0.0001 (1.0000, 1.0000, 0.9879) |
| 1325 | < 0.0001 (0.1975, 0.0060, 0.9854) | < 0.0001 (1.0000, 1.0000, 0.9984) |
| 1326 | 0.8814 (< 0.0001, 0.3983, 0.5936) | < 0.0001 (1.0000, 1.0000, 0.9879) |
| 1327 | < 0.0001 (0.7130, 0.0056, 0.9948) | < 0.0001 (0.0261, 1.0000, 0.9554) |
| 1328 | < 0.0001 (0.2963, 0.0037, 0.9914) | < 0.0001 (1.0000, 1.0000, 0.9734) |
| 1329 | < 0.0001 (1.0000, 0.0397, 0.9903) | < 0.0001 (< 0.0001, 1.0000, 0.9090) |
| 1330 | < 0.0001 (< 0.0001, 0.0060, 0.9998) | < 0.0001 (1.0000, 1.0000, 0.9891) |
| **1331** | **3.7982 (< 0.0001, < 0.0001, 0.0677)** | 0.5000 (0.7407, 0.8889, 0.5051) |
| 1332 | < 0.0001 (0.9990, 0.0073, 0.9987) | < 0.0001 (< 0.0001, 1.0000, 0.7146) |
| 1333 | 0.6335 (1.0000, 0.2426, 0.5016) | < 0.0001 (1.0000, 1.0000, 0.9873) |
| 1334 | 1.0000 (0.2963, 1.0000, 0.9411) | < 0.0001 (< 0.0001, 1.0000, 0.8120) |
| 1335 | < 0.0001 (0.7130, 0.0037, 1.0000) | 0.4586 (0.9031, 0.9011, 0.6706) |
| 1336 | 0.3186 (0.1977, 0.0793, 0.9459) | < 0.0001 (1.0000, 1.0000, 0.9396) |
| 1337 | < 0.0001 (0.9980, 0.0061, 0.9978) | < 0.0001 (1.0000, 1.0000, 0.8996) |
| 1338 | < 0.0001 (0.0585, 0.0061, 1.0000) | < 0.0001 (1.0000, 1.0000, 0.9233) |
| 1339 | 1.0000 (0.0879, 1.0000, 0.9379) | < 0.0001 (< 0.0001, 1.0000, 0.8270) |
| 1340 | < 0.0001 (0.0172, 0.0060, 0.9904) | < 0.0001 (1.0000, 1.0000, 0.9621) |
| 1341 | < 0.0001 (0.0076, 0.0060, 0.9896) | < 0.0001 (1.0000, 1.0000, 0.9963) |
| 1342 | < 0.0001 (0.1346, 0.0060, 0.9947) | < 0.0001 (< 0.0001, 1.0000, 0.9090) |
| 1343 | 0.6389 (0.1312, 0.2469, 0.9728) | < 0.0001 (< 0.0001, 1.0000, 0.8293) |
| 1344 | < 0.0001 (0.6667, 0.0037, 0.9880) | < 0.0001 (< 0.0001, 1.0000, 0.7138) |
| 1345 | < 0.0001 (1.0000, 0.0424, 1.0000) | < 0.0001 (< 0.0001, 1.0000, 0.7437) |
| 1346 | < 0.0001 (0.7854, 0.0425, 0.9998) | < 0.0001 (< 0.0001, 1.0000, 0.7375) |
| 1347 | < 0.0001 (0.3625, 0.0087, 0.9993) | < 0.0001 (1.0000, 1.0000, 0.9893) |
| 1348 | 0.6498 (< 0.0001, 0.3213, 0.9439) | < 0.0001 (1.0000, 1.0000, 0.9890) |
| 1349 | < 0.0001 (0.4444, 0.0061, 0.9937) | 0.4760 (0.7089, 0.7003, 0.2738) |
| 1350 | < 0.0001 (0.4445, 0.0421, 1.0000) | < 0.0001 (1.0000, 1.0000, 0.9682) |
| 1351 | < 0.0001 (0.0062, 0.0061, 0.9999) | < 0.0001 (1.0000, 1.0000, 0.9363) |
| 1352 | < 0.0001 (0.0667, 0.0131, 0.9963) | 0.3310 (< 0.0001, 0.9381, 0.8293) |
| 1353 | < 0.0001 (< 0.0001, 0.0050, 0.9952) | 0.4026 (0.9070, 0.9176, 0.6646) |
| 1354 | < 0.0001 (< 0.0001, 0.0060, 0.9854) | < 0.0001 (0.9153, 1.0000, 0.5567) |
| 1355 | 1.1994 (1.0000, 0.3800, 0.5571) | < 0.0001 (1.0000, 1.0000, 0.9292) |
| 1356 | < 0.0001 (0.5083, 0.0117, 1.0000) | < 0.0001 (1.0000, 1.0000, 0.9584) |
| 1357 | < 0.0001 (0.7130, 0.0053, 0.9980) | < 0.0001 (< 0.0001, 1.0000, 0.7449) |
| 1358 | < 0.0001 (0.0878, 0.0093, 1.0000) | < 0.0001 (1.0000, 1.0000, 1.0000) |
| 1359 | 0.2226 (0.4444, 0.0231, 0.9357) | < 0.0001 (1.0000, 1.0000, 0.9622) |
| 1360 | 0.3170 (0.9331, 0.0782, 0.9770) | < 0.0001 (1.0000, 1.0000, 0.9431) |
| 1361 | < 0.0001 (0.0668, 0.0126, 0.9998) | < 0.0001 (1.0000, 1.0000, 0.9694) |
| 1362 | < 0.0001 (0.6950, 0.0061, 0.9978) | < 0.0001 (1.0000, 1.0000, 0.8561) |
| 1363 | < 0.0001 (0.4883, 0.0061, 0.9807) | < 0.0001 (1.0000, 1.0000, 0.9641) |
| 1364 | < 0.0001 (0.3250, 0.0060, 0.9854) | < 0.0001 (< 0.0001, 1.0000, 0.7375) |
| 1365 | < 0.0001 (< 0.0001, 0.0424, 0.9993) | < 0.0001 (1.0000, 1.0000, 0.9809) |
| 1366 | 0.6345 (0.0586, 0.2434, 0.9995) | < 0.0001 (< 0.0001, 1.0000, 0.6315) |
| **1367** | **7.7737 (**0.0878**,B1289 < 0.0001, 0.0038)** | **2.1201** (1.0000, 0.6363, **0.0149**) |
| 1368 | 1.0000 (0.4451, 1.0000, 0.8511) | < 0.0001 (0.6465, 1.0000, 0.4257) |
| 1369 | < 0.0001 (< 0.0001, 0.0065, 0.9651) | < 0.0001 (1.0000, 1.0000, 0.9364) |
| 1370 | < 0.0001 (0.5083, 0.0093, 0.9912) | < 0.0001 (< 0.0001, 1.0000, 0.6315) |
| 1371 | < 0.0001 (0.6667, 0.0053, 0.9905) | < 0.0001 (1.0000, 1.0000, 0.8629) |
| 1372 | < 0.0001 (0.1976, 0.0086, 1.0000) | < 0.0001 (1.0000, 1.0000, 0.8628) |
| 1373 | < 0.0001 (0.6667, 0.0060, 0.9995) | < 0.0001 (< 0.0001, 1.0000, 0.6926) |
| 1374 | < 0.0001 (1.0000, 0.0085, 0.9998) | < 0.0001 (1.0000, 1.0000, 0.9797) |
| 1375 | < 0.0001 (0.6602, 0.0060, 0.9947) | < 0.0001 (1.0000, 1.0000, 0.9873) |
| 1376 | 1.0000 (0.9877, 1.0000, 0.9798) | < 0.0001 (< 0.0001, 1.0000, 0.6982) |
| 1377 | < 0.0001 (0.6950, 0.0093, 0.9999) | < 0.0001 (1.0000, 1.0000, 0.9330) |
| 1378 | < 0.0001 (0.1976, 0.0061, 0.9997) | < 0.0001 (< 0.0001, 1.0000, 0.9090) |
| 1379 | < 0.0001 (< 0.0001, 0.0050, 0.9817) | < 0.0001 (< 0.0001, 1.0000, 0.6480) |
| 1380 | < 0.0001 (< 0.0001, 0.0073, 1.0000) | < 0.0001 (1.0000, 1.0000, 0.8629) |
| 1381 | < 0.0001 (0.5084, 0.0083, 0.9948) | < 0.0001 (< 0.0001, 1.0000, 0.8413) |
| 1382 | 1.0000 (0.2584, 1.0000, 0.9870) | < 0.0001 (< 0.0001, 1.0000, 0.6926) |
| 1383 | < 0.0001 (0.6451, 0.0050, 1.0000) | < 0.0001 (1.0000, 1.0000, 0.9948) |
| 1384 | < 0.0001 (0.1976, 0.0061, 0.9937) | < 0.0001 (< 0.0001, 1.0000, 0.7484) |
| 1385 | < 0.0001 (1.0000, 0.0083, 0.9999) | < 0.0001 (< 0.0001, 1.0000, 0.8293) |
| 1386 | < 0.0001 (< 0.0001, 0.0056, 0.9984) | < 0.0001 (< 0.0001, 1.0000, 0.7580) |
| 1387 | < 0.0001 (0.0936, 0.0425, 1.0000) | < 0.0001 (1.0000, 1.0000, 0.9961) |
| 1388 | 1.0000 (1.0000, 1.0000, 0.9870) | 0.3344 (< 0.0001, 0.9969, 0.8293) |
| 1389 | < 0.0001 (0.0541, 0.0117, 0.9873) | 0.5000 (0.9939, 0.8889, 0.5935) |
| 1390 | 1.0000 (0.9994, 1.0000, 0.8868) | < 0.0001 (0.9877, 1.0000, 0.8961) |
| 1391 | < 0.0001 (0.1975, 0.0060, 0.9998) | < 0.0001 (< 0.0001, 1.0000, 0.7375) |
| 1392 | < 0.0001 (0.6667, 0.0083, 1.0000) | 0.1249 (< 0.0001, 0.9959, 0.9090) |
| 1393 | < 0.0001 (0.6447, 0.0060, 0.9947) | < 0.0001 (0.9986, 1.0000, 0.9529) |
| 1394 | < 0.0001 (1.0000, 0.0056, 0.9884) | < 0.0001 (1.0000, 1.0000, 0.9392) |
| 1395 | < 0.0001 (0.2753, 0.0424, 0.9998) | < 0.0001 (1.0000, 1.0000, 0.9944) |
| 1396 | < 0.0001 (0.1310, 0.0061, 0.9988) | 1.8027 (1.0000, 0.5064, 0.9998) |
| 1397 | < 0.0001 (< 0.0001, 0.0073, 0.9987) | < 0.0001 (0.7312, 1.0000, 0.4058) |
| 1398 | < 0.0001 (1.0000, 0.0127, 0.9995) | < 0.0001 (1.0000, 1.0000, 0.9331) |
| 1399 | < 0.0001 (0.2972, 0.0065, 0.9894) | < 0.0001 (1.0000, 1.0000, 0.9637) |
| 1400 | 1.2715 (0.1977, 0.3223, 0.1095) | < 0.0001 (1.0000, 1.0000, 0.9814) |
| 1401 | < 0.0001 (0.5084, 0.0065, 0.9987) | < 0.0001 (< 0.0001, 1.0000, 0.7126) |
| 1402 | < 0.0001 (1.0000, 0.0107, 0.9999) | < 0.0001 (1.0000, 1.0000, 0.9794) |
| 1403 | 0.2226 (< 0.0001, 0.0231, 0.9357) | < 0.0001 (< 0.0001, 1.0000, 0.8293) |
| 1404 | 1.0000 (0.3527, 1.0000, 0.8868) | < 0.0001 (1.0000, 1.0000, 0.9584) |
| 1405 | < 0.0001 (0.0932, 0.0060, 0.9998) | < 0.0001 (1.0000, 1.0000, 0.9984) |
| 1406 | 1.0000 (0.0015, 1.0000, 0.9552) | < 0.0001 (1.0000, 1.0000, 0.9999) |
| 1407 | 1.0000 (< 0.0001, 1.0000, 0.9870) | < 0.0001 (1.0000, 1.0000, 0.9794) |
| 1408 | < 0.0001 (< 0.0001, 0.0050, 0.9999) | 0.7302 (1.0000, 0.7988, 0.9646) |
| 1409 | 0.3171 (1.0000, 0.0782, 0.9997) | 0.4994 (0.3960, 0.6674, 0.2197) |
| 1410 | < 0.0001 (< 0.0001, 0.0061, 0.9990) | < 0.0001 (0.4453, 1.0000, 0.2231) |
| 1411 | 0.3106 (0.0195, 0.0738, 0.9702) | < 0.0001 (< 0.0001, 1.0000, 0.6926) |
| 1412 | < 0.0001 (< 0.0001, 0.0126, 1.0000) | < 0.0001 (1.0000, 1.0000, 0.9755) |
| 1413 | 1.0000 (0.4830, 1.0000, 0.9870) | 0.2165 (< 0.0001, 0.9733, 0.8293) |
| 1414 | < 0.0001 (< 0.0001, 0.0117, 0.9996) | < 0.0001 (0.9959, 1.0000, 0.7793) |
| 1415 | 0.9574 (0.1837, 0.4698, 0.9001) | < 0.0001 (1.0000, 1.0000, 0.9879) |
| 1416 | < 0.0001 (0.6667, 0.0061, 0.9807) | < 0.0001 (< 0.0001, 1.0000, 0.7181) |
| 1417 | < 0.0001 (0.3624, 0.0117, 1.0000) | < 0.0001 (1.0000, 1.0000, 0.9479) |
| 1418 | 1.0000 (0.5084, 1.0000, 0.9525) | < 0.0001 (1.0000, 1.0000, 0.9913) |
| 1419 | 0.3170 (< 0.0001, 0.0782, 0.9841) | < 0.0001 (1.0000, 1.0000, 0.9621) |
| 1420 | < 0.0001 (0.7110, 0.0107, 0.9960) | < 0.0001 (1.0000, 1.0000, 0.9219) |
| 1421 | < 0.0001 (< 0.0001, 0.0056, 0.9884) | < 0.0001 (< 0.0001, 1.0000, 0.8609) |
| 1422 | < 0.0001 (0.0879, 0.0060, 0.9966) | < 0.0001 (1.0000, 1.0000, 0.9935) |
| 1423 | < 0.0001 (< 0.0001, 0.0131, 0.9963) | < 0.0001 (< 0.0001, 1.0000, 0.9185) |
| 1424 | < 0.0001 (< 0.0001, 0.0050, 0.9997) | < 0.0001 (1.0000, 1.0000, 0.9980) |
| 1425 | < 0.0001 (< 0.0001, 0.0065, 1.0000) | < 0.0001 (< 0.0001, 1.0000, 0.9386) |
| 1426 | 1.0000 (0.0260, 1.0000, 0.9870) | < 0.0001 (1.0000, 1.0000, 0.9308) |
| 1427 | 1.0000 (1.0000, 1.0000, 0.9798) | < 0.0001 (1.0000, 1.0000, 0.9565) |
| 1428 | < 0.0001 (1.0000, 0.0060, 0.9978) | 0.5092 (1.0000, 0.6626, 0.9935) |
| 1429 | < 0.0001 (< 0.0001, 0.0073, 0.9894) | < 0.0001 (0.6532, 1.0000, 0.6540) |
| 1430 | 1.0000 (0.7065, 1.0000, 0.9342) | 0.4527 (< 0.0001, 0.9029, 0.9266) |
| 1431 | 0.3013 (0.4367, 0.0674, 1.0000) | < 0.0001 (0.8996, 1.0000, 0.7438) |
| 1432 | < 0.0001 (0.1245, 0.0061, 0.9998) | 0.2406 (< 0.0001, 0.9842, 0.8711) |
| 1433 | 0.3244 (0.4444, 0.0834, 0.5027) | 0.8291 (0.9985, 0.7927, 0.9702) |
| 1434 | < 0.0001 (1.0000, 0.0060, 0.9998) | < 0.0001 (0.7640, 1.0000, 0.5611) |
| 1435 | 0.3779 (1.0000, 0.1231, 0.9981) | < 0.0001 (1.0000, 1.0000, 0.9961) |
| 1436 | 1.0000 (0.0491, 1.0000, 0.9798) | < 0.0001 (< 0.0001, 1.0000, 0.8753) |
| 1437 | 0.3359 (0.6882, 0.0917, 0.9410) | < 0.0001 (< 0.0001, 1.0000, 0.7613) |
| 1438 | 0.5574 (0.4444, 0.1801, 0.5244) | < 0.0001 (1.0000, 1.0000, 0.9965) |
| 1439 | < 0.0001 (1.0000, 0.0065, 0.9981) | < 0.0001 (1.0000, 1.0000, 0.9695) |
| 1440 | < 0.0001 (< 0.0001, 0.0061, 0.9988) | < 0.0001 (< 0.0001, 1.0000, 0.8366) |
| 1441 | 0.3359 (0.3300, 0.0917, 0.8794) | < 0.0001 (1.0000, 1.0000, 0.9870) |
| 1442 | < 0.0001 (0.4444, 0.0060, 1.0000) | < 0.0001 (1.0000, 1.0000, 0.9997) |
| 1443 | < 0.0001 (0.4444, 0.0050, 0.9951) | < 0.0001 (1.0000, 1.0000, 0.9846) |
| 1444 | 0.3106 (0.4450, 0.0738, 0.9664) | < 0.0001 (< 0.0001, 1.0000, 0.8858) |
| 1445 | < 0.0001 (< 0.0001, 0.0120, 1.0000) | < 0.0001 (1.0000, 1.0000, 0.9983) |
| 1446 | < 0.0001 (0.1120, 0.0037, 0.9963) | 0.4999 (< 0.0001, 0.8889, 0.9644) |
| 1447 | < 0.0001 (0.6667, 0.0073, 0.9998) | < 0.0001 (0.8889, 1.0000, 0.6964) |
| 1448 | 1.0000 (0.6667, 1.0000, 0.9342) | < 0.0001 (< 0.0001, 1.0000, 0.7587) |
| 1449 | 0.3481 (0.0878, 0.1007, 0.9882) | < 0.0001 (< 0.0001, 1.0000, 0.8753) |
| 1450 | 1.0000 (0.7130, 1.0000, 0.8213) | 0.5000 (1.0000, 0.8889, 0.9777) |
| 1451 | < 0.0001 (< 0.0001, 0.0083, 0.9996) | < 0.0001 (0.8889, 1.0000, 0.7939) |
| 1452 | 1.0927 (0.2490, 0.4399, 0.3942) | 0.4304 (1.0000, 0.9095, 0.9870) |
| 1453 | < 0.0001 (0.4446, 0.0126, 0.9986) | < 0.0001 (0.9745, 1.0000, 0.7415) |
| 1454 | 1.0000 (< 0.0001, 1.0000, 0.8213) | < 0.0001 (< 0.0001, 1.0000, 0.7699) |
| 1455 | < 0.0001 (< 0.0001, 0.0065, 0.9651) | < 0.0001 (1.0000, 1.0000, 0.9965) |
| 1456 | 0.3244 (0.9960, 0.0834, 0.7293) | < 0.0001 (< 0.0001, 1.0000, 0.9099) |
| 1457 | 0.3173 (0.6944, 0.0784, 0.6459) | 0.2500 (< 0.0001, 0.9630, 0.8609) |
| 1458 | 0.3066 (0.7593, 0.0710, 0.9911) | < 0.0001 (0.9630, 1.0000, 0.9320) |
| 1459 | < 0.0001 (0.6944, 0.0117, 0.9999) | < 0.0001 (1.0000, 1.0000, 0.9351) |
| 1460 | 1.0000 (0.2965, 1.0000, 0.9230) | < 0.0001 (1.0000, 1.0000, 0.9864) |
| 1461 | < 0.0001 (0.5926, 0.0126, 1.0000) | 0.5000 (< 0.0001, 0.6667, 0.9644) |
| 1462 | 0.2226 (0.5084, 0.0231, 0.9357) | < 0.0001 (0.6667, 1.0000, 0.3930) |
| 1463 | 0.3065 (0.2326, 0.0710, 0.7449) | < 0.0001 (< 0.0001, 1.0000, 0.8642) |
| 1464 | < 0.0001 (0.6667, 0.0065, 0.9966) | < 0.0001 (< 0.0001, 1.0000, 0.7060) |
| 1465 | 1.0000 (0.4739, 1.0000, 0.9552) | < 0.0001 (1.0000, 1.0000, 0.9836) |
| 1466 | < 0.0001 (0.6667, 0.0060, 0.9854) | < 0.0001 (1.0000, 1.0000, 0.9999) |
| 1467 | < 0.0001 (0.6667, 0.0061, 0.9997) | < 0.0001 (< 0.0001, 1.0000, 0.8119) |
| 1468 | < 0.0001 (0.0668, 0.0093, 0.9869) | < 0.0001 (< 0.0001, 1.0000, 0.8366) |
| 1469 | 1.0000 (0.4444, 1.0000, 0.9798) | < 0.0001 (1.0000, 1.0000, 0.9673) |
| 1470 | 1.0000 (0.4728, 1.0000, 0.9431) | < 0.0001 (< 0.0001, 1.0000, 0.9386) |
| 1471 | < 0.0001 (< 0.0001, 0.0013, 0.9998) | < 0.0001 (1.0000, 1.0000, 0.9996) |
| 1472 | 1.0000 (0.9764, 1.0000, 0.9798) | < 0.0001 (0.0367, 1.0000, 0.9127) |
| 1473 | 1.7919 (0.0126, 0.0665, 0.4268) | < 0.0001 (1.0000, 1.0000, 0.9857) |
| 1474 | 1.1995 (0.6667, 0.3800, 0.9937) | < 0.0001 (0.0030, 1.0000, 0.8702) |
| 1475 | < 0.0001 (0.0397, 0.0044, 0.9891) | < 0.0001 (1.0000, 1.0000, 0.9948) |
| 1476 | 1.0000 (0.5084, 1.0000, 0.9870) | < 0.0001 (1.0000, 1.0000, 0.9677) |
| 1477 | 1.3990 (0.1840, 0.2626, 0.9992) | < 0.0001 (< 0.0001, 1.0000, 0.7798) |
| 1478 | < 0.0001 (< 0.0001, 0.0087, 0.9999) | < 0.0001 (1.0000, 1.0000, 0.9802) |
| 1479 | < 0.0001 (0.2964, 0.0126, 1.0000) | < 0.0001 (< 0.0001, 1.0000, 0.9644) |
| 1480 | < 0.0001 (0.0390, 0.0061, 1.0000) | < 0.0001 (1.0000, 1.0000, 0.9769) |
| 1481 | 1.5819 (< 0.0001, 0.1708, 0.6047) | < 0.0001 (< 0.0001, 1.0000, 0.8609) |
| 1482 | < 0.0001 (0.9946, 0.0037, 1.0000) | < 0.0001 (1.0000, 1.0000, 0.9986) |
| 1483 | < 0.0001 (0.5084, 0.0061, 1.0000) | < 0.0001 (< 0.0001, 1.0000, 0.7668) |
| 1484 | < 0.0001 (0.1087, 0.0142, 0.9998) | < 0.0001 (1.0000, 1.0000, 0.9907) |
| 1485 | < 0.0001 (< 0.0001, 0.0050, 0.9874) | < 0.0001 (1.0000, 1.0000, 0.9427) |
| 1486 | < 0.0001 (0.0879, 0.0093, 0.9999) | < 0.0001 (1.0000, 1.0000, 0.9965) |
| 1487 | 1.0000 (0.1317, 1.0000, 0.9798) | 0.4818 (< 0.0001, 0.8943, 0.9266) |
| 1488 | < 0.0001 (0.5084, 0.0061, 0.9988) | < 0.0001 (0.9062, 1.0000, 0.6945) |
| 1489 | 0.3170 (< 0.0001, 0.0782, 1.0000) | < 0.0001 (< 0.0001, 1.0000, 0.9386) |
| 1490 | 0.6501 (0.9958, 0.3215, 1.0000) | < 0.0001 (1.0000, 1.0000, 0.9936) |
| 1491 | < 0.0001 (0.0026, 0.0093, 0.9977) | < 0.0001 (1.0000, 1.0000, 0.9312) |
| 1492 | < 0.0001 (0.7065, 0.0131, 0.9963) | < 0.0001 (1.0000, 1.0000, 0.9997) |
| 1493 | 4.8930 (0.3625, < 0.0001, 0.0752) | 0.4493 (1.0000, 0.9039, 0.9695) |
| 1494 | 1.0448 (0.9989, 0.4700, 0.1681) | < 0.0001 (0.8997, 1.0000, 0.7315) |
| 1495 | 1.0000 (< 0.0001, 1.0000, 0.9216) | < 0.0001 (1.0000, 1.0000, 0.9906) |
| 1496 | < 0.0001 (1.0000, 0.0117, 0.9963) | < 0.0001 (1.0000, 1.0000, 0.9437) |
| 1497 | 1.0000 (0.9893, 1.0000, 0.9798) | < 0.0001 (1.0000, 1.0000, 0.9993) |
| 1498 | 0.4405 (0.9984, 0.0900, 0.9592) | < 0.0001 (1.0000, 1.0000, 0.9529) |
| 1499 | < 0.0001 (0.7130, 0.0050, 0.9999) | < 0.0001 (0.0374, 1.0000, 0.9987) |
| 1500 | < 0.0001 (0.5084, 0.0126, 0.9972) | < 0.0001 (1.0000, 1.0000, 0.9649) |
| 1501 | < 0.0001 (0.3625, 0.0343, 0.9921) | < 0.0001 (1.0000, 1.0000, 0.9649) |
| 1502 | < 0.0001 (0.6667, 0.0142, 1.0000) | < 0.0001 (1.0000, 1.0000, 0.9779) |
| 1503 | < 0.0001 (0.0303, 0.0050, 0.9951) | < 0.0001 (< 0.0001, 1.0000, 0.9386) |
| 1504 | < 0.0001 (0.0392, 0.0060, 0.9904) | < 0.0001 (< 0.0001, 1.0000, 0.8702) |
| 1505 | < 0.0001 (< 0.0001, 0.0045, 0.9995) | < 0.0001 (1.0000, 1.0000, 0.9914) |
| 1506 | 1.0000 (0.2584, 1.0000, 0.9216) | < 0.0001 (< 0.0001, 1.0000, 0.8346) |
| 1507 | < 0.0001 (0.0400, 0.0056, 0.9995) | 0.4528 (1.0000, 0.9029, 0.9573) |
| 1508 | 1.0000 (0.1978, 1.0000, 0.9525) | < 0.0001 (0.8996, 1.0000, 0.7437) |
| 1509 | < 0.0001 (0.5084, 0.0397, 0.9999) | < 0.0001 (< 0.0001, 1.0000, 0.9386) |
| 1510 | 1.0000 (< 0.0001, 1.0000, 0.9798) | < 0.0001 (1.0000, 1.0000, 0.9871) |
| 1511 | < 0.0001 (0.4444, 0.0065, 0.9894) | < 0.0001 (1.0000, 1.0000, 0.9696) |
| 1512 | < 0.0001 (0.1976, 0.0050, 0.9817) | < 0.0001 (< 0.0001, 1.0000, 0.8346) |
| 1513 | < 0.0001 (0.6993, 0.0093, 0.9994) | 0.4965 (1.0000, 0.6900, 0.9635) |
| 1514 | 0.4048 (< 0.0001, 0.1438, 0.9608) | < 0.0001 (0.6833, 1.0000, 0.4916) |
| 1515 | 1.0000 (< 0.0001, 1.0000, 0.9552) | < 0.0001 (1.0000, 1.0000, 0.9960) |
| 1516 | 0.6337 (0.0390, 0.2427, 0.7357) | < 0.0001 (1.0000, 1.0000, 0.9493) |
| 1517 | < 0.0001 (0.6746, 0.0073, 0.9894) | < 0.0001 (< 0.0001, 1.0000, 0.9644) |
| 1518 | 1.2511 (0.4444, 0.3543, 0.9951) | < 0.0001 (1.0000, 1.0000, 0.9862) |
| 1519 | 0.9510 (0.9994, 0.4651, 0.5453) | < 0.0001 (1.0000, 1.0000, 0.9864) |
| 1520 | < 0.0001 (0.9427, 0.0060, 0.9996) | < 0.0001 (< 0.0001, 1.0000, 0.9185) |
| 1521 | 1.0000 (0.6911, 1.0000, 0.9343) | < 0.0001 (< 0.0001, 1.0000, 0.8642) |
| 1522 | 1.0000 (0.0116, 1.0000, 0.8612) | < 0.0001 (1.0000, 1.0000, 0.9899) |
| 1523 | < 0.0001 (0.5084, 0.0050, 0.9991) | 1.0006 (1.0000, 0.4738, 0.9935) |
| 1524 | < 0.0001 (0.4444, 0.0050, 0.9996) | < 0.0001 (0.4667, 1.0000, 0.3354) |
| 1525 | < 0.0001 (0.6988, 0.0050, 0.9874) | 0.3333 (1.0000, 0.9803, 0.9936) |
| 1526 | < 0.0001 (0.6810, 0.0056, 0.9971) | 0.5003 (0.9803, 0.6883, 0.8283) |
| 1527 | < 0.0001 (0.7248, 0.0083, 1.0000) | < 0.0001 (0.6831, 1.0000, 0.5041) |
| 1528 | < 0.0001 (0.0586, 0.0142, 0.9991) | < 0.0001 (1.0000, 1.0000, 0.9356) |
| 1529 | 1.0000 (0.7130, 1.0000, 0.9411) | 0.7996 (1.0000, 0.8026, 0.9674) |
| 1530 | < 0.0001 (0.1313, 0.0060, 1.0000) | < 0.0001 (0.0228, 1.0000, 0.6585) |
| 1531 | 1.0000 (0.3527, 1.0000, 0.9870) | < 0.0001 (< 0.0001, 1.0000, 0.9874) |
| 1532 | 0.3013 (0.0261, 0.0674, 0.9980) | < 0.0001 (1.0000, 1.0000, 0.9677) |
| 1533 | < 0.0001 (0.0879, 0.0085, 0.9759) | < 0.0001 (1.0000, 1.0000, 0.9902) |
| 1534 | 1.0000 (0.0390, 1.0000, 0.9343) | < 0.0001 (1.0000, 1.0000, 0.9696) |
| 1535 | < 0.0001 (0.4444, 0.0425, 0.9973) | < 0.0001 (1.0000, 1.0000, 0.9864) |
| 1536 | 0.3994 (0.2690, 0.1396, 0.9035) | 0.4149 (1.0000, 0.9140, 1.0000) |
| 1537 | < 0.0001 (0.1318, 0.0056, 0.9984) | < 0.0001 (0.0209, 1.0000, 0.7329) |
| 1538 | 0.2786 (0.6448, 0.0528, 0.9280) | < 0.0001 (1.0000, 1.0000, 0.9857) |
| 1539 | < 0.0001 (0.5084, 0.0142, 0.9966) | < 0.0001 (1.0000, 1.0000, 0.9677) |
| 1540 | < 0.0001 (0.9096, 0.0343, 0.9994) | < 0.0001 (1.0000, 1.0000, 0.9869) |
| 1541 | 0.3186 (0.9918, 0.0793, 0.7890) | < 0.0001 (< 0.0001, 1.0000, 0.8609) |
| 1542 | < 0.0001 (0.8002, 0.0060, 0.9216) | 0.3703 (< 0.0001, 0.9563, 0.8642) |
| 1543 | 0.6377 (0.0116, 0.2459, 0.9789) | < 0.0001 (0.9856, 1.0000, 0.8998) |
| 1544 | < 0.0001 (0.4444, 0.0056, 0.9971) | < 0.0001 (1.0000, 1.0000, 0.9697) |
| 1545 | < 0.0001 (0.6667, 0.0083, 0.9987) | < 0.0001 (1.0000, 1.0000, 0.9997) |
| 1546 | 1.0000 (0.5084, 1.0000, 0.9870) | < 0.0001 (< 0.0001, 1.0000, 0.9185) |
| 1547 | 0.3480 (0.5084, 0.1006, 0.5069) | < 0.0001 (< 0.0001, 1.0000, 0.7847) |
| 1548 | 1.0000 (0.4601, 1.0000, 0.9351) | < 0.0001 (1.0000, 1.0000, 0.9959) |
| 1549 | < 0.0001 (0.6667, 0.0056, 0.9913) | < 0.0001 (1.0000, 1.0000, 0.9855) |
| 1550 | < 0.0001 (0.0339, 0.0083, 0.9999) | 1.0000 (1.0000, 0.4444, 0.9800) |
| 1551 | < 0.0001 (0.6950, 0.0117, 1.0000) | < 0.0001 (0.4444, 1.0000, 0.3597) |
| 1552 | 1.0000 (0.6993, 1.0000, 0.8897) | < 0.0001 (1.0000, 1.0000, 0.9562) |
| 1553 | 1.0000 (0.0116, 1.0000, 0.8886) | < 0.0001 (1.0000, 1.0000, 0.9649) |
| 1554 | < 0.0001 (< 0.0001, 0.0126, 0.9955) | < 0.0001 (1.0000, 1.0000, 0.9987) |
| 1555 | 1.0000 (< 0.0001, 1.0000, 0.9798) | < 0.0001 (< 0.0001, 1.0000, 0.9273) |
| 1556 | < 0.0001 (0.4445, 0.0099, 1.0000) | < 0.0001 (1.0000, 1.0000, 0.9870) |
| 1557 | 1.0000 (0.0390, 1.0000, 0.9490) | < 0.0001 (1.0000, 1.0000, 0.9904) |
| 1558 | < 0.0001 (0.5084, 0.0050, 0.9989) | < 0.0001 (1.0000, 1.0000, 0.9901) |
| 1559 | 1.0000 (0.2963, 1.0000, 0.9379) | < 0.0001 (< 0.0001, 1.0000, 0.9595) |
| 1560 | < 0.0001 (0.3625, 0.0060, 0.9996) | < 0.0001 (< 0.0001, 1.0000, 0.9185) |
| 1561 | < 0.0001 (0.2964, 0.0061, 0.9867) | < 0.0001 (< 0.0001, 1.0000, 0.7613) |
| 1562 | < 0.0001 (0.7066, 0.0061, 0.9807) | 0.0944 (1.0000, 0.9989, 0.9881) |
| 1563 | < 0.0001 (0.4445, 0.0397, 0.9982) | < 0.0001 (0.9925, 1.0000, 0.8319) |
| 1564 | 1.0000 (< 0.0001, 1.0000, 0.9870) | < 0.0001 (1.0000, 1.0000, 0.9124) |
| 1565 | < 0.0001 (0.1843, 0.0037, 0.9914) | < 0.0001 (< 0.0001, 1.0000, 0.7207) |
| 1566 | 0.3173 (< 0.0001, 0.0784, 0.6459) | < 0.0001 (1.0000, 1.0000, 0.9864) |
| 1567 | < 0.0001 (0.9176, 0.0073, 0.9999) | < 0.0001 (1.0000, 1.0000, 0.9596) |
| 1568 | 1.0000 (0.0174, 1.0000, 0.8462) | < 0.0001 (< 0.0001, 1.0000, 0.8753) |
| 1569 | < 0.0001 (< 0.0001, 0.0085, 1.0000) | < 0.0001 (1.0000, 1.0000, 0.9898) |
| 1570 | < 0.0001 (0.2243, 0.0425, 0.9944) | < 0.0001 (1.0000, 1.0000, 0.9453) |
| 1571 | < 0.0001 (0.1318, 0.0065, 0.9999) | < 0.0001 (1.0000, 1.0000, 0.9351) |
| 1572 | 1.0000 (0.4444, 1.0000, 0.9552) | < 0.0001 (1.0000, 1.0000, 0.9230) |
| 1573 | 0.8339 (0.5084, 0.3956, 0.8104) | < 0.0001 (1.0000, 1.0000, 0.9649) |
| 1574 | 1.0000 (0.0878, 1.0000, 0.9798) | < 0.0001 (< 0.0001, 1.0000, 0.8318) |
| 1575 | < 0.0001 (< 0.0001, 0.0056, 0.9971) | 0.3333 (1.0000, 1.0000, 0.9991) |
| 1576 | < 0.0001 (0.0877, 0.0037, 0.9695) | < 0.0001 (1.0000, 1.0000, 0.7836) |
| 1577 | < 0.0001 (0.5084, 0.0083, 1.0000) | 0.1182 (1.0000, 0.9966, 0.9935) |
| 1578 | < 0.0001 (0.0752, 0.0060, 0.9969) | < 0.0001 (0.9978, 1.0000, 0.8999) |
| 1579 | 0.6720 (0.4447, 0.2737, 0.7140) | 0.5092 (1.0000, 0.6626, 0.9350) |
| 1580 | < 0.0001 (0.7130, 0.0065, 0.9981) | < 0.0001 (0.6532, 1.0000, 0.6540) |
| 1581 | < 0.0001 (0.4682, 0.0313, 0.9863) | 1.4027 (1.0000, 0.8164, 0.9934) |
| 1582 | < 0.0001 (0.6784, 0.0126, 1.0000) | < 0.0001 (0.8999, 1.0000, 0.3357) |
| 1583 | < 0.0001 (0.6667, 0.0087, 0.9999) | < 0.0001 (1.0000, 1.0000, 0.9770) |
| 1584 | < 0.0001 (< 0.0001, 0.0142, 0.9999) | < 0.0001 (1.0000, 1.0000, 0.9866) |
| 1585 | < 0.0001 (0.6950, 0.0060, 0.9854) | < 0.0001 (< 0.0001, 1.0000, 0.9595) |
| 1586 | < 0.0001 (0.7130, 0.0060, 0.9947) | < 0.0001 (1.0000, 1.0000, 0.9960) |
| 1587 | < 0.0001 (0.1761, 0.0093, 0.9977) | < 0.0001 (1.0000, 1.0000, 0.9793) |
| 1588 | 1.0000 (0.7065, 1.0000, 0.9798) | < 0.0001 (< 0.0001, 1.0000, 0.7060) |
| 1589 | 1.0000 (0.0476, 1.0000, 0.9379) | < 0.0001 (< 0.0001, 1.0000, 0.8642) |
| 1590 | < 0.0001 (< 0.0001, 0.0397, 0.9991) | < 0.0001 (< 0.0001, 1.0000, 0.8858) |
| 1591 | < 0.0001 (0.1165, 0.0343, 0.9817) | < 0.0001 (< 0.0001, 1.0000, 0.9644) |
| 1592 | 0.3693 (0.0148, 0.1165, 0.9435) | < 0.0001 (1.0000, 1.0000, 0.9635) |
| 1593 | 0.6340 (0.0279, 0.2429, 0.8483) | < 0.0001 (1.0000, 1.0000, 0.9878) |
| 1594 | 1.0000 (0.6667, 1.0000, 0.9798) | 0.4939 (1.0000, 0.8907, 0.9901) |
| 1595 | < 0.0001 (0.0668, 0.0053, 0.9990) | < 0.0001 (0.9013, 1.0000, 0.7481) |
| 1596 | < 0.0001 (0.6667, 0.0056, 0.9884) | 1.0000 (< 0.0001, 0.6804, 0.9266) |
| 1597 | 0.6030 (0.4444, 0.2905, 0.8961) | 1.0419 (0.6804, 0.7284, 0.4455) |
| 1598 | < 0.0001 (0.0668, 0.0065, 0.9856) | < 0.0001 (0.7363, 1.0000, 0.5419) |
| 1599 | 0.3358 (< 0.0001, 0.0916, 0.7046) | 0.8231 (1.0000, 0.7946, 0.9992) |
| 1600 | < 0.0001 (0.1951, 0.0083, 1.0000) | < 0.0001 (0.7646, 1.0000, 0.5417) |
| 1601 | < 0.0001 (0.0762, 0.0343, 0.9817) | 0.4964 (< 0.0001, 0.6900, 0.8096) |
| 1602 | 1.0000 (0.6667, 1.0000, 0.9870) | < 0.0001 (0.6833, 1.0000, 0.4912) |
| 1603 | < 0.0001 (0.6667, 0.0060, 0.9991) | < 0.0001 (< 0.0001, 1.0000, 0.9644) |
| 1604 | < 0.0001 (1.0000, 0.0083, 0.9987) | < 0.0001 (1.0000, 1.0000, 0.9428) |
| 1605 | 1.0000 (0.1317, 1.0000, 0.9525) | < 0.0001 (< 0.0001, 1.0000, 0.8858) |
| 1606 | 1.0000 (< 0.0001, 1.0000, 0.9351) | < 0.0001 (1.0000, 1.0000, 0.9677) |
| 1607 | < 0.0001 (0.0007, 0.0093, 0.9962) | 0.7994 (1.0000, 0.8026, 0.9678) |
| 1608 | < 0.0001 (< 0.0001, 0.0056, 0.9995) | < 0.0001 (0.8025, 1.0000, 0.6585) |
| 1609 | 0.9509 (< 0.0001, 0.4650, 0.5604) | < 0.0001 (1.0000, 1.0000, 0.9961) |
| 1610 | < 0.0001 (0.1319, 0.0053, 0.9997) | 0.1248 (1.0000, 0.9959, 0.9562) |
| 1611 | 2.0946 (0.1165, 0.0536, 0.9615) | < 0.0001 (0.9960, 1.0000, 0.9908) |
| 1612 | 0.3166 (0.3624, 0.0779, 0.8958) | < 0.0001 (1.0000, 1.0000, 0.9952) |
| 1613 | < 0.0001 (0.0242, 0.0053, 0.9958) | 0.1252 (1.0000, 0.9959, 0.9635) |
| 1614 | < 0.0001 (0.4444, 0.0083, 0.9982) | < 0.0001 (0.9959, 1.0000, 0.9726) |
| 1615 | 0.6032 (0.0665, 0.2906, 0.8959) | < 0.0001 (< 0.0001, 1.0000, 0.7731) |
| 1616 | < 0.0001 (0.0476, 0.0050, 0.9999) | < 0.0001 (1.0000, 1.0000, 0.9935) |
| 1617 | < 0.0001 (0.4992, 0.0060, 0.9896) | < 0.0001 (1.0000, 1.0000, 0.9872) |
| 1618 | < 0.0001 (0.7130, 0.0093, 0.9993) | < 0.0001 (1.0000, 1.0000, 0.9676) |
| 1619 | < 0.0001 (0.2392, 0.0056, 0.9884) | < 0.0001 (1.0000, 1.0000, 0.9994) |
| 1620 | < 0.0001 (0.6882, 0.0083, 0.9948) | < 0.0001 (< 0.0001, 1.0000, 0.8702) |
| 1621 | < 0.0001 (0.9874, 0.0117, 1.0000) | < 0.0001 (< 0.0001, 1.0000, 0.9127) |
| 1622 | 0.3167 (0.5084, 0.0780, 0.9998) | 0.9382 (1.0000, 0.7592, 0.9737) |
| 1623 | < 0.0001 (0.0935, 0.0050, 0.9989) | 0.4684 (0.7526, 0.8982, 0.6011) |
| 1624 | < 0.0001 (0.0476, 0.0093, 0.9989) | < 0.0001 (0.9013, 1.0000, 0.7093) |
| 1625 | < 0.0001 (1.0000, 0.0073, 0.9961) | < 0.0001 (1.0000, 1.0000, 0.9998) |
| 1626 | 1.0000 (0.8889, 1.0000, 0.9216) | < 0.0001 (1.0000, 1.0000, 0.9901) |
| 1627 | 1.0000 (0.1317, 1.0000, 0.9351) | < 0.0001 (1.0000, 1.0000, 0.9793) |
| 1628 | 0.9523 (0.6215, 0.4660, 0.9969) | < 0.0001 (1.0000, 1.0000, 0.9698) |
| 1629 | < 0.0001 (0.2965, 0.0056, 0.9884) | 0.4147 (1.0000, 0.9214, 0.9899) |
| 1630 | < 0.0001 (0.6667, 0.0061, 1.0000) | < 0.0001 (0.9040, 1.0000, 0.7386) |
| 1631 | < 0.0001 (0.0879, 0.0083, 0.9987) | < 0.0001 (1.0000, 1.0000, 0.9670) |
| 1632 | 1.0000 (0.2585, 1.0000, 0.8479) | < 0.0001 (1.0000, 1.0000, 0.9992) |
| 1633 | < 0.0001 (0.4774, 0.0397, 0.9991) | < 0.0001 (< 0.0001, 1.0000, 0.9595) |
| 1634 | < 0.0001 (0.0032, 0.0065, 0.9952) | < 0.0001 (1.0000, 1.0000, 0.9936) |
| 1635 | < 0.0001 (< 0.0001, 0.0117, 0.9999) | < 0.0001 (< 0.0001, 1.0000, 0.8768) |
| 1636 | 1.0843 (0.2584, 0.4450, 0.3714) | < 0.0001 (1.0000, 1.0000, 0.9961) |
| 1637 | < 0.0001 (0.5084, 0.0050, 0.9980) | < 0.0001 (1.0000, 1.0000, 0.9952) |
| 1638 | < 0.0001 (0.1975, 0.0060, 0.9947) | < 0.0001 (< 0.0001, 1.0000, 0.8768) |
| 1639 | < 0.0001 (0.6667, 0.0050, 1.0000) | < 0.0001 (< 0.0001, 1.0000, 0.9127) |
| 1640 | 1.0000 (0.4736, 1.0000, 0.9536) | < 0.0001 (1.0000, 1.0000, 0.9677) |
| 1641 | < 0.0001 (0.5846, 0.0056, 0.9991) | < 0.0001 (< 0.0001, 1.0000, 0.9185) |
| 1642 | < 0.0001 (0.7130, 0.0131, 1.0000) | < 0.0001 (1.0000, 1.0000, 0.9947) |
| 1643 | < 0.0001 (< 0.0001, 0.0093, 0.9993) | < 0.0001 (1.0000, 1.0000, 0.9985) |
| 1644 | 1.0000 (0.6667, 1.0000, 0.9816) | < 0.0001 (1.0000, 1.0000, 0.9901) |
| 1645 | < 0.0001 (0.4775, 0.0083, 0.9948) | < 0.0001 (< 0.0001, 1.0000, 0.8366) |
| 1646 | < 0.0001 (0.1975, 0.0065, 0.9733) | < 0.0001 (1.0000, 1.0000, 0.9908) |
| 1647 | < 0.0001 (0.6784, 0.0060, 0.9966) | < 0.0001 (< 0.0001, 1.0000, 0.7791) |
| 1648 | 1.5358 (0.2964, 0.1855, 0.9416) | < 0.0001 (1.0000, 1.0000, 0.9864) |
| 1649 | < 0.0001 (0.0666, 0.0056, 0.9583) | < 0.0001 (< 0.0001, 1.0000, 0.7798) |
| 1650 | < 0.0001 (0.7407, 0.0065, 0.9651) | < 0.0001 (1.0000, 1.0000, 0.9836) |
| 1651 | < 0.0001 (0.5084, 0.0083, 0.9996) | < 0.0001 (1.0000, 1.0000, 0.9956) |
| 1652 | < 0.0001 (0.8532, 0.0060, 0.9808) | < 0.0001 (< 0.0001, 1.0000, 0.9644) |
| 1653 | 1.0000 (0.9576, 1.0000, 0.9343) | < 0.0001 (< 0.0001, 1.0000, 0.7798) |
| 1654 | < 0.0001 (0.2963, 0.0126, 0.9998) | 0.1667 (1.0000, 0.9877, 0.9650) |
| 1655 | < 0.0001 (0.3621, 0.0142, 0.9985) | < 0.0001 (0.9877, 1.0000, 0.9687) |
| 1656 | < 0.0001 (0.7130, 0.0056, 0.9696) | < 0.0001 (1.0000, 1.0000, 0.9952) |
| 1657 | < 0.0001 (0.1975, 0.0053, 0.9676) | < 0.0001 (1.0000, 1.0000, 0.9961) |
| 1658 | < 0.0001 (0.0829, 0.0060, 0.9854) | < 0.0001 (< 0.0001, 1.0000, 0.8609) |
| 1659 | < 0.0001 (0.6592, 0.0083, 0.9950) | < 0.0001 (1.0000, 1.0000, 0.9901) |
| 1660 | 0.2202 (0.5084, 0.0221, 0.9971) | < 0.0001 (1.0000, 1.0000, 0.9935) |
| 1661 | 0.2226 (1.0000, 0.0231, 0.9358) | < 0.0001 (1.0000, 1.0000, 0.9961) |
| 1662 | < 0.0001 (< 0.0001, 0.0093, 0.9994) | < 0.0001 (< 0.0001, 1.0000, 0.9020) |
| 1663 | 0.6133 (0.4446, 0.2260, 0.9801) | < 0.0001 (1.0000, 1.0000, 0.9934) |
| 1664 | < 0.0001 (0.9983, 0.0056, 0.9995) | < 0.0001 (1.0000, 1.0000, 0.9494) |
| 1665 | 0.4043 (< 0.0001, 0.1434, 0.8214) | < 0.0001 (1.0000, 1.0000, 0.9987) |
| 1666 | < 0.0001 (0.9906, 0.0142, 0.9991) | < 0.0001 (1.0000, 1.0000, 0.9940) |
| 1667 | 1.9871 (< 0.0001, 0.0324, 0.2653) | < 0.0001 (< 0.0001, 1.0000, 0.9099) |
| 1668 | < 0.0001 (0.1838, 0.0037, 0.9983) | 0.5000 (1.0000, 0.8889, 0.9960) |
| 1669 | < 0.0001 (0.2117, 0.0061, 0.9990) | < 0.0001 (0.8889, 1.0000, 0.7936) |
| 1670 | 1.0000 (0.9622, 1.0000, 0.9816) | 0.4835 (1.0000, 0.7085, 0.9709) |
| 1671 | < 0.0001 (0.9832, 0.0083, 0.9987) | < 0.0001 (0.6845, 1.0000, 0.4793) |
| 1672 | < 0.0001 (0.3109, 0.0343, 0.9986) | < 0.0001 (1.0000, 1.0000, 1.0000) |
| 1673 | 1.0000 (< 0.0001, 1.0000, 0.7969) | < 0.0001 (< 0.0001, 1.0000, 0.7295) |
| 1674 | < 0.0001 (0.5084, 0.0073, 0.9885) | < 0.0001 (1.0000, 1.0000, 0.9649) |
| 1675 | 1.0000 (0.4448, 1.0000, 0.9411) | < 0.0001 (1.0000, 1.0000, 0.9225) |
| 1676 | 1.0000 (1.0000, 1.0000, 0.9870) | < 0.0001 (1.0000, 1.0000, 0.9670) |
| 1677 | < 0.0001 (< 0.0001, 0.0065, 0.9933) | 0.9999 (1.0000, 0.4445, 0.9596) |
| 1678 | < 0.0001 (0.4992, 0.0073, 1.0000) | < 0.0001 (0.4445, 1.0000, 0.2392) |
| 1679 | < 0.0001 (0.0051, 0.0093, 0.9967) | < 0.0001 (< 0.0001, 1.0000, 0.7207) |
| 1680 | 0.9521 (0.3526, 0.4659, 0.7051) | < 0.0001 (1.0000, 1.0000, 0.9981) |
| 1681 | 1.0000 (0.5084, 1.0000, 0.9552) | < 0.0001 (< 0.0001, 1.0000, 0.9644) |
| 1682 | 1.9178 (< 0.0001, 0.0753, 0.5818) | < 0.0001 (1.0000, 1.0000, 0.9928) |
| 1683 | 1.5944 (0.3349, 0.1668, 0.2370) | < 0.0001 (1.0000, 1.0000, 0.9980) |
| 1684 | < 0.0001 (0.5084, 0.0117, 1.0000) | < 0.0001 (1.0000, 1.0000, 0.9993) |
| 1685 | 2.6970 (0.2490, 0.0081, 0.0929) | < 0.0001 (1.0000, 1.0000, 0.9694) |
| 1686 | 0.3611 (0.0585, 0.1103, 0.5965) | < 0.0001 (1.0000, 1.0000, 0.9977) |
| 1687 | 0.6720 (0.7889, 0.2737, 0.4799) | < 0.0001 (1.0000, 1.0000, 0.9695) |
| 1688 | 1.0000 (0.0116, 1.0000, 0.9870) | < 0.0001 (0.0222, 1.0000, 0.9573) |
| 1689 | < 0.0001 (< 0.0001, 0.0425, 0.9944) | < 0.0001 (< 0.0001, 1.0000, 0.8858) |
| 1690 | 1.0000 (0.2968, 1.0000, 0.9411) | < 0.0001 (1.0000, 1.0000, 0.9427) |
| 1691 | < 0.0001 (0.1243, 0.0060, 0.9896) | < 0.0001 (< 0.0001, 1.0000, 0.9644) |
| 1692 | 0.3166 (0.9993, 0.0779, 0.9481) | < 0.0001 (1.0000, 1.0000, 0.9994) |
| 1693 | 3.0264 (0.4830, 0.0183, 0.1318) | < 0.0001 (1.0000, 1.0000, 0.9994) |
| 1694 | < 0.0001 (0.9376, 0.0060, 0.9999) | < 0.0001 (1.0000, 1.0000, 0.9841) |
| 1695 | < 0.0001 (0.7130, 0.0050, 0.9941) | < 0.0001 (1.0000, 1.0000, 0.9737) |
| 1696 | 0.3477 (0.2582, 0.1004, 0.9985) | < 0.0001 (< 0.0001, 1.0000, 0.9185) |
| 1697 | < 0.0001 (0.2963, 0.0424, 0.9986) | < 0.0001 (1.0000, 1.0000, 0.9914) |
| 1698 | < 0.0001 (0.1575, 0.0065, 0.9933) | < 0.0001 (1.0000, 1.0000, 0.9970) |
| 1699 | 1.2004 (0.3349, 0.3795, 0.9936) | < 0.0001 (1.0000, 1.0000, 0.9846) |
| 1700 | < 0.0001 (0.2333, 0.0099, 0.9932) | < 0.0001 (1.0000, 1.0000, 0.9357) |
| 1701 | < 0.0001 (0.4445, 0.0093, 0.9994) | < 0.0001 (1.0000, 1.0000, 0.9427) |
| 1702 | < 0.0001 (0.1308, 0.0056, 0.9984) | < 0.0001 (1.0000, 1.0000, 1.0000) |
| 1703 | < 0.0001 (0.0668, 0.0061, 0.9867) | < 0.0001 (< 0.0001, 1.0000, 0.9185) |
| 1704 | < 0.0001 (0.1979, 0.0056, 1.0000) | < 0.0001 (< 0.0001, 1.0000, 0.9644) |
| 1705 | < 0.0001 (0.6667, *0.0060*, 0.9966) | < 0.0001 (1.0000, 1.0000, 0.9172) |
| 1706 | < 0.0001 (0.7776, *0.0050*, 0.9951) | < 0.0001 (1.0000, 1.0000, 0.9878) |
| 1707 | 0.2862 (< *0.0001*, 0.0576, 0.8978) | < 0.0001 (1.0000, 1.0000, 0.9836) |
| 1708 | 0.8598 (0.6944, 0.3943, 0.7111) | < 0.0001 (1.0000, 1.0000, 0.9798) |
| 1709 | 0.3170 (0.9993, 0.0782, 0.9290) | < 0.0001 (1.0000, 1.0000, 0.9993) |
| 1710 | < 0.0001 (< *0.0001*, 0.0424, 1.0000) | < 0.0001 (1.0000, 1.0000, 0.9695) |
| 1711 | < 0.0001 (0.4447, 0.0093, 0.9967) | < 0.0001 (1.0000, 1.0000, 0.9307) |
| 1712 | < 0.0001 (0.1759, 0.0343, 0.9925) | < 0.0001 (1.0000, 1.0000, 0.9992) |
| 1713 | < 0.0001 (0.1667, 0.0060, 0.9957) | < 0.0001 (1.0000, 1.0000, 0.9537) |
| 1714 | < 0.0001 (0.2584, 0.0060, 0.9985) | < 0.0001 (1.0000, 1.0000, 0.9949) |
| 1715 | 0.6335 (0.5084, 0.2426, 0.8384) | < 0.0001 (1.0000, 1.0000, 0.9598) |
| 1716 | < 0.0001 (< 0.0001, 0.0073, 0.9894) | < 0.0001 (1.0000, 1.0000, 0.9848) |
| 1717 | < 0.0001 (0.3625, 0.0061, 1.0000) | < 0.0001 (1.0000, 1.0000, 0.9848) |
| 1718 | 0.2200 (< 0.0001, 0.0220, 0.9126) | < 0.0001 (1.0000, 1.0000, 0.9710) |
| 1719 | < 0.0001 (0.7130, 0.0053, 0.9997) | < 0.0001 (1.0000, 1.0000, 0.9987) |
| 1720 | 1.0000 (< 0.0001, 1.0000, 0.9342) | < 0.0001 (1.0000, 1.0000, 0.9584) |
| 1721 | < 0.0001 (0.1320, 0.0044, 0.9999) | < 0.0001 (1.0000, 1.0000, 0.9976) |
| 1722 | < 0.0001 (0.1977, 0.0424, 0.9996) | < 0.0001 (1.0000, 1.0000, 0.9992) |
| 1723 | 1.0000 (0.0392, 1.0000, 0.9342) | 0.5003 (< 0.0001, 0.6883, 0.9127) |
| 1724 | 1.0000 (0.9411, 1.0000, 0.9490) | 0.1667 (0.6831, 0.9877, 0.5041) |
| 1725 | < 0.0001 (< 0.0001, 0.0061, 0.9960) | < 0.0001 (0.9877, 1.0000, 0.9142) |
| 1726 | 1.0000 (0.5084, 1.0000, 0.9525) | < 0.0001 (< 0.0001, 1.0000, 0.8711) |
| 1727 | < 0.0001 (0.0171, 0.0425, 0.9999) | < 0.0001 (1.0000, 1.0000, 0.9947) |
| 1728 | < 0.0001 (< 0.0001, 0.0061, 0.9997) | < 0.0001 (< 0.0001, 1.0000, 0.8753) |
| 1729 | < 0.0001 (< 0.0001, 0.0093, 0.9999) | < 0.0001 (1.0000, 1.0000, 0.9670) |
| 1730 | < 0.0001 (0.7065, 0.0050, 0.9963) | < 0.0001 (< 0.0001, 1.0000, 0.9188) |
| 1731 | < 0.0001 (0.6944, 0.0041, 0.9989) | < 0.0001 (1.0000, 1.0000, 0.9988) |
| 1732 | < 0.0001 (0.4444, 0.0083, 0.9773) | 0.0535 (< 0.0001, 0.9974, 0.9099) |
| 1733 | < 0.0001 (< 0.0001, 0.0083, 0.9948) | < 0.0001 (0.9995, 1.0000, 0.9387) |
| 1734 | 0.3186 (0.1842, 0.0793, 0.5366) | < 0.0001 (1.0000, 1.0000, 1.0000) |
| 1735 | < 0.0001 (0.0936, 0.0061, 0.9937) | < 0.0001 (< 0.0001, 1.0000, 0.9644) |
| 1736 | < 0.0001 (0.7130, 0.0073, 0.9998) | < 0.0001 (1.0000, 1.0000, 0.9987) |
| 1737 | 1.0000 (0.4602, 1.0000, 0.9870) | < 0.0001 (< 0.0001, 1.0000, 0.8324) |
| 1738 | < 0.0001 (0.3260, 0.0061, 0.9807) | < 0.0001 (1.0000, 1.0000, 0.9899) |
| 1739 | < 0.0001 (0.4602, 0.0073, 0.9885) | < 0.0001 (1.0000, 1.0000, 0.9901) |
| 1740 | 0.3186 (0.8285, 0.0793, 0.9974) | < 0.0001 (1.0000, 1.0000, 0.9974) |
| 1741 | < 0.0001 (0.4883, 0.0424, 0.9993) | 0.4999 (1.0000, 0.8889, 0.9869) |
| 1742 | < 0.0001 (0.5083, 0.0044, 0.9995) | < 0.0001 (0.8889, 1.0000, 0.6962) |
| 1743 | 1.0000 (0.3577, 1.0000, 0.9216) | < 0.0001 (< 0.0001, 1.0000, 0.9273) |
| 1744 | < 0.0001 (0.0051, 0.0425, 0.9996) | < 0.0001 (< 0.0001, 1.0000, 0.7207) |
| 1745 | < 0.0001 (0.4445, 0.0093, 0.9977) | < 0.0001 (1.0000, 1.0000, 0.9901) |
| 1746 | < 0.0001 (0.6784, 0.0060, 0.9999) | < 0.0001 (1.0000, 1.0000, 0.9993) |
| 1747 | 1.0000 (< 0.0001, 1.0000, 0.9436) | < 0.0001 (< 0.0001, 1.0000, 0.9266) |
| 1748 | < 0.0001 (< 0.0001, 0.0142, 0.9998) | < 0.0001 (1.0000, 1.0000, 0.9956) |
| 1749 | < 0.0001 (0.1762, 0.0343, 0.9925) | < 0.0001 (1.0000, 1.0000, 0.9788) |
| 1750 | < 0.0001 (< 0.0001, 0.0050, 1.0000) | 0.5092 (1.0000, 0.8862, 0.9839) |
| 1751 | < 0.0001 (0.7130, 0.0107, 0.9975) | < 0.0001 (0.8797, 1.0000, 0.8742) |
| 1752 | < 0.0001 (0.0240, 0.0061, 0.9948) | < 0.0001 (1.0000, 1.0000, 0.9901) |
| 1753 | < 0.0001 (< 0.0001, 0.0083, 0.9996) | 1.0000 (1.0000, 0.7407, 0.9596) |
| 1754 | 1.8157 (0.0877, 0.1127, 0.4022) | 0.4732 (0.7408, 0.8968, 0.6424) |
| 1755 | < 0.0001 (0.2964, 0.0045, 0.9890) | 0.7605 (0.9134, 0.7615, 0.5974) |
| 1756 | 0.3360 (0.0389, 0.0918, 0.8794) | < 0.0001 (0.7164, 1.0000, 0.8026) |
| 1757 | < 0.0001 (0.4883, 0.0099, 1.0000) | < 0.0001 (< 0.0001, 1.0000, 0.8346) |
| 1758 | 0.3065 (0.4444, 0.0709, 0.4453) | < 0.0001 (1.0000, 1.0000, 0.9563) |
| 1759 | < 0.0001 (0.9994, 0.0060, 0.9854) | < 0.0001 (< 0.0001, 1.0000, 0.8711) |
| 1760 | < 0.0001 (0.2963, 0.0053, 0.9675) | < 0.0001 (1.0000, 1.0000, 0.9961) |
| 1761 | < 0.0001 (0.0008, 0.0073, 0.9894) | < 0.0001 (1.0000, 1.0000, 0.9994) |
| 1762 | < 0.0001 (0.1117, 0.0060, 0.9808) | < 0.0001 (< 0.0001, 1.0000, 0.7207) |
| 1763 | 1.3952 (< 0.0001, 0.2642, 0.0885) | < 0.0001 (< 0.0001, 1.0000, 0.8161) |
| 1764 | < 0.0001 (0.0394, 0.0065, 0.9651) | < 0.0001 (1.0000, 1.0000, 0.9537) |
| 1765 | < 0.0001 (0.9548, 0.0083, 1.0000) | < 0.0001 (< 0.0001, 1.0000, 0.9386) |
| 1766 | 1.0000 (0.6667, 1.0000, 0.9870) | < 0.0001 (< 0.0001, 1.0000, 0.7700) |
| 1767 | < 0.0001 (0.7130, 0.0087, 0.9995) | < 0.0001 (< 0.0001, 1.0000, 0.8127) |
| 1768 | < 0.0001 (0.9994, 0.0099, 1.0000) | < 0.0001 (< 0.0001, 1.0000, 0.8609) |
| 1769 | < 0.0001 (< 0.0001, 0.0061, 1.0000) | < 0.0001 (1.0000, 1.0000, 0.9998) |
| 1770 | < 0.0001 (1.0000, 0.0050, 0.9875) | < 0.0001 (1.0000, 1.0000, 0.9868) |
| 1771 | < 0.0001 (< 0.0001, 0.0126, 1.0000) | < 0.0001 (< 0.0001, 1.0000, 0.9386) |
| 1772 | < 0.0001 (0.9631, 0.0050, 0.9874) | < 0.0001 (1.0000, 1.0000, 0.9993) |
| 1773 | < 0.0001 (0.0174, 0.0099, 0.9984) | < 0.0001 (1.0000, 1.0000, 0.9958) |
| 1774 | 1.0000 (0.1317, 1.0000, 0.9411) | < 0.0001 (1.0000, 1.0000, 0.9868) |
| 1775 | < 0.0001 (0.3625, 0.0424, 0.9999) | 1.5000 (1.0000, 0.5926, 0.9584) |
| 1776 | 1.0000 (1.0000, 1.0000, 0.9870) | < 0.0001 (0.5927, 1.0000, 0.4222) |
| 1777 | < 0.0001 (0.2349, 0.0107, 1.0000) | < 0.0001 (< 0.0001, 1.0000, 0.8753) |
| 1778 | 0.3611 (0.1538, 0.1103, 0.9987) | < 0.0001 (1.0000, 1.0000, 0.9890) |
| 1779 | < 0.0001 (0.0023, 0.0099, 0.9984) | < 0.0001 (1.0000, 1.0000, 0.9964) |
| 1780 | < 0.0001 (0.0115, 0.0041, 0.9905) | < 0.0001 (1.0000, 1.0000, 0.9534) |
| 1781 | < 0.0001 (1.0000, 0.0083, 0.9948) | < 0.0001 (< 0.0001, 1.0000, 0.9386) |
| 1782 | < 0.0001 (< 0.0001, 0.0093, 0.9974) | < 0.0001 (1.0000, 1.0000, 0.9737) |
| 1783 | < 0.0001 (0.1977, 0.0053, 0.9999) | < 0.0001 (1.0000, 1.0000, 0.9961) |
| 1784 | < 0.0001 (0.1976, 0.0142, 0.9998) | < 0.0001 (1.0000, 1.0000, 0.9963) |
| 1785 | < 0.0001 (0.7130, 0.0053, 0.9905) | < 0.0001 (1.0000, 1.0000, 0.9716) |
| 1786 | < 0.0001 (0.1313, 0.0425, 0.9944) | < 0.0001 (1.0000, 1.0000, 1.0000) |
| 1787 | < 0.0001 (0.1040, 0.0117, 1.0000) | < 0.0001 (1.0000, 1.0000, 0.9890) |
| 1788 | 1.0000 (0.6602, 1.0000, 0.9525) | < 0.0001 (< 0.0001, 1.0000, 0.9644) |
| 1789 | 1.0000 (< 0.0001, 1.0000, 0.9870) | < 0.0001 (1.0000, 1.0000, 0.9890) |
| 1790 | < 0.0001 (0.7130, 0.0065, 0.9966) | < 0.0001 (1.0000, 1.0000, 0.9667) |
| 1791 | < 0.0001 (< 0.0001, 0.0073, 0.9998) | < 0.0001 (1.0000, 1.0000, 0.9878) |
| 1792 | < 0.0001 (0.2329, 0.0061, 0.9996) | < 0.0001 (1.0000, 1.0000, 0.9946) |
| 1793 | < 0.0001 (0.2688, 0.0099, 0.9975) | < 0.0001 (1.0000, 1.0000, 0.9890) |
| 1794 | < 0.0001 (0.4445, 0.0117, 1.0000) | < 0.0001 (1.0000, 1.0000, 0.9963) |
| 1795 | < 0.0001 (0.0667, 0.0050, 0.9963) | < 0.0001 (1.0000, 1.0000, 0.9976) |
| 1796 | < 0.0001 (0.4445, 0.0065, 0.9894) | < 0.0001 (1.0000, 1.0000, 0.9758) |
| 1797 | < 0.0001 (0.4231, 0.0093, 1.0000) | 0.1665 (< 0.0001, 0.9877, 0.8272) |
| 1798 | < 0.0001 (0.0751, 0.0061, 0.9937) | < 0.0001 (0.9822, 1.0000, 0.7976) |
| 1799 | < 0.0001 (1.0000, 0.0037, 0.9999) | < 0.0001 (1.0000, 1.0000, 0.9538) |
| 1800 | < 0.0001 (0.8889, 0.0060, 0.9937) | < 0.0001 (1.0000, 1.0000, 0.9867) |
| 1801 | < 0.0001 (0.4444, 0.0061, 0.9948) | 1.4614 (1.0000, 0.3534, 0.9696) |
| 1802 | < 0.0001 (< 0.0001, 0.0061, 0.9999) | < 0.0001 (0.3205, 1.0000, 0.2107) |
| 1803 | < 0.0001 (< 0.0001, 0.0044, 0.9891) | 0.2500 (1.0000, 0.9630, 0.9846) |
| 1804 | < 0.0001 (0.1615, 0.0056, 0.9997) | < 0.0001 (0.9630, 1.0000, 0.8515) |
| 1805 | < 0.0001 (0.8889, 0.0142, 0.9943) | < 0.0001 (1.0000, 1.0000, 0.9468) |
| 1806 | < 0.0001 (0.9332, 0.0060, 0.9999) | < 0.0001 (1.0000, 1.0000, 0.9914) |
| 1807 | < 0.0001 (0.0034, 0.0107, 1.0000) | < 0.0001 (1.0000, 1.0000, 0.9936) |
| 1808 | 1.0000 (0.3625, 1.0000, 0.9490) | < 0.0001 (1.0000, 1.0000, 0.9895) |
| 1809 | 0.6336 (< 0.0001, 0.2426, 0.7357) | < 0.0001 (< 0.0001, 1.0000, 0.8770) |
| 1810 | 0.3480 (0.9452, 0.1006, 0.9926) | < 0.0001 (0.0217, 1.0000, 0.9994) |
| 1811 | < 0.0001 (0.2879, 0.0073, 0.9894) | < 0.0001 (1.0000, 1.0000, 0.9998) |
| 1812 | < 0.0001 (0.9663, 0.0424, 0.9991) | 0.1250 (1.0000, 0.9959, 0.9986) |
| 1813 | < 0.0001 (0.0390, 0.0056, 0.9960) | < 0.0001 (0.9959, 1.0000, 0.9943) |
| 1814 | < 0.0001 (0.2963, 0.0073, 0.9987) | < 0.0001 (1.0000, 1.0000, 0.9670) |
| 1815 | < 0.0001 (0.3259, 0.0061, 0.9958) | < 0.0001 (< 0.0001, 1.0000, 0.8142) |
| 1816 | < 0.0001 (0.6784, 0.0061, 1.0000) | < 0.0001 (1.0000, 1.0000, 0.9901) |
| 1817 | < 0.0001 (0.1436, 0.0061, 0.9948) | < 0.0001 (< 0.0001, 1.0000, 0.8702) |
| 1818 | 0.6624 (0.6988, 0.2221, 0.9723) | < 0.0001 (1.0000, 1.0000, 0.9984) |
| 1819 | < 0.0001 (< 0.0001, 0.0117, 1.0000) | < 0.0001 (1.0000, 1.0000, 0.9696) |
| 1820 | 1.0000 (0.2333, 1.0000, 0.9870) | 0.4768 (1.0000, 0.6992, 0.9969) |
| 1821 | < 0.0001 (0.6667, 0.0050, 0.9999) | 0.1078 (0.7056, 0.9975, 0.3050) |
| 1822 | < 0.0001 (0.0023, 0.0061, 0.9960) | < 0.0001 (0.9998, 1.0000, 0.9563) |
| 1823 | < 0.0001 (< 0.0001, 0.0050, 0.9996) | < 0.0001 (< 0.0001, 1.0000, 0.9386) |
| 1824 | < 0.0001 (0.2963, 0.0093, 0.9967) | < 0.0001 (1.0000, 1.0000, 0.9306) |
| 1825 | < 0.0001 (0.1317, 0.0093, 0.9993) | < 0.0001 (1.0000, 1.0000, 0.9922) |
| 1826 | < 0.0001 (1.0000, 0.0056, 0.9991) | < 0.0001 (1.0000, 1.0000, 0.9969) |
| 1827 | 0.6029 (0.5084, 0.2175, 0.6870) | < 0.0001 (1.0000, 1.0000, 0.9961) |
| 1828 | 1.0000 (0.3527, 1.0000, 0.9536) | < 0.0001 (< 0.0001, 1.0000, 0.9127) |
| 1829 | < 0.0001 (1.0000, 0.0065, 0.9651) | < 0.0001 (< 0.0001, 1.0000, 0.9127) |
| 1830 | < 0.0001 (< 0.0001, 0.0093, 0.9993) | < 0.0001 (1.0000, 1.0000, 0.9855) |
| 1831 | < 0.0001 (0.4444, 0.0117, 0.9998) | < 0.0001 (< 0.0001, 1.0000, 0.9595) |
| 1832 | 1.0000 (0.5083, 1.0000, 0.9552) | < 0.0001 (1.0000, 1.0000, 0.9688) |
| 1833 | 0.3361 (1.0000, 0.0918, 0.9736) | < 0.0001 (1.0000, 1.0000, 0.9650) |
| 1834 | < 0.0001 (0.4444, 0.0107, 0.9907) | < 0.0001 (< 0.0001, 1.0000, 0.9644) |
| 1835 | < 0.0001 (0.6667, 0.0053, 0.9995) | < 0.0001 (1.0000, 1.0000, 0.9889) |
| 1836 | 0.4047 (0.8769, 0.1437, 0.9886) | < 0.0001 (1.0000, 1.0000, 0.9970) |
| 1837 | 0.3611 (1.0000, 0.1103, 0.9903) | < 0.0001 (1.0000, 1.0000, 0.9728) |
| 1838 | < 0.0001 (0.0438, 0.0060, 0.9937) | 0.0646 (1.0000, 0.9999, 1.0000) |
| 1839 | 1.1261 (0.0666, 0.4200, 0.6662) | < 0.0001 (0.9997, 1.0000, 0.9994) |
| 1840 | 0.3244 (< 0.0001, 0.0834, 0.8798) | 0.4729 (1.0000, 0.9241, 0.9866) |
| 1841 | 3.4431 (0.9970, 0.0109, 0.5352) | < 0.0001 (0.9463, 1.0000, 0.5546) |
| 1842 | < 0.0001 (0.2584, 0.0343, 0.9925) | < 0.0001 (1.0000, 1.0000, 0.9735) |
| 1843 | 0.3015 (0.1046, 0.0675, 0.8410) | 0.4685 (1.0000, 0.8982, 0.9864) |
| 1844 | < 0.0001 (< 0.0001, 0.0073, 0.9894) | < 0.0001 (0.9013, 1.0000, 0.7095) |
| 1845 | < 0.0001 (0.1310, 0.0060, 0.9994) | < 0.0001 (< 0.0001, 1.0000, 0.7613) |
| 1846 | < 0.0001 (0.0585, 0.0093, 1.0000) | < 0.0001 (1.0000, 1.0000, 0.9759) |
| 1847 | < 0.0001 (1.0000, 0.0065, 0.9651) | < 0.0001 (< 0.0001, 1.0000, 0.9020) |
| 1848 | < 0.0001 (0.6667, 0.0313, 0.9862) | < 0.0001 (< 0.0001, 1.0000, 0.8770) |
| 1849 | < 0.0001 (< 0.0001, 0.0061, 0.9937) | < 0.0001 (< 0.0001, 1.0000, 0.7442) |
| 1850 | 1.0000 (0.2966, 1.0000, 0.9798) | < 0.0001 (< 0.0001, 1.0000, 0.9185) |
| 1851 | < 0.0001 (0.9911, 0.0053, 0.9905) | < 0.0001 (1.0000, 1.0000, 0.9677) |
| 1852 | < 0.0001 (0.0260, 0.0107, 0.9960) | < 0.0001 (< 0.0001, 1.0000, 0.9266) |
| 1853 | 1.0000 (0.5084, 1.0000, 0.9216) | < 0.0001 (1.0000, 1.0000, 0.9898) |
| 1854 | < 0.0001 (< 0.0001, 0.0142, 1.0000) | 0.3510 (1.0000, 0.9497, 0.9983) |
| 1855 | 0.3360 (0.7130, 0.0917, 0.9981) | < 0.0001 (0.9783, 1.0000, 0.7517) |
| 1856 | 1.0000 (0.3625, 1.0000, 0.9798) | 0.2057 (< 0.0001, 0.9752, 0.9127) |
| 1857 | < 0.0001 (0.1310, 0.0060, 0.9998) | < 0.0001 (0.9686, 1.0000, 0.8529) |
| 1858 | < 0.0001 (0.0660, 0.0050, 0.9999) | 0.2058 (1.0000, 0.9752, 0.9867) |
| 1859 | < 0.0001 (0.4602, 0.0073, 0.9985) | 0.2500 (0.9686, 0.9629, 0.8533) |
| 1860 | 2.5087 (< 0.0001, 0.0254, 0.3794) | < 0.0001 (0.9630, 1.0000, 0.8689) |
| 1861 | 2.4200 (0.0934, 0.0147, 0.5087) | < 0.0001 (1.0000, 1.0000, 0.9710) |
| 1862 | 0.3105 (0.5084, 0.0737, 0.6886) | 0.1410 (1.0000, 0.9991, 0.9960) |
| 1863 | < 0.0001 (0.0936, 0.0060, 0.9969) | < 0.0001 (0.9994, 1.0000, 0.9929) |
| 1864 | < 0.0001 (0.0779, 0.0050, 0.9991) | < 0.0001 (1.0000, 1.0000, 0.9901) |
| 1865 | < 0.0001 (< 0.0001, 0.0083, 0.9772) | < 0.0001 (1.0000, 1.0000, 0.9649) |
| 1866 | 1.0000 (< 0.0001, 1.0000, 0.9798) | < 0.0001 (< 0.0001, 1.0000, 0.8642) |
| 1867 | < 0.0001 (0.4890, 0.0117, 0.9963) | < 0.0001 (< 0.0001, 1.0000, 0.9266) |
| 1868 | < 0.0001 (0.2326, 0.0083, 1.0000) | < 0.0001 (1.0000, 1.0000, 0.9986) |
| 1869 | < 0.0001 (0.9176, 0.0093, 0.9989) | < 0.0001 (< 0.0001, 1.0000, 0.9099) |
| 1870 | 0.2786 (0.0261, 0.0528, 0.9279) | < 0.0001 (< 0.0001, 1.0000, 0.9185) |
| 1871 | < 0.0001 (0.9093, 0.0083, 1.0000) | < 0.0001 (1.0000, 1.0000, 0.9996) |
| 1872 | < 0.0001 (0.6667, 0.0060, 0.9969) | < 0.0001 (< 0.0001, 1.0000, 0.9099) |
| 1873 | 1.0000 (0.0878, 1.0000, 0.9870) | 0.1077 (1.0000, 0.9975, 0.9538) |
| 1874 | < 0.0001 (1.0000, 0.0056, 0.9985) | < 0.0001 (0.9978, 1.0000, 0.8860) |
| 1875 | < 0.0001 (1.0000, 0.0053, 0.9985) | < 0.0001 (1.0000, 1.0000, 0.9890) |
| 1876 | < 0.0001 (0.0261, 0.0073, 0.9964) | < 0.0001 (1.0000, 1.0000, 0.9982) |
| 1877 | < 0.0001 (< 0.0001, 0.0060, 0.9991) | < 0.0001 (1.0000, 1.0000, 0.9494) |
| 1878 | < 0.0001 (0.6667, 0.0061, 1.0000) | < 0.0001 (< 0.0001, 1.0000, 0.8768) |
| 1879 | < 0.0001 (1.0000, 0.0126, 0.9986) | < 0.0001 (1.0000, 1.0000, 0.9430) |
| 1880 | < 0.0001 (0.1901, 0.0087, 0.9997) | < 0.0001 (1.0000, 1.0000, 0.9870) |
| 1881 | < 0.0001 (0.6505, 0.0425, 0.9991) | 0.7410 (1.0000, 0.7941, 0.9995) |
| 1882 | < 0.0001 (< 0.0001, 0.0343, 0.9808) | < 0.0001 (0.8173, 1.0000, 0.5793) |
| 1883 | 0.7016 (0.6667, 0.2972, 0.9622) | < 0.0001 (1.0000, 1.0000, 0.9901) |
| 1884 | < 0.0001 (0.0034, 0.0061, 0.9807) | < 0.0001 (< 0.0001, 1.0000, 0.8768) |
| 1885 | < 0.0001 (0.9070, 0.0060, 0.9947) | 0.5001 (1.0000, 0.8888, 0.9226) |
| 1886 | < 0.0001 (1.0000, 0.0060, 0.9996) | 1.0164 (0.8888, 0.4385, 0.8835) |
| 1887 | 1.2692 (0.4445, 0.3234, 0.2101) | < 0.0001 (0.4337, 1.0000, 0.5198) |
| 1888 | 0.3479 (< 0.0001, 0.1005, 0.9901) | < 0.0001 (< 0.0001, 1.0000, 0.8272) |
| 1889 | 0.3480 (0.4445, 0.1006, 0.9725) | < 0.0001 (1.0000, 1.0000, 0.9534) |
| 1890 | < 0.0001 (0.0262, 0.0343, 0.9921) | < 0.0001 (1.0000, 1.0000, 0.9811) |
| 1891 | < 0.0001 (0.4446, 0.0397, 0.9995) | < 0.0001 (< 0.0001, 1.0000, 0.7207) |
| 1892 | < 0.0001 (0.4445, 0.0053, 0.9994) | < 0.0001 (< 0.0001, 1.0000, 0.8711) |
| 1893 | < 0.0001 (0.0378, 0.0044, 0.9995) | < 0.0001 (1.0000, 1.0000, 0.9970) |
| 1894 | < 0.0001 (0.1317, 0.0060, 0.9969) | < 0.0001 (< 0.0001, 1.0000, 0.7846) |
| 1895 | < 0.0001 (0.0657, 0.0120, 1.0000) | < 0.0001 (1.0000, 1.0000, 0.9908) |
| 1896 | < 0.0001 (0.4231, 0.0142, 1.0000) | < 0.0001 (1.0000, 1.0000, 0.9986) |
| 1897 | < 0.0001 (0.0668, 0.0073, 0.9998) | < 0.0001 (< 0.0001, 1.0000, 0.8753) |
| 1898 | 0.9062 (0.6882, 0.4312, 0.9765) | < 0.0001 (1.0000, 1.0000, 0.9840) |
| 1899 | < 0.0001 (0.4444, 0.0073, 0.9964) | < 0.0001 (1.0000, 1.0000, 0.9874) |
| 1900 | 0.8330 (0.7680, 0.3950, 0.6732) | < 0.0001 (< 0.0001, 1.0000, 0.7295) |
| 1901 | < 0.0001 (0.6667, 0.0093, 0.9967) | 0.1577 (< 0.0001, 0.9894, 0.8642) |
| 1902 | 0.6336 (0.0035, 0.2427, 0.4854) | < 0.0001 (0.9925, 1.0000, 0.8214) |
| 1903 | < 0.0001 (0.1976, 0.0087, 1.0000) | 0.5077 (1.0000, 0.8858, 0.9928) |
| 1904 | < 0.0001 (0.2333, 0.0060, 0.9904) | < 0.0001 (0.9924, 1.0000, 0.9948) |
| 1905 | < 0.0001 (0.5084, 0.0053, 1.0000) | 0.2024 (1.0000, 0.9974, 0.9356) |
| 1906 | 1.3032 (< 0.0001, 0.3062, 0.2857) | < 0.0001 (0.9962, 1.0000, 0.9751) |
| 1907 | 0.3245 (0.6667, 0.0835, 0.9997) | < 0.0001 (1.0000, 1.0000, 0.9992) |
| 1908 | 1.0000 (0.4446, 1.0000, 0.9798) | < 0.0001 (1.0000, 1.0000, 0.9470) |
| 1909 | < 0.0001 (0.3625, 0.0065, 0.9894) | < 0.0001 (< 0.0001, 1.0000, 0.9386) |
| 1910 | < 0.0001 (< 0.0001, 0.0117, 0.9999) | < 0.0001 (1.0000, 1.0000, 0.9563) |
| 1911 | < 0.0001 (0.5084, 0.0083, 0.9994) | < 0.0001 (1.0000, 1.0000, 0.9956) |
| 1912 | < 0.0001 (0.9997, 0.0060, 0.9995) | < 0.0001 (< 0.0001, 1.0000, 0.9099) |
| 1913 | < 0.0001 (0.2878, 0.0050, 0.9989) | < 0.0001 (1.0000, 1.0000, 0.9436) |
| 1914 | 1.0000 (0.3620, 1.0000, 0.9490) | < 0.0001 (1.0000, 1.0000, 0.9997) |
| 1915 | < 0.0001 (0.4444, 0.0065, 0.9952) | < 0.0001 (< 0.0001, 1.0000, 0.9644) |
| 1916 | 1.0000 (0.5084, 1.0000, 0.9816) | < 0.0001 (1.0000, 1.0000, 0.9989) |
| 1917 | < 0.0001 (< 0.0001, 0.0107, 0.9960) | < 0.0001 (1.0000, 1.0000, 0.9927) |
| 1918 | < 0.0001 (0.6667, 0.0073, 0.9885) | < 0.0001 (< 0.0001, 1.0000, 0.7700) |
| 1919 | 1.0000 (0.1977, 1.0000, 0.9870) | < 0.0001 (1.0000, 1.0000, 0.9699) |
| 1920 | < 0.0001 (0.3528, 0.0056, 0.9998) | < 0.0001 (< 0.0001, 1.0000, 0.7700) |
| 1921 | < 0.0001 (0.4448, 0.0061, 0.9991) | < 0.0001 (< 0.0001, 1.0000, 0.9644) |
| 1922 | < 0.0001 (< 0.0001, 0.0053, 0.9980) | < 0.0001 (1.0000, 1.0000, 0.9901) |
| 1923 | < 0.0001 (0.1759, 0.0117, 1.0000) | < 0.0001 (1.0000, 1.0000, 0.9357) |
| 1924 | 0.3166 (0.4602, 0.0779, 0.9999) | < 0.0001 (1.0000, 1.0000, 0.9649) |
| 1925 | < 0.0001 (< 0.0001, 0.0061, 0.9998) | 0.4940 (1.0000, 0.8907, 0.9901) |
| 1926 | 1.3968 (< 0.0001, 0.2398, 0.6495) | < 0.0001 (0.9013, 1.0000, 0.7481) |
| 1927 | < 0.0001 (0.9096, 0.0041, 0.9904) | < 0.0001 (1.0000, 1.0000, 0.9836) |
| 1928 | < 0.0001 (0.7130, 0.0083, 0.9948) | < 0.0001 (1.0000, 1.0000, 0.9779) |
| 1929 | 0.3170 (0.2752, 0.0782, 0.9290) | < 0.0001 (1.0000, 1.0000, 0.9996) |
| 1930 | < 0.0001 (0.5084, 0.0065, 0.9952) | 0.8436 (1.0000, 0.5815, 0.9585) |
| 1931 | < 0.0001 (< 0.0001, 0.0037, 1.0000) | < 0.0001 (0.5567, 1.0000, 0.4111) |
| 1932 | 1.0000 (0.0878, 1.0000, 0.9436) | < 0.0001 (< 0.0001, 1.0000, 0.9266) |
| 1933 | 1.0000 (0.6603, 1.0000, 0.9431) | < 0.0001 (< 0.0001, 1.0000, 0.8711) |
| 1934 | < 0.0001 (< 0.0001, 0.0037, 0.9695) | < 0.0001 (< 0.0001, 1.0000, 0.7587) |
| 1935 | < 0.0001 (0.2963, 0.0083, 0.9948) | < 0.0001 (1.0000, 1.0000, 0.9986) |
| 1936 | < 0.0001 (0.2963, 0.0060, 0.9966) | < 0.0001 (1.0000, 1.0000, 0.9696) |
| 1937 | 1.0000 (< 0.0001, 1.0000, 0.9552) | 0.3043 (1.0000, 0.9639, 0.9954) |
| 1938 | < 0.0001 (0.1241, 0.0050, 0.9998) | < 0.0001 (0.9957, 1.0000, 0.8862) |
| 1939 | < 0.0001 (0.0015, 0.0060, 0.9999) | < 0.0001 (1.0000, 1.0000, 0.9696) |
| 1940 | 0.2201 (0.1165, 0.0220, 0.9125) | < 0.0001 (< 0.0001, 1.0000, 0.9644) |
| 1941 | 0.3013 (0.5084, 0.0674, 0.9831) | < 0.0001 (1.0000, 1.0000, 0.9822) |
| 1942 | 0.3358 (0.2585, 0.0916, 0.9411) | < 0.0001 (1.0000, 1.0000, 0.9696) |
| 1943 | < 0.0001 (< 0.0001, 0.0060, 0.9947) | < 0.0001 (< 0.0001, 1.0000, 0.8642) |
| 1944 | 1.0086 (0.0034, 0.4941, 0.7887) | < 0.0001 (1.0000, 1.0000, 0.9847) |
| 1945 | 1.2445 (< 0.0001, 0.3366, 0.5334) | < 0.0001 (1.0000, 1.0000, 0.9810) |
| 1946 | < 0.0001 (0.0871, 0.0044, 0.9964) | < 0.0001 (1.0000, 1.0000, 0.9778) |
| 1947 | < 0.0001 (0.1043, 0.0083, 0.9996) | < 0.0001 (1.0000, 1.0000, 0.9895) |
| 1948 | 1.2711 (0.1575, 0.3225, 0.9799) | < 0.0001 (< 0.0001, 1.0000, 0.8609) |
| 1949 | < 0.0001 (0.4883, 0.0425, 0.9993) | < 0.0001 (1.0000, 1.0000, 0.9868) |
| 1950 | < 0.0001 (0.9139, 0.0060, 0.9854) | < 0.0001 (< 0.0001, 1.0000, 0.8711) |
| 1951 | 1.0000 (0.7130, 1.0000, 0.9351) | 0.4143 (1.0000, 0.9142, 1.0000) |
| 1952 | 1.0000 (< 0.0001, 1.0000, 0.9552) | < 0.0001 (0.9004, 1.0000, 0.7324) |
| 1953 | < 0.0001 (0.9258, 0.0107, 0.9999) | < 0.0001 (1.0000, 1.0000, 0.9356) |
| 1954 | 1.0000 (< 0.0001, 1.0000, 0.9480) | < 0.0001 (1.0000, 1.0000, 0.9996) |
| 1955 | < 0.0001 (0.9176, 0.0083, 0.9948) | < 0.0001 (1.0000, 1.0000, 0.9989) |
| 1956 | < 0.0001 (0.2281, 0.0050, 1.0000) | < 0.0001 (1.0000, 1.0000, 0.9650) |
| 1957 | 1.0000 (< 0.0001, 1.0000, 0.9480) | < 0.0001 (1.0000, 1.0000, 0.9777) |
| 1958 | < 0.0001 (0.0077, 0.0061, 0.9937) | < 0.0001 (1.0000, 1.0000, 0.9802) |
| 1959 | 0.2786 (0.0878, 0.0528, 0.9881) | < 0.0001 (1.0000, 1.0000, 0.9738) |
| 1960 | < 0.0001 (0.5084, 0.0061, 0.9987) | < 0.0001 (1.0000, 1.0000, 0.9562) |
| 1961 | < 0.0001 (< 0.0001, 0.0061, 1.0000) | < 0.0001 (< 0.0001, 1.0000, 0.9099) |
| 1962 | < 0.0001 (1.0000, 0.0343, 0.9925) | < 0.0001 (1.0000, 1.0000, 0.9172) |
| 1963 | < 0.0001 (< 0.0001, 0.0044, 0.9964) | < 0.0001 (1.0000, 1.0000, 1.0000) |
| 1964 | < 0.0001 (0.6667, 0.0085, 0.9759) | < 0.0001 (1.0000, 1.0000, 0.9902) |
| 1965 | < 0.0001 (0.7130, 0.0060, 0.9978) | < 0.0001 (1.0000, 1.0000, 0.9834) |
| 1966 | < 0.0001 (< 0.0001, 0.0085, 1.0000) | < 0.0001 (< 0.0001, 1.0000, 0.9386) |
| 1967 | 1.5899 (0.3356, 0.1681, 0.7378) | < 0.0001 (1.0000, 1.0000, 0.9777) |
| 1968 | < 0.0001 (0.5084, 0.0056, 0.9995) | < 0.0001 (1.0000, 1.0000, 0.9837) |
| 1969 | 1.0000 (< 0.0001, 1.0000, 0.9342) | < 0.0001 (1.0000, 1.0000, 0.9934) |
| 1970 | < 0.0001 (0.0011, 0.0053, 0.9870) | < 0.0001 (< 0.0001, 1.0000, 0.8770) |
| 1971 | 2.1752 (0.0882, 0.0460, 0.7191) | < 0.0001 (1.0000, 1.0000, 0.9835) |
| 1972 | 1.1795 (0.3259, 0.3905, 0.8331) | < 0.0001 (1.0000, 1.0000, 0.9985) |
| 1973 | < 0.0001 (0.0125, 0.0343, 0.9808) | < 0.0001 (< 0.0001, 1.0000, 0.9188) |
| 1974 | < 0.0001 (0.2491, 0.0083, 0.9772) | < 0.0001 (1.0000, 1.0000, 0.9538) |
| 1975 | < 0.0001 (< 0.0001, 0.0056, 0.9954) | < 0.0001 (1.0000, 1.0000, 0.9948) |
| 1976 | 1.0000 (0.4445, 1.0000, 0.8213) | 1.0188 (1.0000, 0.4387, 0.9961) |
| 1977 | 1.0000 (1.0000, 1.0000, 0.9404) | < 0.0001 (0.4263, 1.0000, 0.4989) |
| 1978 | < 0.0001 (0.0023, 0.0142, 0.9996) | < 0.0001 (1.0000, 1.0000, 0.9845) |
| 1979 | 1.0000 (0.2585, 1.0000, 0.8511) | < 0.0001 (1.0000, 1.0000, 0.9840) |
| 1980 | < 0.0001 (0.0007, 0.0142, 0.9993) | < 0.0001 (1.0000, 1.0000, 0.9967) |
| 1981 | < 0.0001 (< 0.0001, 0.0056, 1.0000) | 0.4897 (< 0.0001, 0.6806, 0.9020) |
| 1982 | < 0.0001 (1.0000, 0.0056, 0.9994) | < 0.0001 (0.6756, 1.0000, 0.5245) |
| 1983 | 0.3688 (1.0000, 0.1162, 0.9739) | < 0.0001 (< 0.0001, 1.0000, 0.9386) |
| 1984 | < 0.0001 (< 0.0001, 0.0050, 0.9999) | < 0.0001 (1.0000, 1.0000, 0.9869) |
| 1985 | < 0.0001 (0.6667, 0.0053, 0.9870) | < 0.0001 (< 0.0001, 1.0000, 0.8315) |
| 1986 | 1.0000 (0.5083, 1.0000, 0.9351) | < 0.0001 (1.0000, 1.0000, 0.9172) |
| 1987 | 0.3246 (0.0077, 0.0835, 0.9295) | < 0.0001 (1.0000, 1.0000, 0.9821) |
| 1988 | < 0.0001 (0.3258, 0.0065, 0.9999) | < 0.0001 (1.0000, 1.0000, 0.9985) |
| 1989 | 0.7819 (0.0242, 0.3585, 1.0000) | < 0.0001 (1.0000, 1.0000, 0.9737) |
| 1990 | < 0.0001 (0.3349, 0.0053, 0.9905) | < 0.0001 (< 0.0001, 1.0000, 0.9127) |
| 1991 | 3.9228 (0.5084, 0.0002, 0.9969) | < 0.0001 (< 0.0001, 1.0000, 0.7700) |
| 1992 | < 0.0001 (0.0588, 0.0061, 1.0000) | 0.4688 (< 0.0001, 0.8981, 0.9127) |
| 1993 | < 0.0001 (0.0084, 0.0099, 0.9798) | < 0.0001 (0.9012, 1.0000, 0.7108) |
| 1994 | 1.0000 (0.7130, 1.0000, 0.9798) | < 0.0001 (1.0000, 1.0000, 0.9978) |
| 1995 | < 0.0001 (0.4444, 0.0050, 0.9817) | < 0.0001 (1.0000, 1.0000, 0.9868) |
| 1996 | < 0.0001 (0.1317, 0.0073, 0.9995) | < 0.0001 (1.0000, 1.0000, 0.9700) |
| 1997 | < 0.0001 (0.2963, 0.0061, 0.9988) | < 0.0001 (1.0000, 1.0000, 0.9358) |
| 1998 | < 0.0001 (0.1318, 0.0313, 0.9929) | < 0.0001 (1.0000, 1.0000, 0.9357) |
| 1999 | < 0.0001 (0.2963, 0.0060, 0.9999) | < 0.0001 (< 0.0001, 1.0000, 0.8642) |
| 2000 | 1.0000 (0.0878, 1.0000, 0.9870) | < 0.0001 (1.0000, 1.0000, 0.9960) |
| 2001 | 0.6734 (0.0078, 0.3361, 0.9968) | < 0.0001 (1.0000, 1.0000, 0.9868) |
| 2002 | < 0.0001 (0.7130, 0.0065, 0.9993) | < 0.0001 (1.0000, 1.0000, 0.9674) |
| 2003 | 1.0000 (< 0.0001, 1.0000, 0.8657) | < 0.0001 (1.0000, 1.0000, 0.9899) |
| 2004 | < 0.0001 (0.7065, 0.0060, 0.9957) | < 0.0001 (1.0000, 1.0000, 0.9997) |
| 2005 | < 0.0001 (0.0878, 0.0053, 0.9753) | < 0.0001 (< 0.0001, 1.0000, 0.8770) |
| 2006 | 1.0000 (0.3625, 1.0000, 0.9870) | < 0.0001 (< 0.0001, 1.0000, 0.8091) |
| 2007 | < 0.0001 (0.6667, 0.0093, 1.0000) | < 0.0001 (1.0000, 1.0000, 0.9811) |
| 2008 | < 0.0001 (0.6667, 0.0053, 0.9958) | < 0.0001 (1.0000, 1.0000, 0.9650) |
| 2009 | < 0.0001 (0.9715, 0.0060, 0.9854) | < 0.0001 (1.0000, 1.0000, 0.9908) |
| 2010 | < 0.0001 (0.6667, 0.0425, 0.9993) | 0.2343 (1.0000, 0.9675, 0.9696) |
| 2011 | 0.9521 (0.0585, 0.4659, 0.7056) | < 0.0001 (0.9690, 1.0000, 0.8347) |
| 2012 | < 0.0001 (0.5083, 0.0061, 0.9937) | 0.4898 (1.0000, 0.6806, 0.9901) |
| 2013 | < 0.0001 (0.1975, 0.0343, 0.9999) | < 0.0001 (0.6756, 1.0000, 0.5244) |
| 2014 | < 0.0001 (0.7130, 0.0117, 1.0000) | < 0.0001 (1.0000, 1.0000, 0.9978) |
| 2015 | 0.4162 (0.4602, 0.1527, 0.8498) | < 0.0001 (1.0000, 1.0000, 0.9851) |
| 2016 | 0.6038 (0.1975, 0.2910, 0.8913) | 0.4895 (< 0.0001, 0.6810, 0.9386) |
| 2017 | 1.0000 (0.0878, 1.0000, 0.9552) | < 0.0001 (0.6858, 1.0000, 0.4175) |
| 2018 | 1.0000 (< 0.0001, 1.0000, 0.9404) | < 0.0001 (1.0000, 1.0000, 0.9985) |
| 2019 | 1.0000 (< 0.0001, 1.0000, 0.8339) | < 0.0001 (1.0000, 1.0000, 0.9957) |
| 2020 | < 0.0001 (0.4444, 0.0073, 0.9998) | < 0.0001 (< 0.0001, 1.0000, 0.8858) |
| 2021 | < 0.0001 (0.0878, 0.0060, 0.9904) | < 0.0001 (1.0000, 1.0000, 0.9864) |
| 2022 | 0.3480 (0.0937, 0.1006, 1.0000) | 0.5074 (< 0.0001, 0.8867, 0.8142) |
| 2023 | 1.2516 (1.0000, 0.3541, 0.9998) | < 0.0001 (0.8843, 1.0000, 0.8858) |
| 2024 | < 0.0001 (0.2492, 0.0050, 0.9988) | < 0.0001 (< 0.0001, 1.0000, 0.7207) |
| 2025 | 1.0000 (0.0122, 1.0000, 0.9870) | < 0.0001 (1.0000, 1.0000, 0.9959) |
| 2026 | 0.3245 (< 0.0001, 0.0835, 0.9657) | < 0.0001 (1.0000, 1.0000, 0.9494) |
| 2027 | < 0.0001 (0.6667, 0.0056, 0.9985) | < 0.0001 (1.0000, 1.0000, 0.9954) |
| 2028 | < 0.0001 (0.3527, 0.0073, 0.9885) | 0.5003 (1.0000, 0.6883, 0.9987) |
| 2029 | < 0.0001 (0.2383, 0.0142, 0.9999) | < 0.0001 (0.6831, 1.0000, 0.5041) |
| 2030 | < 0.0001 (0.0880, 0.0095, 0.9998) | < 0.0001 (1.0000, 1.0000, 0.9866) |
| 2031 | < 0.0001 (< 0.0001, 0.0126, 1.0000) | < 0.0001 (1.0000, 1.0000, 1.0000) |
| 2032 | < 0.0001 (0.4445, 0.0044, 0.9986) | < 0.0001 (< 0.0001, 1.0000, 0.8770) |
| 2033 | < 0.0001 (0.5084, 0.0060, 0.9854) | < 0.0001 (< 0.0001, 1.0000, 0.7613) |
| 2034 | < 0.0001 (< 0.0001, 0.0073, 0.9998) | < 0.0001 (1.0000, 1.0000, 0.9993) |
| 2035 | < 0.0001 (0.7130, 0.0073, 0.9894) | < 0.0001 (1.0000, 1.0000, 0.9676) |
| 2036 | < 0.0001 (< 0.0001, 0.0044, 1.0000) | < 0.0001 (1.0000, 1.0000, 0.9916) |
| 2037 | 0.3167 (0.9250, 0.0780, 0.9889) | < 0.0001 (1.0000, 1.0000, 0.9867) |
| 2038 | < 0.0001 (0.4444, 0.0053, 0.9753) | < 0.0001 (1.0000, 1.0000, 0.9881) |
| 2039 | < 0.0001 (0.4601, 0.0117, 1.0000) | < 0.0001 (1.0000, 1.0000, 0.9910) |
| 2040 | < 0.0001 (0.7130, 0.0424, 0.9998) | 0.3501 (0.9781, 0.9522, 0.9421) |
| 2041 | < 0.0001 (0.7825, 0.0073, 0.9885) | < 0.0001 (1.0000, 1.0000, 0.7084) |
| 2042 | < 0.0001 (0.5084, 0.0425, 0.9944) | < 0.0001 (1.0000, 1.0000, 0.9310) |
| 2043 | < 0.0001 (0.3426, 0.0397, 0.9982) | < 0.0001 (1.0000, 1.0000, 0.9793) |
| 2044 | 0.2862 (0.0935, 0.0576, 0.8978) | < 0.0001 (1.0000, 1.0000, 0.9393) |
| 2045 | 0.3013 (0.6667, 0.0674, 0.9708) | < 0.0001 (1.0000, 1.0000, 0.9295) |
| 2046 | < 0.0001 (< 0.0001, 0.0061, 0.9948) | < 0.0001 (1.0000, 1.0000, 0.9233) |
| 2047 | < 0.0001 (< 0.0001, 0.0065, 0.9856) | < 0.0001 (1.0000, 1.0000, 0.9995) |
| 2048 | < 0.0001 (0.3420, 0.0415, 1.0000) | < 0.0001 (1.0000, 1.0000, 0.9563) |
| 2049 | < 0.0001 (0.5549, 0.0093, 0.9993) | < 0.0001 (< 0.0001, 1.0000, 0.9745) |
| 2050 | < 0.0001 (0.2999, 0.0083, 0.9970) | < 0.0001 (1.0000, 1.0000, 0.9334) |
| 2051 | 1.0000 (1.0000, 1.0000, 0.9798) | < 0.0001 (< 0.0001, 1.0000, 0.9895) |
| 2052 | < 0.0001 (0.9881, 0.0425, 0.9944) | < 0.0001 (1.0000, 1.0000, 0.7823) |
| 2053 | < 0.0001 (0.1836, 0.0061, 0.9998) | < 0.0001 (1.0000, 1.0000, 0.9215) |
| 2054 | 0.8330 (0.0667, 0.3950, 0.9540) | < 0.0001 (< 0.0001, 1.0000, 0.9595) |
| 2055 | 0.3167 (0.0116, 0.0779, 0.7199) | < 0.0001 (1.0000, 1.0000, 0.9334) |
| 2056 | 1.0453 (0.3121, 0.4697, 0.9681) | < 0.0001 (1.0000, 1.0000, 0.9945) |
| 2057 | < 0.0001 (0.4445, 0.0061, 1.0000) | < 0.0001 (1.0000, 1.0000, 0.9745) |
| 2058 | 1.0000 (0.4445, 1.0000, 0.9560) | < 0.0001 (< 0.0001, 1.0000, 0.9765) |
| 2059 | < 0.0001 (0.0005, 0.0061, 0.9937) | < 0.0001 (< 0.0001, 1.0000, 0.8853) |
| 2060 | < 0.0001 (< 0.0001, 0.0060, 0.9978) | < 0.0001 (1.0000, 1.0000, 0.7382) |
| 2061 | 0.3482 (0.0393, 0.1007, 1.0000) | < 0.0001 (1.0000, 1.0000, 0.9736) |
| 2062 | 0.8096 (0.4883, 0.3785, 0.7038) | < 0.0001 (1.0000, 1.0000, 0.9889) |
| 2063 | < 0.0001 (0.6667, 0.0142, 0.9991) | < 0.0001 (< 0.0001, 1.0000, 0.8990) |
| 2064 | 0.3687 (0.1051, 0.1161, 0.9868) | < 0.0001 (< 0.0001, 1.0000, 0.8289) |
| 2065 | < 0.0001 (0.5084, 0.0065, 0.9996) | < 0.0001 (1.0000, 1.0000, 0.9334) |
| 2066 | 1.0000 (0.7130, 1.0000, 0.9436) | < 0.0001 (1.0000, 1.0000, 0.9304) |
| 2067 | 0.5728 (0.6157, 0.1927, 0.7412) | < 0.0001 (1.0000, 1.0000, 0.9233) |
| 2068 | 1.0000 (0.6667, 1.0000, 0.9798) | < 0.0001 (< 0.0001, 1.0000, 0.9837) |
| 2069 | < 0.0001 (0.1976, 0.0087, 0.9982) | < 0.0001 (1.0000, 1.0000, 0.8207) |
| 2070 | < 0.0001 (0.8891, 0.0117, 0.9874) | < 0.0001 (1.0000, 1.0000, 0.9884) |
| 2071 | < 0.0001 (0.4445, 0.0065, 0.9933) | 0.8678 (0.9046, 0.7807, 0.9737) |
| 2072 | < 0.0001 (0.0878, 0.0056, 0.9997) | < 0.0001 (< 0.0001, 1.0000, 0.6661) |
| 2073 | < 0.0001 (0.0123, 0.0093, 1.0000) | < 0.0001 (< 0.0001, 1.0000, 0.7607) |
| 2074 | 0.2863 (0.7130, 0.0576, 0.9529) | < 0.0001 (< 0.0001, 1.0000, 0.7259) |
| 2075 | 1.0000 (< 0.0001, 1.0000, 0.9490) | < 0.0001 (1.0000, 1.0000, 0.7259) |
| 2076 | < 0.0001 (0.6667, 0.0053, 0.9753) | < 0.0001 (1.0000, 1.0000, 0.9264) |
| 2077 | 1.0000 (0.0116, 1.0000, 0.9490) | < 0.0001 (1.0000, 1.0000, 0.9946) |
| 2078 | 0.9611 (< 0.0001, 0.4725, 0.7650) | < 0.0001 (1.0000, 1.0000, 0.9215) |
| 2079 | < 0.0001 (0.2963, 0.0436, 1.0000) | < 0.0001 (< 0.0001, 1.0000, 0.9664) |
| 2080 | < 0.0001 (0.0585, 0.0073, 0.9961) | < 0.0001 (1.0000, 1.0000, 0.9334) |
| 2081 | < 0.0001 (0.2963, 0.0065, 0.9894) | < 0.0001 (1.0000, 1.0000, 0.9889) |
| 2082 | < 0.0001 (0.4829, 0.0053, 0.9753) | < 0.0001 (< 0.0001, 1.0000, 0.9413) |
| 2083 | < 0.0001 (< 0.0001, 0.0053, 0.9997) | < 0.0001 (1.0000, 1.0000, 0.7823) |
| 2084 | 1.0000 (0.0475, 1.0000, 0.9351) | < 0.0001 (1.0000, 1.0000, 0.9134) |
| 2085 | < 0.0001 (0.5084, 0.0083, 0.9996) | < 0.0001 (< 0.0001, 1.0000, 0.9680) |
| 2086 | < 0.0001 (0.2817, 0.0073, 0.9999) | < 0.0001 (1.0000, 1.0000, 0.8631) |
| 2087 | 0.3480 (0.4445, 0.1006, 0.9401) | < 0.0001 (1.0000, 1.0000, 0.9461) |
| 2088 | < 0.0001 (0.6784, 0.0060, 1.0000) | < 0.0001 (1.0000, 1.0000, 0.9988) |
| 2089 | < 0.0001 (1.0000, 0.0050, 1.0000) | < 0.0001 (1.0000, 1.0000, 0.9754) |
| 2090 | 1.0000 (0.1313, 1.0000, 0.9431) | < 0.0001 (1.0000, 1.0000, 0.8865) |
| 2091 | 1.0000 (0.8002, 1.0000, 0.8565) | 0.9696 (0.6818, 0.4727, 0.9765) |
| 2092 | < 0.0001 (0.2964, 0.0056, 0.9914) | < 0.0001 (1.0000, 1.0000, 0.3939) |
| 2093 | < 0.0001 (0.2280, 0.0073, 0.9961) | < 0.0001 (1.0000, 1.0000, 0.9980) |
| 2094 | < 0.0001 (0.1313, 0.0041, 0.9996) | < 0.0001 (1.0000, 1.0000, 0.9801) |
| 2095 | < 0.0001 (0.2963, 0.0060, 0.9904) | 0.5000 (0.9877, 0.8889, 0.9393) |
| 2096 | 0.3170 (0.7130, 0.0782, 0.9676) | 0.8718 (0.3664, 0.3951, 0.9417) |
| 2097 | < 0.0001 (< 0.0001, 0.0424, 0.9996) | < 0.0001 (1.0000, 1.0000, 0.2236) |
| 2098 | 0.2862 (0.7408, 0.0576, 0.8979) | 0.4306 (0.9066, 0.9094, 0.9377) |
| 2099 | < 0.0001 (0.2753, 0.0093, 1.0000) | < 0.0001 (1.0000, 1.0000, 0.6047) |
| 2100 | < 0.0001 (0.6667, 0.0085, 0.9980) | < 0.0001 (< 0.0001, 1.0000, 0.9590) |
| 2101 | 1.0000 (< 0.0001, 1.0000, 0.9798) | < 0.0001 (1.0000, 1.0000, 0.9334) |
| 2102 | 0.3170 (0.3527, 0.0782, 0.5432) | < 0.0001 (< 0.0001, 1.0000, 0.9704) |
| 2103 | < 0.0001 (0.1976, 0.0065, 0.9999) | < 0.0001 (< 0.0001, 1.0000, 0.9334) |
| 2104 | < 0.0001 (< 0.0001, 0.0061, 0.9987) | < 0.0001 (1.0000, 1.0000, 0.7259) |
| 2105 | 1.0000 (0.0242, 1.0000, 0.9411) | < 0.0001 (1.0000, 1.0000, 0.9775) |
| 2106 | < 0.0001 (0.0585, 0.0083, 0.9948) | < 0.0001 (1.0000, 1.0000, 1.0000) |
| 2107 | 1.0000 (0.0003, 1.0000, 0.8837) | < 0.0001 (1.0000, 1.0000, 0.9995) |
| 2108 | 0.3925 (< 0.0001, 0.1343, 0.9992) | < 0.0001 (1.0000, 1.0000, 0.9906) |
| 2109 | < 0.0001 (0.4736, 0.0065, 0.9651) | < 0.0001 (< 0.0001, 1.0000, 0.9474) |
| 2110 | < 0.0001 (0.1977, 0.0099, 1.0000) | < 0.0001 (< 0.0001, 1.0000, 0.9334) |
| 2111 | < 0.0001 (0.0878, 0.0343, 0.9817) | 0.5147 (0.6541, 0.6481, 0.7143) |
| 2112 | < 0.0001 (0.1318, 0.0061, 0.9999) | < 0.0001 (< 0.0001, 1.0000, 0.4394) |
| 2113 | < 0.0001 (0.0392, 0.0425, 0.9999) | < 0.0001 (1.0000, 1.0000, 0.8030) |
| 2114 | 0.3167 (0.1244, 0.0779, 0.7201) | < 0.0001 (< 0.0001, 1.0000, 0.8954) |
| 2115 | 0.3193 (< 0.0001, 0.0798, 0.9850) | < 0.0001 (1.0000, 1.0000, 0.7541) |
| 2116 | < 0.0001 (0.1311, 0.0117, 1.0000) | < 0.0001 (1.0000, 1.0000, 0.9561) |
| 2117 | 1.0000 (0.2963, 1.0000, 0.9431) | < 0.0001 (< 0.0001, 1.0000, 0.9741) |
| 2118 | < 0.0001 (0.3514, 0.0065, 0.9894) | < 0.0001 (1.0000, 1.0000, 0.9286) |
| 2119 | < 0.0001 (0.0391, 0.0117, 1.0000) | 0.1509 (0.9950, 0.9963, 0.9812) |
| 2120 | < 0.0001 (0.0007, 0.0061, 0.9960) | < 0.0001 (1.0000, 1.0000, 0.9971) |
| 2121 | < 0.0001 (0.0260, 0.0120, 0.9994) | 0.4999 (0.6668, 0.6668, 0.9562) |
| 2122 | < 0.0001 (0.0087, 0.0060, 1.0000) | 6.4987 (0.0794, 0.0274, 0.3165) |
| 2123 | < 0.0001 (0.9176, 0.0060, 0.9998) | < 0.0001 (1.0000, 1.0000, 0.9750) |
| 2124 | < 0.0001 (0.4158, 0.0085, 0.9999) | < 0.0001 (1.0000, 1.0000, 1.0000) |
| 2125 | < 0.0001 (0.9387, 0.0107, 0.9960) | < 0.0001 (< 0.0001, 1.0000, 0.9377) |
| 2126 | < 0.0001 (1.0000, 0.0343, 0.9999) | 0.0714 (0.9547, 0.9998, 0.7259) |
| 2127 | 0.2200 (1.0000, 0.0220, 0.9126) | 0.2500 (0.9630, 0.9630, 0.7544) |
| 2128 | < 0.0001 (0.4444, 0.0056, 0.9960) | < 0.0001 (1.0000, 1.0000, 0.9349) |
| 2129 | < 0.0001 (< 0.0001, 0.0126, 0.9997) | < 0.0001 (< 0.0001, 1.0000, 1.0000) |
| 2130 | 0.6344 (0.0667, 0.2434, 0.6702) | < 0.0001 (1.0000, 1.0000, 0.7849) |
| 2131 | < 0.0001 (< 0.0001, 0.0073, 0.9894) | < 0.0001 (1.0000, 1.0000, 0.9936) |
| 2132 | < 0.0001 (0.5084, 0.0083, 0.9988) | < 0.0001 (< 0.0001, 1.0000, 0.9297) |
| 2133 | < 0.0001 (0.0175, 0.0424, 1.0000) | < 0.0001 (< 0.0001, 1.0000, 0.8853) |
| 2134 | < 0.0001 (0.1481, 0.0060, 0.9991) | < 0.0001 (1.0000, 1.0000, 0.9334) |
| 2135 | < 0.0001 (0.9236, 0.0060, 0.9969) | < 0.0001 (1.0000, 1.0000, 0.9604) |
| 2136 | 1.0000 (0.3625, 1.0000, 0.9870) | < 0.0001 (1.0000, 1.0000, 0.9134) |
| 2137 | < 0.0001 (1.0000, 0.0093, 0.9962) | 0.2500 (0.9630, 0.9630, 0.9233) |
| 2138 | < 0.0001 (0.1976, 0.0073, 0.9894) | < 0.0001 (1.0000, 1.0000, 0.9236) |
| 2139 | < 0.0001 (0.9258, 0.0099, 0.9953) | < 0.0001 (1.0000, 1.0000, 0.9770) |
| 2140 | < 0.0001 (0.6667, 0.0117, 0.9993) | < 0.0001 (1.0000, 1.0000, 0.9983) |
| 2141 | 1.0000 (0.0878, 1.0000, 0.9870) | < 0.0001 (1.0000, 1.0000, 0.9412) |
| 2142 | < 0.0001 (0.0030, 0.0056, 0.9998) | < 0.0001 (1.0000, 1.0000, 0.9895) |
| 2143 | < 0.0001 (0.6711, 0.0060, 0.9969) | < 0.0001 (1.0000, 1.0000, 0.9956) |
| 2144 | < 0.0001 (0.1316, 0.0050, 0.9951) | < 0.0001 (< 0.0001, 1.0000, 0.9088) |
| 2145 | 1.0000 (1.0000, 1.0000, 0.9798) | < 0.0001 (1.0000, 1.0000, 0.9334) |
| 2146 | 1.0110 (0.1759, 0.4925, 0.7878) | < 0.0001 (1.0000, 1.0000, 0.9377) |
| 2147 | < 0.0001 (1.0000, 0.0061, 0.9988) | < 0.0001 (1.0000, 1.0000, 0.9813) |
| 2148 | < 0.0001 (< 0.0001, 0.0126, 1.0000) | < 0.0001 (1.0000, 1.0000, 0.9953) |
| 2149 | 0.3245 (< 0.0001, 0.0835, 0.7774) | < 0.0001 (1.0000, 1.0000, 0.9974) |
| 2150 | 1.0000 (0.4444, 1.0000, 0.8886) | < 0.0001 (1.0000, 1.0000, 0.9985) |
| 2151 | 1.0000 (0.0023, 1.0000, 0.9870) | < 0.0001 (1.0000, 1.0000, 0.9953) |
| 2152 | 0.4162 (0.9997, 0.1527, 0.9820) | < 0.0001 (< 0.0001, 1.0000, 0.9662) |
| 2153 | < 0.0001 (< 0.0001, 0.0083, 0.9970) | < 0.0001 (< 0.0001, 1.0000, 0.9334) |
| 2154 | < 0.0001 (0.3616, 0.0083, 0.9987) | < 0.0001 (< 0.0001, 1.0000, 0.7541) |
| 2155 | 1.0000 (0.5483, 1.0000, 0.8868) | < 0.0001 (1.0000, 1.0000, 0.8355) |
| 2156 | < 0.0001 (0.3625, 0.0050, 0.9997) | < 0.0001 (1.0000, 1.0000, 0.9853) |
| 2157 | < 0.0001 (< 0.0001, 0.0061, 0.9988) | < 0.0001 (< 0.0001, 1.0000, 0.9393) |
| 2158 | 0.6720 (0.0175, 0.2737, 0.7136) | < 0.0001 (1.0000, 1.0000, 0.8072) |
| 2159 | 1.0000 (< 0.0001, 1.0000, 0.9379) | < 0.0001 (1.0000, 1.0000, 0.9297) |
| 2160 | 1.0000 (0.0004, 1.0000, 0.9216) | < 0.0001 (1.0000, 1.0000, 0.9933) |
| 2161 | < 0.0001 (0.4444, 0.0060, 0.9854) | 0.4842 (0.4786, 0.7028, 0.9295) |
| 2162 | 0.2920 (0.1975, 0.0613, 0.8865) | < 0.0001 (1.0000, 1.0000, 0.2921) |
| 2163 | < 0.0001 (0.2585, 0.0061, 0.9978) | < 0.0001 (< 0.0001, 1.0000, 0.9271) |
| 2164 | < 0.0001 (0.1840, 0.0099, 0.9853) | < 0.0001 (1.0000, 1.0000, 0.6917) |
| 2165 | 0.5849 (0.2964, 0.2027, 0.9028) | < 0.0001 (1.0000, 1.0000, 0.9987) |
| 2166 | < 0.0001 (0.1738, 0.0014, 0.9870) | < 0.0001 (< 0.0001, 1.0000, 0.8866) |
| 2167 | < 0.0001 (0.5084, 0.0061, 0.9995) | < 0.0001 (1.0000, 1.0000, 0.8584) |
| 2168 | < 0.0001 (0.0937, 0.0424, 0.9944) | < 0.0001 (1.0000, 1.0000, 0.9735) |
| 2169 | 1.0000 (< 0.0001, 1.0000, 0.9404) | < 0.0001 (< 0.0001, 1.0000, 0.9596) |
| 2170 | < 0.0001 (0.6944, 0.0073, 0.9961) | < 0.0001 (1.0000, 1.0000, 0.7143) |
| 2171 | < 0.0001 (0.2964, 0.0425, 0.9973) | < 0.0001 (1.0000, 1.0000, 0.9281) |
| 2172 | 1.0000 (0.7066, 1.0000, 0.9552) | 2.4315 (0.1112, 0.3634, 0.9890) |
| 2173 | < 0.0001 (0.1312, 0.0065, 0.9989) | < 0.0001 (1.0000, 1.0000, 0.9270) |
| 2174 | < 0.0001 (0.6603, 0.0061, 1.0000) | < 0.0001 (1.0000, 1.0000, 0.9852) |
| 2175 | 0.7991 (0.9983, 0.3709, 0.9821) | < 0.0001 (1.0000, 1.0000, 0.9680) |
| 2176 | < 0.0001 (0.1843, 0.0343, 0.9925) | < 0.0001 (1.0000, 1.0000, 0.9393) |
| 2177 | < 0.0001 (0.0391, 0.0050, 0.9989) | 0.2500 (0.9877, 0.9630, 0.9854) |
| 2178 | 0.6356 (0.5928, 0.2443, 0.3575) | 0.6718 (0.9858, 0.9848, 0.8755) |
| 2179 | < 0.0001 (0.2584, 0.0061, 0.9998) | < 0.0001 (< 0.0001, 1.0000, 0.6023) |
| 2180 | < 0.0001 (0.0263, 0.0397, 0.9999) | < 0.0001 (1.0000, 1.0000, 0.8289) |
| 2181 | < 0.0001 (0.0585, 0.0060, 0.9996) | < 0.0001 (< 0.0001, 1.0000, 0.9382) |
| 2182 | 0.6407 (0.9954, 0.2484, 0.9870) | < 0.0001 (1.0000, 1.0000, 0.8355) |
| 2183 | 1.3673 (0.0519, 0.3372, 0.8746) | < 0.0001 (< 0.0001, 1.0000, 0.9900) |
| 2184 | < 0.0001 (0.0660, 0.0397, 0.9982) | < 0.0001 (1.0000, 1.0000, 0.8510) |
| 2185 | 0.2786 (0.8889, 0.0528, 0.7574) | < 0.0001 (< 0.0001, 1.0000, 0.9590) |
| 2186 | 0.3480 (0.0339, 0.1006, 0.9439) | < 0.0001 (< 0.0001, 1.0000, 0.8510) |
| 2187 | 1.0000 (0.7130, 1.0000, 0.9870) | < 0.0001 (0.4638, 1.0000, 0.9444) |
| 2188 | < 0.0001 (< 0.0001, 0.0050, 0.9963) | < 0.0001 (1.0000, 1.0000, 0.9398) |
| 2189 | 0.4455 (0.1842, 0.0934, 0.8750) | < 0.0001 (< 0.0001, 1.0000, 0.9537) |
| 2190 | < 0.0001 (0.0022, 0.0060, 0.9854) | < 0.0001 (1.0000, 1.0000, 0.9791) |
| 2191 | 0.3480 (< 0.0001, 0.1006, 0.9959) | < 0.0001 (1.0000, 1.0000, 0.8921) |
| 2192 | < 0.0001 (0.3349, 0.0117, 1.0000) | < 0.0001 (1.0000, 1.0000, 0.8734) |
| 2193 | < 0.0001 (0.6944, 0.0117, 1.0000) | 1.4757 (< 0.0001, 0.3385, 0.9920) |
| 2194 | < 0.0001 (0.7130, 0.0126, 1.0000) | < 0.0001 (1.0000, 1.0000, 0.1940) |
| 2195 | < 0.0001 (0.9997, 0.0126, 0.9972) | < 0.0001 (1.0000, 1.0000, 0.9708) |
| 2196 | 1.0000 (0.1317, 1.0000, 0.9870) | 0.3993 (< 0.0001, 0.9186, 0.9448) |
| 2197 | 1.0000 (0.5082, 1.0000, 0.9230) | < 0.0001 (1.0000, 1.0000, 0.6577) |
| 2198 | 0.3635 (0.3574, 0.1121, 0.7573) | 0.3502 (1.0000, 0.9520, 0.9761) |
| 2199 | < 0.0001 (0.0173, 0.0060, 0.9904) | 0.1520 (1.0000, 0.9904, 0.7636) |
| 2200 | < 0.0001 (< 0.0001, 0.0056, 0.9696) | 0.2664 (1.0000, 0.9744, 0.9176) |
| 2201 | 1.0000 (0.1318, 1.0000, 0.8516) | < 0.0001 (< 0.0001, 1.0000, 0.7360) |
| 2202 | < 0.0001 (0.1976, 0.0060, 0.9978) | 0.4664 (< 0.0001, 0.7147, 0.8951) |
| 2203 | < 0.0001 (0.0587, 0.0060, 0.9969) | < 0.0001 (1.0000, 1.0000, 0.4714) |
| 2204 | 1.0000 (0.0052, 1.0000, 0.9816) | < 0.0001 (< 0.0001, 1.0000, 0.7787) |
| 2205 | < 0.0001 (0.7130, 0.0056, 0.9994) | < 0.0001 (< 0.0001, 1.0000, 0.8919) |
| 2206 | 1.3009 (0.4992, 0.3609, 0.9999) | < 0.0001 (< 0.0001, 1.0000, 0.7887) |
| 2207 | < 0.0001 (0.3573, 0.0060, 0.9947) | < 0.0001 (1.0000, 1.0000, 0.9822) |
| 2208 | 0.3104 (0.2581, 0.0736, 0.7545) | 0.5000 (1.0000, 0.8889, 0.9961) |
| **2209** | **12.5737 (**0.1312**, < 0.0001, < 0.0001)** | **2.6397 (< 0.0001, 0.0274, 0.0054)** |
| 2210 | < 0.0001 (0.2326, 0.0083, 1.0000) | 0.4382 (1.0000, 0.9072, 0.4558) |
| 2211 | < 0.0001 (0.6667, 0.0397, 1.0000) | 1.2012 (1.0000, 0.6764, 0.6952) |
| 2212 | 1.0000 (0.4445, 1.0000, 0.9216) | < 0.0001 (< 0.0001, 1.0000, 0.5895) |
| 2213 | 1.3946 (0.1760, 0.2645, 0.9996) | 0.4541 (1.0000, 0.9025, 0.9992) |
| 2214 | 0.3187 (< 0.0001, 0.0794, 0.9983) | 1.3388 (0.9011, 0.9843, 0.9156) |
| 2215 | < 0.0001 (0.3624, 0.0050, 1.0000) | < 0.0001 (< 0.0001, 1.0000, 0.4977) |
| 2216 | 0.7789 (0.1317, 0.3563, 0.4561) | < 0.0001 (1.0000, 1.0000, 0.9347) |
| 2217 | < 0.0001 (0.6783, 0.0117, 0.9999) | 0.3994 (1.0000, 0.9185, 0.7810) |
| 2218 | < 0.0001 (0.3623, 0.0087, 0.9972) | < 0.0001 (0.9548, 1.0000, 0.7604) |
| 2219 | 1.0000 (0.1731, 1.0000, 0.9798) | < 0.0001 (1.0000, 1.0000, 0.9925) |
| 2220 | 1.0000 (1.0000, 1.0000, 0.9870) | < 0.0001 (1.0000, 1.0000, 0.9981) |
| 2221 | 0.3188 (0.0467, 0.0795, 0.9992) | < 0.0001 (1.0000, 1.0000, 0.9960) |
| 2222 | < 0.0001 (0.9919, 0.0073, 0.9961) | < 0.0001 (< 0.0001, 1.0000, 0.9554) |
| 2223 | < 0.0001 (0.0338, 0.0083, 1.0000) | < 0.0001 (1.0000, 1.0000, 0.9477) |
| 2224 | 0.7225 (0.2964, 0.3136, 0.9890) | 0.5000 (0.9877, 0.6667, 0.8512) |
| 2225 | < 0.0001 (0.0081, 0.0060, 1.0000) | < 0.0001 (0.8999, 1.0000, 0.5378) |
| 2226 | < 0.0001 (0.3622, 0.0050, 0.9998) | < 0.0001 (0.6911, 1.0000, 0.9708) |
| 2227 | < 0.0001 (0.0392, 0.0060, 0.9969) | < 0.0001 (< 0.0001, 1.0000, 0.9902) |
| 2228 | < 0.0001 (0.0476, 0.0060, 1.0000) | 0.5161 (1.0000, 0.8841, 0.8921) |
| 2229 | < 0.0001 (< 0.0001, 0.0061, 1.0000) | 1.8072 (1.0000, 0.4481, 0.8142) |
| 2230 | < 0.0001 (0.9554, 0.0060, 0.9853) | 0.4685 (1.0000, 0.8982, 0.5242) |
| 2231 | 1.0000 (< 0.0001, 1.0000, 0.9870) | < 0.0001 (0.6548, 1.0000, 0.7182) |
| 2232 | < 0.0001 (< 0.0001, 0.0397, 0.9960) | 0.4981 (1.0000, 0.8526, 0.9985) |
| 2233 | < 0.0001 (0.9861, 0.0424, 0.9996) | 2.5000 (1.0000, 0.3512, 0.8130) |
| 2234 | < 0.0001 (0.2583, 0.0126, 0.9999) | < 0.0001 (1.0000, 1.0000, 0.2341) |
| 2235 | < 0.0001 (0.2965, 0.0107, 0.9993) | < 0.0001 (0.7450, 1.0000, 0.8402) |
| 2236 | < 0.0001 (< 0.0001, 0.0126, 0.9999) | < 0.0001 (1.0000, 1.0000, 0.9705) |
| 2237 | < 0.0001 (1.0000, 0.0126, 1.0000) | < 0.0001 (1.0000, 1.0000, 0.9957) |
| 2238 | < 0.0001 (< 0.0001, 0.0397, 0.9991) | < 0.0001 (1.0000, 1.0000, 0.8365) |
| 2239 | 1.0000 (0.0391, 1.0000, 0.9870) | < 0.0001 (0.9676, 1.0000, 0.9820) |
| 2240 | < 0.0001 (0.7130, 0.0060, 0.9809) | < 0.0001 (1.0000, 1.0000, 0.9916) |
| 2241 | 1.0000 (0.7130, 1.0000, 0.9525) | < 0.0001 (< 0.0001, 1.0000, 0.9456) |
| 2242 | < 0.0001 (0.3527, 0.0425, 0.9998) | < 0.0001 (0.9548, 1.0000, 0.8736) |
| 2243 | < 0.0001 (0.3625, 0.0060, 0.9808) | < 0.0001 (0.6667, 1.0000, 0.7904) |
| 2244 | 1.0000 (0.0126, 1.0000, 0.8619) | < 0.0001 (1.0000, 1.0000, 0.9866) |
| 2245 | < 0.0001 (< 0.0001, 0.0073, 0.9894) | < 0.0001 (1.0000, 1.0000, 0.9790) |
| 2246 | < 0.0001 (1.0000, 0.0093, 1.0000) | 0.9990 (< 0.0001, 0.4453, 0.9995) |
| 2247 | < 0.0001 (< 0.0001, 0.0073, 0.9894) | 1.0323 (0.8889, 0.7312, 0.4006) |
| 2248 | 0.6337 (0.7130, 0.2427, 0.7357) | < 0.0001 (< 0.0001, 1.0000, 0.6654) |
| 2249 | < 0.0001 (0.0016, 0.0343, 0.9999) | < 0.0001 (1.0000, 1.0000, 0.9133) |
| 2250 | < 0.0001 (< 0.0001, 0.0085, 0.9915) | < 0.0001 (< 0.0001, 1.0000, 0.9919) |
| 2251 | < 0.0001 (0.9977, 0.0120, 1.0000) | < 0.0001 (1.0000, 1.0000, 0.9963) |
| 2252 | < 0.0001 (0.6667, 0.0343, 0.9817) | 0.5000 (< 0.0001, 0.8889, 0.9445) |
| 2253 | < 0.0001 (0.6455, 0.0050, 0.9989) | < 0.0001 (< 0.0001, 1.0000, 0.8080) |
| 2254 | < 0.0001 (0.2963, 0.0065, 0.9976) | < 0.0001 (< 0.0001, 1.0000, 0.8438) |
| 2255 | < 0.0001 (0.1977, 0.0053, 0.9980) | 1.0000 (1.0000, 0.6804, 0.9894) |
| 2256 | 1.0000 (< 0.0001, 1.0000, 0.9230) | < 0.0001 (< 0.0001, 1.0000, 0.7117) |
| 2257 | < 0.0001 (0.0174, 0.0425, 0.9992) | < 0.0001 (< 0.0001, 1.0000, 0.9722) |
| 2258 | < 0.0001 (0.6911, 0.0061, 0.9987) | < 0.0001 (1.0000, 1.0000, 0.9447) |
| **2259** | **8.1898 (**0.5084, **< 0.0001, 0.0002)** | 2.5940 (1.0000, 1.0000, **0.0197**) |
| 2260 | < 0.0001 (< 0.0001, 0.0117, 0.9993) | < 0.0001 (1.0000, 1.0000, 0.9776) |
| 2261 | < 0.0001 (0.9954, 0.0425, 0.9999) | < 0.0001 (1.0000, 1.0000, 0.7004) |
| 2262 | < 0.0001 (1.0000, 0.0060, 0.9808) | < 0.0001 (1.0000, 1.0000, 0.8352) |
| 2263 | < 0.0001 (0.2969, 0.0087, 0.9972) | < 0.0001 (1.0000, 1.0000, 0.9234) |
| 2264 | 0.3480 (0.0003, 0.1006, 0.9995) | < 0.0001 (1.0000, 1.0000, 0.8352) |
| 2265 | 0.8949 (1.0000, 0.4105, 0.6784) | < 0.0001 (< 0.0001, 1.0000, 0.8735) |
| 2266 | < 0.0001 (0.6944, 0.0050, 0.9874) | < 0.0001 (< 0.0001, 1.0000, 0.9997) |
| 2267 | < 0.0001 (0.5084, 0.0126, 0.9986) | 1.0002 (1.0000, 0.4444, 0.9969) |
| 2268 | 1.0000 (0.9998, 1.0000, 0.9525) | < 0.0001 (1.0000, 1.0000, 0.9987) |
| 2269 | 1.1168 (< 0.0001, 0.4142, 0.7546) | < 0.0001 (0.6810, 1.0000, 0.5495) |
| 2270 | 1.0000 (0.1317, 1.0000, 0.9480) | < 0.0001 (< 0.0001, 1.0000, 0.8757) |
| 2271 | < 0.0001 (< 0.0001, 0.0083, 0.9987) | < 0.0001 (0.6836, 1.0000, 0.9751) |
| 2272 | < 0.0001 (0.0261, 0.0061, 0.9988) | < 0.0001 (< 0.0001, 1.0000, 0.9971) |
| 2273 | 1.0000 (0.3420, 1.0000, 0.8868) | 0.0833 (< 0.0001, 0.9995, 0.9990) |
| 2274 | < 0.0001 (< 0.0001, 0.0053, 0.9980) | 0.7985 (1.0000, 0.8022, 0.9916) |
| 2275 | < 0.0001 (0.9947, 0.0073, 0.9894) | 1.4998 (1.0000, 0.2964, 0.9994) |
| 2276 | 1.0000 (0.4446, 1.0000, 0.9870) | < 0.0001 (1.0000, 1.0000, 0.3049) |
| 2277 | < 0.0001 (< 0.0001, 0.0056, 0.9974) | < 0.0001 (1.0000, 1.0000, 0.1626) |
| 2278 | 1.0000 (0.3259, 1.0000, 0.9798) | < 0.0001 (1.0000, 1.0000, 0.9737) |
| 2279 | < 0.0001 (0.6667, 0.0425, 0.9969) | 0.2500 (1.0000, 0.9630, 0.9895) |
| 2280 | 0.3245 (0.3623, 0.0835, 0.9999) | 0.1665 (< 0.0001, 0.9877, 0.9739) |
| 2281 | 1.9050 (0.0590, 0.0772, 0.2318) | < 0.0001 (0.6953, 1.0000, 0.8462) |
| 2282 | < 0.0001 (< 0.0001, 0.0050, 0.9963) | < 0.0001 (< 0.0001, 1.0000, 0.9941) |
| 2283 | 0.3166 (0.6784, 0.0779, 0.9515) | 1.0001 (1.0000, 0.7407, 0.9738) |
| 2284 | 0.2786 (0.6667, 0.0528, 0.7574) | 0.2500 (0.9996, 0.9630, 0.9979) |
| 2285 | < 0.0001 (< 0.0001, 0.0065, 0.9894) | < 0.0001 (1.0000, 1.0000, 0.6432) |
| 2286 | 0.4401 (< 0.0001, 0.0897, 0.8333) | < 0.0001 (1.0000, 1.0000, 0.8994) |
| 2287 | 0.2798 (< 0.0001, 0.0535, 0.9680) | 0.8101 (0.0262, 0.9987, 0.9886) |
| 2288 | 1.6788 (0.3420, 0.1216, 0.3855) | 10.3525 (1.0000, 0.0019, 0.9927) |
| 2289 | < 0.0001 (0.1975, 0.0397, 0.9903) | < 0.0001 (1.0000, 1.0000, 0.6431) |
| 2290 | 0.3890 (< 0.0001, 0.1316, 0.6137) | < 0.0001 (1.0000, 1.0000, 1.0000) |
| 2291 | 1.0000 (0.0385, 1.0000, 0.9343) | < 0.0001 (1.0000, 1.0000, 0.7005) |
| 2292 | < 0.0001 (0.2585, 0.0343, 0.9964) | < 0.0001 (1.0000, 1.0000, 0.9958) |
| 2293 | 1.0000 (< 0.0001, 1.0000, 0.9342) | < 0.0001 (1.0000, 1.0000, 0.8404) |
| 2294 | 1.5932 (0.9958, 0.1672, 0.4885) | < 0.0001 (< 0.0001, 1.0000, 0.9903) |
| 2295 | < 0.0001 (0.4602, 0.0073, 0.9999) | 0.4986 (1.0000, 0.8895, 0.9996) |
| 2296 | 1.3947 (0.1436, 0.2644, 0.5157) | 1.9975 (1.0000, 0.4614, 0.9776) |
| **2297** | **4.4765** (0.2964, < **0.0001**, < **0.0001**) | < 0.0001 (< 0.0001, 1.0000, 0.8049) |
| 2298 | 0.3358 (0.8900, 0.0916, 0.8851) | 0.2500 (1.0000, 0.9630, 0.2764) |
| 2299 | 2.1560 (0.4561, 0.0359, 0.1418) | < 0.0001 (1.0000, 1.0000, 0.8402) |
| 2300 | < 0.0001 (0.6667, 0.0117, 1.0000) | < 0.0001 (1.0000, 1.0000, 0.9038) |
| 2301 | 0.5575 (0.6784, 0.1802, 0.9425) | < 0.0001 (0.9630, 1.0000, 0.8352) |
| 2302 | 2.7044 (0.5928, 0.0016, 0.2809) | < 0.0001 (1.0000, 1.0000, 0.9708) |
| 2303 | 0.3173 (0.1842, 0.0784, 0.9592) | 0.3999 (0.0041, 0.9184, 0.9692) |
| 2304 | 0.3358 (0.9919, 0.0916, 0.7046) | < 0.0001 (< 0.0001, 1.0000, 0.9925) |
| 2305 | 0.3891 (< 0.0001, 0.1317, 0.8826) | < 0.0001 (1.0000, 1.0000, 0.7604) |
| 2306 | 0.3994 (0.2865, 0.1396, 0.9910) | < 0.0001 (1.0000, 1.0000, 0.9816) |
| 2307 | < 0.0001 (0.4686, 0.0424, 1.0000) | < 0.0001 (0.7961, 1.0000, 0.9805) |
| 2308 | < 0.0001 (0.8002, 0.0065, 0.9993) | < 0.0001 (1.0000, 1.0000, 0.9927) |
| 2309 | < 0.0001 (0.0117, 0.0397, 0.9982) | < 0.0001 (< 0.0001, 1.0000, 0.9964) |
| 2310 | < 0.0001 (0.0416, 0.0041, 0.9905) | < 0.0001 (< 0.0001, 1.0000, 0.7924) |
| 2311 | < 0.0001 (0.1318, 0.0093, 1.0000) | < 0.0001 (< 0.0001, 1.0000, 0.9537) |
| 2312 | 0.3188 (0.0261, 0.0794, 0.9815) | < 0.0001 (1.0000, 1.0000, 0.7324) |
| 2313 | < 0.0001 (0.4602, 0.0050, 0.9951) | < 0.0001 (1.0000, 1.0000, 0.9999) |
| 2314 | < 0.0001 (0.2242, 0.0061, 0.9960) | < 0.0001 (< 0.0001, 1.0000, 0.9976) |
| 2315 | 1.0000 (0.3527, 1.0000, 0.9798) | < 0.0001 (< 0.0001, 1.0000, 0.8921) |
| 2316 | 0.4045 (0.6746, 0.1436, 0.9608) | < 0.0001 (1.0000, 1.0000, 0.9495) |
| 2317 | 1.3481 (0.9905, 0.3438, 0.6794) | < 0.0001 (0.9959, 1.0000, 0.7435) |
| 2318 | 0.4164 (1.0000, 0.1528, 0.9323) | 0.3337 (1.0000, 0.9989, 0.7810) |
| 2319 | < 0.0001 (0.1317, 0.0061, 0.9998) | < 0.0001 (< 0.0001, 1.0000, 0.9688) |
| 2320 | 0.4570 (0.0879, 0.1686, 0.9944) | < 0.0001 (1.0000, 1.0000, 0.7996) |
| 2321 | 2.7246 (0.7130, 0.0050, 0.2096) | < 0.0001 (0.9731, 1.0000, 0.9915) |
| 2322 | 1.0000 (< 0.0001, 1.0000, 0.9490) | < 0.0001 (1.0000, 1.0000, 0.8438) |
| 2323 | < 0.0001 (< 0.0001, 0.0061, 0.9958) | < 0.0001 (< 0.0001, 1.0000, 0.9987) |
| 2324 | < 0.0001 (0.7130, 0.0083, 1.0000) | < 0.0001 (1.0000, 1.0000, 0.7004) |
| 2325 | 1.0000 (0.7130, 1.0000, 0.8956) | < 0.0001 (0.6667, 1.0000, 0.9912) |
| 2326 | < 0.0001 (< 0.0001, 0.0050, 0.9941) | < 0.0001 (1.0000, 1.0000, 0.9912) |
| 2327 | 0.3611 (0.2385, 0.1103, 0.9997) | < 0.0001 (1.0000, 1.0000, 0.9969) |
| 2328 | < 0.0001 (< 0.0001, 0.0425, 0.9973) | < 0.0001 (0.8927, 1.0000, 0.9907) |
| 2329 | < 0.0001 (0.0051, 0.0424, 0.9937) | < 0.0001 (1.0000, 1.0000, 0.9965) |
| 2330 | 1.0000 (0.8889, 1.0000, 0.9431) | < 0.0001 (1.0000, 1.0000, 0.9347) |
| 2331 | < 0.0001 (0.2963, 0.0099, 0.9998) | < 0.0001 (1.0000, 1.0000, 0.9963) |
| 2332 | < 0.0001 (0.6667, 0.0397, 0.9991) | < 0.0001 (0.9716, 1.0000, 0.9537) |
| 2333 | < 0.0001 (0.0260, 0.0060, 0.9999) | < 0.0001 (1.0000, 1.0000, 0.7904) |
| 2334 | 0.6030 (< 0.0001, 0.2175, 0.6855) | < 0.0001 (1.0000, 1.0000, 0.9786) |
| 2335 | 0.8101 (< 0.0001, 0.3789, 0.9066) | < 0.0001 (< 0.0001, 1.0000, 0.9438) |
| 2336 | < 0.0001 (0.4160, 0.0065, 0.9651) | < 0.0001 (0.9630, 1.0000, 0.9958) |
| 2337 | < 0.0001 (< 0.0001, 0.0142, 0.9998) | < 0.0001 (1.0000, 1.0000, 0.9668) |
| 2338 | < 0.0001 (0.5084, 0.0060, 1.0000) | < 0.0001 (< 0.0001, 1.0000, 0.9135) |
| 2339 | < 0.0001 (< 0.0001, 0.0053, 0.9905) | < 0.0001 (< 0.0001, 1.0000, 0.9895) |
| 2340 | 0.3013 (< 0.0001, 0.0674, 0.9815) | 0.5000 (1.0000, 0.6667, 0.9999) |
| 2341 | < 0.0001 (0.9996, 0.0131, 0.9963) | < 0.0001 (< 0.0001, 1.0000, 0.9892) |
| 2342 | < 0.0001 (0.9986, 0.0397, 0.9991) | < 0.0001 (1.0000, 1.0000, 0.5393) |
| 2343 | 1.2075 (0.6993, 0.3575, 0.7216) | < 0.0001 (1.0000, 1.0000, 0.9971) |
| 2344 | < 0.0001 (0.2238, 0.0131, 0.9963) | < 0.0001 (1.0000, 1.0000, 0.9902) |
| 2345 | 1.0000 (0.5537, 1.0000, 0.9816) | < 0.0001 (< 0.0001, 1.0000, 0.9995) |
| 2346 | < 0.0001 (1.0000, 0.0053, 0.9905) | < 0.0001 (0.0264, 1.0000, 0.9997) |
| 2347 | 0.3481 (0.6667, 0.1006, 0.9583) | < 0.0001 (1.0000, 1.0000, 0.9400) |
| 2348 | < 0.0001 (< 0.0001, 0.0343, 0.9817) | < 0.0001 (< 0.0001, 1.0000, 0.9987) |
| 2349 | 1.0000 (1.0000, 1.0000, 0.9480) | 1.4999 (0.8889, 0.5926, 0.9398) |
| 2350 | < 0.0001 (0.0279, 0.0037, 0.9947) | < 0.0001 (1.0000, 1.0000, 0.9462) |
| 2351 | 1.0000 (< 0.0001, 1.0000, 0.9480) | < 0.0001 (1.0000, 1.0000, 0.5127) |
| 2352 | < 0.0001 (0.3625, 0.0425, 0.9969) | < 0.0001 (1.0000, 1.0000, 0.8402) |
| 2353 | < 0.0001 (0.7901, 0.0117, 1.0000) | < 0.0001 (< 0.0001, 1.0000, 0.9725) |
| 2354 | < 0.0001 (0.1127, 0.0050, 1.0000) | < 0.0001 (1.0000, 1.0000, 1.0000) |
| 2355 | < 0.0001 (0.1169, 0.0126, 1.0000) | < 0.0001 (1.0000, 1.0000, 0.9537) |
| 2356 | 1.2828 (< 0.0001, 0.3164, 0.3682) | < 0.0001 (1.0000, 1.0000, 0.8911) |
| 2357 | < 0.0001 (0.0260, 0.0313, 0.9862) | < 0.0001 (1.0000, 1.0000, 0.9448) |
| 2358 | < 0.0001 (0.4602, 0.0056, 0.9876) | < 0.0001 (1.0000, 1.0000, 0.9449) |
| 2359 | 0.7788 (0.3620, 0.3562, 0.9952) | < 0.0001 (< 0.0001, 1.0000, 0.9377) |
| 2360 | < 0.0001 (0.6882, 0.0093, 0.9999) | < 0.0001 (< 0.0001, 1.0000, 0.9887) |
| 2361 | < 0.0001 (0.6669, 0.0117, 1.0000) | 1.0007 (1.0000, 0.9978, 0.7004) |
| 2362 | 0.3480 (0.3625, 0.1006, 1.0000) | < 0.0001 (< 0.0001, 1.0000, 0.9897) |
| 2363 | 1.6279 (< 0.0001, 0.1570, 0.1916) | < 0.0001 (1.0000, 1.0000, 0.5927) |
| 2364 | 1.3952 (0.6667, 0.2642, 0.0885) | < 0.0001 (< 0.0001, 1.0000, 0.9962) |
| 2365 | < 0.0001 (0.0723, 0.0060, 0.9991) | < 0.0001 (1.0000, 1.0000, 0.8341) |
| 2366 | 0.6973 (0.1317, 0.2939, 0.9690) | < 0.0001 (< 0.0001, 1.0000, 0.9791) |
| 2367 | < 0.0001 (0.7113, 0.0120, 1.0000) | < 0.0001 (< 0.0001, 1.0000, 0.9986) |
| 2368 | 0.6377 (0.4444, 0.2460, 0.8216) | < 0.0001 (0.9998, 1.0000, 0.9504) |
| 2369 | 2.4021 (< 0.0001, 0.0306, 0.9313) | < 0.0001 (1.0000, 1.0000, 0.9959) |
| 2370 | < 0.0001 (0.6667, 0.0424, 1.0000) | 0.5033 (0.8945, 0.8984, 0.9437) |
| 2371 | < 0.0001 (0.7406, 0.0414, 1.0000) | < 0.0001 (< 0.0001, 1.0000, 0.9687) |
| 2372 | 1.2788 (0.0586, 0.3184, 0.5951) | < 0.0001 (1.0000, 1.0000, 0.7698) |
| 2373 | 0.3186 (< 0.0001, 0.0793, 0.9524) | < 0.0001 (< 0.0001, 1.0000, 0.9984) |
| 2374 | < 0.0001 (< 0.0001, 0.0060, 1.0000) | < 0.0001 (< 0.0001, 1.0000, 0.9668) |
| 2375 | < 0.0001 (< 0.0001, 0.0087, 1.0000) | 0.2500 (< 0.0001, 0.9630, 0.9444) |
| 2376 | 0.6994 (1.0000, 0.3520, 0.8781) | < 0.0001 (< 0.0001, 1.0000, 0.8512) |
| **2377** | **10.0837 (**0.9998**, < 0.0001, < 0.0001)** | **13.8961 (0.0019, 0.0019, < 0.0001)** |
| 2378 | 1.0000 (0.9993, 1.0000, 0.9174) | < 0.0001 (< 0.0001, 1.0000, 0.9668) |
| 2379 | < 0.0001 (< 0.0001, 0.0083, 1.0000) | < 0.0001 (1.0000, 1.0000, 0.4556) |
| 2380 | 1.0000 (0.5083, 1.0000, 0.8886) | < 0.0001 (< 0.0001, 1.0000, 0.9708) |
| 2381 | < 0.0001 (0.1243, 0.0126, 0.9972) | < 0.0001 (< 0.0001, 1.0000, 0.9958) |
| 2382 | < 0.0001 (0.0339, 0.0050, 1.0000) | < 0.0001 (1.0000, 1.0000, 0.8749) |
| 2383 | < 0.0001 (0.2584, 0.0083, 0.9999) | < 0.0001 (1.0000, 1.0000, 0.9763) |
| 2384 | 0.6029 (< 0.0001, 0.2175, 0.9930) | < 0.0001 (1.0000, 1.0000, 0.9900) |
| 2385 | 2.0943 (0.7825, 0.0536, 0.6269) | < 0.0001 (0.9821, 1.0000, 0.7653) |
| 2386 | 1.0000 (0.9998, 1.0000, 0.9490) | 0.2930 (< 0.0001, 0.9819, 0.9827) |
| 2387 | 0.3247 (0.3624, 0.0835, 0.9943) | < 0.0001 (0.9012, 1.0000, 0.9790) |
| 2388 | 0.3190 (< 0.0001, 0.0796, 0.9458) | 0.1250 (< 0.0001, 0.9959, 0.9572) |
| 2389 | 0.9036 (0.7922, 0.4292, 0.7471) | < 0.0001 (< 0.0001, 1.0000, 0.9916) |
| 2390 | < 0.0001 (0.2964, 0.0126, 1.0000) | < 0.0001 (1.0000, 1.0000, 0.9975) |
| 2391 | < 0.0001 (1.0000, 0.0126, 1.0000) | < 0.0001 (1.0000, 1.0000, 0.9687) |
| 2392 | 0.7785 (0.2963, 0.3560, 0.9468) | < 0.0001 (1.0000, 1.0000, 0.9704) |
| 2393 | 0.7073 (0.2583, 0.3017, 0.9615) | < 0.0001 (1.0000, 1.0000, 0.9668) |
| 2394 | < 0.0001 (0.1317, 0.0099, 0.9954) | < 0.0001 (1.0000, 1.0000, 0.8473) |
| 2395 | < 0.0001 (1.0000, 0.0142, 0.9993) | < 0.0001 (1.0000, 1.0000, 0.9925) |
| 2396 | < 0.0001 (0.7130, 0.0061, 0.9999) | < 0.0001 (1.0000, 1.0000, 0.8921) |
| 2397 | 1.0000 (0.2963, 1.0000, 0.8837) | < 0.0001 (1.0000, 1.0000, 0.7810) |
| 2398 | 1.0000 (0.1317, 1.0000, 0.9870) | < 0.0001 (1.0000, 1.0000, 0.9198) |
| 2399 | < 0.0001 (1.0000, 0.0083, 0.9921) | 0.4540 (0.7960, 0.9025, 0.9887) |
| 2400 | < 0.0001 (0.7130, 0.0093, 1.0000) | < 0.0001 (0.9998, 1.0000, 0.9965) |
| 2401 | < 0.0001 (0.4159, 0.0056, 1.0000) | < 0.0001 (< 0.0001, 1.0000, 0.8401) |
| 2402 | 1.0000 (< 0.0001, 1.0000, 0.9798) | < 0.0001 (1.0000, 1.0000, 0.7566) |
| 2403 | < 0.0001 (0.2963, 0.0343, 0.9964) | < 0.0001 (< 0.0001, 1.0000, 0.9909) |
| 2404 | 1.0000 (0.0392, 1.0000, 0.8455) | < 0.0001 (1.0000, 1.0000, 0.8921) |
| 2405 | < 0.0001 (0.6667, 0.0073, 0.9894) | < 0.0001 (1.0000, 1.0000, 0.9988) |
| 2406 | < 0.0001 (< 0.0001, 0.0061, 0.9988) | < 0.0001 (1.0000, 1.0000, 0.9688) |
| 2407 | 0.2228 (0.1760, 0.0232, 0.9357) | < 0.0001 (1.0000, 1.0000, 0.9708) |
| 2408 | < 0.0001 (0.4445, 0.0085, 1.0000) | < 0.0001 (0.0015, 1.0000, 0.7498) |
| 2409 | 1.0000 (0.4601, 1.0000, 0.9536) | < 0.0001 (1.0000, 1.0000, 0.9901) |
| 2410 | < 0.0001 (0.6667, 0.0061, 1.0000) | < 0.0001 (1.0000, 1.0000, 0.9987) |
| 2411 | < 0.0001 (0.5084, 0.0343, 0.9925) | < 0.0001 (< 0.0001, 1.0000, 0.7653) |
| 2412 | < 0.0001 (0.1313, 0.0095, 0.9998) | < 0.0001 (< 0.0001, 1.0000, 0.9692) |
| 2413 | < 0.0001 (0.1977, 0.0096, 0.9998) | < 0.0001 (1.0000, 1.0000, 0.7990) |
| 2414 | < 0.0001 (0.0878, 0.0050, 1.0000) | 0.3335 (< 0.0001, 0.9994, 0.9483) |
| 2415 | < 0.0001 (0.7130, 0.0061, 0.9998) | < 0.0001 (1.0000, 1.0000, 1.0000) |
| 2416 | < 0.0001 (< 0.0001, 0.0061, 0.9995) | < 0.0001 (1.0000, 1.0000, 0.8014) |
| 2417 | < 0.0001 (< 0.0001, 0.0060, 0.9947) | < 0.0001 (1.0000, 1.0000, 0.9096) |
| 2418 | < 0.0001 (1.0000, 0.0060, 1.0000) | < 0.0001 (1.0000, 1.0000, 0.7787) |
| 2419 | 1.0000 (0.2963, 1.0000, 0.9870) | 0.7255 (0.8810, 0.7925, 0.8969) |
| 2420 | < 0.0001 (0.4444, 0.0060, 0.9904) | 0.5161 (1.0000, 0.6596, 0.8921) |
| 2421 | < 0.0001 (0.6667, 0.0053, 0.9958) | < 0.0001 (1.0000, 1.0000, 0.7423) |
| 2422 | < 0.0001 (0.6882, 0.0061, 0.9807) | < 0.0001 (1.0000, 1.0000, 0.5561) |
| 2423 | < 0.0001 (0.0390, 0.0083, 1.0000) | < 0.0001 (0.6671, 1.0000, 0.7211) |
| 2424 | < 0.0001 (0.9728, 0.0083, 0.9948) | 4.1689 (1.0000, 0.6088, 0.9857) |
| 2425 | < 0.0001 (0.4360, 0.0065, 0.9857) | 0.9996 (0.6550, 0.4448, 0.9729) |
| 2426 | < 0.0001 (< 0.0001, 0.0056, 0.9984) | < 0.0001 (< 0.0001, 1.0000, 0.9167) |
| 2427 | < 0.0001 (1.0000, 0.0060, 1.0000) | < 0.0001 (1.0000, 1.0000, 0.3902) |
| 2428 | < 0.0001 (0.4445, 0.0083, 0.9996) | 0.5000 (< 0.0001, 0.8889, 0.8921) |
| 2429 | 0.3186 (< 0.0001, 0.0793, 0.9906) | 1.4052 (1.0000, 0.8106, 0.9708) |
| 2430 | < 0.0001 (< 0.0001, 0.0050, 0.9999) | 0.4919 (1.0000, 0.6970, 0.9803) |
| 2431 | < 0.0001 (< 0.0001, 0.0065, 0.9998) | < 0.0001 (1.0000, 1.0000, 0.4143) |
| 2432 | 0.3480 (0.9436, 0.1006, 0.9977) | < 0.0001 (< 0.0001, 1.0000, 0.4556) |
| 2433 | < 0.0001 (< 0.0001, 0.0093, 1.0000) | 0.9781 (1.0000, 0.4972, 0.9444) |
| 2434 | < 0.0001 (1.0000, 0.0073, 0.9961) | < 0.0001 (1.0000, 1.0000, 0.9537) |
| 2435 | < 0.0001 (< 0.0001, 0.0436, 1.0000) | < 0.0001 (< 0.0001, 1.0000, 0.2830) |
| 2436 | < 0.0001 (< 0.0001, 0.0073, 0.9894) | < 0.0001 (< 0.0001, 1.0000, 0.9913) |
| 2437 | 0.3510 (0.1306, 0.1027, 0.9973) | < 0.0001 (0.9998, 1.0000, 0.8442) |
| 2438 | 0.9051 (0.1842, 0.4303, 0.5766) | < 0.0001 (< 0.0001, 1.0000, 0.9737) |
| 2439 | 1.0000 (1.0000, 1.0000, 0.9490) | < 0.0001 (1.0000, 1.0000, 0.9923) |
| 2440 | < 0.0001 (0.5084, 0.0093, 0.9999) | 0.9549 (1.0000, 0.5048, 0.9788) |
| 2441 | < 0.0001 (< 0.0001, 0.0060, 1.0000) | < 0.0001 (< 0.0001, 1.0000, 0.9661) |
| 2442 | 1.0000 (0.0390, 1.0000, 0.9870) | < 0.0001 (< 0.0001, 1.0000, 0.3107) |
| 2443 | < 0.0001 (0.6667, 0.0343, 0.9967) | < 0.0001 (1.0000, 1.0000, 0.9965) |
| 2444 | < 0.0001 (0.6667, 0.0060, 0.9904) | < 0.0001 (1.0000, 1.0000, 0.9524) |
| 2445 | < 0.0001 (0.7407, 0.0060, 0.9896) | < 0.0001 (0.9959, 1.0000, 0.9876) |
| 2446 | < 0.0001 (0.2963, 0.0083, 0.9772) | < 0.0001 (< 0.0001, 1.0000, 0.9962) |
| 2447 | < 0.0001 (0.1072, 0.0060, 0.9996) | < 0.0001 (0.9997, 1.0000, 0.9904) |
| 2448 | 1.0000 (0.9992, 1.0000, 0.9230) | < 0.0001 (0.9676, 1.0000, 0.8999) |
| 2449 | 0.6344 (0.6667, 0.2433, 0.9729) | < 0.0001 (< 0.0001, 1.0000, 0.9188) |
| 2450 | 0.6683 (0.4689, 0.2273, 0.7810) | < 0.0001 (1.0000, 1.0000, 0.9894) |
| 2451 | < 0.0001 (0.1042, 0.0424, 0.9999) | < 0.0001 (0.0006, 1.0000, 0.9349) |
| 2452 | < 0.0001 (0.1321, 0.0060, 0.9985) | < 0.0001 (1.0000, 1.0000, 0.9904) |
| 2453 | < 0.0001 (0.6950, 0.0397, 0.9995) | < 0.0001 (1.0000, 1.0000, 0.9693) |
| 2454 | < 0.0001 (0.4444, 0.0083, 1.0000) | < 0.0001 (1.0000, 1.0000, 0.9893) |
| 2455 | < 0.0001 (0.4444, 0.0107, 0.9960) | < 0.0001 (1.0000, 1.0000, 0.9993) |
| 2456 | < 0.0001 (0.6668, 0.0425, 0.9999) | < 0.0001 (< 0.0001, 1.0000, 0.7907) |
| 2457 | 0.3186 (0.4444, 0.0793, 0.9293) | < 0.0001 (1.0000, 1.0000, 0.8864) |
| 2458 | < 0.0001 (0.3349, 0.0061, 0.9988) | < 0.0001 (< 0.0001, 1.0000, 0.9717) |
| 2459 | 1.7492 (0.1840, 0.1266, 0.8318) | < 0.0001 (1.0000, 1.0000, 0.7819) |
| 2460 | < 0.0001 (0.0937, 0.0083, 1.0000) | < 0.0001 (1.0000, 1.0000, 0.9480) |
| 2461 | < 0.0001 (0.0878, 0.0083, 0.9999) | < 0.0001 (1.0000, 1.0000, 0.9012) |
| 2462 | < 0.0001 (0.7130, 0.0053, 0.9753) | < 0.0001 (1.0000, 1.0000, 0.9719) |
| 2463 | < 0.0001 (< 0.0001, 0.0093, 0.9974) | < 0.0001 (1.0000, 1.0000, 0.9903) |
| 2464 | 0.3778 (1.0000, 0.1230, 0.9967) | < 0.0001 (1.0000, 1.0000, 0.8856) |
| 2465 | < 0.0001 (< 0.0001, 0.0083, 0.9987) | < 0.0001 (1.0000, 1.0000, 0.9885) |
| 2466 | 0.3167 (0.6988, 0.0780, 0.7199) | < 0.0001 (1.0000, 1.0000, 0.9608) |
| 2467 | < 0.0001 (0.0821, 0.0050, 0.9997) | 0.4094 (1.0000, 0.9233, 0.9661) |
| 2468 | < 0.0001 (0.1310, 0.0083, 1.0000) | < 0.0001 (1.0000, 1.0000, 0.9895) |
| 2469 | < 0.0001 (0.0935, 0.0313, 0.9701) | < 0.0001 (0.9877, 1.0000, 0.7617) |
| 2470 | < 0.0001 (1.0000, 0.0073, 0.9985) | < 0.0001 (1.0000, 1.0000, 0.8947) |
| 2471 | < 0.0001 (0.1760, 0.0061, 0.9995) | 0.3333 (0.8983, 0.9547, 0.9717) |
| 2472 | 1.0000 (0.1976, 1.0000, 0.9216) | < 0.0001 (1.0000, 1.0000, 0.9831) |
| 2473 | 0.3169 (0.1975, 0.0781, 0.9917) | < 0.0001 (0.6811, 1.0000, 0.7511) |
| 2474 | < 0.0001 (0.3625, 0.0397, 0.9995) | < 0.0001 (0.7845, 1.0000, 0.9905) |
| 2475 | 2.5467 (1.0000, 0.0111, 0.9308) | < 0.0001 (< 0.0001, 1.0000, 0.9693) |
| 2476 | < 0.0001 (< 0.0001, 0.0050, 0.9991) | < 0.0001 (1.0000, 1.0000, 0.9893) |
| 2477 | 0.3187 (0.1168, 0.0794, 1.0000) | 0.1667 (1.0000, 0.9877, 0.9012) |
| 2478 | < 0.0001 (< 0.0001, 0.0061, 0.9997) | 0.2475 (1.0000, 0.9637, 0.9891) |
| 2479 | < 0.0001 (0.2963, 0.0061, 1.0000) | 0.9898 (1.0000, 0.6559, 0.9146) |
| 2480 | < 0.0001 (0.6667, 0.0083, 1.0000) | < 0.0001 (0.4560, 1.0000, 0.7145) |
| 2481 | 1.0000 (< 0.0001, 1.0000, 0.9569) | < 0.0001 (< 0.0001, 1.0000, 0.2735) |
| 2482 | < 0.0001 (0.0023, 0.0126, 0.9999) | < 0.0001 (< 0.0001, 1.0000, 0.8947) |
| 2483 | 1.0000 (0.0163, 1.0000, 0.9798) | < 0.0001 (1.0000, 1.0000, 0.9998) |
| 2484 | 1.0000 (< 0.0001, 1.0000, 0.9404) | 0.5136 (0.9920, 0.6605, 0.9814) |
| 2485 | 0.3065 (0.7130, 0.0710, 0.9209) | < 0.0001 (1.0000, 1.0000, 0.9714) |
| 2486 | < 0.0001 (1.0000, 0.0060, 0.9991) | < 0.0001 (< 0.0001, 1.0000, 0.6812) |
| 2487 | < 0.0001 (< 0.0001, 0.0061, 1.0000) | < 0.0001 (< 0.0001, 1.0000, 0.9990) |
| 2488 | 0.2786 (0.7831, 0.0528, 0.9443) | 0.6931 (0.9993, 0.8151, 0.9885) |
| 2489 | 1.0000 (0.7130, 1.0000, 0.8601) | < 0.0001 (1.0000, 1.0000, 0.9747) |
| 2490 | < 0.0001 (0.7620, 0.0083, 0.9994) | < 0.0001 (1.0000, 1.0000, 0.4033) |
| 2491 | 1.0000 (< 0.0001, 1.0000, 0.9798) | < 0.0001 (0.9675, 1.0000, 0.9983) |
| 2492 | < 0.0001 (0.4445, 0.0053, 0.9999) | 0.2039 (< 0.0001, 0.9757, 0.9905) |
| 2493 | < 0.0001 (0.1317, 0.0061, 0.9948) | < 0.0001 (1.0000, 1.0000, 0.9747) |
| 2494 | < 0.0001 (0.0260, 0.0050, 0.9951) | < 0.0001 (1.0000, 1.0000, 0.8670) |
| 2495 | < 0.0001 (0.9664, 0.0050, 0.9980) | 0.3333 (1.0000, 0.9547, 0.9429) |
| 2496 | < 0.0001 (0.2879, 0.0061, 0.9987) | 0.5000 (1.0000, 0.6667, 0.7404) |
| 2497 | < 0.0001 (0.6784, 0.0060, 1.0000) | < 0.0001 (1.0000, 1.0000, 0.7583) |
| 2498 | 1.0000 (0.6183, 1.0000, 0.9216) | < 0.0001 (< 0.0001, 1.0000, 0.4058) |
| 2499 | < 0.0001 (0.1317, 0.0126, 0.9999) | < 0.0001 (1.0000, 1.0000, 0.9693) |
| 2500 | 1.0000 (< 0.0001, 1.0000, 0.8871) | 0.5000 (1.0000, 0.8889, 0.9727) |
| 2501 | < 0.0001 (0.4890, 0.0099, 0.9853) | < 0.0001 (1.0000, 1.0000, 0.8009) |
| 2502 | < 0.0001 (0.9982, 0.0060, 1.0000) | < 0.0001 (1.0000, 1.0000, 0.8938) |
| 2503 | 0.2227 (< 0.0001, 0.0231, 0.9357) | < 0.0001 (1.0000, 1.0000, 0.8478) |
| 2504 | < 0.0001 (< 0.0001, 0.0085, 0.9915) | < 0.0001 (0.0005, 1.0000, 0.9765) |
| 2505 | < 0.0001 (0.0393, 0.0056, 0.9884) | < 0.0001 (1.0000, 1.0000, 0.9020) |
| 2506 | < 0.0001 (0.1318, 0.0056, 0.9822) | < 0.0001 (< 0.0001, 1.0000, 0.9430) |
| 2507 | 1.0000 (0.3356, 1.0000, 0.9798) | < 0.0001 (0.1463, 1.0000, 0.9378) |
| 2508 | 0.6498 (0.1975, 0.3213, 0.9808) | < 0.0001 (1.0000, 1.0000, 0.9349) |
| 2509 | 0.4046 (0.0116, 0.1437, 0.8214) | < 0.0001 (1.0000, 1.0000, 0.9012) |
| 2510 | < 0.0001 (0.0878, 0.0087, 0.9988) | < 0.0001 (< 0.0001, 1.0000, 0.9885) |
| 2511 | < 0.0001 (0.2966, 0.0425, 0.9995) | < 0.0001 (1.0000, 1.0000, 0.8999) |
| 2512 | < 0.0001 (0.3121, 0.0107, 0.9907) | < 0.0001 (1.0000, 1.0000, 0.9480) |
| **2513** | **10.2100 (**0.4486**, < 0.0001, 0.0081)** | **2.8259** (1.0000, 1.0000, **0.0408**) |
| 2514 | 3.3303 (0.5084, 0.0004, 0.2213) | < 0.0001 (0.0226, 1.0000, 0.9995) |
| 2515 | 1.0000 (0.7769, 1.0000, 0.9798) | < 0.0001 (1.0000, 1.0000, 0.9891) |
| 2516 | 0.3012 (0.7130, 0.0673, 0.9489) | < 0.0001 (< 0.0001, 1.0000, 0.9861) |
| 2517 | 0.7990 (0.2963, 0.3709, 0.9999) | < 0.0001 (< 0.0001, 1.0000, 0.9751) |
| 2518 | 2.8840 (0.0585, 0.0006, 0.1283) | < 0.0001 (< 0.0001, 1.0000, 0.9965) |
| 2519 | 0.3168 (0.6667, 0.0780, 0.8956) | < 0.0001 (0.0292, 1.0000, 0.9976) |
| 2520 | < 0.0001 (< 0.0001, 0.0053, 0.9905) | < 0.0001 (1.0000, 1.0000, 0.8947) |
| 2521 | 1.0000 (0.2198, 1.0000, 0.9870) | < 0.0001 (1.0000, 1.0000, 0.9571) |
| 2522 | 2.6986 (0.3625, 0.0080, 0.0234) | 0.4782 (1.0000, 0.7102, 0.9876) |
| **2523** | **2.2228 (**0.6684**, 0.0080, 0.0502)** | < 0.0001 (1.0000, 1.0000, 0.9877) |
| 2524 | 1.0000 (0.6667, 1.0000, 0.9552) | 0.4937 (0.8810, 0.6752, 0.5077) |
| 2525 | < 0.0001 (0.0002, 0.0060, 0.9947) | < 0.0001 (1.0000, 1.0000, 0.9299) |
| 2526 | < 0.0001 (0.4823, 0.0424, 0.9998) | < 0.0001 (< 0.0001, 1.0000, 0.3752) |
| 2527 | < 0.0001 (0.6667, 0.0060, 0.9966) | < 0.0001 (< 0.0001, 1.0000, 0.8020) |
| 2528 | < 0.0001 (0.1317, 0.0045, 0.9890) | < 0.0001 (< 0.0001, 1.0000, 0.8292) |
| 2529 | < 0.0001 (0.9334, 0.0056, 0.9913) | < 0.0001 (0.8889, 1.0000, 0.9693) |
| 2530 | < 0.0001 (0.2585, 0.0056, 0.9960) | < 0.0001 (1.0000, 1.0000, 0.9837) |
| 2531 | 1.0000 (1.0000, 1.0000, 0.9480) | < 0.0001 (1.0000, 1.0000, 0.9694) |
| 2532 | < 0.0001 (< 0.0001, 0.0060, 0.9987) | < 0.0001 (1.0000, 1.0000, 0.9747) |
| 2533 | < 0.0001 (1.0000, 0.0060, 0.9854) | < 0.0001 (1.0000, 1.0000, 0.9434) |
| 2534 | < 0.0001 (0.1790, 0.0085, 0.9980) | 0.4937 (1.0000, 0.6820, 0.9966) |
| 2535 | < 0.0001 (0.2684, 0.0050, 1.0000) | < 0.0001 (1.0000, 1.0000, 0.9480) |
| 2536 | 1.0000 (1.0000, 1.0000, 0.8401) | < 0.0001 (< 0.0001, 1.0000, 0.3753) |
| 2537 | < 0.0001 (0.1243, 0.0060, 0.9854) | 0.3335 (1.0000, 0.9996, 0.8545) |
| 2538 | < 0.0001 (1.0000, 0.0073, 0.9894) | < 0.0001 (< 0.0001, 1.0000, 0.9957) |
| 2539 | 1.0000 (0.4444, 1.0000, 0.8370) | < 0.0001 (< 0.0001, 1.0000, 0.7551) |
| 2540 | 1.0000 (0.4745, 1.0000, 0.9798) | 0.2375 (1.0000, 0.9700, 0.9963) |
| 2541 | 1.0000 (1.0000, 1.0000, 0.9379) | < 0.0001 (1.0000, 1.0000, 0.9921) |
| 2542 | < 0.0001 (0.4446, 0.0073, 0.9985) | < 0.0001 (1.0000, 1.0000, 0.9361) |
| 2543 | < 0.0001 (0.4445, 0.0044, 0.9995) | < 0.0001 (1.0000, 1.0000, 0.9990) |
| 2544 | 1.0000 (0.7130, 1.0000, 0.8956) | < 0.0001 (1.0000, 1.0000, 0.9745) |
| 2545 | < 0.0001 (0.0585, 0.0053, 0.9999) | < 0.0001 (< 0.0001, 1.0000, 0.9972) |
| 2546 | 0.6031 (0.2964, 0.2906, 0.9430) | < 0.0001 (1.0000, 1.0000, 0.9613) |
| 2547 | 1.0000 (0.9992, 1.0000, 0.9870) | < 0.0001 (1.0000, 1.0000, 0.9720) |
| 2548 | < 0.0001 (0.7130, 0.0050, 0.9989) | < 0.0001 (< 0.0001, 1.0000, 0.9995) |
| 2549 | 1.5872 (0.2965, 0.1690, 0.4484) | < 0.0001 (1.0000, 1.0000, 0.8999) |
| 2550 | < 0.0001 (0.2963, 0.0073, 0.9998) | < 0.0001 (1.0000, 1.0000, 0.9727) |
| 2551 | < 0.0001 (0.4444, 0.0073, 0.9987) | < 0.0001 (1.0000, 1.0000, 0.9693) |
| 2552 | < 0.0001 (0.0392, 0.0117, 0.9998) | < 0.0001 (< 0.0001, 1.0000, 0.9378) |
| 2553 | 0.9049 (0.6950, 0.4302, 0.8639) | < 0.0001 (1.0000, 1.0000, 0.9746) |
| 2554 | 0.2864 (0.1317, 0.0577, 0.9528) | 0.2500 (1.0000, 0.9630, 0.9930) |
| 2555 | < 0.0001 (0.9996, 0.0142, 0.9830) | < 0.0001 (1.0000, 1.0000, 0.9188) |
| 2556 | < 0.0001 (< 0.0001, 0.0085, 0.9980) | < 0.0001 (1.0000, 1.0000, 0.9097) |
| 2557 | 0.3166 (0.9764, 0.0779, 0.8901) | < 0.0001 (< 0.0001, 1.0000, 0.9694) |
| 2558 | < 0.0001 (0.5084, 0.0073, 0.9987) | < 0.0001 (0.9959, 1.0000, 0.9845) |
| 2559 | 0.3186 (0.3576, 0.0793, 0.9906) | < 0.0001 (1.0000, 1.0000, 0.8947) |
| 2560 | 0.3171 (0.2679, 0.0782, 0.9480) | 0.7131 (1.0000, 0.8267, 0.9717) |
| 2561 | 2.5471 (0.1671, 0.0111, 0.5525) | < 0.0001 (1.0000, 1.0000, 0.9984) |
| 2562 | 1.0000 (0.2584, 1.0000, 0.9351) | < 0.0001 (< 0.0001, 1.0000, 0.6726) |
| 2563 | < 0.0001 (0.7130, 0.0083, 1.0000) | < 0.0001 (1.0000, 1.0000, 0.9878) |
| 2564 | < 0.0001 (< 0.0001, 0.0065, 0.9933) | < 0.0001 (1.0000, 1.0000, 0.9299) |
| 2565 | < 0.0001 (0.1842, 0.0085, 0.9992) | < 0.0001 (< 0.0001, 1.0000, 0.8715) |
| 2566 | 1.6235 (< 0.0001, 0.1851, 0.6858) | < 0.0001 (1.0000, 1.0000, 0.7476) |
| 2567 | 0.6375 (0.3625, 0.2458, 1.0000) | < 0.0001 (1.0000, 1.0000, 0.9541) |
| 2568 | < 0.0001 (0.0033, 0.0061, 0.9997) | < 0.0001 (1.0000, 1.0000, 0.9693) |
| 2569 | < 0.0001 (0.0390, 0.0060, 0.9896) | < 0.0001 (1.0000, 1.0000, 0.9480) |
| 2570 | 2.6124 (0.1320, 0.0143, 0.5176) | 0.1250 (1.0000, 0.9959, 0.8122) |
| 2571 | < 0.0001 (0.2685, 0.0061, 1.0000) | < 0.0001 (1.0000, 1.0000, 0.9927) |
| 2572 | 0.5740 (0.6667, 0.1937, 0.9998) | < 0.0001 (< 0.0001, 1.0000, 0.9384) |
| 2573 | < 0.0001 (0.4444, 0.0060, 0.9896) | < 0.0001 (1.0000, 1.0000, 0.9774) |
| 2574 | 0.3358 (0.1317, 0.0916, 0.9866) | 0.2297 (1.0000, 0.9688, 0.9661) |
| 2575 | < 0.0001 (0.3623, 0.0056, 0.9696) | < 0.0001 (< 0.0001, 1.0000, 0.9746) |
| 2576 | 1.0000 (0.6667, 1.0000, 0.8264) | < 0.0001 (< 0.0001, 1.0000, 0.6982) |
| 2577 | 1.0493 (0.2116, 0.4671, 0.7825) | < 0.0001 (< 0.0001, 1.0000, 0.9877) |
| 2578 | 0.3013 (0.4823, 0.0674, 0.9863) | 0.5000 (0.6667, 0.6667, 0.9299) |
| 2579 | < 0.0001 (0.2964, 0.0060, 0.9854) | < 0.0001 (1.0000, 1.0000, 0.9219) |
| 2580 | < 0.0001 (0.6603, 0.0056, 0.9998) | < 0.0001 (< 0.0001, 1.0000, 0.5892) |
| 2581 | < 0.0001 (0.1843, 0.0099, 0.9932) | 0.4726 (1.0000, 0.8970, 1.0000) |
| 2582 | 1.4465 (0.6944, 0.2435, 0.7161) | < 0.0001 (< 0.0001, 1.0000, 0.9693) |
| 2583 | 1.0000 (0.7130, 1.0000, 0.8798) | < 0.0001 (1.0000, 1.0000, 0.7395) |
| 2584 | < 0.0001 (0.1317, 0.0107, 0.9863) | < 0.0001 (1.0000, 1.0000, 0.9933) |
| 2585 | 1.0000 (0.6667, 1.0000, 0.9411) | 0.4670 (1.0000, 0.8987, 0.9765) |
| 2586 | < 0.0001 (0.1975, 0.0056, 0.9696) | < 0.0001 (1.0000, 1.0000, 0.9717) |
| 2587 | < 0.0001 (< 0.0001, 0.0050, 0.9951) | < 0.0001 (< 0.0001, 1.0000, 0.7821) |
| 2588 | < 0.0001 (0.6882, 0.0061, 1.0000) | < 0.0001 (< 0.0001, 1.0000, 0.9573) |
| 2589 | 1.0000 (0.6743, 1.0000, 0.9379) | 0.2500 (1.0000, 0.9630, 0.9449) |
| 2590 | < 0.0001 (< 0.0001, 0.0425, 0.9973) | < 0.0001 (1.0000, 1.0000, 0.8717) |
| 2591 | 1.0000 (0.8891, 1.0000, 0.9342) | < 0.0001 (1.0000, 1.0000, 0.8877) |
| 2592 | 1.0000 (0.0391, 1.0000, 0.9798) | < 0.0001 (< 0.0001, 1.0000, 0.9724) |
| 2593 | < 0.0001 (0.6667, 0.0424, 1.0000) | < 0.0001 (1.0000, 1.0000, 0.7975) |
| 2594 | 0.3481 (0.3624, 0.1007, 1.0000) | < 0.0001 (1.0000, 1.0000, 0.8020) |
| 2595 | < 0.0001 (0.7130, 0.0073, 0.9985) | < 0.0001 (0.9628, 1.0000, 0.9708) |
| 2596 | < 0.0001 (1.0000, 0.0095, 0.9931) | < 0.0001 (1.0000, 1.0000, 0.8864) |
| 2597 | < 0.0001 (0.6667, 0.0041, 0.9905) | < 0.0001 (1.0000, 1.0000, 0.9891) |
| 2598 | < 0.0001 (< 0.0001, 0.0117, 1.0000) | < 0.0001 (0.9630, 1.0000, 0.9993) |
| 2599 | 0.7990 (0.6667, 0.3709, 0.9998) | < 0.0001 (0.9920, 1.0000, 0.9939) |
| 2600 | 1.0000 (< 0.0001, 1.0000, 0.9351) | < 0.0001 (< 0.0001, 1.0000, 0.9498) |
| 2601 | 1.0000 (0.0663, 1.0000, 0.9623) | < 0.0001 (1.0000, 1.0000, 0.9916) |
| 2602 | < 0.0001 (< 0.0001, 0.0083, 0.9949) | 0.5000 (< 0.0001, 0.8889, 0.9628) |
| 2603 | < 0.0001 (< 0.0001, 0.0060, 0.9999) | < 0.0001 (1.0000, 1.0000, 0.8952) |
| 2604 | 0.6336 (0.3356, 0.2427, 0.4854) | < 0.0001 (< 0.0001, 1.0000, 0.6326) |
| 2605 | 1.0000 (0.5706, 1.0000, 0.9525) | < 0.0001 (1.0000, 1.0000, 0.9719) |
| 2606 | 0.6344 (0.6667, 0.2433, 0.8173) | < 0.0001 (1.0000, 1.0000, 0.9717) |
| 2607 | 1.0000 (< 0.0001, 1.0000, 0.8612) | < 0.0001 (< 0.0001, 1.0000, 0.9964) |
| 2608 | 1.0000 (0.6667, 1.0000, 0.8565) | < 0.0001 (< 0.0001, 1.0000, 0.9020) |
| 2609 | < 0.0001 (0.0588, 0.0060, 0.9947) | < 0.0001 (1.0000, 1.0000, 0.9971) |
| 2610 | < 0.0001 (0.0235, 0.0087, 0.9927) | < 0.0001 (< 0.0001, 1.0000, 0.9962) |
| 2611 | < 0.0001 (0.4602, 0.0060, 0.9854) | < 0.0001 (1.0000, 1.0000, 0.9860) |
| 2612 | < 0.0001 (< 0.0001, 0.0044, 0.9995) | < 0.0001 (1.0000, 1.0000, 0.9988) |
| 2613 | < 0.0001 (0.4776, 0.0056, 0.9971) | < 0.0001 (1.0000, 1.0000, 0.9910) |
| 2614 | < 0.0001 (0.4180, 0.0056, 1.0000) | < 0.0001 (< 0.0001, 1.0000, 0.8947) |
| 2615 | 1.0000 (0.6993, 1.0000, 0.9798) | < 0.0001 (0.2080, 1.0000, 0.9661) |
| 2616 | 0.3533 (0.4830, 0.1045, 0.5279) | < 0.0001 (0.9103, 1.0000, 0.9523) |
| 2617 | < 0.0001 (0.1978, 0.0142, 0.9830) | < 0.0001 (1.0000, 1.0000, 0.8952) |
| 2618 | < 0.0001 (0.4992, 0.0142, 0.9998) | < 0.0001 (< 0.0001, 1.0000, 0.9873) |
| 2619 | 0.6692 (0.5082, 0.2282, 0.7800) | < 0.0001 (1.0000, 1.0000, 0.9480) |
| 2620 | < 0.0001 (0.0667, 0.0061, 0.9997) | < 0.0001 (1.0000, 1.0000, 0.9885) |
| 2621 | < 0.0001 (< 0.0001, 0.0050, 0.9988) | 0.0833 (1.0000, 0.9995, 0.8626) |
| 2622 | 1.2086 (0.0438, 0.3976, 0.9399) | < 0.0001 (< 0.0001, 1.0000, 0.8494) |
| 2623 | 0.3167 (0.3625, 0.0780, 0.9957) | 0.5194 (1.0000, 0.8831, 0.9999) |
| 2624 | < 0.0001 (0.6667, 0.0045, 0.9890) | < 0.0001 (0.6667, 1.0000, 0.9939) |
| 2625 | < 0.0001 (0.6784, 0.0083, 1.0000) | < 0.0001 (0.3158, 1.0000, 0.7444) |
| 2626 | < 0.0001 (< 0.0001, 0.0343, 0.9921) | < 0.0001 (< 0.0001, 1.0000, 0.8947) |
| 2627 | < 0.0001 (0.3349, 0.0073, 0.9985) | < 0.0001 (< 0.0001, 1.0000, 0.9983) |
| 2628 | < 0.0001 (0.4232, 0.0083, 1.0000) | < 0.0001 (0.0004, 1.0000, 0.8589) |
| 2629 | < 0.0001 (0.9989, 0.0044, 0.9891) | < 0.0001 (0.9900, 1.0000, 0.9299) |
| 2630 | < 0.0001 (1.0000, 0.0056, 0.9884) | < 0.0001 (< 0.0001, 1.0000, 0.9019) |
| 2631 | < 0.0001 (1.0000, 0.0142, 0.9979) | < 0.0001 (0.7005, 1.0000, 0.9299) |
| 2632 | 1.0000 (0.1319, 1.0000, 0.9490) | < 0.0001 (< 0.0001, 1.0000, 0.9977) |
| 2633 | < 0.0001 (0.4602, 0.0060, 0.9904) | < 0.0001 (1.0000, 1.0000, 0.8589) |
| 2634 | < 0.0001 (0.0663, 0.0056, 0.9991) | < 0.0001 (1.0000, 1.0000, 0.9993) |
| 2635 | < 0.0001 (0.2581, 0.0056, 0.9954) | < 0.0001 (1.0000, 1.0000, 0.8545) |
| 2636 | 0.3611 (< 0.0001, 0.1103, 0.9899) | < 0.0001 (1.0000, 1.0000, 0.9661) |
| 2637 | < 0.0001 (0.1975, 0.0060, 0.9947) | < 0.0001 (1.0000, 1.0000, 0.9996) |
| 2638 | < 0.0001 (1.0000, 0.0099, 0.9975) | 0.3507 (1.0000, 0.9504, 0.9965) |
| 2639 | 1.0000 (0.4775, 1.0000, 0.9351) | < 0.0001 (0.8773, 1.0000, 0.9679) |
| 2640 | < 0.0001 (< 0.0001, 0.0073, 0.9894) | 0.2327 (1.0000, 0.9680, 0.7200) |
| 2641 | < 0.0001 (< 0.0001, 0.0093, 0.9977) | < 0.0001 (< 0.0001, 1.0000, 0.8952) |
| 2642 | 0.6035 (0.0936, 0.2908, 0.9377) | < 0.0001 (1.0000, 1.0000, 0.7720) |
| 2643 | < 0.0001 (0.4445, 0.0425, 0.9969) | < 0.0001 (1.0000, 1.0000, 0.9661) |
| 2644 | 1.0000 (0.6603, 1.0000, 0.9525) | < 0.0001 (1.0000, 1.0000, 0.8952) |
| 2645 | 1.0000 (< 0.0001, 1.0000, 0.9174) | < 0.0001 (1.0000, 1.0000, 0.9938) |
| 2646 | < 0.0001 (0.1975, 0.0037, 0.9881) | < 0.0001 (1.0000, 1.0000, 0.9824) |
| 2647 | < 0.0001 (0.1977, 0.0142, 0.9763) | < 0.0001 (1.0000, 1.0000, 0.9613) |
| 2648 | < 0.0001 (0.6667, 0.0060, 0.9947) | < 0.0001 (1.0000, 1.0000, 0.9893) |
| 2649 | 1.0000 (0.0002, 1.0000, 0.9798) | < 0.0001 (< 0.0001, 1.0000, 0.9861) |
| 2650 | < 0.0001 (0.8889, 0.0053, 0.9905) | < 0.0001 (< 0.0001, 1.0000, 0.9542) |
| 2651 | < 0.0001 (0.6667, 0.0060, 0.9996) | < 0.0001 (1.0000, 1.0000, 0.9727) |
| 2652 | 0.3480 (< 0.0001, 0.1006, 0.9995) | 0.7363 (0.9676, 0.7696, 0.9997) |
| 2653 | 0.3016 (0.1791, 0.0676, 0.9916) | 0.0714 (< 0.0001, 0.9998, 0.9877) |
| 2654 | 1.0000 (0.7130, 1.0000, 0.9870) | < 0.0001 (0.9996, 1.0000, 0.8368) |
| 2655 | < 0.0001 (1.0000, 0.0060, 0.9969) | < 0.0001 (1.0000, 1.0000, 0.9965) |
| 2656 | 0.2786 (0.3625, 0.0528, 0.8733) | < 0.0001 (< 0.0001, 1.0000, 0.8999) |
| 2657 | < 0.0001 (1.0000, 0.0060, 0.9978) | < 0.0001 (1.0000, 1.0000, 0.9500) |
| 2658 | 1.0000 (0.0937, 1.0000, 0.9525) | < 0.0001 (< 0.0001, 1.0000, 0.9524) |
| 2659 | < 0.0001 (< 0.0001, 0.0065, 0.9856) | < 0.0001 (1.0000, 1.0000, 0.9943) |
| 2660 | 0.3485 (< 0.0001, 0.1010, 0.9923) | < 0.0001 (1.0000, 1.0000, 0.9931) |
| 2661 | < 0.0001 (0.1126, 0.0117, 1.0000) | < 0.0001 (1.0000, 1.0000, 0.9984) |
| 2662 | < 0.0001 (0.3413, 0.0142, 0.9979) | < 0.0001 (1.0000, 1.0000, 0.9288) |
| 2663 | 0.5842 (< 0.0001, 0.2021, 0.9034) | < 0.0001 (1.0000, 1.0000, 0.9955) |
| 2664 | < 0.0001 (< 0.0001, 0.0093, 0.9996) | < 0.0001 (< 0.0001, 1.0000, 0.9991) |
| 2665 | < 0.0001 (0.6950, 0.0107, 0.9863) | < 0.0001 (< 0.0001, 1.0000, 0.9897) |
| 2666 | < 0.0001 (0.4444, 0.0073, 0.9894) | < 0.0001 (< 0.0001, 1.0000, 0.8952) |
| 2667 | 1.4138 (0.6370, 0.2565, 0.8044) | < 0.0001 (< 0.0001, 1.0000, 0.9661) |
| 2668 | < 0.0001 (0.6784, 0.0087, 0.9926) | < 0.0001 (1.0000, 1.0000, 0.9541) |
| 2669 | < 0.0001 (0.4447, 0.0065, 0.9733) | < 0.0001 (1.0000, 1.0000, 0.9480) |
| 2670 | < 0.0001 (0.0468, 0.0050, 0.9963) | < 0.0001 (< 0.0001, 1.0000, 0.9859) |
| 2671 | 0.3992 (0.4830, 0.1395, 0.9976) | < 0.0001 (1.0000, 1.0000, 0.9917) |
| 2672 | < 0.0001 (0.6667, 0.0126, 0.9986) | 0.5133 (< 0.0001, 0.8849, 0.9530) |
| 2673 | 1.0000 (< 0.0001, 1.0000, 0.8511) | < 0.0001 (< 0.0001, 1.0000, 0.9627) |
| 2674 | < 0.0001 (< 0.0001, 0.0065, 0.9733) | < 0.0001 (1.0000, 1.0000, 0.8849) |
| 2675 | < 0.0001 (< 0.0001, 0.0060, 0.9957) | < 0.0001 (< 0.0001, 1.0000, 0.9753) |
| 2676 | 1.0000 (0.0665, 1.0000, 0.9216) | 0.4998 (1.0000, 0.6669, 0.9685) |
| 2677 | < 0.0001 (1.0000, 0.0073, 0.9987) | < 0.0001 (1.0000, 1.0000, 0.9859) |
| 2678 | < 0.0001 (< 0.0001, 0.0107, 1.0000) | 0.5134 (1.0000, 0.6608, 0.5741) |
| 2679 | < 0.0001 (0.5084, 0.0120, 0.9971) | < 0.0001 (1.0000, 1.0000, 0.9845) |
| 2680 | 1.0000 (< 0.0001, 1.0000, 0.9536) | < 0.0001 (< 0.0001, 1.0000, 0.6814) |
| 2681 | < 0.0001 (< 0.0001, 0.0037, 0.9987) | < 0.0001 (1.0000, 1.0000, 0.9299) |
| 2682 | < 0.0001 (0.1123, 0.0061, 0.9808) | < 0.0001 (1.0000, 1.0000, 0.9837) |
| 2683 | < 0.0001 (0.7130, 0.0073, 0.9894) | < 0.0001 (< 0.0001, 1.0000, 0.9661) |
| 2684 | 0.3480 (0.2865, 0.1006, 0.8101) | < 0.0001 (1.0000, 1.0000, 0.9774) |
| 2685 | < 0.0001 (0.1320, 0.0073, 0.9961) | < 0.0001 (1.0000, 1.0000, 0.9797) |
| 2686 | < 0.0001 (0.0117, 0.0073, 0.9894) | < 0.0001 (1.0000, 1.0000, 0.9608) |
| 2687 | < 0.0001 (0.4360, 0.0099, 0.9999) | < 0.0001 (< 0.0001, 1.0000, 0.9299) |
| 2688 | < 0.0001 (< 0.0001, 0.0041, 0.9970) | < 0.0001 (1.0000, 1.0000, 0.9977) |
| 2689 | < 0.0001 (0.0588, 0.0050, 1.0000) | < 0.0001 (< 0.0001, 1.0000, 0.9996) |
| 2690 | 0.9511 (0.1977, 0.4652, 0.8443) | 0.3334 (1.0000, 0.9998, 0.8864) |
| 2691 | < 0.0001 (0.1977, 0.0095, 0.9999) | < 0.0001 (1.0000, 1.0000, 0.8999) |
| 2692 | < 0.0001 (0.2583, 0.0083, 0.9999) | < 0.0001 (< 0.0001, 1.0000, 0.7553) |
| 2693 | < 0.0001 (0.2963, 0.0126, 0.9986) | < 0.0001 (1.0000, 1.0000, 0.8947) |
| 2694 | 1.0000 (< 0.0001, 1.0000, 0.9351) | < 0.0001 (1.0000, 1.0000, 0.9904) |
| 2695 | 0.2227 (0.6667, 0.0231, 0.9356) | < 0.0001 (1.0000, 1.0000, 0.9751) |
| 2696 | < 0.0001 (1.0000, 0.0083, 0.9994) | < 0.0001 (1.0000, 1.0000, 0.8717) |
| 2697 | 1.0000 (< 0.0001, 1.0000, 0.9404) | < 0.0001 (1.0000, 1.0000, 0.9480) |
| 2698 | < 0.0001 (0.6950, 0.0065, 0.9993) | 0.1667 (< 0.0001, 0.9877, 0.9746) |
| 2699 | 1.0000 (0.4823, 1.0000, 0.9798) | < 0.0001 (1.0000, 1.0000, 0.9694) |
| 2700 | < 0.0001 (0.6667, 0.0424, 0.9986) | 0.0948 (1.0000, 0.9989, 0.9777) |
| 2701 | < 0.0001 (< 0.0001, 0.0099, 0.9853) | 0.2321 (1.0000, 0.9681, 0.8715) |
| 2702 | 1.0000 (1.0000, 1.0000, 0.8849) | < 0.0001 (1.0000, 1.0000, 0.9909) |
| 2703 | 0.3105 (0.4736, 0.0737, 0.9997) | < 0.0001 (1.0000, 1.0000, 0.8745) |
| 2704 | < 0.0001 (0.4992, 0.0093, 0.9967) | 0.1667 (1.0000, 0.9974, 0.8947) |
| 2705 | 0.5728 (0.4445, 0.1927, 0.8514) | < 0.0001 (< 0.0001, 1.0000, 0.9814) |
| 2706 | 1.0000 (0.0880, 1.0000, 0.9351) | < 0.0001 (1.0000, 1.0000, 0.9992) |
| 2707 | < 0.0001 (< 0.0001, 0.0117, 1.0000) | < 0.0001 (1.0000, 1.0000, 0.9500) |
| 2708 | < 0.0001 (0.1317, 0.0087, 0.9949) | < 0.0001 (1.0000, 1.0000, 0.9944) |
| 2709 | 1.0000 (0.8889, 1.0000, 0.9525) | < 0.0001 (1.0000, 1.0000, 0.9693) |
| 2710 | 1.0000 (0.1621, 1.0000, 0.9431) | < 0.0001 (1.0000, 1.0000, 0.9325) |
| 2711 | 1.0000 (0.0116, 1.0000, 0.9525) | < 0.0001 (1.0000, 1.0000, 0.8864) |
| 2712 | < 0.0001 (0.6667, 0.0126, 1.0000) | < 0.0001 (1.0000, 1.0000, 0.9774) |
| 2713 | 1.0000 (0.7432, 1.0000, 0.8871) | < 0.0001 (1.0000, 1.0000, 0.9020) |
| 2714 | < 0.0001 (0.0936, 0.0093, 0.9998) | < 0.0001 (< 0.0001, 1.0000, 0.9997) |
| 2715 | 1.0000 (0.2963, 1.0000, 0.8837) | < 0.0001 (1.0000, 1.0000, 0.9953) |
| 2716 | 1.0000 (0.4444, 1.0000, 0.9870) | < 0.0001 (< 0.0001, 1.0000, 0.9876) |
| 2717 | < 0.0001 (< 0.0001, 0.0093, 1.0000) | < 0.0001 (1.0000, 1.0000, 0.9904) |
| 2718 | < 0.0001 (0.9956, 0.0060, 0.9999) | < 0.0001 (1.0000, 1.0000, 0.9860) |
| 2719 | < 0.0001 (0.2583, 0.0083, 0.9993) | < 0.0001 (1.0000, 1.0000, 0.9770) |
| 2720 | 0.4406 (0.3511, 0.0901, 0.9593) | < 0.0001 (0.9947, 1.0000, 0.9943) |
| 2721 | 1.0000 (0.1317, 1.0000, 0.9404) | < 0.0001 (1.0000, 1.0000, 0.9500) |
| 2722 | 0.6362 (0.7190, 0.2447, 0.8471) | 0.1667 (1.0000, 0.9877, 0.9931) |
| 2723 | 1.0000 (0.2964, 1.0000, 0.9870) | 0.0656 (1.0000, 0.9999, 0.9752) |
| 2724 | < 0.0001 (< 0.0001, 0.0073, 0.9987) | 0.4082 (0.6811, 0.9160, 0.9206) |
| 2725 | < 0.0001 (1.0000, 0.0424, 0.9993) | < 0.0001 (1.0000, 1.0000, 0.9911) |
| 2726 | < 0.0001 (0.1760, 0.0126, 0.9898) | 0.4975 (1.0000, 0.6826, 0.7423) |
| 2727 | < 0.0001 (0.5084, 0.0343, 0.9921) | 0.9240 (1.0000, 0.7635, 0.9950) |
| 2728 | 0.3507 (0.2583, 0.1026, 0.9693) | < 0.0001 (< 0.0001, 1.0000, 0.5086) |
| 2729 | < 0.0001 (0.9874, 0.0096, 1.0000) | < 0.0001 (0.0179, 1.0000, 0.4059) |
| 2730 | 0.4166 (1.0000, 0.1530, 0.8033) | < 0.0001 (1.0000, 1.0000, 0.9020) |
| 2731 | < 0.0001 (< 0.0001, 0.0056, 0.9884) | < 0.0001 (1.0000, 1.0000, 0.9578) |
| 2732 | < 0.0001 (0.3624, 0.0056, 0.9999) | < 0.0001 (1.0000, 1.0000, 0.9891) |
| 2733 | 0.6358 (0.1317, 0.2444, 0.7816) | 1.0131 (< 0.0001, 0.4331, 0.9529) |
| 2734 | 1.3490 (0.1842, 0.3435, 0.9620) | < 0.0001 (1.0000, 1.0000, 0.9823) |
| 2735 | 1.0450 (0.3624, 0.4698, 0.9952) | < 0.0001 (1.0000, 1.0000, 0.3296) |
| 2736 | < 0.0001 (0.6988, 0.0044, 0.9891) | < 0.0001 (1.0000, 1.0000, 0.7922) |
| 2737 | < 0.0001 (0.0123, 0.0053, 0.9905) | 0.1532 (< 0.0001, 0.9902, 0.8952) |
| 2738 | < 0.0001 (0.2963, 0.0093, 0.9993) | < 0.0001 (< 0.0001, 1.0000, 0.9944) |
| 2739 | < 0.0001 (0.6602, 0.0126, 0.9901) | < 0.0001 (0.3051, 1.0000, 0.7886) |
| 2740 | < 0.0001 (< 0.0001, 0.0131, 0.9963) | < 0.0001 (0.9877, 1.0000, 0.9995) |
| 2741 | < 0.0001 (0.3624, 0.0095, 0.9998) | 0.1154 (1.0000, 0.9969, 0.9436) |
| 2742 | < 0.0001 (0.6667, 0.0053, 0.9985) | < 0.0001 (< 0.0001, 1.0000, 0.9480) |
| 2743 | < 0.0001 (0.6667, 0.0107, 1.0000) | < 0.0001 (< 0.0001, 1.0000, 0.9764) |
| 2744 | < 0.0001 (< 0.0001, 0.0050, 1.0000) | 0.2319 (< 0.0001, 0.9682, 0.9726) |
| 2745 | < 0.0001 (< 0.0001, 0.0099, 0.9953) | < 0.0001 (< 0.0001, 1.0000, 0.9219) |
| 2746 | < 0.0001 (0.6667, 0.0073, 0.9894) | < 0.0001 (1.0000, 1.0000, 0.8727) |
| 2747 | 1.0000 (0.6667, 1.0000, 0.9870) | < 0.0001 (< 0.0001, 1.0000, 0.8122) |
| 2748 | < 0.0001 (0.2963, 0.0142, 0.9985) | < 0.0001 (1.0000, 1.0000, 0.9897) |
| 2749 | 1.0000 (0.6612, 1.0000, 0.9798) | < 0.0001 (< 0.0001, 1.0000, 0.9983) |
| 2750 | < 0.0001 (0.6667, 0.0083, 0.9948) | < 0.0001 (< 0.0001, 1.0000, 0.9916) |
| 2751 | < 0.0001 (0.7130, 0.0037, 0.9993) | < 0.0001 (1.0000, 1.0000, 0.9747) |
| 2752 | < 0.0001 (0.1842, 0.0311, 0.9991) | < 0.0001 (1.0000, 1.0000, 0.9966) |
| 2753 | < 0.0001 (0.4444, 0.0061, 0.9996) | < 0.0001 (0.7845, 1.0000, 0.9480) |
| 2754 | 0.8099 (0.7130, 0.3787, 0.9067) | < 0.0001 (1.0000, 1.0000, 0.9780) |
| 2755 | < 0.0001 (1.0000, 0.0056, 0.9995) | < 0.0001 (< 0.0001, 1.0000, 0.9916) |
| 2756 | < 0.0001 (0.0821, 0.0053, 0.9971) | < 0.0001 (1.0000, 1.0000, 0.9954) |
| 2757 | 0.3477 (0.1788, 0.1004, 0.9800) | < 0.0001 (1.0000, 1.0000, 0.9962) |
| 2758 | 0.6960 (0.1841, 0.2929, 0.9814) | < 0.0001 (1.0000, 1.0000, 0.9706) |
| 2759 | < 0.0001 (0.7066, 0.0099, 0.9932) | < 0.0001 (1.0000, 1.0000, 0.9542) |
| 2760 | 0.4047 (0.6944, 0.1437, 0.8214) | 2.4889 (1.0000, 0.1472, 0.9647) |
| 2761 | 0.3480 (0.1837, 0.1006, 0.7936) | < 0.0001 (1.0000, 1.0000, 0.9299) |
| 2762 | < 0.0001 (0.9176, 0.0053, 0.9992) | < 0.0001 (1.0000, 1.0000, 0.9264) |
| 2763 | 1.0000 (< 0.0001, 1.0000, 0.9870) | < 0.0001 (1.0000, 1.0000, 0.9958) |
| 2764 | < 0.0001 (0.2963, 0.0050, 0.9963) | < 0.0001 (1.0000, 1.0000, 0.9895) |
| 2765 | < 0.0001 (0.0588, 0.0037, 0.9964) | < 0.0001 (1.0000, 1.0000, 0.7945) |
| 2766 | < 0.0001 (0.7562, 0.0343, 0.9817) | < 0.0001 (1.0000, 1.0000, 0.9852) |
| 2767 | 1.0000 (0.9996, 1.0000, 0.9525) | < 0.0001 (1.0000, 1.0000, 0.9983) |
| 2768 | < 0.0001 (1.0000, 0.0126, 0.9999) | < 0.0001 (1.0000, 1.0000, 0.9325) |
| 2769 | < 0.0001 (0.1843, 0.0425, 1.0000) | < 0.0001 (1.0000, 1.0000, 0.9999) |
| 2770 | 1.0000 (0.2964, 1.0000, 0.9216) | < 0.0001 (1.0000, 1.0000, 0.9767) |
| 2771 | 1.0000 (0.0936, 1.0000, 0.9342) | < 0.0001 (1.0000, 1.0000, 0.8478) |
| 2772 | 0.2226 (< 0.0001, 0.0231, 0.9358) | < 0.0001 (1.0000, 1.0000, 0.7404) |
| 2773 | 0.3358 (0.3526, 0.0916, 0.6903) | < 0.0001 (< 0.0001, 1.0000, 0.8864) |
| 2774 | < 0.0001 (0.0279, 0.0060, 0.9966) | < 0.0001 (1.0000, 1.0000, 0.9859) |
| 2775 | 0.2862 (0.0394, 0.0576, 0.8978) | < 0.0001 (< 0.0001, 1.0000, 0.9999) |
| 2776 | < 0.0001 (0.6784, 0.0050, 0.9988) | < 0.0001 (0.9997, 1.0000, 0.9966) |
| 2777 | 1.0000 (0.6784, 1.0000, 0.9798) | 0.5133 (1.0000, 0.8849, 0.9823) |
| 2778 | < 0.0001 (0.4231, 0.0060, 0.9969) | < 0.0001 (1.0000, 1.0000, 0.9990) |
| 2779 | < 0.0001 (< 0.0001, 0.0060, 0.9991) | < 0.0001 (0.9547, 1.0000, 0.8847) |
| 2780 | 0.3611 (0.1976, 0.1103, 0.9999) | < 0.0001 (0.6667, 1.0000, 0.9904) |
| 2781 | 1.0000 (0.4883, 1.0000, 0.9174) | < 0.0001 (0.0002, 1.0000, 0.9524) |
| 2782 | 0.4162 (0.9991, 0.1527, 1.0000) | 0.5000 (< 0.0001, 0.8889, 0.9480) |
| 2783 | 0.3358 (< 0.0001, 0.0916, 0.9738) | < 0.0001 (1.0000, 1.0000, 0.8020) |
| 2784 | < 0.0001 (< 0.0001, 0.0073, 0.9885) | < 0.0001 (1.0000, 1.0000, 0.7528) |
| 2785 | < 0.0001 (0.2492, 0.0065, 1.0000) | < 0.0001 (1.0000, 1.0000, 0.9890) |
| 2786 | 1.7441 (0.0666, 0.1278, 0.9950) | < 0.0001 (< 0.0001, 1.0000, 0.9971) |
| 2787 | < 0.0001 (1.0000, 0.0053, 0.9753) | < 0.0001 (1.0000, 1.0000, 0.9948) |
| 2788 | < 0.0001 (0.1977, 0.0397, 0.9982) | < 0.0001 (1.0000, 1.0000, 0.9913) |
| 2789 | < 0.0001 (0.1317, 0.0060, 0.9947) | < 0.0001 (1.0000, 1.0000, 0.9985) |
| 2790 | < 0.0001 (0.1127, 0.0037, 0.9880) | < 0.0001 (1.0000, 1.0000, 0.9774) |
| 2791 | 1.0000 (0.3625, 1.0000, 0.9216) | < 0.0001 (1.0000, 1.0000, 0.9661) |
| 2792 | < 0.0001 (0.5083, 0.0050, 0.9963) | < 0.0001 (< 0.0001, 1.0000, 0.9962) |
| 2793 | 1.0000 (0.5084, 1.0000, 0.9411) | < 0.0001 (< 0.0001, 1.0000, 0.9661) |
| 2794 | < 0.0001 (0.1975, 0.0061, 1.0000) | < 0.0001 (< 0.0001, 1.0000, 0.8115) |
| 2795 | < 0.0001 (0.1977, 0.0083, 1.0000) | < 0.0001 (1.0000, 1.0000, 0.9448) |
| 2796 | < 0.0001 (0.6667, 0.0060, 0.9978) | < 0.0001 (1.0000, 1.0000, 0.9997) |
| 2797 | < 0.0001 (0.0877, 0.0060, 0.9995) | < 0.0001 (1.0000, 1.0000, 0.9947) |
| 2798 | < 0.0001 (0.6667, 0.0093, 0.9977) | < 0.0001 (< 0.0001, 1.0000, 0.9958) |
| 2799 | < 0.0001 (0.4446, 0.0422, 1.0000) | < 0.0001 (< 0.0001, 1.0000, 0.9830) |
| 2800 | < 0.0001 (0.3413, 0.0126, 0.9999) | < 0.0001 (1.0000, 1.0000, 0.8967) |
| 2801 | 0.6135 (0.7130, 0.2261, 0.8056) | < 0.0001 (< 0.0001, 1.0000, 0.9608) |
| 2802 | < 0.0001 (< 0.0001, 0.0131, 0.9998) | < 0.0001 (1.0000, 1.0000, 0.9980) |
| 2803 | < 0.0001 (< 0.0001, 0.0065, 0.9987) | < 0.0001 (1.0000, 1.0000, 0.8494) |
| 2804 | 0.3014 (0.6882, 0.0675, 0.9916) | < 0.0001 (1.0000, 1.0000, 0.9859) |
| 2805 | 0.5575 (< 0.0001, 0.1802, 0.9989) | < 0.0001 (1.0000, 1.0000, 0.9991) |
| 2806 | 0.3166 (0.6882, 0.0779, 0.9481) | < 0.0001 (< 0.0001, 1.0000, 0.9750) |
| 2807 | < 0.0001 (< 0.0001, 0.0060, 0.9998) | < 0.0001 (< 0.0001, 1.0000, 0.8952) |
| 2808 | 1.2686 (0.7130, 0.3238, 0.2254) | < 0.0001 (1.0000, 1.0000, 0.9746) |
| 2809 | 0.6144 (0.9406, 0.2269, 0.5663) | < 0.0001 (1.0000, 1.0000, 0.9859) |
| 2810 | < 0.0001 (0.5926, 0.0041, 0.9905) | < 0.0001 (1.0000, 1.0000, 0.9752) |
| 2811 | < 0.0001 (0.9849, 0.0060, 0.9978) | 0.1250 (1.0000, 0.9959, 0.9906) |
| 2812 | < 0.0001 (0.1760, 0.0073, 0.9985) | < 0.0001 (1.0000, 1.0000, 0.9012) |
| 2813 | < 0.0001 (0.2326, 0.0312, 0.9996) | < 0.0001 (1.0000, 1.0000, 0.9242) |
| 2814 | < 0.0001 (1.0000, 0.0126, 0.9972) | < 0.0001 (< 0.0001, 1.0000, 0.9998) |
| 2815 | 1.1468 (0.1702, 0.3945, 0.4279) | < 0.0001 (1.0000, 1.0000, 0.9719) |
| 2816 | 1.0000 (1.0000, 1.0000, 0.8339) | < 0.0001 (1.0000, 1.0000, 0.9996) |
| 2817 | 1.0000 (0.6944, 1.0000, 0.9404) | < 0.0001 (1.0000, 1.0000, 0.9524) |
| 2818 | < 0.0001 (0.1543, 0.0142, 1.0000) | < 0.0001 (< 0.0001, 1.0000, 0.9719) |
| 2819 | 0.2786 (< 0.0001, 0.0528, 0.9821) | < 0.0001 (< 0.0001, 1.0000, 0.9876) |
| 2820 | 0.7858 (0.7130, 0.3613, 0.9898) | < 0.0001 (1.0000, 1.0000, 0.9299) |
| 2821 | < 0.0001 (< 0.0001, 0.0050, 0.9960) | < 0.0001 (< 0.0001, 1.0000, 0.9746) |
| 2822 | 1.0000 (1.0000, 1.0000, 0.9798) | < 0.0001 (< 0.0001, 1.0000, 0.9945) |
| 2823 | 0.3686 (0.1975, 0.1160, 1.0000) | < 0.0001 (< 0.0001, 1.0000, 0.9859) |
| 2824 | 1.1071 (< 0.0001, 0.4313, 0.7888) | < 0.0001 (< 0.0001, 1.0000, 0.9943) |
| 2825 | < 0.0001 (< 0.0001, 0.0065, 0.9651) | < 0.0001 (1.0000, 1.0000, 0.9780) |
| 2826 | 0.6720 (0.9989, 0.2738, 0.8313) | < 0.0001 (1.0000, 1.0000, 0.9891) |
| 2827 | < 0.0001 (0.6603, 0.0053, 0.9753) | < 0.0001 (1.0000, 1.0000, 0.8717) |
| 2828 | < 0.0001 (0.4602, 0.0099, 0.9997) | < 0.0001 (0.6521, 1.0000, 0.9921) |
| 2829 | < 0.0001 (0.2392, 0.0093, 0.9993) | < 0.0001 (1.0000, 1.0000, 0.9498) |
| 2830 | < 0.0001 (0.1317, 0.0142, 0.9998) | < 0.0001 (1.0000, 1.0000, 0.9480) |
| 2831 | < 0.0001 (0.1975, 0.0044, 0.9995) | 0.5000 (1.0000, 0.6667, 0.7907) |
| 2832 | < 0.0001 (0.0936, 0.0050, 0.9999) | < 0.0001 (< 0.0001, 1.0000, 0.9480) |
| 2833 | 1.0000 (< 0.0001, 1.0000, 0.9798) | < 0.0001 (1.0000, 1.0000, 0.6348) |
| 2834 | < 0.0001 (0.3525, 0.0053, 0.9997) | < 0.0001 (0.8269, 1.0000, 0.9914) |
| 2835 | < 0.0001 (0.9996, 0.0142, 0.9985) | < 0.0001 (< 0.0001, 1.0000, 0.7907) |
| 2836 | < 0.0001 (< 0.0001, 0.0099, 0.9984) | < 0.0001 (0.8889, 1.0000, 0.9912) |
| 2837 | < 0.0001 (1.0000, 0.0056, 1.0000) | < 0.0001 (1.0000, 1.0000, 0.8999) |
| 2838 | < 0.0001 (0.2490, 0.0037, 0.9974) | < 0.0001 (< 0.0001, 1.0000, 0.9891) |
| 2839 | < 0.0001 (0.4446, 0.0142, 0.9915) | < 0.0001 (1.0000, 1.0000, 0.9965) |
| 2840 | 0.9745 (0.3356, 0.4821, 0.5508) | < 0.0001 (0.3158, 1.0000, 0.9571) |
| 2841 | 1.0000 (0.6667, 1.0000, 0.9525) | < 0.0001 (1.0000, 1.0000, 0.9860) |
| **2842** | **8.8079 (0.0034, < 0.0001, < 0.0001)** | **2.4993** (< **0.0001**, 1.0000, 0.0446) |
| 2843 | < 0.0001 (0.4359, 0.0093, 0.9977) | < 0.0001 (1.0000, 1.0000, 0.9661) |
| 2844 | < 0.0001 (< 0.0001, 0.0050, 0.9989) | < 0.0001 (< 0.0001, 1.0000, 0.9977) |
| 2845 | < 0.0001 (0.0357, 0.0056, 0.9877) | < 0.0001 (1.0000, 1.0000, 0.9928) |
| 2846 | < 0.0001 (< 0.0001, 0.0107, 0.9975) | < 0.0001 (1.0000, 1.0000, 0.9528) |
| 2847 | < 0.0001 (0.3526, 0.0061, 0.9995) | < 0.0001 (< 0.0001, 1.0000, 0.8952) |
| 2848 | < 0.0001 (0.3625, 0.0037, 0.9695) | 0.7021 (< 0.0001, 0.9017, 0.9719) |
| 2849 | < 0.0001 (0.1318, 0.0120, 1.0000) | < 0.0001 (< 0.0001, 1.0000, 0.9978) |
| 2850 | < 0.0001 (0.1317, 0.0425, 0.9973) | < 0.0001 (0.6810, 1.0000, 0.0453) |
| 2851 | < 0.0001 (0.1314, 0.0083, 0.9841) | 0.2500 (1.0000, 0.9630, 0.9746) |
| 2852 | < 0.0001 (0.2969, 0.0053, 0.9676) | 0.1540 (1.0000, 0.9900, 0.9746) |
| 2853 | < 0.0001 (0.2963, 0.0060, 0.9854) | < 0.0001 (1.0000, 1.0000, 0.8148) |
| 2854 | < 0.0001 (0.0005, 0.0083, 0.9996) | < 0.0001 (0.4906, 1.0000, 0.8340) |
| 2855 | < 0.0001 (< 0.0001, 0.0083, 1.0000) | < 0.0001 (1.0000, 1.0000, 0.8184) |
| 2856 | < 0.0001 (0.6949, 0.0041, 0.9989) | < 0.0001 (< 0.0001, 1.0000, 0.9685) |
| 2857 | < 0.0001 (0.6667, 0.0126, 0.9972) | < 0.0001 (1.0000, 1.0000, 0.9349) |
| 2858 | < 0.0001 (0.4345, 0.0126, 1.0000) | < 0.0001 (1.0000, 1.0000, 0.9941) |
| 2859 | 0.8098 (0.2117, 0.3786, 0.9067) | < 0.0001 (1.0000, 1.0000, 0.7907) |
| 2860 | < 0.0001 (0.3624, 0.0060, 0.9998) | < 0.0001 (< 0.0001, 1.0000, 0.9992) |
| 2861 | < 0.0001 (0.6667, 0.0093, 0.9967) | < 0.0001 (1.0000, 1.0000, 0.9913) |
| 2862 | 1.0000 (0.3121, 1.0000, 0.9798) | < 0.0001 (1.0000, 1.0000, 0.9661) |
| 2863 | < 0.0001 (0.4448, 0.0065, 0.9733) | < 0.0001 (1.0000, 1.0000, 0.8363) |
| 2864 | 0.6030 (< 0.0001, 0.2175, 0.8786) | < 0.0001 (< 0.0001, 1.0000, 0.9719) |
| 2865 | < 0.0001 (< 0.0001, 0.0060, 0.9999) | < 0.0001 (1.0000, 1.0000, 0.8952) |
| 2866 | 0.3013 (0.6667, 0.0674, 0.9954) | < 0.0001 (1.0000, 1.0000, 0.9839) |
| 2867 | 0.3066 (0.9764, 0.0710, 0.9861) | < 0.0001 (1.0000, 1.0000, 0.9288) |
| 2868 | 0.3480 (0.9981, 0.1006, 0.9882) | 2.0253 (1.0000, 0.1882, 0.9613) |
| 2869 | < 0.0001 (< 0.0001, 0.0050, 0.9996) | 0.4619 (< 0.0001, 0.9002, 0.9480) |
| 2870 | < 0.0001 (0.5926, 0.0050, 0.9874) | < 0.0001 (1.0000, 1.0000, 0.1143) |
| 2871 | 1.0000 (1.0000, 1.0000, 0.9870) | < 0.0001 (< 0.0001, 1.0000, 0.6253) |
| 2872 | < 0.0001 (< 0.0001, 0.0061, 0.9998) | < 0.0001 (< 0.0001, 1.0000, 0.9756) |
| 2873 | 1.0000 (0.6667, 1.0000, 0.9870) | < 0.0001 (1.0000, 1.0000, 0.8999) |
| 2874 | < 0.0001 (0.6667, 0.0050, 0.9980) | < 0.0001 (1.0000, 1.0000, 0.9891) |
| 2875 | < 0.0001 (0.2963, 0.0065, 0.9952) | < 0.0001 (1.0000, 1.0000, 0.9885) |
| 2876 | < 0.0001 (< 0.0001, 0.0050, 1.0000) | < 0.0001 (1.0000, 1.0000, 0.9521) |
| 2877 | < 0.0001 (1.0000, 0.0050, 1.0000) | 0.5000 (1.0000, 0.6667, 0.8947) |
| 2878 | < 0.0001 (0.0078, 0.0093, 0.9999) | 1.4347 (1.0000, 0.3580, 0.9647) |
| 2879 | < 0.0001 (< 0.0001, 0.0117, 0.9993) | < 0.0001 (1.0000, 1.0000, 0.6944) |
| 2880 | < 0.0001 (0.2963, 0.0056, 0.9884) | < 0.0001 (1.0000, 1.0000, 0.2061) |
| 2881 | < 0.0001 (0.1308, 0.0343, 0.9925) | < 0.0001 (1.0000, 1.0000, 0.8363) |
| 2882 | < 0.0001 (0.0032, 0.0343, 0.9995) | 1.3367 (< 0.0001, 0.9900, 0.9480) |
| 2883 | < 0.0001 (0.1087, 0.0313, 0.9929) | < 0.0001 (< 0.0001, 1.0000, 0.9891) |
| 2884 | < 0.0001 (0.7130, 0.0083, 0.9948) | 0.4865 (< 0.0001, 0.6852, 0.4381) |
| 2885 | < 0.0001 (0.1314, 0.0117, 1.0000) | < 0.0001 (1.0000, 1.0000, 0.9299) |
| 2886 | < 0.0001 (< 0.0001, 0.0060, 0.9995) | < 0.0001 (1.0000, 1.0000, 0.2814) |
| 2887 | < 0.0001 (0.4602, 0.0073, 0.9964) | < 0.0001 (1.0000, 1.0000, 0.8952) |
| 2888 | < 0.0001 (0.9981, 0.0060, 0.9989) | < 0.0001 (1.0000, 1.0000, 0.9891) |
| 2889 | < 0.0001 (0.0572, 0.0083, 0.9948) | < 0.0001 (0.8999, 1.0000, 0.9893) |
| 2890 | < 0.0001 (0.8002, 0.0065, 0.9987) | < 0.0001 (1.0000, 1.0000, 0.9891) |
| 2891 | < 0.0001 (0.0880, 0.0050, 0.9960) | < 0.0001 (1.0000, 1.0000, 0.9885) |
| 2892 | 1.0000 (0.6668, 1.0000, 0.9379) | 0.5353 (< 0.0001, 0.8784, 0.9964) |
| 2893 | 0.3188 (0.1977, 0.0794, 0.9922) | < 0.0001 (< 0.0001, 1.0000, 0.9997) |
| 2894 | < 0.0001 (0.6667, 0.0093, 1.0000) | < 0.0001 (1.0000, 1.0000, 0.8157) |
| 2895 | < 0.0001 (< 0.0001, 0.0073, 0.9885) | < 0.0001 (1.0000, 1.0000, 0.9988) |
| 2896 | < 0.0001 (0.1317, 0.0093, 1.0000) | < 0.0001 (0.9630, 1.0000, 0.8947) |
| 2897 | 1.0000 (< 0.0001, 1.0000, 0.9552) | < 0.0001 (< 0.0001, 1.0000, 0.9925) |
| 2898 | < 0.0001 (< 0.0001, 0.0073, 0.9885) | < 0.0001 (< 0.0001, 1.0000, 0.9726) |
| 2899 | < 0.0001 (< 0.0001, 0.0060, 0.9978) | < 0.0001 (0.9039, 1.0000, 0.9993) |
| 2900 | 0.3480 (0.3300, 0.1006, 0.7936) | < 0.0001 (1.0000, 1.0000, 0.9950) |
| 2901 | < 0.0001 (0.6667, 0.0060, 0.9904) | < 0.0001 (< 0.0001, 1.0000, 0.9963) |
| 2902 | 0.4162 (0.1543, 0.1527, 0.9985) | < 0.0001 (< 0.0001, 1.0000, 0.9989) |
| 2903 | < 0.0001 (1.0000, 0.0083, 0.9948) | < 0.0001 (1.0000, 1.0000, 0.9781) |
| 2904 | < 0.0001 (0.1245, 0.0126, 0.9992) | < 0.0001 (1.0000, 1.0000, 0.9661) |
| 2905 | < 0.0001 (0.0242, 0.0053, 0.9997) | 0.2324 (< 0.0001, 0.9681, 0.9661) |
| 2906 | < 0.0001 (< 0.0001, 0.0073, 0.9964) | < 0.0001 (1.0000, 1.0000, 0.9952) |
| 2907 | 0.3013 (0.4444, 0.0674, 0.8848) | < 0.0001 (1.0000, 1.0000, 0.8825) |
| 2908 | < 0.0001 (< 0.0001, 0.0050, 0.9988) | < 0.0001 (< 0.0001, 1.0000, 0.8999) |
| 2909 | < 0.0001 (0.5533, 0.0053, 0.9994) | < 0.0001 (1.0000, 1.0000, 0.8952) |
| 2910 | 1.0000 (0.3099, 1.0000, 0.9798) | < 0.0001 (1.0000, 1.0000, 0.9747) |
| 2911 | < 0.0001 (0.9985, 0.0397, 0.9739) | < 0.0001 (1.0000, 1.0000, 0.9012) |
| 2912 | 1.0000 (< 0.0001, 1.0000, 0.9798) | < 0.0001 (1.0000, 1.0000, 0.9958) |
| 2913 | 0.3186 (0.2965, 0.0793, 0.8148) | < 0.0001 (< 0.0001, 1.0000, 0.8864) |
| 2914 | < 0.0001 (0.0585, 0.0117, 1.0000) | < 0.0001 (1.0000, 1.0000, 0.9717) |
| 2915 | < 0.0001 (0.7130, 0.0397, 0.9903) | < 0.0001 (0.9900, 1.0000, 0.9991) |
| 2916 | < 0.0001 (0.2491, 0.0061, 0.9998) | < 0.0001 (< 0.0001, 1.0000, 0.9694) |
| 2917 | 1.0000 (1.0000, 1.0000, 0.9342) | < 0.0001 (1.0000, 1.0000, 0.9746) |
| 2918 | < 0.0001 (0.0589, 0.0073, 0.9985) | < 0.0001 (1.0000, 1.0000, 0.9756) |
| 2919 | < 0.0001 (0.2964, 0.0060, 0.9998) | < 0.0001 (1.0000, 1.0000, 0.9019) |
| 2920 | 0.7020 (< 0.0001, 0.2976, 0.6086) | < 0.0001 (1.0000, 1.0000, 0.8864) |
| 2921 | < 0.0001 (1.0000, 0.0093, 0.9994) | < 0.0001 (0.8893, 1.0000, 0.8184) |
| 2922 | 1.0000 (0.1243, 1.0000, 0.9342) | < 0.0001 (1.0000, 1.0000, 0.8999) |
| 2923 | 0.2227 (0.1316, 0.0231, 0.9358) | < 0.0001 (1.0000, 1.0000, 0.9982) |
| 2924 | < 0.0001 (0.3625, 0.0087, 0.9999) | < 0.0001 (1.0000, 1.0000, 0.9840) |
| 2925 | < 0.0001 (< 0.0001, 0.0107, 1.0000) | < 0.0001 (< 0.0001, 1.0000, 0.9480) |
| 2926 | < 0.0001 (0.1387, 0.0313, 0.9862) | < 0.0001 (1.0000, 1.0000, 0.9830) |
| 2927 | 1.0000 (< 0.0001, 1.0000, 0.9404) | < 0.0001 (< 0.0001, 1.0000, 0.9661) |
| 2928 | < 0.0001 (0.0174, 0.0073, 0.9961) | < 0.0001 (< 0.0001, 1.0000, 0.9480) |
| 2929 | < 0.0001 (0.4446, 0.0117, 1.0000) | < 0.0001 (< 0.0001, 1.0000, 0.9943) |
| 2930 | 0.6614 (0.0262, 0.2211, 0.7115) | < 0.0001 (1.0000, 1.0000, 0.8864) |
| 2931 | < 0.0001 (0.0002, 0.0044, 0.9964) | < 0.0001 (1.0000, 1.0000, 0.9629) |
| 2932 | < 0.0001 (< 0.0001, 0.0060, 0.9989) | < 0.0001 (1.0000, 1.0000, 0.9892) |
| 2933 | 0.3480 (0.4890, 0.1006, 0.9926) | < 0.0001 (1.0000, 1.0000, 0.9913) |
| 2934 | 0.3065 (0.3121, 0.0710, 0.9849) | < 0.0001 (1.0000, 1.0000, 0.9983) |
| 2935 | < 0.0001 (0.0390, 0.0037, 0.9769) | < 0.0001 (< 0.0001, 1.0000, 0.8952) |
| 2936 | < 0.0001 (0.2965, 0.0053, 0.9870) | < 0.0001 (1.0000, 1.0000, 1.0000) |
| 2937 | < 0.0001 (< 0.0001, 0.0061, 0.9988) | < 0.0001 (1.0000, 1.0000, 0.9997) |
| 2938 | < 0.0001 (0.3624, 0.0061, 0.9996) | < 0.0001 (0.9824, 1.0000, 0.8055) |
| 2939 | 0.6500 (< 0.0001, 0.3214, 0.8943) | < 0.0001 (0.0188, 1.0000, 0.9993) |
| 2940 | < 0.0001 (0.0035, 0.0056, 0.9997) | < 0.0001 (< 0.0001, 1.0000, 0.9189) |
| 2941 | < 0.0001 (0.9176, 0.0061, 0.9987) | < 0.0001 (1.0000, 1.0000, 0.9738) |
| 2942 | 0.2226 (0.8743, 0.0231, 0.9358) | < 0.0001 (0.9547, 1.0000, 0.8856) |
| 2943 | 1.0000 (0.1319, 1.0000, 0.8736) | < 0.0001 (1.0000, 1.0000, 0.9287) |
| 2944 | < 0.0001 (0.1318, 0.0073, 0.9885) | < 0.0001 (< 0.0001, 1.0000, 0.8947) |
| 2945 | 1.0000 (0.4992, 1.0000, 0.9404) | < 0.0001 (1.0000, 1.0000, 0.9449) |
| 2946 | < 0.0001 (0.2963, 0.0343, 0.9968) | < 0.0001 (1.0000, 1.0000, 0.9726) |
| 2947 | < 0.0001 (< 0.0001, 0.0053, 0.9870) | < 0.0001 (1.0000, 1.0000, 0.9480) |
| 2948 | < 0.0001 (0.9066, 0.0343, 0.9964) | < 0.0001 (< 0.0001, 1.0000, 0.9724) |
| 2949 | 1.0000 (1.0000, 1.0000, 0.9798) | < 0.0001 (1.0000, 1.0000, 0.9694) |
| 2950 | **3.4583 (**0.8889**, 0.0002, 0.0135)** | < 0.0001 (1.0000, 1.0000, 0.9746) |
| 2951 | 0.5575 (0.0051, 0.1802, 0.9150) | < 0.0001 (1.0000, 1.0000, 0.9904) |
| 2952 | < 0.0001 (0.2020, 0.0061, 0.9987) | < 0.0001 (1.0000, 1.0000, 0.9500) |
| 2953 | < 0.0001 (0.8739, 0.0044, 0.9964) | < 0.0001 (1.0000, 1.0000, 0.9498) |
| 2954 | < 0.0001 (< 0.0001, 0.0073, 1.0000) | < 0.0001 (< 0.0001, 1.0000, 0.9693) |
| 2955 | < 0.0001 (0.5084, 0.0061, 0.9999) | < 0.0001 (1.0000, 1.0000, 0.9876) |
| 2956 | < 0.0001 (0.7130, 0.0083, 0.9772) | < 0.0001 (1.0000, 1.0000, 0.9188) |
| 2957 | 1.0000 (0.4448, 1.0000, 0.9351) | < 0.0001 (1.0000, 1.0000, 0.9943) |
| 2958 | < 0.0001 (< 0.0001, 0.0065, 0.9933) | < 0.0001 (1.0000, 1.0000, 0.9718) |
| 2959 | 0.3611 (0.9176, 0.1103, 0.8307) | < 0.0001 (< 0.0001, 1.0000, 0.9743) |
| 2960 | 0.9511 (0.6667, 0.4651, 0.6922) | < 0.0001 (< 0.0001, 1.0000, 0.7907) |
| 2961 | 1.0000 (0.9747, 1.0000, 0.8736) | < 0.0001 (0.4685, 1.0000, 0.9869) |
| 2962 | 1.0000 (0.1544, 1.0000, 0.9798) | < 0.0001 (1.0000, 1.0000, 0.9823) |
| 2963 | < 0.0001 (0.2244, 0.0073, 0.9995) | < 0.0001 (1.0000, 1.0000, 0.9938) |
| 2964 | 1.1163 (< 0.0001, 0.4046, 0.6040) | < 0.0001 (1.0000, 1.0000, 0.9904) |
| 2965 | 1.0000 (< 0.0001, 1.0000, 0.9525) | < 0.0001 (1.0000, 1.0000, 0.9937) |
| 2966 | 0.3244 (< 0.0001, 0.0834, 0.4299) | < 0.0001 (< 0.0001, 1.0000, 0.9808) |
| 2967 | 1.0000 (< 0.0001, 1.0000, 0.9404) | < 0.0001 (1.0000, 1.0000, 0.9974) |
| 2968 | < 0.0001 (1.0000, 0.0073, 0.9995) | < 0.0001 (1.0000, 1.0000, 0.9708) |
| 2969 | < 0.0001 (< 0.0001, 0.0085, 0.9998) | < 0.0001 (1.0000, 1.0000, 0.7730) |
| 2970 | < 0.0001 (0.4360, 0.0074, 0.9995) | < 0.0001 (1.0000, 1.0000, 0.9569) |
| 2971 | < 0.0001 (0.0937, 0.0061, 0.9948) | 0.1340 (1.0000, 0.9948, 0.8947) |
| 2972 | < 0.0001 (0.0878, 0.0099, 0.9999) | < 0.0001 (1.0000, 1.0000, 0.9527) |
| 2973 | 0.6735 (0.4361, 0.3362, 0.9996) | < 0.0001 (< 0.0001, 1.0000, 0.9743) |
| 2974 | 0.3635 (0.1318, 0.1122, 0.9277) | < 0.0001 (1.0000, 1.0000, 0.9985) |
| 2975 | < 0.0001 (0.9967, 0.0056, 0.9999) | 0.4975 (1.0000, 0.6826, 0.9935) |
| 2976 | 1.0000 (< 0.0001, 1.0000, 0.9351) | < 0.0001 (< 0.0001, 1.0000, 0.9998) |
| 2977 | < 0.0001 (0.2962, 0.0087, 0.9988) | < 0.0001 (1.0000, 1.0000, 0.9894) |
| 2978 | < 0.0001 (0.1760, 0.0099, 0.9853) | < 0.0001 (1.0000, 1.0000, 0.9429) |
| 2979 | < 0.0001 (1.0000, 0.0050, 0.9999) | < 0.0001 (1.0000, 1.0000, 0.5085) |
| 2980 | < 0.0001 (< 0.0001, 0.0087, 0.9982) | < 0.0001 (< 0.0001, 1.0000, 0.9890) |
| 2981 | < 0.0001 (< 0.0001, 0.0060, 0.9999) | < 0.0001 (1.0000, 1.0000, 0.9956) |
| 2982 | < 0.0001 (0.4360, 0.0142, 0.9996) | < 0.0001 (< 0.0001, 1.0000, 0.9627) |
| 2983 | < 0.0001 (0.4823, 0.0053, 0.9997) | < 0.0001 (< 0.0001, 1.0000, 0.7286) |
| 2984 | 1.0000 (0.9999, 1.0000, 0.9870) | < 0.0001 (0.7005, 1.0000, 0.9449) |
| 2985 | 1.0000 (< 0.0001, 1.0000, 0.9870) | < 0.0001 (< 0.0001, 1.0000, 0.9967) |
| 2986 | < 0.0001 (< 0.0001, 0.0060, 0.9996) | < 0.0001 (0.9974, 1.0000, 0.9866) |
| 2987 | 0.3166 (0.6784, 0.0779, 0.9776) | < 0.0001 (1.0000, 1.0000, 0.9429) |
| 2988 | 1.0000 (0.3625, 1.0000, 0.9342) | < 0.0001 (1.0000, 1.0000, 0.9480) |
| 2989 | < 0.0001 (0.4609, 0.0073, 0.9404) | < 0.0001 (< 0.0001, 1.0000, 0.9752) |
| 2990 | < 0.0001 (0.3623, 0.0060, 0.9896) | 1.4989 (1.0000, 0.3135, 0.9921) |
| 2991 | < 0.0001 (< 0.0001, 0.0060, 0.9966) | 0.1667 (1.0000, 0.9877, 0.9977) |
| 2992 | < 0.0001 (< 0.0001, 0.0093, 1.0000) | < 0.0001 (1.0000, 1.0000, 0.8553) |
| 2993 | < 0.0001 (< 0.0001, 0.0060, 0.9854) | < 0.0001 (< 0.0001, 1.0000, 0.9436) |
| 2994 | < 0.0001 (0.0394, 0.0073, 0.9964) | < 0.0001 (1.0000, 1.0000, 0.2016) |
| 2995 | < 0.0001 (0.2963, 0.0117, 1.0000) | < 0.0001 (1.0000, 1.0000, 0.8797) |
| 2996 | < 0.0001 (0.9335, 0.0060, 0.9947) | < 0.0001 (1.0000, 1.0000, 0.9450) |
| 2997 | < 0.0001 (0.6833, 0.0060, 0.9904) | < 0.0001 (< 0.0001, 1.0000, 0.7907) |
| 2998 | < 0.0001 (0.6882, 0.0061, 0.9960) | < 0.0001 (0.9620, 1.0000, 0.8947) |
| 2999 | 1.0000 (0.6950, 1.0000, 0.9431) | < 0.0001 (0.6638, 1.0000, 0.7404) |
| 3000 | 1.0000 (0.4444, 1.0000, 0.9216) | < 0.0001 (< 0.0001, 1.0000, 0.8947) |
| 3001 | < 0.0001 (0.4602, 0.0037, 0.9770) | < 0.0001 (< 0.0001, 1.0000, 0.9866) |
| 3002 | 1.0000 (0.6603, 1.0000, 0.9342) | < 0.0001 (< 0.0001, 1.0000, 0.9019) |
| 3003 | < 0.0001 (0.3625, 0.0061, 1.0000) | < 0.0001 (< 0.0001, 1.0000, 0.9859) |
| 3004 | < 0.0001 (1.0000, 0.0060, 0.9977) | 0.9186 (< 0.0001, 0.7652, 0.8292) |
| 3005 | 1.0000 (< 0.0001, 1.0000, 0.9798) | < 0.0001 (< 0.0001, 1.0000, 0.8952) |
| 3006 | < 0.0001 (< 0.0001, 0.0117, 0.9976) | < 0.0001 (1.0000, 1.0000, 0.9894) |
| 3007 | 1.0000 (0.1975, 1.0000, 0.9870) | < 0.0001 (< 0.0001, 1.0000, 0.9911) |
| 3008 | 1.0000 (0.7130, 1.0000, 0.9798) | < 0.0001 (1.0000, 1.0000, 0.3221) |
| 3009 | < 0.0001 (0.3485, 0.0126, 0.9999) | < 0.0001 (1.0000, 1.0000, 0.9895) |
| 3010 | < 0.0001 (0.5084, 0.0060, 0.9854) | < 0.0001 (1.0000, 1.0000, 0.8947) |
| 3011 | < 0.0001 (0.1166, 0.0061, 0.9978) | < 0.0001 (< 0.0001, 1.0000, 0.9929) |
| 3012 | < 0.0001 (0.0022, 0.0085, 0.9996) | < 0.0001 (1.0000, 1.0000, 0.9860) |
| 3013 | < 0.0001 (0.4608, 0.0117, 0.9989) | < 0.0001 (1.0000, 1.0000, 0.9569) |
| 3014 | < 0.0001 (0.3624, 0.0424, 0.9999) | < 0.0001 (1.0000, 1.0000, 0.9911) |
| 3015 | < 0.0001 (0.3624, 0.0074, 0.9404) | < 0.0001 (< 0.0001, 1.0000, 0.9992) |
| 3016 | < 0.0001 (0.4450, 0.0117, 1.0000) | < 0.0001 (< 0.0001, 1.0000, 0.9751) |
| 3017 | < 0.0001 (< 0.0001, 0.0073, 1.0000) | < 0.0001 (< 0.0001, 1.0000, 0.9756) |
| 3018 | 1.0000 (1.0000, 1.0000, 0.8455) | < 0.0001 (1.0000, 1.0000, 0.9890) |
| 3019 | < 0.0001 (0.4830, 0.0425, 0.9998) | < 0.0001 (< 0.0001, 1.0000, 0.9576) |
| 3020 | < 0.0001 (0.3625, 0.0056, 0.9954) | < 0.0001 (1.0000, 1.0000, 0.9718) |
| 3021 | < 0.0001 (0.0584, 0.0117, 0.9989) | < 0.0001 (< 0.0001, 1.0000, 0.9823) |
| 3022 | < 0.0001 (0.6667, 0.0126, 0.9898) | < 0.0001 (< 0.0001, 1.0000, 0.9921) |
| 3023 | < 0.0001 (0.4445, 0.0053, 0.9984) | < 0.0001 (1.0000, 1.0000, 0.9916) |
| 3024 | 1.0000 (0.3417, 1.0000, 0.9351) | < 0.0001 (1.0000, 1.0000, 0.9838) |
| 3025 | < 0.0001 (0.2962, 0.0065, 0.9733) | < 0.0001 (< 0.0001, 1.0000, 0.9725) |
| 3026 | < 0.0001 (0.3623, 0.0099, 0.9953) | < 0.0001 (1.0000, 1.0000, 0.9965) |
| 3027 | 1.0000 (0.5083, 1.0000, 0.9798) | 0.3334 (0.8887, 0.9998, 0.9521) |
| 3028 | < 0.0001 (0.7130, 0.0126, 0.9955) | < 0.0001 (< 0.0001, 1.0000, 0.9299) |
| 3029 | 0.3173 (0.0104, 0.0784, 0.9592) | < 0.0001 (0.9676, 1.0000, 0.9747) |
| 3030 | 0.2921 (< 0.0001, 0.0613, 0.8866) | 0.3333 (< 0.0001, 0.9547, 0.9299) |
| 3031 | < 0.0001 (0.6134, 0.0060, 0.9969) | 0.5000 (< 0.0001, 0.6667, 0.7552) |
| 3032 | < 0.0001 (0.9066, 0.0053, 0.9753) | < 0.0001 (1.0000, 1.0000, 0.9952) |
| 3033 | 1.0000 (0.2971, 1.0000, 0.9536) | < 0.0001 (1.0000, 1.0000, 0.9999) |
| 3034 | < 0.0001 (0.9212, 0.0060, 0.9854) | < 0.0001 (< 0.0001, 1.0000, 0.7365) |
| 3035 | < 0.0001 (0.6603, 0.0060, 0.9947) | < 0.0001 (1.0000, 1.0000, 0.3947) |
| 3036 | < 0.0001 (0.9165, 0.0056, 0.9822) | < 0.0001 (1.0000, 1.0000, 0.7286) |
| 3037 | < 0.0001 (0.0088, 0.0083, 0.9997) | < 0.0001 (0.4288, 1.0000, 0.8864) |
| 3038 | < 0.0001 (0.4883, 0.0073, 0.9964) | < 0.0001 (< 0.0001, 1.0000, 0.9746) |
| 3039 | < 0.0001 (0.3573, 0.0056, 0.9984) | < 0.0001 (1.0000, 1.0000, 0.9774) |
| 3040 | < 0.0001 (0.0261, 0.0397, 0.9739) | < 0.0001 (1.0000, 1.0000, 0.9973) |
| 3041 | < 0.0001 (0.6950, 0.0056, 0.9984) | < 0.0001 (1.0000, 1.0000, 0.9661) |
| 3042 | < 0.0001 (0.4883, 0.0126, 0.9898) | < 0.0001 (1.0000, 1.0000, 0.9237) |
| 3043 | 0.6035 (0.9002, 0.2908, 0.9853) | < 0.0001 (1.0000, 1.0000, 0.9975) |
| 3044 | < 0.0001 (0.3122, 0.0060, 0.9999) | < 0.0001 (< 0.0001, 1.0000, 0.9889) |
| 3045 | < 0.0001 (0.8901, 0.0424, 0.9993) | < 0.0001 (< 0.0001, 1.0000, 0.9997) |
| 3046 | < 0.0001 (0.9971, 0.0056, 0.9974) | < 0.0001 (1.0000, 1.0000, 0.9746) |
| 3047 | 0.4167 (0.0935, 0.1530, 0.9969) | < 0.0001 (< 0.0001, 1.0000, 0.8947) |
| 3048 | < 0.0001 (0.2966, 0.0060, 0.9957) | < 0.0001 (1.0000, 1.0000, 0.7286) |
| 3049 | < 0.0001 (0.6602, 0.0424, 0.9944) | < 0.0001 (1.0000, 1.0000, 0.8714) |
| 3050 | < 0.0001 (0.1313, 0.0085, 0.9915) | < 0.0001 (< 0.0001, 1.0000, 0.9751) |
| 3051 | 0.3359 (0.7130, 0.0917, 0.6902) | < 0.0001 (1.0000, 1.0000, 0.9895) |
| 3052 | < 0.0001 (< 0.0001, 0.0083, 0.9773) | < 0.0001 (1.0000, 1.0000, 0.9706) |
| 3053 | < 0.0001 (< 0.0001, 0.0056, 0.9944) | < 0.0001 (0.3512, 1.0000, 0.8864) |
| 3054 | 0.3013 (1.0000, 0.0674, 0.9916) | < 0.0001 (0.7508, 1.0000, 0.8715) |
| 3055 | 0.3638 (0.3281, 0.1124, 0.9981) | < 0.0001 (1.0000, 1.0000, 0.9914) |
| 3056 | 0.3614 (< 0.0001, 0.1105, 1.0000) | < 0.0001 (1.0000, 1.0000, 0.8478) |
| 3057 | < 0.0001 (0.5099, 0.0056, 0.9985) | < 0.0001 (< 0.0001, 1.0000, 0.9921) |
| 3058 | < 0.0001 (1.0000, 0.0056, 0.9696) | < 0.0001 (< 0.0001, 1.0000, 0.9693) |
| 3059 | 0.3166 (0.3694, 0.0779, 0.7199) | < 0.0001 (1.0000, 1.0000, 0.9913) |
| 3060 | < 0.0001 (0.4003, 0.0060, 0.9985) | < 0.0001 (1.0000, 1.0000, 0.9879) |
| 3061 | < 0.0001 (0.7459, 0.0142, 0.9993) | < 0.0001 (1.0000, 1.0000, 0.8122) |
| 3062 | < 0.0001 (0.4771, 0.0061, 0.9937) | < 0.0001 (< 0.0001, 1.0000, 0.8115) |
| 3063 | 1.0000 (1.0000, 1.0000, 0.8213) | < 0.0001 (1.0000, 1.0000, 0.9945) |
| 3064 | < 0.0001 (0.8642, 0.0065, 0.9894) | < 0.0001 (1.0000, 1.0000, 0.9774) |
| 3065 | < 0.0001 (0.1817, 0.0050, 0.9999) | < 0.0001 (1.0000, 1.0000, 0.9780) |
| 3066 | < 0.0001 (0.9998, 0.0061, 0.9991) | < 0.0001 (1.0000, 1.0000, 0.9885) |
| 3067 | < 0.0001 (< 0.0001, 0.0056, 0.9943) | < 0.0001 (1.0000, 1.0000, 0.9756) |
| 3068 | 1.0000 (0.1626, 1.0000, 0.9798) | < 0.0001 (1.0000, 1.0000, 0.9875) |
| 3069 | < 0.0001 (0.5826, 0.0065, 0.9952) | < 0.0001 (1.0000, 1.0000, 0.8714) |
| 3070 | < 0.0001 (0.7204, 0.0050, 0.9998) | < 0.0001 (< 0.0001, 1.0000, 0.9442) |
| 3071 | < 0.0001 (0.6714, 0.0085, 0.9997) | < 0.0001 (1.0000, 1.0000, 0.9859) |
| 3072 | < 0.0001 (0.4832, 0.0060, 0.9985) | < 0.0001 (1.0000, 1.0000, 0.9751) |
| 3073 | < 0.0001 (0.7679, 0.0050, 0.9960) | < 0.0001 (< 0.0001, 1.0000, 0.9661) |
| **3074** | **3.2247 (**0.7770**, 0.0006, 0.0492)** | < 0.0001 (0.7846, 1.0000, 0.9661) |
| 3075 | 0.3186 (0.1926, 0.0793, 0.9685) | < 0.0001 (< 0.0001, 1.0000, 0.9719) |
| 3076 | < 0.0001 (0.0006, 0.0065, 0.9733) | < 0.0001 (0.7595, 1.0000, 0.8967) |
| 3077 | < 0.0001 (0.0983, 0.0397, 0.9960) | < 0.0001 (1.0000, 1.0000, 0.8947) |
| 3078 | 1.0000 (0.9017, 1.0000, 0.9351) | < 0.0001 (1.0000, 1.0000, 0.8864) |
| 3079 | 1.0000 (0.8589, 1.0000, 0.8264) | 1.3794 (1.0000, 0.6252, 0.9299) |
| 3080 | 0.3358 (0.8359, 0.0916, 0.8797) | < 0.0001 (1.0000, 1.0000, 0.9841) |
| 3081 | 0.3187 (< 0.0001, 0.0793, 0.9922) | < 0.0001 (< 0.0001, 1.0000, 0.9429) |
| 3082 | < 0.0001 (0.6458, 0.0060, 0.9904) | < 0.0001 (0.9919, 1.0000, 0.9939) |
| 3083 | < 0.0001 (< 0.0001, 0.0083, 0.9948) | < 0.0001 (< 0.0001, 1.0000, 0.1822) |
| 3084 | 1.0000 (1.0000, 1.0000, 0.8565) | < 0.0001 (1.0000, 1.0000, 0.9814) |
| 3085 | < 0.0001 (0.0832, 0.0073, 0.9894) | 0.6662 (1.0000, 0.8269, 0.9859) |
| 3086 | < 0.0001 (0.0648, 0.0399, 1.0000) | < 0.0001 (1.0000, 1.0000, 0.9961) |
| 3087 | < 0.0001 (0.8871, 0.0126, 0.9955) | 0.5000 (< 0.0001, 0.8889, 0.9012) |
| 3088 | 0.6493 (0.9998, 0.2554, 0.8162) | < 0.0001 (1.0000, 1.0000, 0.9825) |
| 3089 | 0.3105 (< 0.0001, 0.0736, 0.9993) | < 0.0001 (0.9167, 1.0000, 0.8545) |
| 3090 | < 0.0001 (0.9066, 0.0073, 0.9885) | < 0.0001 (0.7447, 1.0000, 0.9012) |
| 3091 | < 0.0001 (1.0000, 0.0099, 0.9990) | 0.4783 (0.4610, 0.7100, 0.7317) |
| 3092 | < 0.0001 (< 0.0001, 0.0095, 0.9931) | < 0.0001 (1.0000, 1.0000, 0.9747) |
| 3093 | 0.3506 (0.6667, 0.1025, 0.9542) | < 0.0001 (1.0000, 1.0000, 0.8864) |
| 3094 | < 0.0001 (< 0.0001, 0.0126, 0.9998) | < 0.0001 (< 0.0001, 1.0000, 0.9997) |
| 3095 | 0.6376 (0.1761, 0.2459, 1.0000) | < 0.0001 (1.0000, 1.0000, 0.2061) |
| 3096 | < 0.0001 (< 0.0001, 0.0041, 0.9905) | < 0.0001 (< 0.0001, 1.0000, 0.9995) |
| 3097 | < 0.0001 (0.9990, 0.0061, 0.9867) | < 0.0001 (< 0.0001, 1.0000, 0.9020) |
| 3098 | < 0.0001 (0.5926, 0.0060, 0.9998) | < 0.0001 (1.0000, 1.0000, 0.9921) |
| 3099 | < 0.0001 (0.4402, 0.0060, 0.9808) | < 0.0001 (1.0000, 1.0000, 0.9480) |
| 3100 | < 0.0001 (0.4444, 0.0073, 0.9964) | < 0.0001 (1.0000, 1.0000, 0.9938) |
| 3101 | < 0.0001 (0.7522, 0.0126, 0.9898) | 0.4774 (1.0000, 0.7105, 0.9736) |
| 3102 | < 0.0001 (< 0.0001, 0.0050, 0.9998) | < 0.0001 (1.0000, 1.0000, 0.8952) |
| 3103 | < 0.0001 (0.6603, 0.0142, 0.9997) | < 0.0001 (1.0000, 1.0000, 0.7404) |
| 3104 | < 0.0001 (0.9997, 0.0085, 0.9967) | < 0.0001 (1.0000, 1.0000, 0.8967) |
| 3105 | 0.3686 (0.9630, 0.1160, 0.9739) | 0.9746 (1.0000, 0.4679, 0.4914) |
| 3106 | < 0.0001 (0.9653, 0.0060, 0.9998) | < 0.0001 (1.0000, 1.0000, 0.9719) |
| 3107 | < 0.0001 (< 0.0001, 0.0083, 0.9772) | < 0.0001 (1.0000, 1.0000, 0.9904) |
| 3108 | < 0.0001 (0.0853, 0.0083, 0.9948) | < 0.0001 (< 0.0001, 1.0000, 0.9941) |
| 3109 | < 0.0001 (0.5057, 0.0060, 0.9998) | < 0.0001 (1.0000, 1.0000, 0.2149) |
| 3110 | < 0.0001 (1.0000, 0.0083, 0.9948) | < 0.0001 (< 0.0001, 1.0000, 0.9893) |
| 3111 | 0.2226 (0.7407, 0.0231, 0.9357) | < 0.0001 (0.9997, 1.0000, 0.9661) |
| 3112 | < 0.0001 (1.0000, 0.0085, 0.9980) | < 0.0001 (1.0000, 1.0000, 0.9628) |
| 3113 | < 0.0001 (1.0000, 0.0093, 1.0000) | < 0.0001 (1.0000, 1.0000, 0.9995) |
| 3114 | 0.6384 (0.6373, 0.2466, 0.9012) | < 0.0001 (0.9547, 1.0000, 0.9325) |
| 3115 | 0.9569 (0.1778, 0.4694, 0.4734) | < 0.0001 (0.6667, 1.0000, 0.8864) |
| 3116 | < 0.0001 (1.0000, 0.0065, 0.9651) | < 0.0001 (< 0.0001, 1.0000, 0.9220) |
| 3117 | 1.0000 (0.5027, 1.0000, 0.9216) | < 0.0001 (< 0.0001, 1.0000, 0.9997) |
| 3118 | 0.2920 (0.4766, 0.0613, 0.9950) | < 0.0001 (1.0000, 1.0000, 0.9925) |
| 3119 | < 0.0001 (< 0.0001, 0.0050, 0.9988) | < 0.0001 (1.0000, 1.0000, 0.9299) |
| 3120 | < 0.0001 (0.9962, 0.0083, 0.9999) | < 0.0001 (0.0003, 1.0000, 0.9946) |
| 3121 | 1.0000 (0.4810, 1.0000, 0.9870) | < 0.0001 (< 0.0001, 1.0000, 0.9746) |
| 3122 | < 0.0001 (0.0387, 0.0065, 0.9933) | < 0.0001 (1.0000, 1.0000, 0.9984) |
| 3123 | < 0.0001 (0.4485, 0.0083, 1.0000) | < 0.0001 (1.0000, 1.0000, 0.9966) |
| 3124 | < 0.0001 (0.7221, 0.0060, 1.0000) | < 0.0001 (1.0000, 1.0000, 0.8020) |
| 3125 | < 0.0001 (0.2503, 0.0107, 1.0000) | < 0.0001 (1.0000, 1.0000, 0.9814) |
| 3126 | < 0.0001 (< 0.0001, 0.0044, 0.9891) | < 0.0001 (1.0000, 1.0000, 0.8562) |
| 3127 | < 0.0001 (0.6014, 0.0073, 0.9885) | < 0.0001 (< 0.0001, 1.0000, 0.8363) |
| 3128 | < 0.0001 (0.7082, 0.0065, 0.9856) | < 0.0001 (< 0.0001, 1.0000, 0.9962) |
| 3129 | 1.0000 (0.6489, 1.0000, 0.9230) | < 0.0001 (< 0.0001, 1.0000, 0.9921) |
| 3130 | < 0.0001 (0.6979, 0.0041, 0.9996) | < 0.0001 (1.0000, 1.0000, 0.9996) |
| 3131 | < 0.0001 (0.5840, 0.0056, 0.9960) | < 0.0001 (1.0000, 1.0000, 0.9943) |
| 3132 | < 0.0001 (0.4601, 0.0037, 0.9880) | < 0.0001 (1.0000, 1.0000, 0.9724) |
| 3133 | 1.0000 (0.5034, 1.0000, 0.9216) | < 0.0001 (< 0.0001, 1.0000, 0.9945) |
| 3134 | 1.0000 (0.9792, 1.0000, 0.9174) | < 0.0001 (< 0.0001, 1.0000, 0.9824) |
| 3135 | < 0.0001 (0.8301, 0.0083, 0.9988) | < 0.0001 (1.0000, 1.0000, 0.9908) |
| 3136 | < 0.0001 (0.4949, 0.0083, 1.0000) | < 0.0001 (< 0.0001, 1.0000, 1.0000) |
| 3137 | < 0.0001 (< 0.0001, 0.0050, 0.9963) | < 0.0001 (1.0000, 1.0000, 0.9661) |
| 3138 | < 0.0001 (0.0880, 0.0142, 0.9991) | < 0.0001 (1.0000, 1.0000, 0.9498) |
| 3139 | < 0.0001 (0.7674, 0.0061, 0.9987) | < 0.0001 (1.0000, 1.0000, 0.9019) |
| 3140 | < 0.0001 (1.0000, 0.0425, 0.9997) | 0.4951 (1.0000, 0.8904, 0.9521) |
| 3141 | < 0.0001 (0.2745, 0.0053, 0.9958) | < 0.0001 (< 0.0001, 1.0000, 0.9977) |
| 3142 | < 0.0001 (0.4070, 0.0050, 0.9941) | < 0.0001 (< 0.0001, 1.0000, 0.9965) |
| 3143 | < 0.0001 (0.6027, 0.0117, 0.9976) | < 0.0001 (1.0000, 1.0000, 0.9449) |
| 3144 | < 0.0001 (< 0.0001, 0.0050, 0.9874) | < 0.0001 (1.0000, 1.0000, 0.7116) |
| 3145 | < 0.0001 (< 0.0001, 0.0053, 0.9905) | < 0.0001 (1.0000, 1.0000, 0.9839) |
| 3146 | < 0.0001 (< 0.0001, 0.0425, 0.9973) | < 0.0001 (1.0000, 1.0000, 0.9891) |
| 3147 | 0.6380 (< 0.0001, 0.2462, 0.7443) | 0.2500 (1.0000, 0.9630, 0.9480) |
| 3148 | < 0.0001 (< 0.0001, 0.0083, 0.9999) | < 0.0001 (1.0000, 1.0000, 0.9012) |
| 3149 | 1.0000 (1.0000, 1.0000, 0.9230) | < 0.0001 (< 0.0001, 1.0000, 0.9945) |
| 3150 | < 0.0001 (0.6139, 0.0425, 0.9944) | 0.4722 (1.0000, 0.8971, 0.9931) |
| 3151 | < 0.0001 (0.5572, 0.0083, 0.9996) | < 0.0001 (1.0000, 1.0000, 0.8192) |
| 3152 | < 0.0001 (< 0.0001, 0.0041, 0.9905) | < 0.0001 (1.0000, 1.0000, 0.8864) |
| 3153 | 1.0000 (1.0000, 1.0000, 0.9870) | < 0.0001 (< 0.0001, 1.0000, 0.9480) |
| 3154 | 1.0000 (< 0.0001, 1.0000, 0.9870) | < 0.0001 (< 0.0001, 1.0000, 0.6394) |
| 3155 | < 0.0001 (< 0.0001, 0.0073, 0.9885) | < 0.0001 (1.0000, 1.0000, 0.9938) |
| 3156 | 1.0000 (< 0.0001, 1.0000, 0.9343) | < 0.0001 (< 0.0001, 1.0000, 0.9480) |
| 3157 | < 0.0001 (< 0.0001, 0.0073, 0.9885) | < 0.0001 (< 0.0001, 1.0000, 0.8714) |
| 3158 | 1.0000 (< 0.0001, 1.0000, 0.9216) | < 0.0001 (< 0.0001, 1.0000, 0.9608) |
| 3159 | 1.0000 (< 0.0001, 1.0000, 0.9525) | < 0.0001 (< 0.0001, 1.0000, 0.9532) |
| 3160 | 0.3170 (< 0.0001, 0.0782, 0.9918) | < 0.0001 (1.0000, 1.0000, 0.9524) |
| 3161 | < 0.0001 (< 0.0001, 0.0056, 0.9943) | < 0.0001 (1.0000, 1.0000, 0.9735) |
| 3162 | 0.3611 (< 0.0001, 0.1103, 0.9903) | < 0.0001 (1.0000, 1.0000, 0.9990) |
| 3163 | 1.3009 (< 0.0001, 0.3073, 0.1677) | < 0.0001 (0.6521, 1.0000, 0.9498) |
| 3164 | < 0.0001 (< 0.0001, 0.0065, 0.9981) | < 0.0001 (1.0000, 1.0000, 0.9780) |
| 3165 | < 0.0001 (< 0.0001, 0.0083, 0.9948) | < 0.0001 (1.0000, 1.0000, 0.9846) |
| 3166 | < 0.0001 (< 0.0001, 0.0073, 0.9995) | 0.1650 (1.0000, 0.9880, 1.0000) |
| 3167 | 1.0000 (< 0.0001, 1.0000, 0.9379) | < 0.0001 (< 0.0001, 1.0000, 0.9653) |
| 3168 | 0.3993 (< 0.0001, 0.1396, 0.9947) | < 0.0001 (1.0000, 1.0000, 0.8947) |
| 3169 | 1.8148 (< 0.0001, 0.0923, 0.9628) | < 0.0001 (0.8269, 1.0000, 0.9787) |
| 3170 | 1.0000 (< 0.0001, 1.0000, 0.9379) | < 0.0001 (< 0.0001, 1.0000, 0.8980) |
| 3171 | 0.3106 (< 0.0001, 0.0737, 0.8790) | < 0.0001 (0.8889, 1.0000, 0.9661) |
| 3172 | < 0.0001 (< 0.0001, 0.0060, 0.9998) | 0.4988 (1.0000, 0.8893, 0.9904) |
| 3173 | 1.0000 (0.8627, 1.0000, 0.9216) | < 0.0001 (< 0.0001, 1.0000, 0.9219) |
| 3174 | < 0.0001 (0.9997, 0.0093, 1.0000) | < 0.0001 (1.0000, 1.0000, 0.9694) |
| 3175 | 1.9051 (0.0118, 0.0772, 0.2214) | < 0.0001 (0.3158, 1.0000, 0.9746) |
| 3176 | < 0.0001 (0.9421, 0.0083, 0.9999) | < 0.0001 (1.0000, 1.0000, 0.8454) |
| 3177 | 1.0000 (0.1982, 1.0000, 0.9411) | < 0.0001 (< 0.0001, 1.0000, 0.9885) |
| 3178 | < 0.0001 (1.0000, 0.0050, 0.9963) | < 0.0001 (1.0000, 1.0000, 0.9984) |
| 3179 | 1.0000 (< 0.0001, 1.0000, 0.9798) | < 0.0001 (< 0.0001, 1.0000, 0.9522) |
| 3180 | < 0.0001 (< 0.0001, 0.0425, 0.9973) | < 0.0001 (1.0000, 1.0000, 0.9524) |
| 3181 | < 0.0001 (0.7056, 0.0117, 0.9996) | < 0.0001 (1.0000, 1.0000, 0.9889) |
| 3182 | 1.0000 (< 0.0001, 1.0000, 0.9404) | < 0.0001 (< 0.0001, 1.0000, 0.8494) |
| 3183 | < 0.0001 (< 0.0001, 0.0053, 0.9676) | < 0.0001 (< 0.0001, 1.0000, 0.8864) |
| 3184 | 1.0000 (< 0.0001, 1.0000, 0.9343) | < 0.0001 (< 0.0001, 1.0000, 0.8714) |
| 3185 | 0.3358 (< 0.0001, 0.0916, 0.9411) | < 0.0001 (0.6810, 1.0000, 0.9219) |
| 3186 | < 0.0001 (< 0.0001, 0.0073, 0.9994) | < 0.0001 (1.0000, 1.0000, 0.9719) |
| 3187 | < 0.0001 (0.7971, 0.0044, 0.9998) | < 0.0001 (1.0000, 1.0000, 0.9787) |
| 3188 | < 0.0001 (0.3823, 0.0126, 1.0000) | < 0.0001 (1.0000, 1.0000, 0.9981) |
| 3189 | 1.1166 (0.8996, 0.4143, 0.5977) | 0.3506 (0.4906, 0.9508, 0.9429) |
| 3190 | < 0.0001 (0.0026, 0.0085, 0.9999) | < 0.0001 (1.0000, 1.0000, 0.8999) |
| 3191 | 1.0000 (0.0022, 1.0000, 0.9870) | < 0.0001 (< 0.0001, 1.0000, 0.9971) |
| 3192 | < 0.0001 (0.3209, 0.0061, 0.9978) | < 0.0001 (1.0000, 1.0000, 0.9952) |
| 3193 | < 0.0001 (0.8998, 0.0083, 1.0000) | 0.3333 (1.0000, 0.9547, 0.7202) |
| 3194 | < 0.0001 (0.5788, 0.0037, 0.9695) | < 0.0001 (1.0000, 1.0000, 0.9764) |
| 3195 | 1.0000 (< 0.0001, 1.0000, 0.9216) | < 0.0001 (< 0.0001, 1.0000, 0.9524) |
| 3196 | < 0.0001 (< 0.0001, 0.0065, 0.9651) | < 0.0001 (1.0000, 1.0000, 0.9962) |
| 3197 | < 0.0001 (< 0.0001, 0.0065, 1.0000) | < 0.0001 (1.0000, 1.0000, 0.8015) |
| 3198 | < 0.0001 (0.7553, 0.0087, 0.9988) | < 0.0001 (1.0000, 1.0000, 0.9694) |
| 3199 | < 0.0001 (< 0.0001, 0.0083, 0.9987) | < 0.0001 (< 0.0001, 1.0000, 0.9480) |
| 3200 | < 0.0001 (< 0.0001, 0.0083, 0.9995) | < 0.0001 (1.0000, 1.0000, 0.9889) |
| 3201 | < 0.0001 (0.0561, 0.0060, 0.9904) | < 0.0001 (1.0000, 1.0000, 0.9680) |
| 3202 | < 0.0001 (< 0.0001, 0.0093, 1.0000) | < 0.0001 (1.0000, 1.0000, 0.9999) |
| 3203 | < 0.0001 (< 0.0001, 0.0424, 0.9999) | < 0.0001 (1.0000, 1.0000, 0.8020) |
| 3204 | 1.0000 (0.8297, 1.0000, 0.8612) | < 0.0001 (< 0.0001, 1.0000, 0.9878) |
| 3205 | < 0.0001 (0.1221, 0.0065, 0.9996) | < 0.0001 (1.0000, 1.0000, 0.9866) |
| 3206 | 1.0000 (0.5596, 1.0000, 0.8772) | < 0.0001 (< 0.0001, 1.0000, 0.9573) |
| 3207 | < 0.0001 (0.2293, 0.0117, 0.9989) | < 0.0001 (< 0.0001, 1.0000, 0.9938) |
| 3208 | < 0.0001 (< 0.0001, 0.0061, 0.9987) | < 0.0001 (1.0000, 1.0000, 0.9866) |
| 3209 | < 0.0001 (< 0.0001, 0.0050, 0.9998) | < 0.0001 (1.0000, 1.0000, 0.9019) |
| 3210 | < 0.0001 (0.8855, 0.0120, 1.0000) | < 0.0001 (1.0000, 1.0000, 0.9927) |
| 3211 | < 0.0001 (0.4429, 0.0126, 0.9997) | < 0.0001 (1.0000, 1.0000, 0.9977) |
| 3212 | 0.3686 (0.0666, 0.1160, 0.9739) | 0.9201 (1.0000, 0.7280, 0.9962) |
| 3213 | < 0.0001 (0.1951, 0.0117, 0.9993) | < 0.0001 (1.0000, 1.0000, 0.9608) |
| 3214 | < 0.0001 (0.4424, 0.0126, 0.9898) | < 0.0001 (1.0000, 1.0000, 0.9661) |
| 3215 | < 0.0001 (< 0.0001, 0.0053, 0.9995) | < 0.0001 (1.0000, 1.0000, 0.9480) |
| 3216 | 0.3187 (< 0.0001, 0.0794, 1.0000) | < 0.0001 (1.0000, 1.0000, 0.5347) |
| 3217 | 1.0000 (< 0.0001, 1.0000, 0.9870) | < 0.0001 (< 0.0001, 1.0000, 0.9963) |
| 3218 | 1.0000 (< 0.0001, 1.0000, 0.9536) | < 0.0001 (< 0.0001, 1.0000, 0.9983) |
| 3219 | 0.3168 (< 0.0001, 0.0781, 0.8957) | < 0.0001 (< 0.0001, 1.0000, 0.9945) |
| 3220 | < 0.0001 (< 0.0001, 0.0083, 0.9921) | < 0.0001 (1.0000, 1.0000, 1.0000) |
| 3221 | < 0.0001 (0.8083, 0.0085, 0.9997) | < 0.0001 (1.0000, 1.0000, 0.8626) |
| 3222 | < 0.0001 (< 0.0001, 0.0060, 0.9995) | < 0.0001 (1.0000, 1.0000, 0.9746) |
| 3223 | < 0.0001 (< 0.0001, 0.0065, 0.9894) | < 0.0001 (1.0000, 1.0000, 0.9902) |
| 3224 | 0.3170 (< 0.0001, 0.0782, 0.8731) | < 0.0001 (0.8999, 1.0000, 0.9448) |
| 3225 | < 0.0001 (< 0.0001, 0.0343, 0.9964) | < 0.0001 (1.0000, 1.0000, 0.9937) |
| 3226 | < 0.0001 (0.9216, 0.0073, 0.9964) | < 0.0001 (1.0000, 1.0000, 0.9860) |
| 3227 | 1.0000 (0.8154, 1.0000, 0.9798) | < 0.0001 (< 0.0001, 1.0000, 0.9944) |
| 3228 | 1.0000 (0.7329, 1.0000, 0.9379) | < 0.0001 (< 0.0001, 1.0000, 0.9661) |
| 3229 | < 0.0001 (0.6509, 0.0093, 0.9974) | < 0.0001 (1.0000, 1.0000, 0.9962) |
| 3230 | < 0.0001 (1.0000, 0.0131, 0.9963) | < 0.0001 (1.0000, 1.0000, 0.9974) |
| 3231 | 0.6031 (< 0.0001, 0.2176, 0.8322) | < 0.0001 (0.9630, 1.0000, 0.8669) |
| 3232 | 1.0000 (< 0.0001, 1.0000, 0.9216) | < 0.0001 (< 0.0001, 1.0000, 0.9841) |
| 3233 | 1.0000 (< 0.0001, 1.0000, 0.9798) | < 0.0001 (< 0.0001, 1.0000, 0.9950) |
| 3234 | 1.6677 (< 0.0001, 0.1732, 0.2506) | < 0.0001 (0.9039, 1.0000, 0.9955) |
| 3235 | < 0.0001 (< 0.0001, 0.0065, 0.9998) | 0.4865 (1.0000, 0.6852, 0.8055) |
| 3236 | 1.0000 (< 0.0001, 1.0000, 0.9798) | < 0.0001 (< 0.0001, 1.0000, 0.9876) |
| 3237 | < 0.0001 (< 0.0001, 0.0343, 0.9817) | 0.2000 (< 0.0001, 0.9931, 0.8436) |
| 3238 | < 0.0001 (< 0.0001, 0.0343, 0.9817) | < 0.0001 (1.0000, 1.0000, 0.9299) |
| 3239 | < 0.0001 (< 0.0001, 0.0083, 0.9971) | < 0.0001 (1.0000, 1.0000, 0.2952) |
| 3240 | 1.0000 (< 0.0001, 1.0000, 0.9552) | < 0.0001 (< 0.0001, 1.0000, 0.9480) |
| 3241 | < 0.0001 (< 0.0001, 0.0117, 0.9963) | < 0.0001 (1.0000, 1.0000, 0.9724) |
| 3242 | < 0.0001 (< 0.0001, 0.0087, 1.0000) | < 0.0001 (1.0000, 1.0000, 0.9975) |
| 3243 | 1.0000 (< 0.0001, 1.0000, 0.9536) | < 0.0001 (< 0.0001, 1.0000, 0.9774) |
| 3244 | < 0.0001 (< 0.0001, 0.0053, 0.9958) | < 0.0001 (1.0000, 1.0000, 0.8864) |
| 3245 | < 0.0001 (< 0.0001, 0.0099, 0.9998) | < 0.0001 (1.0000, 1.0000, 0.9746) |
| 3246 | < 0.0001 (< 0.0001, 0.0117, 1.0000) | < 0.0001 (1.0000, 1.0000, 0.9774) |
| 3247 | < 0.0001 (< 0.0001, 0.0050, 0.9817) | < 0.0001 (1.0000, 1.0000, 0.9971) |
| 3248 | 1.0000 (< 0.0001, 1.0000, 0.9351) | < 0.0001 (< 0.0001, 1.0000, 0.9480) |
| 3249 | < 0.0001 (< 0.0001, 0.0397, 0.9995) | 0.2566 (1.0000, 0.9610, 0.9852) |
| 3250 | 0.3170 (< 0.0001, 0.0782, 0.9969) | 0.5134 (0.9900, 0.6608, 0.9709) |
| 3251 | 1.0000 (< 0.0001, 1.0000, 0.9870) | < 0.0001 (< 0.0001, 1.0000, 0.9589) |
| 3252 | < 0.0001 (< 0.0001, 0.0060, 0.9966) | < 0.0001 (1.0000, 1.0000, 0.9349) |
| 3253 | < 0.0001 (< 0.0001, 0.0065, 0.9856) | < 0.0001 (1.0000, 1.0000, 0.8466) |
| 3254 | < 0.0001 (< 0.0001, 0.0060, 1.0000) | < 0.0001 (1.0000, 1.0000, 0.4740) |
| 3255 | < 0.0001 (< 0.0001, 0.0044, 0.9964) | < 0.0001 (1.0000, 1.0000, 0.8952) |
| 3256 | 0.3013 (< 0.0001, 0.0674, 0.9489) | < 0.0001 (0.8893, 1.0000, 0.9480) |
| 3257 | < 0.0001 (< 0.0001, 0.0424, 0.9993) | < 0.0001 (1.0000, 1.0000, 0.9661) |
| 3258 | < 0.0001 (< 0.0001, 0.0083, 0.9999) | < 0.0001 (1.0000, 1.0000, 0.9378) |
| 3259 | < 0.0001 (< 0.0001, 0.0083, 0.9772) | < 0.0001 (1.0000, 1.0000, 0.9378) |
| 3260 | 1.0000 (< 0.0001, 1.0000, 0.9552) | < 0.0001 (< 0.0001, 1.0000, 0.8952) |
| 3261 | < 0.0001 (< 0.0001, 0.0087, 0.9997) | < 0.0001 (1.0000, 1.0000, 0.9752) |
| 3262 | 1.0000 (< 0.0001, 1.0000, 0.8849) | < 0.0001 (< 0.0001, 1.0000, 0.9661) |
| 3263 | 1.0000 (< 0.0001, 1.0000, 0.9216) | < 0.0001 (< 0.0001, 1.0000, 0.9994) |
| 3264 | 1.0000 (< 0.0001, 1.0000, 0.9230) | < 0.0001 (< 0.0001, 1.0000, 0.9961) |
| 3265 | < 0.0001 (< 0.0001, 0.0065, 0.9651) | < 0.0001 (1.0000, 1.0000, 0.9998) |
| 3266 | < 0.0001 (< 0.0001, 0.0073, 0.9885) | < 0.0001 (1.0000, 1.0000, 0.9480) |
| 3267 | < 0.0001 (< 0.0001, 0.0397, 0.9960) | < 0.0001 (1.0000, 1.0000, 0.9500) |
| 3268 | < 0.0001 (0.6813, 0.0083, 1.0000) | < 0.0001 (1.0000, 1.0000, 0.9837) |
| 3269 | < 0.0001 (< 0.0001, 0.0056, 0.9971) | < 0.0001 (1.0000, 1.0000, 0.9913) |
| 3270 | 1.0000 (< 0.0001, 1.0000, 0.9342) | < 0.0001 (< 0.0001, 1.0000, 0.9480) |
| 3271 | 0.3186 (< 0.0001, 0.0793, 0.9925) | < 0.0001 (1.0000, 1.0000, 0.9618) |
| 3272 | < 0.0001 (< 0.0001, 0.0085, 1.0000) | < 0.0001 (1.0000, 1.0000, 0.8952) |
| 3273 | 0.2200 (< 0.0001, 0.0220, 0.9125) | < 0.0001 (0.9824, 1.0000, 0.9891) |
| 3274 | < 0.0001 (< 0.0001, 0.0107, 0.9960) | < 0.0001 (1.0000, 1.0000, 0.7404) |
| 3275 | 1.0000 (< 0.0001, 1.0000, 0.9552) | < 0.0001 (< 0.0001, 1.0000, 0.9719) |
| 3276 | < 0.0001 (< 0.0001, 0.0056, 1.0000) | < 0.0001 (1.0000, 1.0000, 0.9480) |
| 3277 | 1.2775 (< 0.0001, 0.3192, 0.8983) | < 0.0001 (0.9547, 1.0000, 0.9661) |
| 3278 | < 0.0001 (0.0130, 0.0060, 0.9947) | 0.5007 (1.0000, 0.8887, 0.9720) |
| 3279 | 1.0000 (0.3129, 1.0000, 0.9798) | < 0.0001 (< 0.0001, 1.0000, 0.9448) |
| 3280 | < 0.0001 (0.8729, 0.0053, 0.9958) | 0.2319 (1.0000, 0.9682, 0.9012) |
| 3281 | < 0.0001 (0.9367, 0.0083, 0.9999) | < 0.0001 (1.0000, 1.0000, 0.9985) |
| 3282 | < 0.0001 (< 0.0001, 0.0085, 1.0000) | < 0.0001 (0.0176, 1.0000, 0.8937) |
| 3283 | < 0.0001 (< 0.0001, 0.0065, 0.9733) | < 0.0001 (< 0.0001, 1.0000, 0.9299) |
| 3284 | < 0.0001 (< 0.0001, 0.0050, 0.9992) | < 0.0001 (1.0000, 1.0000, 0.8722) |
| 3285 | < 0.0001 (< 0.0001, 0.0061, 0.9991) | < 0.0001 (1.0000, 1.0000, 0.9012) |
| 3286 | < 0.0001 (< 0.0001, 0.0087, 0.9926) | < 0.0001 (1.0000, 1.0000, 0.9480) |
| 3287 | < 0.0001 (< 0.0001, 0.0424, 0.9998) | < 0.0001 (1.0000, 1.0000, 0.9325) |
| 3288 | < 0.0001 (< 0.0001, 0.0061, 0.9937) | 1.0270 (1.0000, 0.4364, 0.9987) |
| 3289 | 1.0000 (< 0.0001, 1.0000, 0.9431) | < 0.0001 (< 0.0001, 1.0000, 0.8478) |
| 3290 | < 0.0001 (< 0.0001, 0.0107, 0.9960) | < 0.0001 (1.0000, 1.0000, 0.9939) |
| 3291 | < 0.0001 (< 0.0001, 0.0142, 0.9997) | < 0.0001 (1.0000, 1.0000, 0.9916) |
| 3292 | < 0.0001 (< 0.0001, 0.0061, 0.9998) | < 0.0001 (1.0000, 1.0000, 0.5135) |
| 3293 | < 0.0001 (0.8112, 0.0343, 0.9967) | < 0.0001 (1.0000, 1.0000, 0.8864) |
| 3294 | 1.0000 (0.9093, 1.0000, 0.9870) | < 0.0001 (< 0.0001, 1.0000, 0.9747) |
| 3295 | 1.0000 (0.9831, 1.0000, 0.9798) | < 0.0001 (< 0.0001, 1.0000, 0.9528) |
| 3296 | 1.3507 (< 0.0001, 0.3429, 0.9898) | < 0.0001 (0.4685, 1.0000, 0.9717) |
| 3297 | < 0.0001 (< 0.0001, 0.0126, 0.9998) | < 0.0001 (1.0000, 1.0000, 0.9449) |
| 3298 | < 0.0001 (0.6227, 0.0424, 0.9996) | < 0.0001 (1.0000, 1.0000, 0.9996) |
| 3299 | < 0.0001 (0.6341, 0.0142, 1.0000) | < 0.0001 (1.0000, 1.0000, 0.9480) |
| 3300 | < 0.0001 (< 0.0001, 0.0117, 1.0000) | < 0.0001 (1.0000, 1.0000, 0.7975) |
| 3301 | 1.0000 (0.4145, 1.0000, 0.8897) | < 0.0001 (< 0.0001, 1.0000, 0.9930) |
| 3302 | < 0.0001 (0.9505, 0.0044, 0.9964) | < 0.0001 (1.0000, 1.0000, 0.8184) |
| 3303 | < 0.0001 (0.8080, 0.0044, 0.9986) | < 0.0001 (1.0000, 1.0000, 0.9768) |
| 3304 | < 0.0001 (0.5412, 0.0065, 0.9976) | 2.0000 (1.0000, 0.4609, 0.9931) |
| 3305 | < 0.0001 (0.8182, 0.0425, 0.9986) | 1.0348 (1.0000, 0.7414, 0.7945) |
| 3306 | < 0.0001 (0.4465, 0.0095, 0.9981) | < 0.0001 (1.0000, 1.0000, 0.9894) |
| 3307 | < 0.0001 (< 0.0001, 0.0126, 0.9992) | < 0.0001 (1.0000, 1.0000, 0.9746) |
| 3308 | 1.0000 (0.4854, 1.0000, 0.9870) | < 0.0001 (< 0.0001, 1.0000, 0.2024) |
| 3309 | < 0.0001 (0.4960, 0.0061, 0.9987) | < 0.0001 (1.0000, 1.0000, 0.6276) |
| 3310 | < 0.0001 (0.4337, 0.0053, 1.0000) | < 0.0001 (1.0000, 1.0000, 0.9756) |
| 3311 | < 0.0001 (0.7203, 0.0061, 0.9867) | < 0.0001 (< 0.0001, 1.0000, 0.9971) |
| 3312 | 0.3170 (< 0.0001, 0.0782, 0.9841) | < 0.0001 (1.0000, 1.0000, 0.9480) |
| 3313 | < 0.0001 (0.6441, 0.0087, 0.9997) | < 0.0001 (1.0000, 1.0000, 0.9349) |
| 3314 | < 0.0001 (0.4321, 0.0107, 0.9999) | < 0.0001 (1.0000, 1.0000, 0.9719) |
| 3315 | < 0.0001 (< 0.0001, 0.0061, 0.9937) | < 0.0001 (< 0.0001, 1.0000, 0.9780) |
| 3316 | < 0.0001 (< 0.0001, 0.0083, 0.9982) | < 0.0001 (1.0000, 1.0000, 0.9950) |
| 3317 | 1.0000 (0.0061, 1.0000, 0.8619) | < 0.0001 (< 0.0001, 1.0000, 0.8714) |
| 3318 | 1.0000 (0.4448, 1.0000, 0.9525) | < 0.0001 (< 0.0001, 1.0000, 0.9859) |
| 3319 | 0.6135 (0.8942, 0.2262, 0.9121) | < 0.0001 (0.7005, 1.0000, 0.9866) |
| 3320 | 1.0000 (< 0.0001, 1.0000, 0.9798) | < 0.0001 (< 0.0001, 1.0000, 0.9996) |
| 3321 | 0.6964 (0.4202, 0.2932, 0.9999) | < 0.0001 (0.9974, 1.0000, 0.9889) |
| 3322 | < 0.0001 (0.0005, 0.0093, 1.0000) | < 0.0001 (1.0000, 1.0000, 0.9613) |
| 3323 | 0.6029 (< 0.0001, 0.2175, 0.8785) | 0.0353 (1.0000, 1.0000, 1.0000) |
| 3324 | 1.0000 (0.2187, 1.0000, 0.9216) | < 0.0001 (< 0.0001, 1.0000, 0.9746) |
| 3325 | < 0.0001 (0.9981, 0.0044, 0.9964) | 0.4596 (1.0000, 0.9046, 0.9349) |
| 3326 | < 0.0001 (0.8705, 0.0050, 0.9874) | < 0.0001 (1.0000, 1.0000, 1.0000) |
| 3327 | < 0.0001 (0.0810, 0.0050, 0.9991) | 0.9305 (1.0000, 0.7616, 1.0000) |
| 3328 | 1.0000 (0.4687, 1.0000, 0.9798) | < 0.0001 (< 0.0001, 1.0000, 0.8864) |
| 3329 | < 0.0001 (0.3104, 0.0107, 0.9907) | < 0.0001 (1.0000, 1.0000, 0.3224) |
| 3330 | 0.3170 (< 0.0001, 0.0782, 0.8731) | < 0.0001 (1.0000, 1.0000, 0.8952) |
| 3331 | < 0.0001 (< 0.0001, 0.0099, 0.9798) | 0.0592 (1.0000, 1.0000, 0.5686) |
| 3332 | 0.6376 (< 0.0001, 0.2459, 0.8650) | < 0.0001 (< 0.0001, 1.0000, 0.9541) |
| 3333 | 1.2713 (< 0.0001, 0.3224, 0.4743) | 0.2298 (0.9620, 0.9688, 0.9859) |
| 3334 | 0.6350 (< 0.0001, 0.2438, 0.4130) | < 0.0001 (0.6638, 1.0000, 0.9817) |
| 3335 | 1.0000 (0.0329, 1.0000, 0.9404) | < 0.0001 (< 0.0001, 1.0000, 0.9999) |
| 3336 | 1.0000 (0.2373, 1.0000, 0.9798) | < 0.0001 (< 0.0001, 1.0000, 0.9661) |
| 3337 | 1.0000 (0.9990, 1.0000, 0.9870) | < 0.0001 (< 0.0001, 1.0000, 0.7881) |
| 3338 | 1.0000 (0.4985, 1.0000, 0.9490) | < 0.0001 (< 0.0001, 1.0000, 0.9498) |
| 3339 | 0.3013 (< 0.0001, 0.0674, 0.7951) | < 0.0001 (< 0.0001, 1.0000, 0.9980) |
| 3340 | < 0.0001 (< 0.0001, 0.0073, 0.9894) | 0.4619 (< 0.0001, 0.9037, 0.9746) |
| 3341 | 0.3248 (< 0.0001, 0.0837, 0.9890) | 0.9889 (1.0000, 0.7440, 0.9746) |
| 3342 | 1.0000 (0.2519, 1.0000, 0.9870) | 1.9996 (< 0.0001, 0.4610, 0.7906) |
| 3343 | < 0.0001 (0.1511, 0.0397, 0.9999) | < 0.0001 (1.0000, 1.0000, 0.9719) |
| 3344 | < 0.0001 (0.9515, 0.0073, 0.9998) | < 0.0001 (1.0000, 1.0000, 0.5272) |
| 3345 | < 0.0001 (0.8150, 0.0083, 1.0000) | < 0.0001 (1.0000, 1.0000, 0.6997) |
| 3346 | 1.0000 (1.0000, 1.0000, 0.9798) | < 0.0001 (< 0.0001, 1.0000, 0.2340) |
| 3347 | < 0.0001 (0.0893, 0.0053, 0.9958) | < 0.0001 (1.0000, 1.0000, 0.9970) |
| 3348 | < 0.0001 (0.0820, 0.0085, 0.9994) | < 0.0001 (1.0000, 1.0000, 0.9521) |
| 3349 | < 0.0001 (0.7650, 0.0083, 0.9997) | < 0.0001 (1.0000, 1.0000, 0.8952) |
| 3350 | 1.0000 (0.4135, 1.0000, 0.9798) | < 0.0001 (< 0.0001, 1.0000, 0.9873) |
| 3351 | 1.0000 (< 0.0001, 1.0000, 0.9816) | < 0.0001 (< 0.0001, 1.0000, 0.9378) |
| 3352 | < 0.0001 (< 0.0001, 0.0073, 0.9964) | < 0.0001 (< 0.0001, 1.0000, 0.9231) |
| 3353 | < 0.0001 (< 0.0001, 0.0073, 0.9964) | < 0.0001 (1.0000, 1.0000, 1.0000) |
| 3354 | 1.0000 (< 0.0001, 1.0000, 0.8264) | < 0.0001 (< 0.0001, 1.0000, 0.9891) |
| 3355 | 0.3358 (< 0.0001, 0.0916, 0.8794) | < 0.0001 (1.0000, 1.0000, 0.7404) |
| 3356 | 1.0000 (< 0.0001, 1.0000, 0.9798) | < 0.0001 (< 0.0001, 1.0000, 0.9719) |
| 3357 | 1.0000 (< 0.0001, 1.0000, 0.9870) | < 0.0001 (< 0.0001, 1.0000, 0.9480) |
| 3358 | < 0.0001 (< 0.0001, 0.0073, 0.9885) | < 0.0001 (1.0000, 1.0000, 0.9661) |
| 3359 | < 0.0001 (< 0.0001, 0.0065, 0.9976) | < 0.0001 (1.0000, 1.0000, 0.9720) |
| 3360 | < 0.0001 (< 0.0001, 0.0060, 0.9987) | < 0.0001 (< 0.0001, 1.0000, 0.9448) |
| 3361 | < 0.0001 (< 0.0001, 0.0050, 0.9996) | < 0.0001 (1.0000, 1.0000, 0.9012) |
| 3362 | 0.3611 (< 0.0001, 0.1103, 0.9971) | 0.5007 (0.8887, 0.8887, 0.9985) |
| 3363 | 1.0000 (< 0.0001, 1.0000, 0.9525) | < 0.0001 (< 0.0001, 1.0000, 0.8937) |
| 3364 | 0.3358 (< 0.0001, 0.0916, 0.9960) | 0.2319 (0.9676, 0.9682, 0.9299) |
| 3365 | 1.0000 (1.0000, 1.0000, 0.9379) | < 0.0001 (< 0.0001, 1.0000, 0.8722) |
| 3366 | 1.0000 (0.6503, 1.0000, 0.9798) | < 0.0001 (< 0.0001, 1.0000, 0.9012) |
| 3367 | < 0.0001 (< 0.0001, 0.0037, 0.9880) | < 0.0001 (1.0000, 1.0000, 0.9480) |
| 3368 | < 0.0001 (< 0.0001, 0.0065, 0.9998) | < 0.0001 (1.0000, 1.0000, 0.9325) |
| 3369 | < 0.0001 (< 0.0001, 0.0083, 0.9949) | < 0.0001 (< 0.0001, 1.0000, 0.9987) |
| 3370 | 0.3611 (< 0.0001, 0.1103, 0.9903) | < 0.0001 (1.0000, 1.0000, 0.8478) |
| 3371 | < 0.0001 (0.3403, 0.0142, 0.9997) | < 0.0001 (1.0000, 1.0000, 0.9939) |
| 3372 | 1.6235 (0.2332, 0.1851, 0.4052) | 1.0270 (0.4288, 0.4364, 0.9916) |
| 3373 | 0.3173 (0.6591, 0.0784, 0.8550) | < 0.0001 (< 0.0001, 1.0000, 0.5135) |
| 3374 | < 0.0001 (0.9998, 0.0060, 0.9966) | < 0.0001 (1.0000, 1.0000, 0.8864) |
| 3375 | < 0.0001 (0.8843, 0.0313, 0.9862) | < 0.0001 (1.0000, 1.0000, 0.9747) |
| 3376 | < 0.0001 (0.4592, 0.0073, 0.9894) | < 0.0001 (1.0000, 1.0000, 0.9528) |
| 3377 | < 0.0001 (0.8319, 0.0065, 0.9952) | < 0.0001 (1.0000, 1.0000, 0.9717) |
| 3378 | < 0.0001 (< 0.0001, 0.0060, 1.0000) | < 0.0001 (1.0000, 1.0000, 0.9449) |
| 3379 | 1.0000 (< 0.0001, 1.0000, 0.9798) | < 0.0001 (< 0.0001, 1.0000, 0.9996) |
| 3380 | 1.0000 (< 0.0001, 1.0000, 0.8511) | < 0.0001 (< 0.0001, 1.0000, 0.9480) |
| 3381 | < 0.0001 (< 0.0001, 0.0056, 0.9991) | < 0.0001 (1.0000, 1.0000, 0.7975) |
| 3382 | < 0.0001 (< 0.0001, 0.0037, 0.9914) | < 0.0001 (< 0.0001, 1.0000, 0.9930) |
| 3383 | < 0.0001 (< 0.0001, 0.0425, 0.9872) | < 0.0001 (1.0000, 1.0000, 0.8184) |
| 3384 | 0.3170 (< 0.0001, 0.0782, 0.9998) | < 0.0001 (1.0000, 1.0000, 0.9768) |
| 3385 | < 0.0001 (< 0.0001, 0.0056, 0.9876) | < 0.0001 (< 0.0001, 1.0000, 0.9931) |
| 3386 | < 0.0001 (< 0.0001, 0.0050, 1.0000) | < 0.0001 (1.0000, 1.0000, 0.7945) |
| 3387 | < 0.0001 (0.8462, 0.0044, 0.9999) | < 0.0001 (1.0000, 1.0000, 0.9894) |
| 3388 | 3.8912 (0.9034, < 0.0001, 0.1435) | 2.0000 (0.3512, 0.4609, 0.9746) |
| 3389 | 0.5771 (0.8270, 0.2727, 0.8916) | 1.0348 (0.7508, 0.7414, 0.2024) |
| 3390 | < 0.0001 (0.2605, 0.0061, 0.9987) | < 0.0001 (1.0000, 1.0000, 0.6276) |
| 3391 | < 0.0001 (0.3508, 0.0050, 0.9991) | < 0.0001 (1.0000, 1.0000, 0.9756) |
| 3392 | 1.0000 (0.7597, 1.0000, 0.9798) | < 0.0001 (< 0.0001, 1.0000, 0.9971) |
| 3393 | 1.0000 (0.2712, 1.0000, 0.9480) | < 0.0001 (< 0.0001, 1.0000, 0.9480) |
| 3394 | 0.3358 (0.6186, 0.0916, 0.8795) | < 0.0001 (1.0000, 1.0000, 0.9349) |
| 3395 | 0.3067 (0.4232, 0.0710, 0.9378) | < 0.0001 (1.0000, 1.0000, 0.9719) |
| 3396 | < 0.0001 (0.7421, 0.0060, 1.0000) | < 0.0001 (1.0000, 1.0000, 0.9780) |
| 3397 | < 0.0001 (0.9381, 0.0343, 0.9817) | < 0.0001 (< 0.0001, 1.0000, 0.9950) |
| 3398 | < 0.0001 (0.0380, 0.0083, 0.9987) | < 0.0001 (1.0000, 1.0000, 0.8714) |
| 3399 | < 0.0001 (0.6700, 0.0061, 0.9978) | < 0.0001 (1.0000, 1.0000, 0.9859) |
| 3400 | < 0.0001 (0.0666, 0.0060, 1.0000) | < 0.0001 (1.0000, 1.0000, 0.9866) |
| 3401 | < 0.0001 (0.2069, 0.0053, 0.9959) | < 0.0001 (1.0000, 1.0000, 0.9996) |
| 3402 | < 0.0001 (0.8421, 0.0343, 0.9964) | < 0.0001 (1.0000, 1.0000, 0.9889) |
| 3403 | < 0.0001 (0.5082, 0.0083, 1.0000) | < 0.0001 (1.0000, 1.0000, 0.9613) |
| 3404 | < 0.0001 (< 0.0001, 0.0044, 0.9964) | < 0.0001 (1.0000, 1.0000, 1.0000) |
| 3405 | < 0.0001 (0.2419, 0.0061, 0.9948) | < 0.0001 (< 0.0001, 1.0000, 0.9746) |
| 3406 | < 0.0001 (0.6844, 0.0060, 1.0000) | < 0.0001 (1.0000, 1.0000, 0.9349) |
| 3407 | 0.3170 (0.9759, 0.0782, 1.0000) | 0.0353 (1.0000, 1.0000, 1.0000) |
| 3408 | 1.0000 (< 0.0001, 1.0000, 0.9216) | < 0.0001 (< 0.0001, 1.0000, 1.0000) |
| 3409 | 1.3002 (< 0.0001, 0.3077, 0.3976) | 0.4596 (0.7846, 0.9046, 0.8864) |
| 3410 | 1.0000 (< 0.0001, 1.0000, 0.9404) | < 0.0001 (< 0.0001, 1.0000, 0.3224) |
| 3411 | 0.6339 (< 0.0001, 0.2429, 0.9194) | 0.9305 (0.7595, 0.7616, 0.8952) |
| 3412 | < 0.0001 (< 0.0001, 0.0142, 0.9915) | < 0.0001 (1.0000, 1.0000, 0.5686) |
| 3413 | 0.6965 (< 0.0001, 0.2933, 0.8538) | < 0.0001 (1.0000, 1.0000, 0.9541) |
| 3414 | < 0.0001 (0.0005, 0.0065, 0.9994) | < 0.0001 (1.0000, 1.0000, 0.9859) |
| 3415 | 0.8329 (0.3589, 0.3949, 1.0000) | 0.0592 (1.0000, 1.0000, 0.9817) |
| 3416 | 1.0000 (0.1294, 1.0000, 0.9870) | < 0.0001 (< 0.0001, 1.0000, 0.9999) |
| 3417 | 0.5577 (0.8260, 0.1803, 0.9225) | 0.2298 (0.9919, 0.9688, 0.9661) |
| 3418 | 0.4047 (0.7387, 0.1437, 0.9608) | < 0.0001 (< 0.0001, 1.0000, 0.7881) |
| 3419 | < 0.0001 (0.7278, 0.0425, 0.9998) | < 0.0001 (1.0000, 1.0000, 0.9498) |
| 3420 | < 0.0001 (0.2009, 0.0060, 0.9969) | < 0.0001 (1.0000, 1.0000, 0.9980) |
| 3421 | < 0.0001 (< 0.0001, 0.0044, 0.9986) | < 0.0001 (1.0000, 1.0000, 0.9746) |
| 3422 | 1.0000 (0.0275, 1.0000, 0.8456) | < 0.0001 (< 0.0001, 1.0000, 0.9746) |
| 3423 | 0.3358 (0.6141, 0.0916, 0.8794) | < 0.0001 (1.0000, 1.0000, 0.7906) |
| 3424 | 0.6492 (0.4652, 0.2554, 0.8465) | 0.4619 (0.9167, 0.9037, 0.9719) |
| 3425 | 0.9038 (0.2551, 0.4293, 0.7471) | 0.9889 (0.7447, 0.7440, 0.5272) |
| 3426 | 1.8136 (0.4849, 0.0925, 0.4064) | 1.9996 (0.4610, 0.4610, 0.6997) |
| 3427 | < 0.0001 (0.1277, 0.0397, 0.9998) | < 0.0001 (1.0000, 1.0000, 0.2340) |
| 3428 | < 0.0001 (0.5028, 0.0083, 0.9772) | < 0.0001 (1.0000, 1.0000, 0.9970) |
| 3429 | 1.0000 (0.3936, 1.0000, 0.9404) | < 0.0001 (< 0.0001, 1.0000, 0.9521) |
| 3430 | 0.3246 (0.0894, 0.0836, 0.9818) | < 0.0001 (1.0000, 1.0000, 0.8952) |
| 3431 | < 0.0001 (0.5950, 0.0050, 0.9951) | < 0.0001 (< 0.0001, 1.0000, 0.9873) |
| 3432 | 1.0000 (0.7886, 1.0000, 0.9440) | < 0.0001 (< 0.0001, 1.0000, 0.9378) |
| 3433 | < 0.0001 (0.9990, 0.0421, 1.0000) | < 0.0001 (1.0000, 1.0000, 0.9231) |
