# Supplementary material for: Evolutionary dynamics and molecular epidemiology of West Nile virus in New York State: 1999–2015
Source: Virus Evol. 2019 Jul 21;5(2):vez020. doi: 10.1093/ve/vez020 (PMC6642743; doi:10.1093/ve/vez020)
Supplement: vez020_Supplementary_Data [file vez020_supplementary_data.zip › Supplementary Table 2.docx]

**Supplementary Table 2.** Sequencing primers used for amplicon generation and library preparation for deep sequencing.

| **Amplicon** | **F. Name** | **F. Sequence** | **R. Name** | **R. Sequence** | **Size** |
| --- | --- | --- | --- | --- | --- |
| 1 | WNV01F | AGTAGTTCGCCTGTGTGARCTG | WNV3029R | ACATTCAGTYGTGTTGCTYTCTC | 3028 |
| 2 | WNV77F | TGTGAGGATTAAYAACAATTAACAC | WNV3218R | CCATCGCCCCAYAARGTRTGC | 3141 |
| 3 | WNV2442F | GCTYTTCCTCTCMGTGAACG | WNV5783R | ATTTTGGGTACTCMGTCTCR | 3341 |
| 4 | WNV2628F | AGTGTGCGGTYTACGRTCAG | WNV6041R | ARTCGTCYTCATTCGTGTGC | 3413 |
| 5 | WNV5304F | RATGGCTGAAGCMYTGAGAG | WNV8441R | TTCCATTCTTCCYARRAGCACC | 3137 |
| 5a | WNV5199F | TGYTGAGGAARAAACAGATCACT | WNV8518R | CCKSGTTCCACTTCCCAAG | 3319 |
| 6 | WNV5571F | AGGYACYTCAGATCCATTCC | WNV8832R | GCACRTACTTCACTCCTTCTG | 3261 |
| 6a | WNV5683F | YTGGAACWCTGGATAYGAATG | WNV8736R | GTGATRGTGTCCCATGGYTT | 3053 |
| 7 | WNV7983F | YTGGTGYTACTAYATGGCAACC | WNV11172R | ARCASARGATCTCCTAGTCTATCC | 3189 |
| 8 | WNV8351F | AAAYCCRCTCTCACGRAAYTCC | WNV11244R | ATCCTGTGTTCTCGCACCACCAG | 2893 |
| 8a | WNV8237F | TGCACCGAGGRCCAAGGG | WNV10972R | ATCYCCTAGTCYATCCCAGG | 2735 |
