# Supplementary material for: Evolutionary dynamics and molecular epidemiology of West Nile virus in New York State: 1999–2015
Source: Virus Evol. 2019 Jul 21;5(2):vez020. doi: 10.1093/ve/vez020 (PMC6642743; doi:10.1093/ve/vez020)
Supplement: vez020_Supplementary_Data [file vez020_supplementary_data.zip › Supplementary Table 3.docx]

**Supplementary Table 3.** dN/dS values by gene and host for West Nile virus in NYS.

|  | **Avian** | **Mosquito** | **Combined** |
| --- | --- | --- | --- |
| n = | 287 | 245 | 532 |
| *C* | 0.373 | 0.594 | 0.477 |
| *prM* | 0.064 | 0.107 | 0.084 |
| *E* | 0.074 | 0.066 | 0.070 |
| *NS1* | 0.085 | 0.092 | 0.088 |
| *NS2A* | 0.093 | 0.114 | 0.103 |
| *NS2B* | 0.059 | 0.051 | 0.055 |
| *NS3* | 0.055 | 0.053 | 0.054 |
| *NS4A* | 0.108 | 0.087 | 0.098 |
| *NS4B* | 0.107 | 0.175 | 0.139 |
| *NS5* | 0.057 | 0.054 | 0.055 |
